# π‐Radical Cascades to *Peri*‐Fused Triangulene Dimers

**DOI:** 10.1002/anie.6610209

**Published:** 2026-05-13

**Authors:** Paula L. Widmer, Leoš Valenta, Maximilian Mayländer, Jules Hutter, Francis J. Carta, Simon Jurt, Olivier Blacque, Laurent Bigler, Sabine Richert, Tomáš Šolomek, Michal Juríček

**Affiliations:** ^1^ Department of Chemistry University of Zurich Zurich Switzerland; ^2^ Institute of Physical Chemistry University of Freiburg Freiburg Germany; ^3^ Institute of Physical Chemistry II Ulm University Ulm Germany; ^4^ Van't Hoff Institute for Molecular Sciences University of Amsterdam Amsterdam the Netherlands; ^5^ Prievidza Chemical Society Prievidza Slovakia

**Keywords:** π‐radical cascade, non‐Kekulé hydrocarbon, *peri*‐fusion, steric effects, triangulene

## Abstract

Open‐shell molecular graphene fragments represent versatile synthons of graphene‐based carbon nanostructures because of their ability to undergo multi‐step π‐radical cascades that enable the formation of multiple bonds and rings in a single step. However, the use of graphene‐based π‐radicals in synthesis remains limited due to our incomplete understanding of their reactivity. This limitation primarily arises from the inherent difficulty of controlling reactions involving multiple reactive centers, as is the case with π‐delocalized radicals. To address this challenge and advance research on π‐radical reactivity, we establish reaction control in a system that can formally feature multiple unpaired π‐electrons. Specifically, we examine oxidative *peri*‐fusion of the dihydro‐precursor of the prototypic non‐Kekulé hydrocarbon triangulene. By investigating the reactive intermediates that dictate selectivity, we demonstrate that monoradical, rather than diradical, intermediates play a key role. Through the precise placement of steric bulk around the periphery, we modulate reactivity at specific positions, steering selectivity toward doubly or singly *peri*‐fused dimeric products. Our study demonstrates that, when controlled, the reactivity of open‐shell molecular graphene fragments can serve as a step‐economic and synthetically valuable tool.

## Graphical abstract

We demonstrate the potential of open‐shell molecular graphene fragments as synthons for constructing graphene nanostructures via π‐radical cascades. By identifying monoradical intermediates as key selectivity‐determining species and tuning steric effects in a triangulene precursor, we show that reactivity in a formally diradical system can be rationally guided and controlled, establishing principles extendable to multiradical systems.

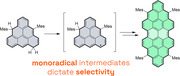

## 1 Introduction

The infinite structural space of graphene‐based carbon nanostructures inspires chemists to push the limits of synthetic methodologies, unlocking exciting opportunities in energy research and nanotechnology [1, 2, 3]. However, despite substantial progress in the bottom‐up synthesis of small molecular graphene fragments [4, 5, 6, 7], their extension into larger structures remains a formidable challenge in solution‐phase synthesis [8, 9, 10]. Therefore, there is a need for developing transformative methods that streamline the synthesis of complex polycyclic aromatic frameworks with precise size, shape, and edge topology by reducing the number of synthetic steps. Such advances would also expand the toolbox of existing synthetic methods like the Scholl reaction [11], metal‐catalyzed reactions [12, 13], and annulative π‐extension [14, 15].

### 1.1 π‐Radical Cascades

A promising approach involves π‐radical cascades of hydro‐precursors of open‐shell graphene fragments, which, under oxidative conditions, enable the formation of multiple bonds and rings in a single step [16]. The use of open‐shell synthons, however, has been limited, as the field primarily focuses on their properties that arise from the presence of unpaired π‐electrons [17]. Consequently, the reactions of these π‐radicals are rarely studied, as they are commonly viewed as undesirable decomposition pathways. Yet, radical reactions in general are among the fastest and most efficient tools in the synthetic arsenal of organic chemists, despite being challenging to control due to a low reaction selectivity and a low kinetic stability of the reactants. In the case of π‐radicals, unpaired electrons delocalize over multiple sites of the conjugated backbone, making reaction control and reagent handling even more delicate. Nevertheless, there is significant potential in harnessing the efficient nature of π‐radical cascade reactions, provided that strategies for achieving selectivity, such as introducing steric bulk to block undesired positions or preorganization of reactive centers, are employed.

### 1.2 Phenalene: A Benchmark System

**FIGURE 1:**
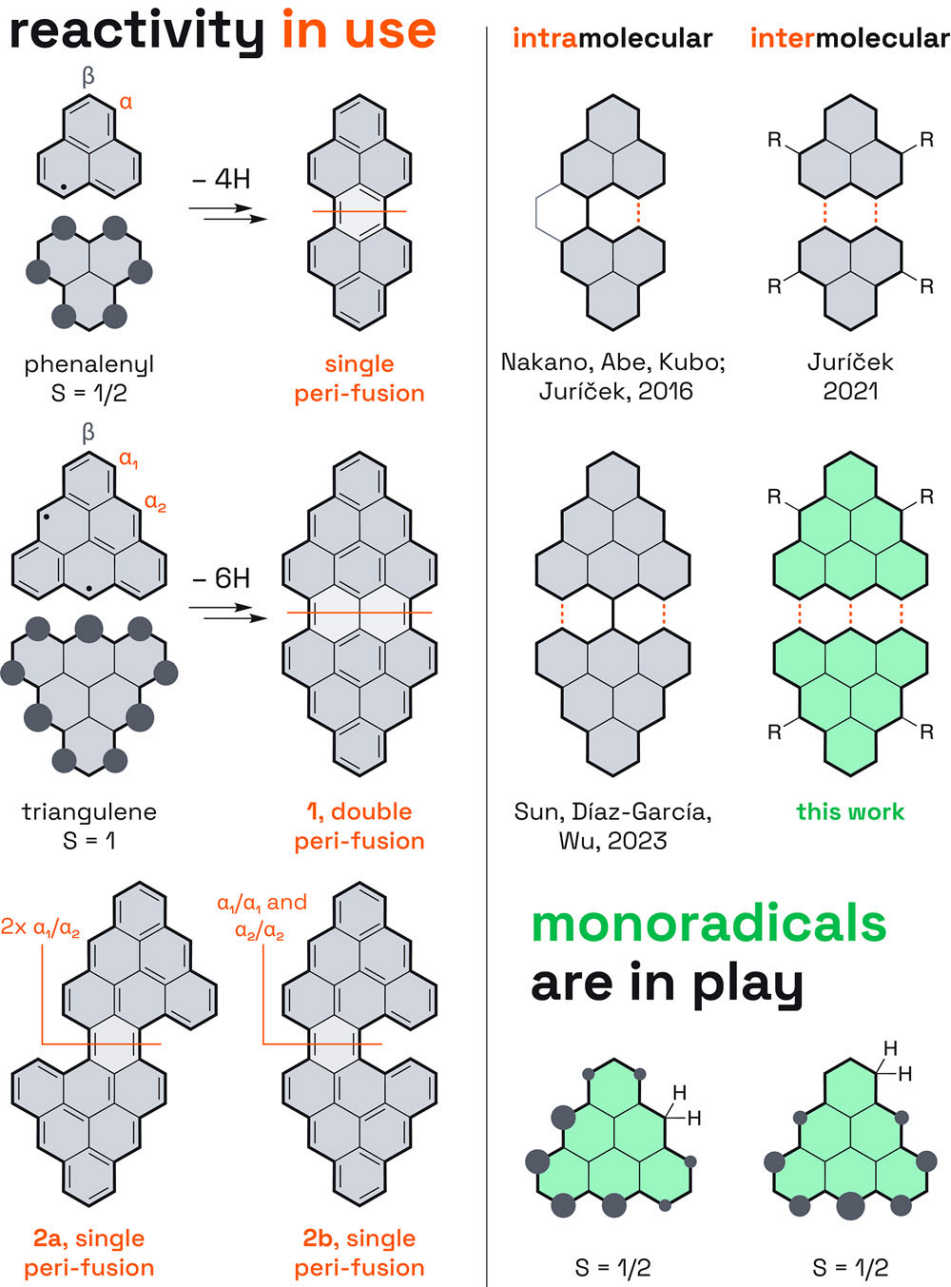
Through the lens of methodology. (left) Reactivity of graphene‐fragment π‐radicals as a useful synthetic tool for the *peri*‐fusion of zigzag edges. (top right) Intra‐ versus intermolecular fusion: selectivity control by pre‐organization or steric effects. (bottom right) Key intermediates: two isomers of hydrotriangulene monoradicals; positive spin density is indicated by gray circles.

The smallest open‐shell molecular graphene fragment, phenalenyl radical, exemplifies this potential (Figure 1). Under oxidative conditions, two molecules of its hydro‐precursor, phenalene, undergo a π‐radical cascade to form peropyrene—a *peri*‐fusion process in which two zigzag edges are “sewn together” in a five‐step sequence that includes the formation of phenalenyl from phenalene, followed by σ‐dimerization, oxidation, electrocyclization, and aromatization. As a net result, two σ‐bonds, one π‐bond, and a new ring are formed, accompanied by the formal loss of six hydrogen atoms (–4H when counted from the phenalenyl radical) [18].

The synthetic utility of this reaction has been demonstrated by us and others both intermolecularly, using steric effects, and intramolecularly, through preorganization. Intermolecular *peri*‐fusion of two phenalene (sub)units has been employed in the preparation of structurally complex contorted hydrocarbons, such as cycloparaphenylene (CPP) double nanohoops [19, 20] (Figure 1, R,R = CPP) and hypercethrene, a hydrocarbon featuring one peropyrene unit, two [7]helicene units, and two sp^3^ quaternary centers [21]. In the latter case, the phenalene cascade was part of a more elaborate sequence that, remarkably, involved the formation of four σ‐bonds, one π‐bond, and three new rings over the course of up to nine steps, accompanied by a formal loss of 10 hydrogen atoms. The intramolecular variant of the phenalene cascade relies on the pre‐connection of the phenalene units [18, 22, 23] or thereof derived benzophenalene [24], cyclopentadiene‐fused rylene [25], olympicene [26], or analogous indenothiophene [27] units. Depending on the linking mode, the system undergoes either a full π‐radical cascade (–6H) when the units are linked via β‐positions [22] or a partial cascade (–4H) when the units are linked via α‐positions. In the latter case, the initial oxidation generates diradicaloid systems with partial open‐shell character, like biphenalenylidene [18] and cethrene [23], which also appear as intermediates in the corresponding intermolecular processes. While the advantage of the intramolecular process over the intermolecular one lies in eliminating concerns about selectivity, it requires the preorganization of molecules through at least one pre‐formed C─C bond, thereby increasing the required synthetic effort.

### 1.3 Dihydrotriangulene: Beyond Single *Peri*‐fusion

To test the robustness of π‐radical cascade methodology, we turned our attention to more complex systems that can formally feature two unpaired electrons [28, 29, 30]. As our model, we selected the dihydro‐precursor of triangulene, the next homolog in the series of open‐shell triangular graphene fragments after phenalenyl. What makes *peri*‐fusion of dihydrotriangulene more complex compared to phenalene is the potential formation of three different products: one doubly *peri*‐fused (**1**) and two singly *peri*‐fused (**2a** and **2b**, Figure 1). In the double‐fusion pathway, three σ‐bonds, two π‐bonds, and two rings are formed, with a formal loss of 10 hydrogen atoms. In comparison, the single‐fusion pathway forms two σ‐bonds, two π‐bonds, and one ring, accompanied by a formal loss of eight hydrogen atoms, with **2b** representing the penultimate intermediate of the double‐fusion process. Moreover, the potential presence of two unpaired electrons in triangulene raises the prospect of competing oligomerization alongside *peri*‐fusion.

To date, the *peri*‐fusion of dihydrotriangulene units has been achieved exclusively through intramolecular approaches [31], where one less σ‐bond needs to be formed. By pre‐linking dihydro‐triangulene units at their α_2_‐positions and incorporating six substituents, a doubly *peri*‐fused product was synthesized [32, 33, 34], a strategy also employed in the synthesis of [4]rhombene [35]. Linkage of multiple triangulene units in an α–β fashion on the Au(111) surface enabled the formation of graphene nanoribbons with triangulene units fused via a five‐membered ring [36]. In these cases, selectivity was inherently ensured by pre‐connecting the reactive positions, thereby dictating the final product. In an intermolecular setting [37, 38], however, selectivity depends on the σ‐dimerization step of the cascade [21], making it crucial to understand the nature of the radical intermediates.

In contrast to previous studies on intramolecular processes (where a tetraradical is proposed [32] due to the presence of two pre‐connected dihydrotriangulene units), our present findings reveal that monoradical species act as key intermediates in the intermolecular pathway. This finding has significant implications for the design and application of π‐radical cascades, with several factors becoming critical: (1) multiple isomers of dihydrotriangulene can give rise to different monoradical intermediates, (2) unlike phenalenyl, where all six α‐positions exhibit equal reactivity, hydrotriangulene mono‐radicals display an uneven spin‐density distribution (Figure 1), resulting in position‐dependent reactivity, and (3) the steric effects of substituents, which are crucial for selectivity control, further increase complexity by influencing the number and nature of monoradical isomers.

The insights offered here clarify the underlying mechanism and demonstrate how strategically placed substituents can guide selective reactions. This not only deepens our fundamental understanding of π‐radical cascades but also expands their potential as a synthetic methodology.

## 2 Results and Discussion

### 2.1 Synthesis

**SCHEME 1:**
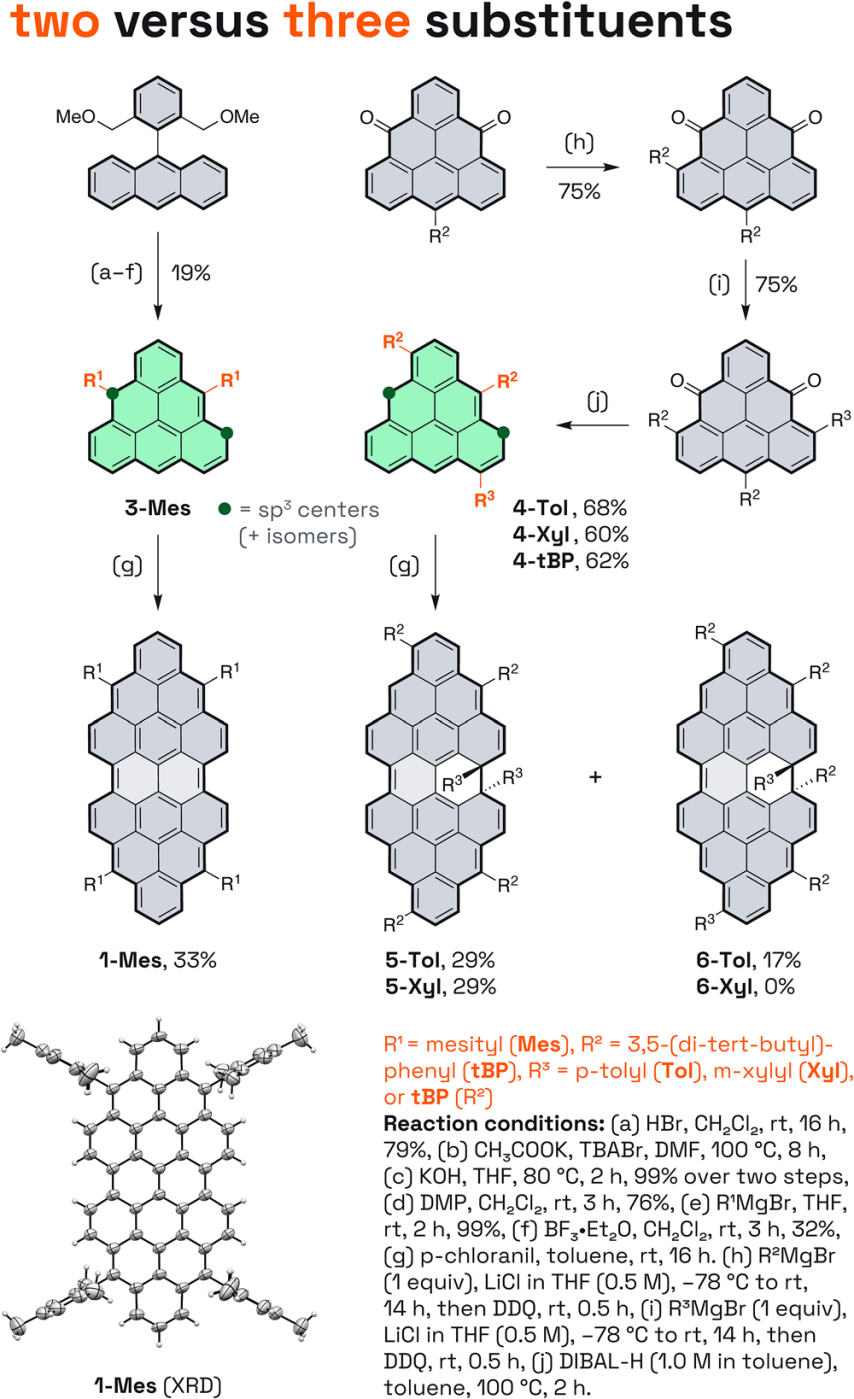
The model systems. Synthesis of dihydro‐precursors **3** and **4** equipped with two and three substituents, respectively, and the products of their oxidative *peri*‐fusions, including the solid‐state structure of **1‐Mes** [39]. Structures of all formed isomers of **3** and **4** are shown in Figures 2 and 5, respectively (for a full overview, see Figures S1 and S2).

The starting substrates for the oxidative *peri*‐fusion studies were dihydro‐precursors of triangulene, which contain two sp^3^ centers. Two model systems were employed: a disubstituted (**3**) and a trisubstituted (**4**) variant of dihydrotriangulene (Scheme 1). In **3**, two edges are blocked by bulky substituents at their centers (α_2_‐positions), while one edge remains fully exposed. In **4**, all three edges are blocked: one fully with a bulky substituent at the α_2_‐position and the other two partially, each with one bulky or semi‐bulky substituent at the α_1_‐position.

**FIGURE 2:**
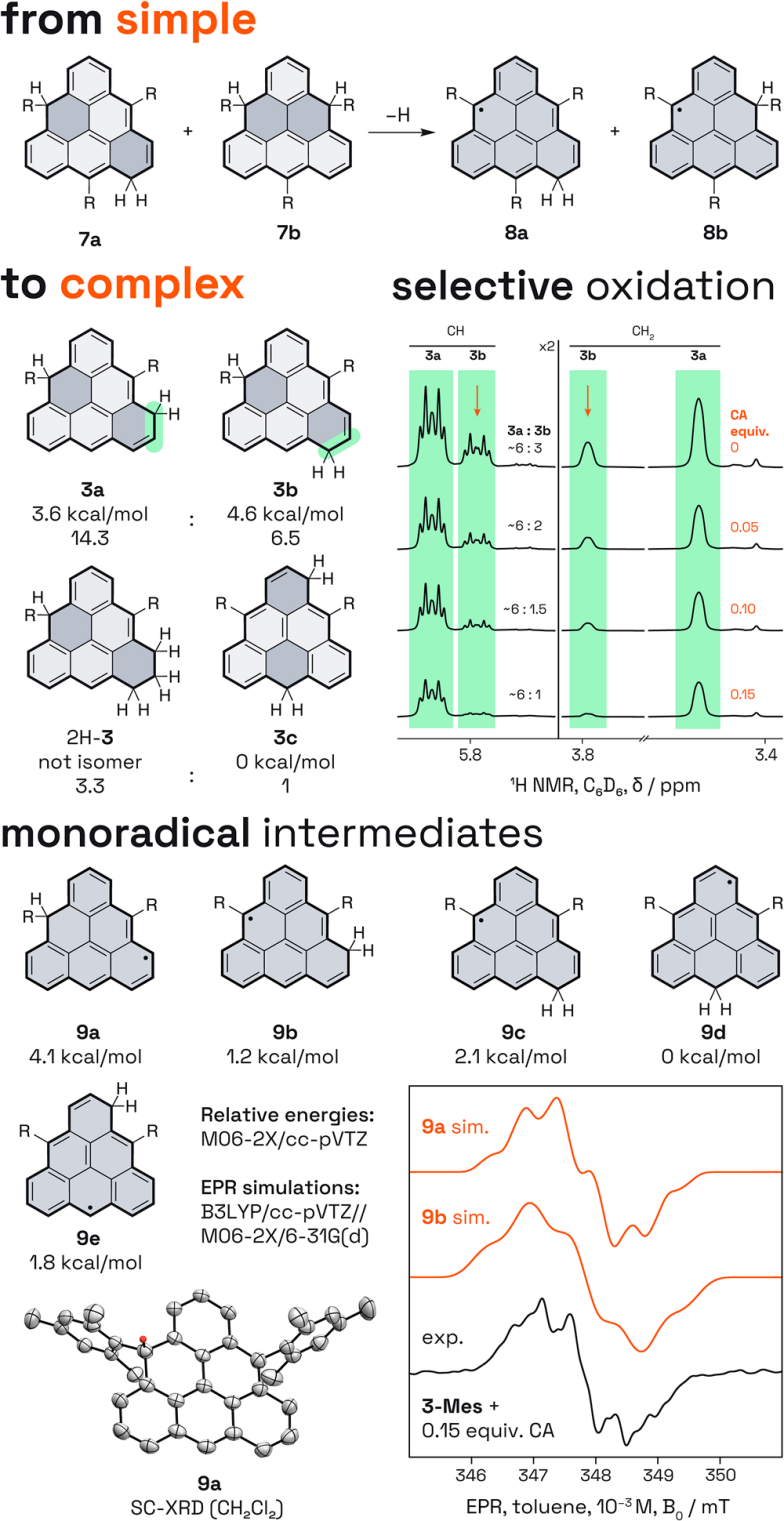
The monoradical scenario. Analysis of the possible dihydro‐precursors and monoradical species they can form, aided by NMR and EPR spectroscopy, and XRD. The helicene and phenylanthracene subunits in **3**, 2*H*‐**3**, and **7** are highlighted with light gray. In the solid‐state structure of **9a**, hydrogen atoms are omitted for clarity, except for C(sp^3^)–*H* highlighted in red, and thermal ellipsoids are shown at a 50% probability level. **7**, **8**: R = H or mesityl; **3**, 2*H*‐**3**, **9**: R = mesityl. CA = *p*‐chloranil. The numerical values listed below the calculated energies represent the relative isomer ratios between the observed compounds **3**.

The dihydro‐precursor of triangulene equipped with two mesityls as the R^1^ blocking groups (**3‐Mes**) was prepared using a strategy similar to that employed previously for trimesityl‐triangulene [28]. Starting from 9‐(2,6‐bis(methoxymethyl)phenyl)anthracene that can be accessed in one step from the commercial 9‐bromoanthracene, the disubstituted precursor **3‐Mes** was prepared over six steps with 19% overall yield (for full synthetic sequence, see Scheme S1). The 2D NMR analysis revealed that **3‐Mes** is a mixture of three isomeric dihydro‐precursors **3a–c**, along with 13% of the tetrahydro‐derivative 2*H*‐**3**, which results from the overreduction of **3a** and **3b** (see Figure 2). Through in‐depth 2D NMR spectroscopic analysis, including the full assignment of proton and carbon resonances for the major species **3a**, **3b**, and 2*H*‐**3**, the precise positions of the sp^3^ carbon atoms in these isomers were identified (see the Supporting Information).

The dihydro‐precursors of triangulene equipped with three blocking groups (**4**) were prepared following a previously reported strategy of nucleophilic additions to triangulene‐4,8‐dione [40]. The reaction conditions were slightly modified compared to the original procedure to improve the efficiency, namely, we employed a turbo version [41] of Grignard reagents (RMgBr·LiCl) and 2,3‐dichloro‐5,6‐dicyano‐1,4‐benzoquinone (DDQ) instead of I_2_/O_2_ for the oxidation of the formed enol intermediates. The stepwise nature of these additions allowed for easy modulation of the nucleophile size, enabling the preparation of three variants of **4** with varying sizes of R^3^, while keeping R^2^ constant (R^2^ = 3,5‐di‐*tert*‐butylphenyl): **4‐Tol**, R^3^ = *p*‐tolyl; **4‐Xyl**, R^3^ = *m*‐xylyl; and **4‐tBP** [40], R^3^ = R^2^ = 3,5‐di‐*tert*‐ butylphenyl). The final reduction of the diketo intermediates with DIBAL‐H yielded an isomeric mixture of dihydro precursors **4** (Figure 5), similar to **3**.

### 2.2 Products of the Cascades

Oxidation of **3‐Mes** with 5 equivalents of *p*‐chloranil, formally removing 10H, yielded the doubly *peri*‐fused product **1‐Mes** (Scheme 1), isolated in 33% yield as the sole product. Neither of the possible singly fused dimers, **2a** or **2b** (Figure 1), was observed. For **4**, double fusion to a fully conjugated sp^2^ system is not possible because each edge is blocked by a substituent. Of the two singly fused products, only **2b** appears to have formed; however, the isolated product was not **2b** itself but a product resulting from its 6π electrocyclization, analogously to related systems such as cethrene [23, 42, 43] and biphenalenylidene [18]. The size of the peripheral substituents affects both the steric availability of reactive positions and the orientation of the molecules during σ‐dimerization, ultimately influencing the reaction outcome. When R^3^ = R^2^ = 3,5‐di‐*tert*‐butylphenyl, no fused product was observed due to excessive bulk disrupting the cascade (see discussion below, section 2.6). In contrast, a singly fused electrocyclized product, **5‐Xyl**, formed in 29% yield when R^3^ = *m*‐xylyl. Interestingly, a mixture of symmetric (**5‐Tol**, 29% yield) and non‐symmetric (**6‐Tol**, 17% yield) products was obtained with the smallest substituent, R^3^ = *p*‐tolyl.

### 2.3 Initial Considerations

To rationalize the observed product formation, it is key to understand the factors that govern the selectivity of the π‐radical cascade. This requires determining the nature of the key intermediates—monoradical versus diradical—as each pathway has distinct implications for the reaction's complexity.

1. Pristine triangulene has two possible dihydro‐precursors: **7a** with a [4]helicene subunit and **7b** with a phenylanthracene subunit (Figure 2, top; R = H). The same applies to triangulene bearing three mesityl groups at the edge centers, except that in this case, **7b** has two possible stereoisomers (*syn*/*anti* orientation of the R groups). Previous studies have shown that oxidizing the dihydro‐precursor **7** with *p*‐chloranil occurs stepwise, first forming a monoradical, then triangulene diradical [28]. Regardless of R (H or mesityl), two monoradical intermediates (**8a** and **8b**) are possible, while symmetry allows only a single diradical. This demonstrates that even in symmetrically substituted dihydro‐precursors, monoradical intermediates introduce greater complexity than diradicals.
2. For **3**, six dihydro‐precursors exist in total. Three possess a helicene subunit (**3a**–**c**, Figure 2, middle) and three a phenylanthracene subunit (*syn*/*anti*‐**3d** and **3e**, Figure S1). This mixture can generate up to five isomeric monoradicals **9a–e** (Figure 2, bottom), whereas only a single diradical species is possible. This contrast, compared to the symmetric cases, underscores the need to identify the intermediates of the cascade to elucidate the selectivity rules of the *peri*‐fusion process.

### 2.4 Two Substituents

Out of the six isomers of **3‐Mes**, only **3a–c** were isolated as a mixture (Figure 2, middle), each featuring a helicene subunit. Although these isomers are more stable than those with a phenylanthracene subunit (Tables S1 and S2), the mixture is predominantly composed of the helicene precursors **3a** and **3b** that are less stable than **3c**. This suggests that their formation by reduction (Scheme S1) is kinetically controlled, similar to the case of the trisubstituted analog **7** (R = mesityl) [44]. Additionally, the mixture contains a tetrahydro‐precursor, 2*H*‐**3**, formed by overreduction of **3a** and **3b**. Precursors **3a** and **3b** together account for over 80% of the mixture. Therefore, the subsequent discussion focuses on them, as they sufficiently capture the key aspects.

Oxidation of **3a** and **3b** can yield three monoradicals (Figure 2, bottom). Monoradical **9a** can be obtained from both **3a** and **3b** through hydrogen abstraction from the CH_2_ group, whereas hydrogen abstraction from the CHR group (R = mesityl) yields **9b** from **3a** and **9c** from **3b** (Figure 2). The least hindered C(sp^3^)─H bond is the CH_2_ group in **3b** [45]. This position is the most likely site for hydrogen abstraction during oxidation. Indeed, the addition of substoichiometric amounts of *p*‐chloranil in benzene‐*d*_6_ results in a clearly faster, though not exclusive, consumption of **3b** compared to **3a** when monitored by NMR spectroscopy [46], supporting this hypothesis (Figure 2, middle right). Notably, **3b** has nearly disappeared using 0.15 equivalents of *p*‐chloranil, which corresponds well with its ∼26% content in the mixture (*p*‐chloranil abstracts 2×H). We therefore conclude that **3b** converts primarily to **9a**. In the case of **3a**, we could not distinguish which sp^3^ center undergoes faster hydrogen abstraction. Consequently, formation of both **9a** and **9b** is plausible. Together, these observations suggest that **9a** and **9b** are the predominant monoradicals generated from the **3‐Mes** mixture under the applied oxidation conditions. To experimentally identify the monoradical species present, the CH_2_Cl_2_ solution of the **3‐Mes** mixture without *p*‐chloranil was slowly evaporated under air at room temperature, yielding single crystals [39]. X‐ray diffraction revealed that these crystals belong to monoradical **9a** (Figure 2, bottom), consistent with **9a** being one of the predominant species under these conditions. Computational analysis predicts **9a** to be ∼3 kcal/mol less stable than **9b** and ∼4 kcal/mol less stable than the most stable monoradical **9d** (Table S6). This implies that **9a** is a kinetically trapped product that does not interconvert to the thermodynamically favored **9d**. Had such interconversion occurred, *peri*‐fusion would not take place, as all reactive positions in **9d** are blocked by mesityl groups. Further insight into the monoradical composition was obtained via electron paramagnetic resonance (EPR) spectroscopy. An aliquot was taken from the reaction mixture 10 min after the addition of 0.15 equivalents of *p*‐chloranil and analyzed by EPR, at which point the system appeared to have reached a steady composition (see Notes [46] for details). This titration point was chosen because it coincided with the near‐complete disappearance of **3b**, while **3a** was still observable (see Figure 2 and the corresponding discussion above). The resulting spectrum reveals a mixture of monoradicals (Figure 2). No EPR signature of a diradical could be detected. Simulations of the EPR spectra of the possible monoradicals suggest that the mixture includes **9a** and **9b** as major isomers, along with minor species derived from 2*H*‐**3** and **3c** (EPR simulations of **9c–e** are shown in Figure S3). The presence of these minor monoradicals can be attributed to the formation of σ‐dimers from **9a** and **9b** (see below), with EPR detecting both the minor species and any nondepleted population of **9a** and **9b**.

### 2.5 Formation of σ‐Dimers

**FIGURE 3:**
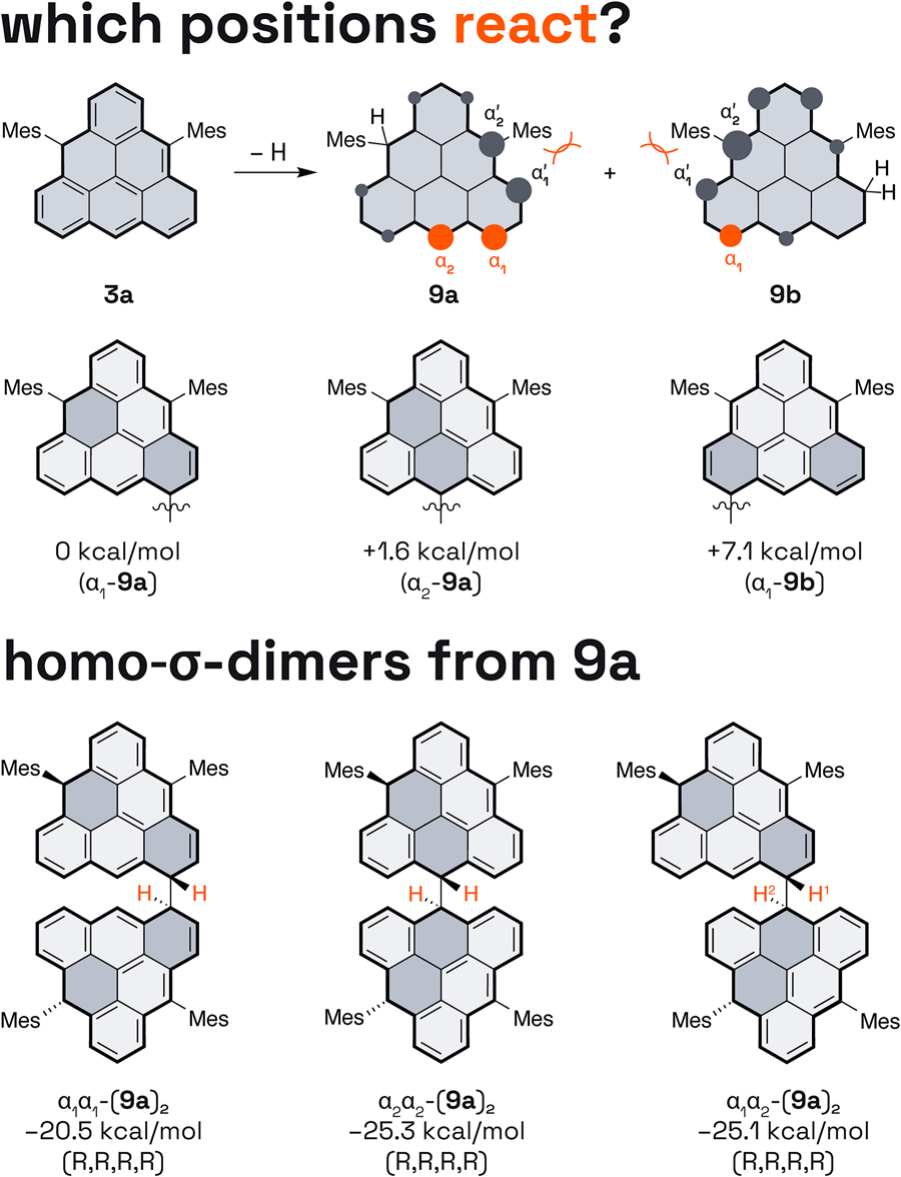
The σ‐dimer analysis. (top) Variation in electronic stabilization depending on the position of the C─C bond during σ‐dimer formation from monoradicals **9a** and **9b**. The displayed values represent the relative energies of the corresponding dihydro‐precursors (Figure S1 and Table S1). (bottom) Possible homo‐σ‐dimers derived from **9a**, with the respective σ‐dimer formation enthalpies for their (*R*,*R*,*R*,*R*)‐stereoisomers. Shown values were calculated using M06‐2X/cc‐pVTZ. Sterically blocked and sterically available positions are highlighted with gray and orange circles, respectively.

The second step in the cascade, σ‐dimer formation, plays a crucial role in determining selectivity. The predominance of monoradicals **9a** and **9b** narrows down the possible combinations of σ‐dimers. Monoradical **9a** has two sterically accessible reactive positions, α_1_ and α_2_ (Figure 3, top, orange). Formation of a C─C bond via these positions results in σ‐dimers that contain either a helicene or a phenylanthracene subunit (highlighted with light gray), both of comparable stability (1.6 kcal/mol difference).

Positions α′_1_ and α′_2_ are blocked by the proximity of the mesityl group, preventing radical dimerization, although the corresponding σ‐dimers also possess helicene and phenylanthracene subunits (Figure S4a). All other positions in **9a** with appreciable π‐electron density are irrelevant, as their participation would lead to significant thermodynamic σ‐dimer destabilization (Figure S4b). Similar reasoning for **9b** suggests that only position α_1_ is likely to participate in the σ‐dimerization (Figure 3, top, orange), even though it results in a pyrene and not the helicene or phenylanthracene subunits (highlighted with light gray) in the dimer. This destabilizes the σ‐dimer by 5–7 kcal/mol per subunit. Hence, the most stable σ‐dimers are the three possible homo‐σ‐dimers derived from **9a**—α_1_α_1_‐(**9a**)_2_, α_1_α_2_‐(**9a**)_2_, and α_2_α_2_‐(**9a**)_2_ (Figure 3, bottom), while the less stable σ‐dimers include homo‐σ‐dimer α_1_α_1_‐(**9b**)_2_ and two hetero‐σ‐dimers, α_1_α_1_‐(**9a**)(**9b**) and α_2_α_1_‐(**9a**)(**9b**) (Figure S5).

**FIGURE 4:**
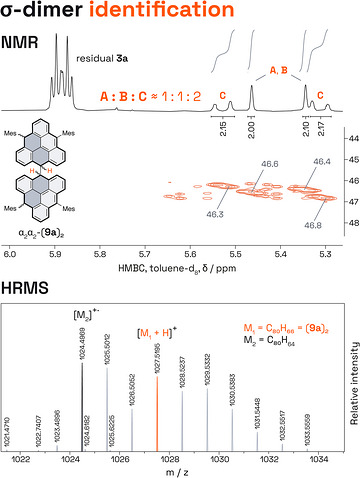
Identification of σ‐dimers. (top) The ^1^H–^13^C HMBC NMR spectrum acquired on a sample of **3‐Mes** after addition of 0.45 equivalents of *p*‐chloranil in toluene‐*d*_8_. (bottom) The APPI‐HRMS analysis of the mixture used for the HMBC measurement showed interfered signals, which were deconvoluted and assigned. The two most prominent species, [C_80_H_66_ + H]^+^ (corresponding to the σ‐dimer, *M*_1_, orange) and [C_80_H_64_]^+•^ (*M*_2_, black), are highlighted as their monoisotopic signals.

To investigate whether σ‐dimers could be observed, we monitored the mixture of **3‐Mes** by ^1^H NMR spectroscopy and mass spectrometry as oxidant *p*‐chloranil was added in portions. Upon reaching 0.45 equivalents, an amount insufficient to oxidize all **3‐Mes** to monoradicals, new signals appeared between 5.3–5.6 ppm in the ^1^H NMR spectrum (toluene‐*d*_8_), indicative of the formation of three distinct σ‐dimers: A, B, and C (Figure 4, top).

**SCHEME 2:**
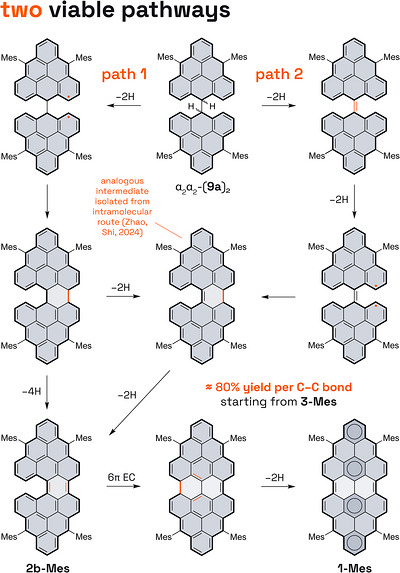
Two plausible pathways. (path 1) After σ‐dimerization, the sp^3^ centers of the dihydrotriangulene units with mesityl substituents undergo hydrogen abstraction, followed by σ‐bond formation, successive oxidation to two double bonds, electrocyclization, and final oxidation. (path 2) After σ‐dimerization, the newly formed σ‐bond oxidizes to a double bond, then the sp^3^ centers undergo hydrogen abstraction, σ‐bond formation, oxidation to a double bond, with the final steps identical to path 1.

The on‐flow atmospheric‐pressure photoionization (APPI) high‐resolution mass spectrometry (HRMS) analysis of the same sample showed mixtures of unresolved ions (Figure 4, bottom). Therefore, the spectra were deconvoluted by mean‐square‐error (MSE) minimization, using theoretical isotope distributions of applicable chemical formulas as exemplified in Figure S6. Best MSE matches were found for two types of ions, [*M*]^+•^ and [*M* + H]^+^, wherefore [C_80_H_66_ + H]^+^ and [C_80_H_64_]^+•^ were the most prominent molecular ions (Figures 4, S6, and S7). The first signal corresponds to the σ‐dimer, while the latter arises from the oxidized species (–2H), formed either directly from the σ‐dimer (Scheme 2, top right, top left, or middle left) or from σ‐dimers of diradical species that could be present in small amounts. To exclude in‐source generation of these species, stability of the products was tested by measuring at two different collision cell energies, 10 (Table S3) and 70 eV (Table S4), with no significant changes in the proportion of identified molecular ions found (Figure S8).

To identify the structure of the σ‐dimers, HMBC (Figure 4, top) and COSY (Figure S9) NMR spectra were recorded. A characteristic feature of the σ‐dimers is the HMBC correlation between the hydrogen atom attached to the sp^3^ carbon atom of the linking σ‐bond in one unit (Figure 4, highlighted in orange) and the sp^3^ carbon atom of the σ‐bond in the second unit, observed across two bonds. This correlation appears for all three σ‐dimers in the spectrum (Figure 4, top) and is consistent with previous observations [21]. The COSY spectrum narrows the six possible σ‐dimer structures to a single option, α_2_α_2_‐(**9a**)_2_, as there is no correlation of the C(sp^3^)–*H* signals to the C(sp^2^)–*H* signals in the double bond region (∼5.8–6.7 ppm), observed for dihydro‐precursors **3a** and **3b**. Finally, the multiplicity of the observed signals—two singlets and a set of doublets—indicates the presence of two symmetric (A, B) and one non‐symmetric (C) σ‐dimer.

The α_2_α_2_‐(**9a**)_2_ σ‐dimer can, in principle, give rise to eight diastereomers, but configurational degeneracy arising from symmetry‐related stereocenters reduces this number to six. Based on DFT calculations (Table S5 and Figures S10–S13), we assign signals A and B to the nearly isoergic diastereomers (*S,S,S,S*)‐α_2_α_2_‐(**9a**)_2_, and (*R,S,R,S*)‐α_2_α_2_‐(**9a**)_2_. While the former diastereomer adopts a conformation with *C*_2_ symmetry, the latter formally represents an achiral *meso* form of α_2_α_2_‐(**9a**)_2_. Although the lowest energy conformer of *meso*‐(*R,S,R,S*)‐α_2_α_2_‐(**9a**)_2_ lacks *C*_s_ symmetry, rapid rotation around the α_2_α_2_ σ‐bond interconverts conformers in a manner that time‐averages the NMR resonances, producing a spectrum as observed for the *C*_2_‐symmetric (*S,S,S,S*)‐α_2_α_2_‐(**9a**)_2_. The remaining signals belong to C, the (*R,S,R,R*)‐α_2_α_2_‐(**9a**)_2_ diastereomer destabilized by ∼5 kcal/mol. The A–C diastereomers correspond to the three most stable structures among the six possible diastereomers of α_2_α_2_‐(**9a**)_2_. Their higher stability arises from their ability to adopt an eclipsed conformation that maximizes dispersion interactions between individual subunits of the σ‐dimer. The remaining diastereomers can only adopt staggered conformations due to steric constraints dictated by the position and the configurations of the mesityl substituents. The observed ratio of A:B:C ∼ 1:1:2 (Figure 4, top) suggests a statistical distribution, consistent with σ‐dimerization occurring under kinetic control, wherein the less stable, kinetically labile staggered diastereomers gradually convert into the more stable, kinetically trapped eclipsed diastereomers that are observed or subsequent intermediates of the cascade.

Interestingly, DFT calculations do not definitively establish whether σ‐dimer formation from monoradicals **9‐Mes** is thermodynamically or kinetically controlled, as the computed formation energies vary significantly with the used functional and its ability to account for dispersion. However, experimental observations point toward kinetic control, which aligns well with predictions from the M06‐2X and MN15 functionals (Table S5). Although some α_1_α_1_‐(**9a**)_2_ and α_1_α_2_‐(**9a**)_2_ σ‐dimers are computed to be nearly as stable as the observed α_2_α_2_‐(**9a**)_2_ (within ∼5 kcal/mol; Figure 3), they are not detected—presumably because they react more rapidly in the subsequent step (path 1 or 2, Scheme 2) and are consumed before α_2_α_2_‐(**9a**)_2_. In addition, while the formation energies of the homo‐ and hetero‐σ‐dimers derived from **9b** still favor σ‐dimerization, they are higher than that of the least stable (**9a**)_2_ σ‐dimer, α_1_α_1_‐(**9a**)_2_, by ∼5–10 kcal/mol, due to the involvement of pyrene subunits and/or less favorable eclipsed/staggered conformations. As a result, although these species may form, they likely remain undetected by NMR spectroscopy due to rapid exchange with monoradical species, facilitated by a lower dissociation energy, and may gradually convert over time into the more stable, kinetically trapped α_1_α_1_‐(**9a**)_2_, α_1_α_2_‐(**9a**)_2_, and α_2_α_2_‐(**9a**)_2_.

Under the preparative conditions with 5 equivalents of *p*‐chloranil, the α_1_α_1_ and α_2_α_2_ linking modes in (**9a**)_2_ ultimately lead to the doubly *peri*‐fused product **1‐Mes**, while the α_1_α_2_ linking mode would yield the singly fused product **2a‐Mes** (Figure 1). The isolated 33% yield of **1‐Mes** is within expectations, especially considering that the previously reported intramolecular approach—which bypasses σ‐dimerization—achieved a yield of 50% and 55% [32]. The absence of the singly fused product **2a‐Mes** suggests that either the reaction cascade does not go to completion or that **2a‐Mes** is unstable, decomposing during work‐up or purification.

These findings underscore the importance of understanding the nature of intermediates, as some can act as “*peri*‐fusion dead‐ends.” Gaining insight into these intermediates could offer strategies to avoid such pitfalls. Additionally, comprehending the reaction mechanism up to the σ‐dimerization step is crucial for effective process control. While several pathways are possible after the σ‐dimerization (following the established mechanism for the *peri*‐fusion of phenalenyl) [18], the selectivity is determined at this stage, and not in the subsequent steps. Two possible pathways, path 1 and path 2, are outlined in Scheme 2, both proceeding through a common intermediate (center), analogous to the one recently isolated in an intramolecular variant [33, 34].

### 2.6 Three Substituents

To validate our understanding of the disubstituted dihydro‐triangulene system, we extended our study to a more complex trisubstituted variant. In this case, double *peri*‐fusion to a fully conjugated sp^2^ system is not possible, as all sides are blocked by a substituent, and the singly *peri*‐fused product **2b** (Figure 1) is expected. Although not directly observed, the formation of products **5** and **6** (Scheme 1) requires their intermediacy, as these represent ring‐closed products from the 6π electrocyclization of **2b**.

**FIGURE 5:**
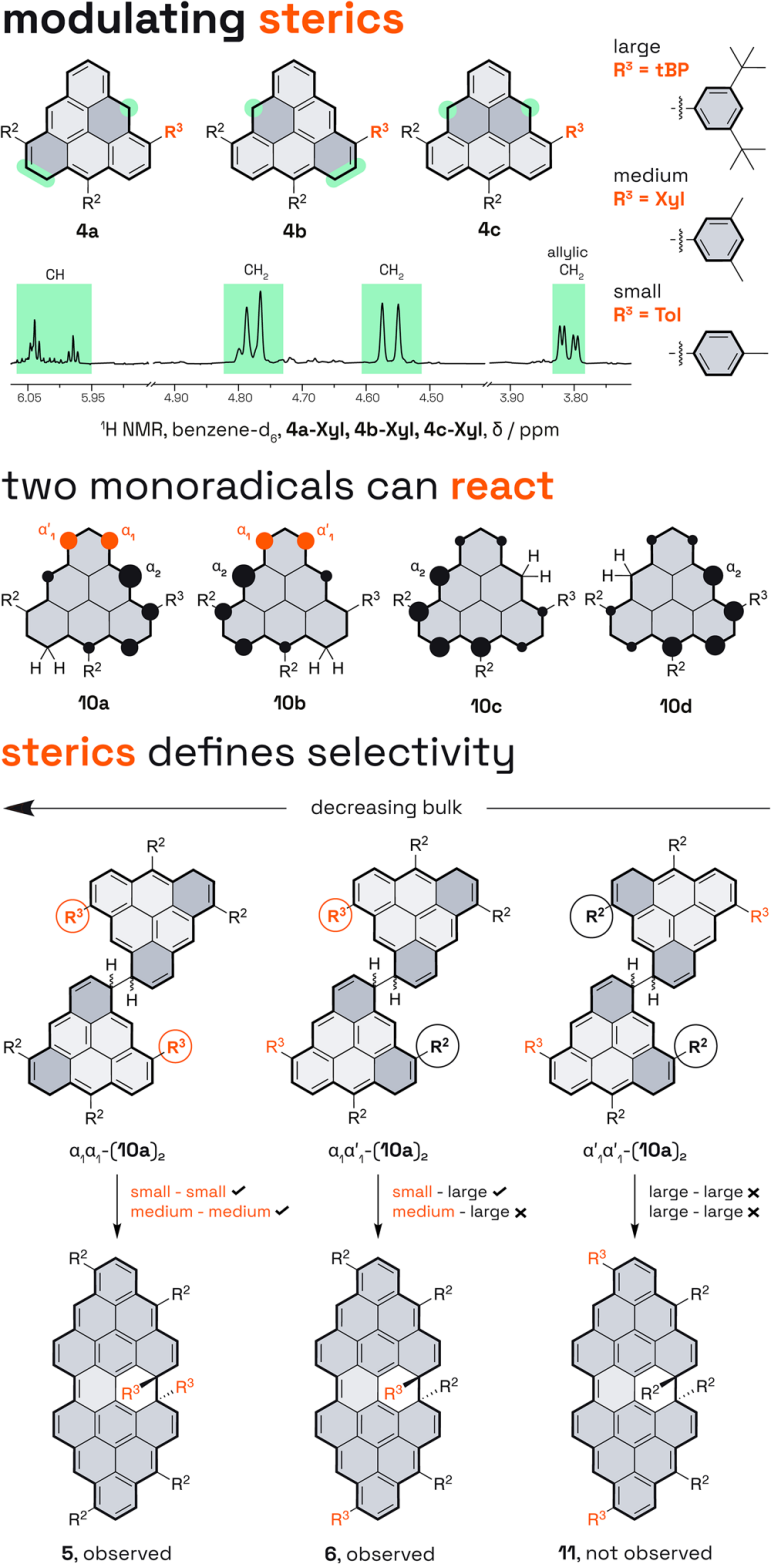
Validating selectivity principles. (top) Observed dihydro‐precursors **4a–c** and the possible monoradical species **10a–d** that they can form. (bottom) Three potential σ‐dimers are illustrated with **10a**, highlighting the relationship between steric bulk and linking mode (bottom), and the corresponding *peri*‐fused products.

For the trisubstituted model system, we identified three distinct isomers of dihydro‐precursors for both **4‐Tol** and **4‐Xyl** (**4a–c**; Figure 5, top), and two isomers for **4‐tBP** (simplified by symmetry, **4a** = **4b**). Oxidation of this mixture leads to four monoradicals for **4‐Tol** and **4‐Xyl** (**10a–d**; Figure 5), while for **4‐tBP**, symmetry reduces the number to two monoradicals (**10a** = **10b** and **10c** = **10d**). Based on electronic and steric considerations similar to those applied earlier, only monoradicals **10a** and **10b** are expected to engage in *peri*‐fusion, with positions α_1_ and α′_1_ available for σ‐dimerization. Monoradicals **10c** and **10d** are deemed unreactive if formed. Positions α_1_ and α′_1_ exhibit comparable electron density, leading to a predicted statistical distribution of σ‐dimers unless steric factors influenced the outcome. To test this hypothesis, we examined the impact of the steric bulk of the R^3^ substituent on the product distribution. Increasing the steric bulk of R^3^ would hinder the σ‐dimerization step or subsequent transformations. Indeed, no dimerized product was observed when R^3^ was a highly bulky 3,5‐di‐*tert*‐butylphenyl group. An intermediate size of R^3^ (*m*‐xylenyl) provided the product **5‐Xyl** derived from the α_1_α_1_‐σ‐dimer. Remarkably, when R^3^ was small (*p*‐tolyl), σ‐dimerization not only occurred with α_1_α_1_ selectivity forming **5‐Tol** but also with α_1_α′_1_ selectivity, yielding the non‐symmetric product **6‐Tol**. The α′_1_α′_1_ combination would lead to product **11** (Figure 5, bottom right), subject to steric constraints comparable to those in the homodimerization of **4‐tBP**, which likewise did not yield an isolable product. These findings highlight how steric effects and reactivity work together to shape the reaction outcome. They build on what we observed in the disubstituted model and strengthen the selectivity principles we established there.

## 3 Conclusion

The use of open‐shell molecular fragments as building blocks for complex carbon‐based nanostructures remains a significant challenge, primarily due to the limited understanding of their reactivity. This work aims to enhance the utility of this approach by shedding light on key aspects of the reaction mechanisms. While the triangulene diradical has been proposed for the intramolecular pathway [32] as an intermediate in the *peri*‐fusion of dihydrotriangulene, our findings demonstrate that the intermolecular reaction proceeds via monoradical intermediates. Combining experimental and computational analyses, we identified multiple dimerization patterns and fusion pathways within the π‐radical cascade, showing that the seemingly complex process can be simplified with a clearer mechanistic picture.

We further showed that steric effects can be used to steer the reaction outcome within the cascade, and that the generation of monoradicals and their subsequent σ‐dimerization appear to proceed under kinetic control—factors that ultimately dictate the observed selectivity. For example, had the oxidative dimerization of dihydrotriangulene proceeded via the most stable monoradical enabled by interconversion between the monoradicals, the dimerization would not have occurred. Hence, harnessing π‐radical cascades remains a promising concept, but further understanding is essential for their successful application. This work represents a step toward unlocking that potential, but it also highlights the need for refining these pathways to fully harness it.

## Author Contributions

**Paula L. Widmer**: writing ‐ original draft, writing ‐ review and editing, visualization, formal analysis, investigation, methodology, data curation, validation. **Leoš Valenta**: conceptualization, investigation, methodology, writing ‐ review and editing, formal analysis, validation, writing ‐ original draft. **Maximilian Mayländer**: investigation, methodology, formal analysis, validation. **Jules Hutter**: investigation, writing ‐ review and editing, methodology, formal analysis, validation. **Francis J. Carta**: methodology. **Simon Jurt**: methodology, formal analysis. **Olivier Blacque**: methodology, formal analysis, validation. **Laurent Bigler**: resources, supervision, formal analysis, writing ‐ review and editing, validation. **Sabine Richert**: investigation, methodology, formal analysis, supervision, resources, writing ‐ review and editing, funding acquisition, validation. **Tomáš Šolomek**: conceptualization, investigation, funding acquisition, writing ‐ original draft, methodology, validation, formal analysis, writing ‐ review and editing, resources. **Michal Juríček**: conceptualization, investigation, funding acquisition, writing ‐ original draft, writing ‐ review and editing, visualization, validation, supervision, resources, project administration, formal analysis, data curation.

## Conflicts of Interest

The authors declare no conflicts of interest.

## Supporting information




**Supporting File 1**: The authors have cited additional references in the Supporting Information [47, 48, 49, 50, 51].

**Supporting File 2**: anie72546‐sup‐0002‐Data.zip.

## Data Availability

The data that support the findings of this study are available in the main text and the Supporting Information of this article. The raw data underlying this study are openly available in the public repository Zenodo at https://zenodo.org/record/17658940 (https://doi.org/10.5281/zenodo.17658940).
