# Supplementary material for: π‐Radical Cascades to Peri‐Fused Triangulene Dimers
Source: Angew Chem Int Ed Engl. 2026 May 13;65(27):e6610209. doi: 10.1002/anie.6610209 (PMC13327587; doi:10.1002/anie.6610209)
Supplement: Supplementary file 1 — Supporting File 1: The authors have cited additional references in the Supporting Information [47, 48, 49, 50, 51]. [file ANIE-65-e6610209-s001.pdf]

Supporting Information

π-Radical Cascades to *Peri*-Fused Triangulene Dimers

Paula L. Widmer,[a]+ Leoš Valenta,[a]+ Maximilian Mayländer,[b] Jules Hutter,[a] Francis J. Carta,[a] Simon Jurt,[a] Olivier Blacque,[a] Laurent Bigler,[a] Sabine Richert,*[b][c] Tomáš Šolomek,*[d][e] and Michal Juríček*[a][e]

[a] P. L. Widmer, Dr. L. Valenta, J. Hutter, F. J. Carta, S. Jurt, Dr. O. Blacque, Prof. L. Bigler, Prof. M. Juríček
Department of Chemistry, University of Zurich, Winterthurerstrasse 190, 8057 Zurich, Switzerland
E-mail: michal.juricek@chem.uzh.ch

[b] Dr. M. Mayländer, Prof. S. Richert
Institute of Physical Chemistry, University of Freiburg, Albertstraße 21, 79104 Freiburg, Germany

[c] Prof. S. Richert
Institute of Physical Chemistry II, Ulm University, Lise-Meitner-Straße 16, 89081 Ulm, Germany
E-mail: sabine.richert@uni-ulm.de

[d] Prof. T. Šolomek
Van't Hoff Institute for Molecular Sciences, University of Amsterdam, PO Box 94157, 1090 GD Amsterdam, The Netherlands
E-mail: t.solomek@uva.nl

[e] Prof. T. Šolomek, Prof. M Juríček
Prievidza Chemical Society, M. Hodžu 10/16, 971 01 Prievidza, Slovakia

+ These authors contributed equally to this work.

Content

1. Supporting Figures S2

2. General Information S21

3. Experimental Procedures S24

4. Copies of the NMR and HRMS Spectra S40

5. Additional Spectra S103

6. X-Ray Crystallography S110

7. Cartesian Coordinates S113

8. Supporting References S124

The raw data underlying this study are openly available in the public repository Zenodo at https://zenodo.org/record/17658940 (DOI: 10.5281/zenodo.17658940).

1. Supporting Figures

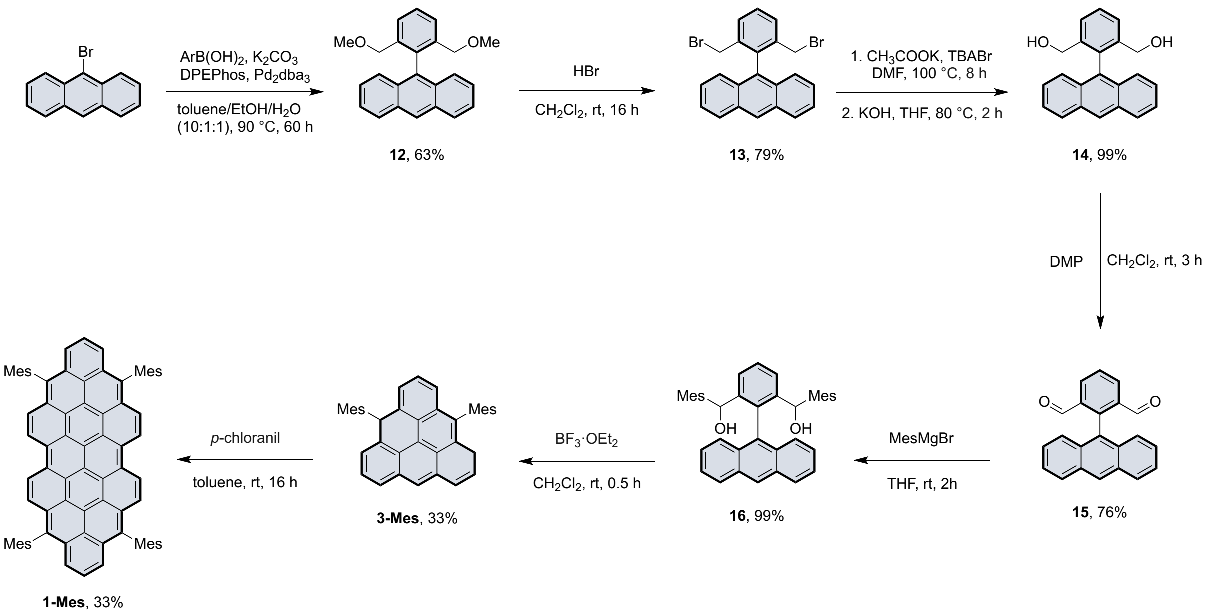

Scheme S1. Synthesis of **1-Mes.** Ar = 2,6-bis(methoxymethyl)phenyl.

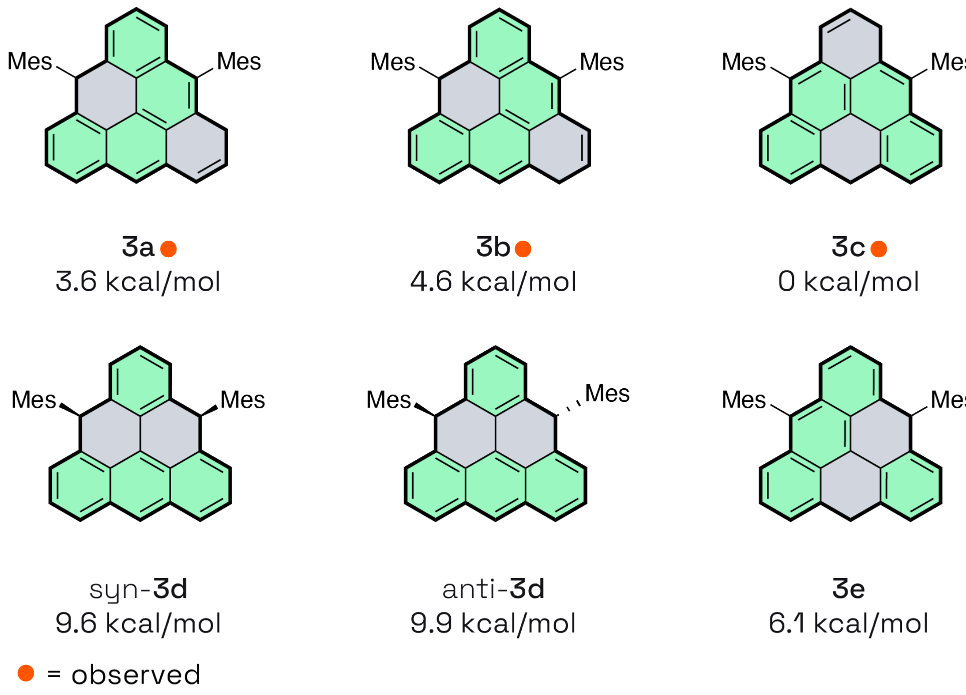

Figure S1. Relative energies of the six isomers of dihydro-precursor **3-Mes**, calculated with DFT/M06-2X/6-31G(d). For a complete set of relative energies with various functionals, see Table S1 below.

Table S1. Relative energies[a] (in kcal/mol) of isomers of dihydro-precursor **3-Mes** shown in Figure S1 calculated with different density functionals.

Precursor	B3LYP	BMK	M06-2X	D3-M06-2X[b]	M06L	MN15
**3a**	5.2	4.1	3.6	3.5	4.0	3.4
**3b**	5.7	4.8	4.6	4.5	4.8	4.7
**3c**	0.0	0.0	0.0	0.0	0.0	0.0
syn-**3d**	12.3	10.6	9.6	9.4	9.3	9.2
anti-**3d**	12.4	10.6	9.9	9.8	9.8	9.5
**3e**	6.9	6.4	6.1	6.1	5.6	6.1

[a]The energies were obtained using cc-pVTZ basis set on the M06-2X/6-31G(d) gas-phase geometries. The zero-point vibrational energy correction is included providing energies at 0 K. Superfine integral grid in Gaussian 16 was used in both the geometry optimizations and single-point-energy calculations. [b]Includes the empirical dispersion-energy correction at the GD3 level.[1]

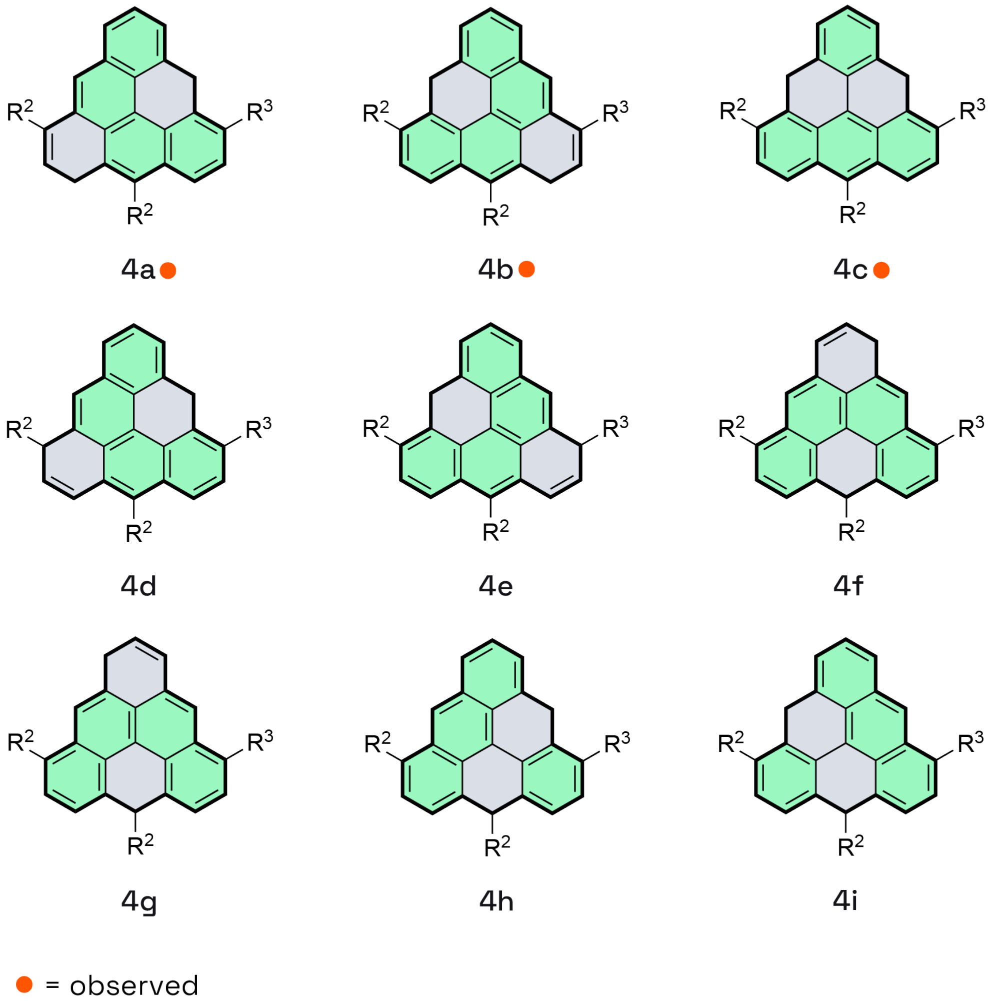

Figure S2. The nine possible isomers of dihydro-precursor **4**.

Table S2. Relative energies[a] (in kcal/mol) of the parent dihydro-precursors with different aromatic subunits calculated with different density functionals.

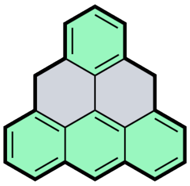

anthracene

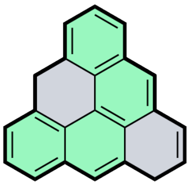

helicene

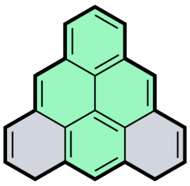

pyrene 1

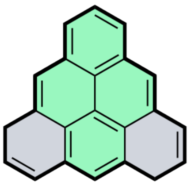

pyrene 2

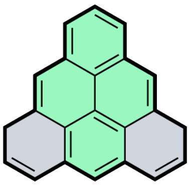

pyrene 3

Precursor	B3LYP	BMK	M06-2X
**anthracene**	1.2	1.5	1.6
**helicene**	0.0	0.0	0.0
**pyrene 1**	5.0	5.6	5.9
**pyrene 2**	6.2	6.8	7.1
**pyrene 3**	7.4	8.2	8.6

[a]The energies were obtained using cc-pVTZ basis set on the M06-2X/6-31G(d) gas-phase geometries. The zero-point vibrational energy correction is included providing energies at 0 K. Superfine integral grid in Gaussian 16 was used in both the geometry optimizations and single-point-energy calculations.

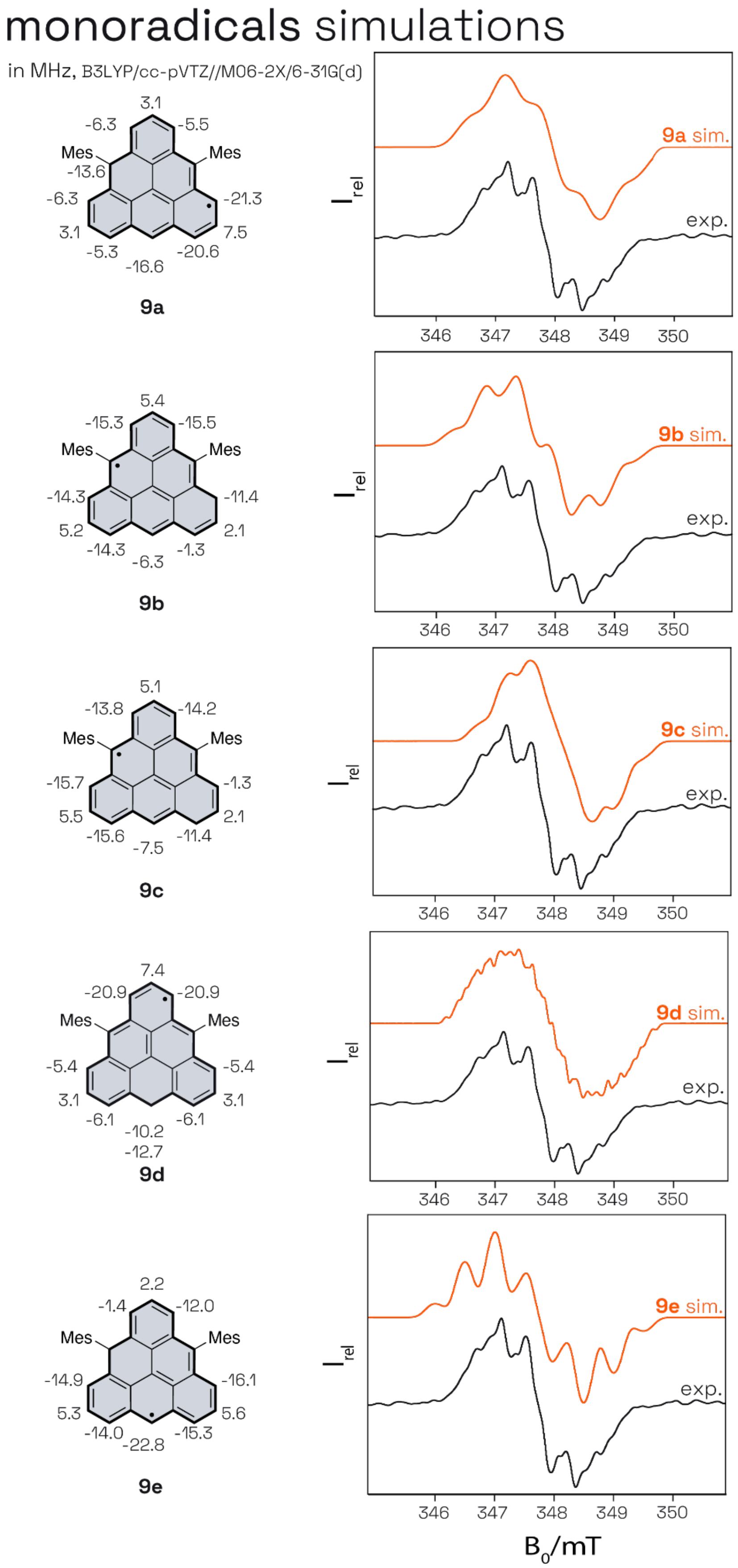

Figure S3. Simulated versus measured EPR spectra of monoradicals **9a–9e**; simulated using calculated hyperfine coupling constants shown on the left.

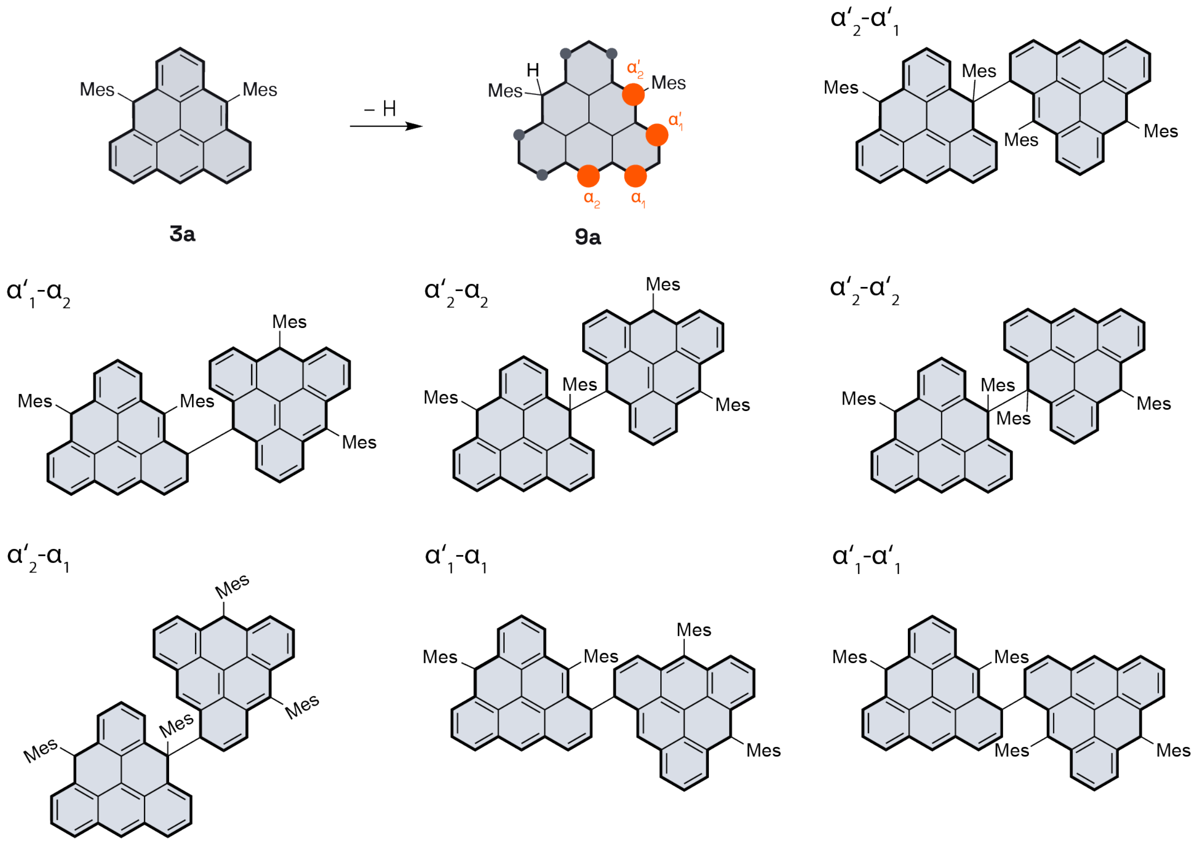

Figure S4a. Possible σ-dimer (**9a**)$_2$ regioisomers with at least one subunit linked via the sterically hindered positions α'$_1$ or α'$_2$ in **9a**.

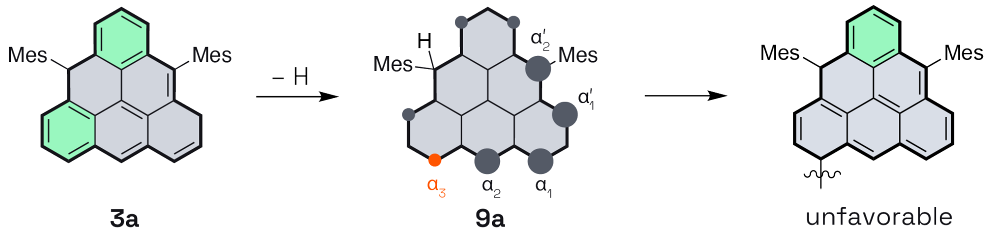

Figure S4b. Linkage via the α$_3$–α$_3$ positions yields σ-dimer (**9a**)$_2$ that is destabilized due to the loss of two Clar sextets.

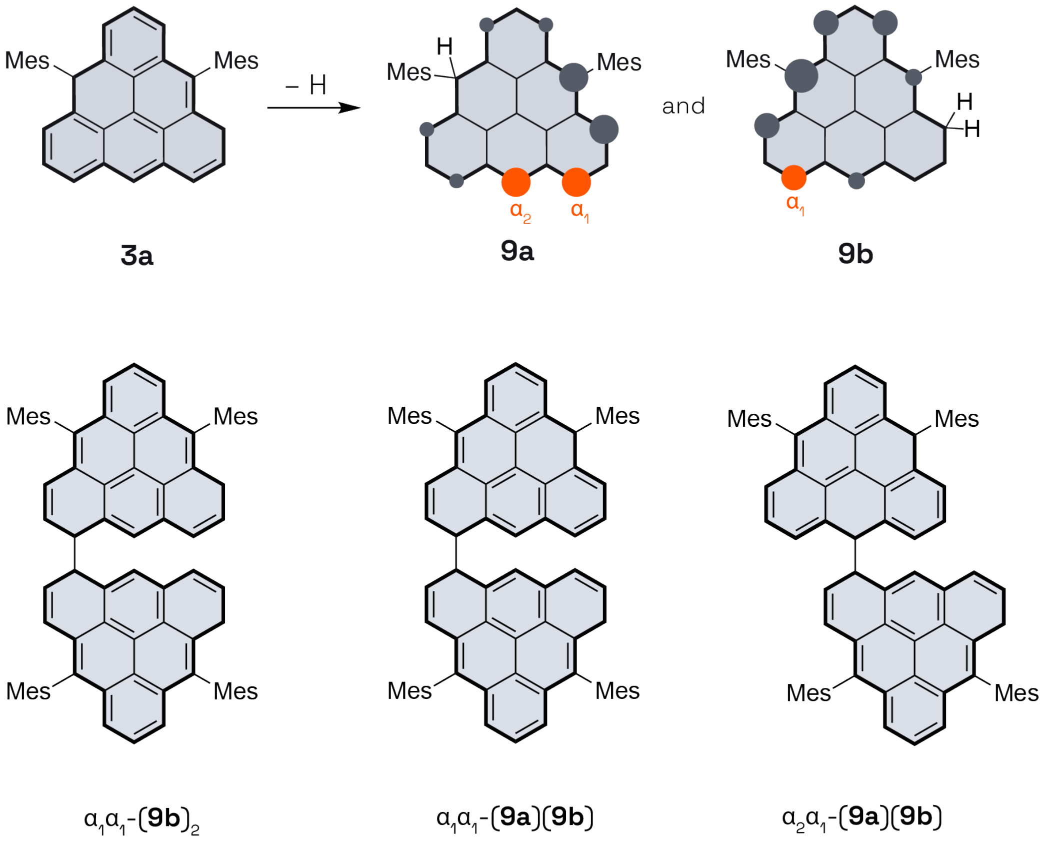

Figure S5. σ-Dimers linked through the most accessible positions in **9a** and **9b** (highlighted in orange), each containing at least one **9b** unit.

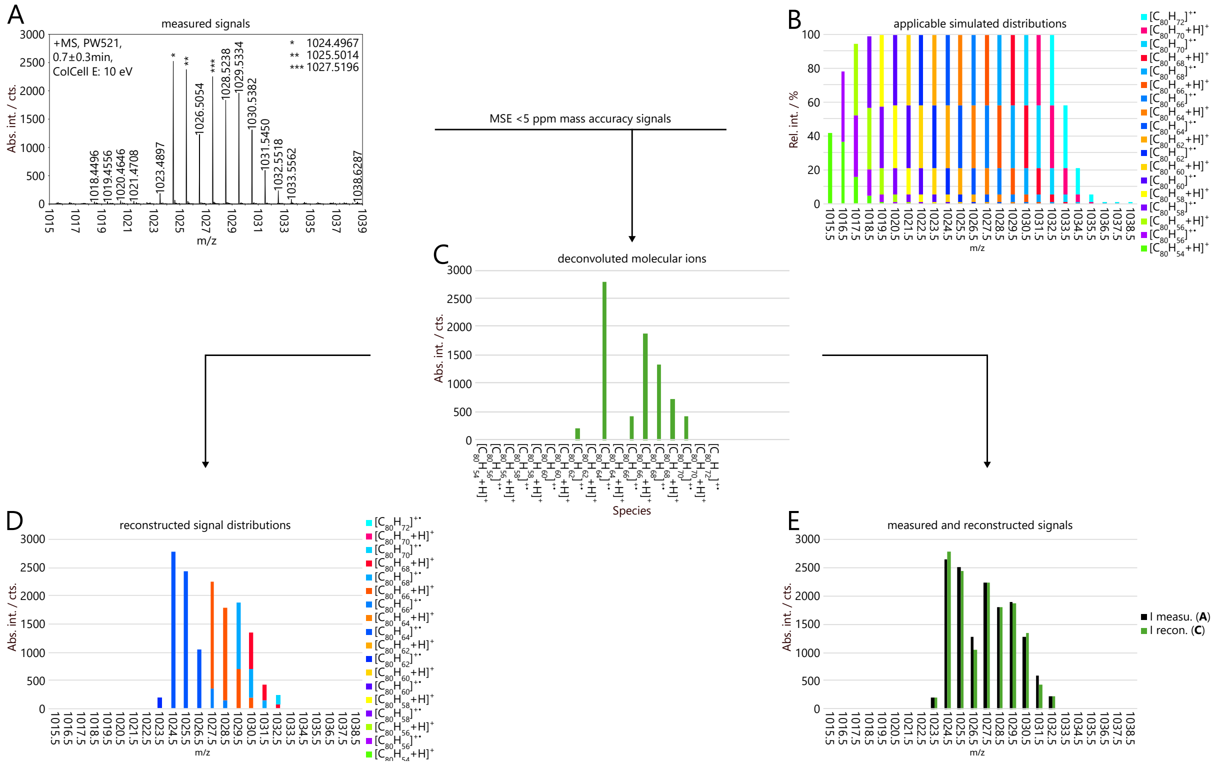

Figure S6. Workflow used for the evaluation of molecular ions and spectrum reconstruction with simulated isotopic distribution of potential reaction-intermediate ions. The intensity and *m*/*z* values from the recorded mass spectrum (A, Table S3) were read out and matched to the appropriate reaction intermediates (from $C_{80}H_{54}$ to $C_{80}H_{74}$) for the ion adducts $[M+H]^+$ and $[M]^{+\bullet}$. Therefore, the respective ion distributions (B) were simulated by enviPat[2], and the recorded signals were modeled by mean-square-error (MSE) minimization with simulated *m*/*z* values at mass accuracy <5 ppm (in relation to recorded signals). (C) MSE minimizes the mean squared differences between simulated and measured intensities of all *m*/*z* values (<5 ppm) in an iterative process by changing the molecular ions intensities until a local minimum is found. This process is necessary to deconvolute the recorded signals, which showed interference due to small mass differences ($C_{80}H_{54}$ + $n$$H_2$), and assign the right proportion of the simulated distributions of the molecular ions to the measured signals. (D) The intensities of the molecular ions were then used to reconstruct the recorded signals by adding the proportion of each molecular-ion species together with the respective simulated patterns. (E) In the end, the reconstructed spectrum was compared with the measured one. Several molecular ions were identified as shown in (C), whereof the two most prominent species were $[C_{80}H_{66} + H]^+$, the σ-dimer, and $[C_{80}H_{64}]^{+\bullet}$.

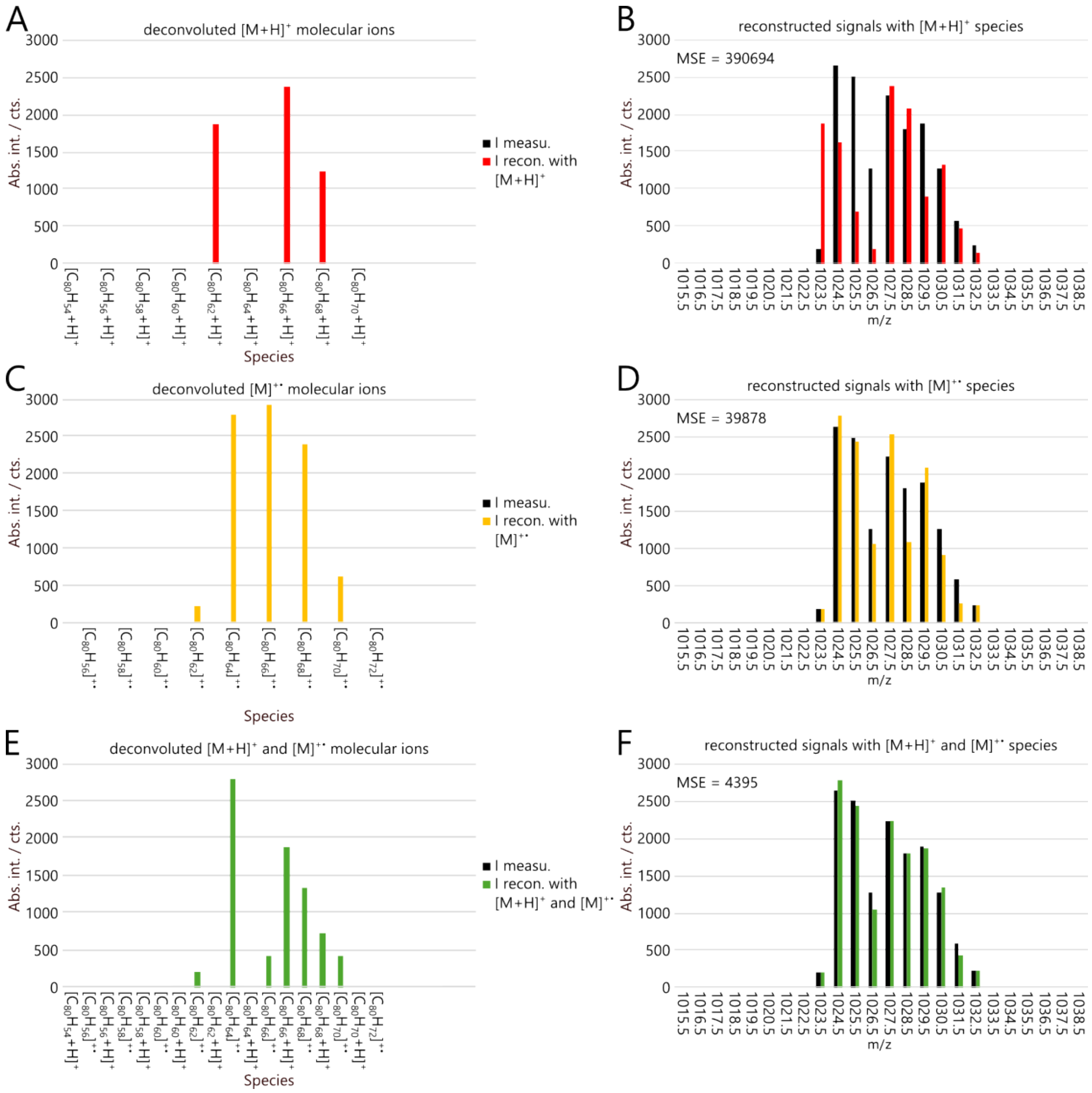

Figure S7. Deconvoluted molecular ions (left) and comparison of measured and reconstructed signals (right). According to the workflow presented in Figure S6, simulated distributions of appropriate reaction intermediates (from $C_{80}H_{54}$ to $C_{80}H_{74}$) for $[M+H]^+$ (A and B), $[M]^{+\bullet}$ (C and D), and combined $[M+H]^+$ and $[M]^{+\bullet}$ (E and F) adduct ions were used. The reconstructed mass spectrum was calculated with simulated distributions of respective molecular ions and compared with the measured spectrum. The quality of the model is characterized with the respective mean square error (MSE) of the measured and reconstructed intensities and showed best agreement when both species, $[M+H]^+$ and $[M]^{+\bullet}$, were included.

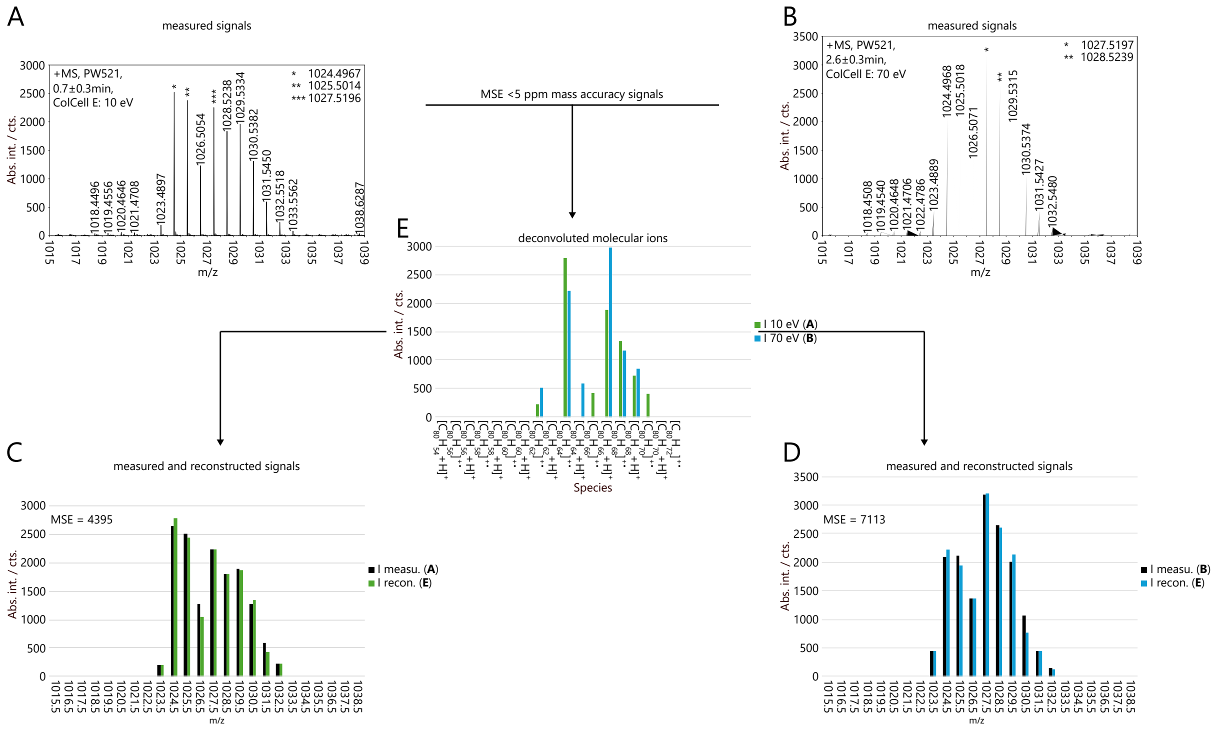

Figure S8. Measured (top) and reconstructed mass spectra (bottom) of the reaction intermediates analyzed by on-flow APPI-TimsTOF MS with collision-cell energies (ColCell E) of 10 (left) and 70 eV (right), together with the comparison of the molecular ions (middle). Signals obtained at 10 eV (A; Table S3) and 70 eV (B; Table S4) were evaluated using the workflow presented in Figure S6. Both measurements yielded good model agreement with the experimental spectra, although the 10 eV data (C) showed lower MSE values than the 70 eV data (D). Comparison of the deconvoluted molecular ions (E) showed similar signal distributions, with $[C_{80}H_{66} + H]^{+}$ and $[C_{80}H_{64}]^{+\bullet}$ appearing as the most prominent ion species in both cases. Overall, the similar proportions indicate that the observed species were indeed chemically formed in the reaction mixture rather than in-source dimers.

Table S3. Detected signals of the reaction products measured with APPI-HRMS at a collision energy (ColCell E) of 10 eV. Listed are peak number (#), mass-to-charge (*m/z*) values, mass resolutions (*R*), signal-to-noise ratios (*S/N*), absolute intensities (abs. *I*), relative intensities (rel. *I*), and full width at half maximum (FWHM) values.

**+MS, 0.8 ± 0.2 min, ColCell E: 10 eV**						
#	*m/z*	*R*	*S/N*	abs. *I*	rel. *I*	FWHM
119	1023.49	40000	35.8	189	1.8	0.0256
120	1024.497	40000	501.8	2655	25.5	0.0256
121	1025.501	40000	473.9	2507	24.1	0.0256
122	1026.505	40000	241.2	1276	12.3	0.0257
123	1027.52	40000	423.4	2240	21.5	0.0257
124	1028.524	40000	341.8	1809	17.4	0.0257
125	1029.533	40000	356.6	1887	18.1	0.0257
126	1030.538	40000	240.6	1273	12.2	0.0258
127	1031.545	40000	109.9	582	5.6	0.0258
128	1032.552	40000	44.1	233	2.2	0.0258

Table S4. Detected signals of the reaction products measured with APPI-HRMS at a collision energy (ColCell E) of 70 eV. Listed are peak number (#), mass-to-charge (*m*/*z*) values, applied resolutions (*R*), signal-to-noise ratios (*S*/*N*), absolute intensities (abs. *I*), relative intensities (rel. *I*), and full width at half maximum (FWHM).

**+MS, 2.0 ± 0.1 min, ColCell E: 70 eV**						
#	*m*/*z*	*R*	*S*/*N*	abs. *I*	rel. *I*	FWHM
166	1023.489	40000	64.2	443	14	0.0256
167	1024.497	40000	301.4	2080	65.5	0.0256
168	1025.502	40000	305	2105	66.3	0.0256
169	1026.507	40000	197.9	1366	43	0.0257
170	1027.52	40000	459.9	3174	100	0.0257
171	1028.524	40000	383.4	2646	83.4	0.0257
172	1029.532	40000	289.7	1999	63	0.0257
173	1030.537	40000	155.6	1074	33.8	0.0258
174	1031.543	40000	63	435	13.7	0.0258
175	1032.548	40000	21.6	149	4.7	0.0258

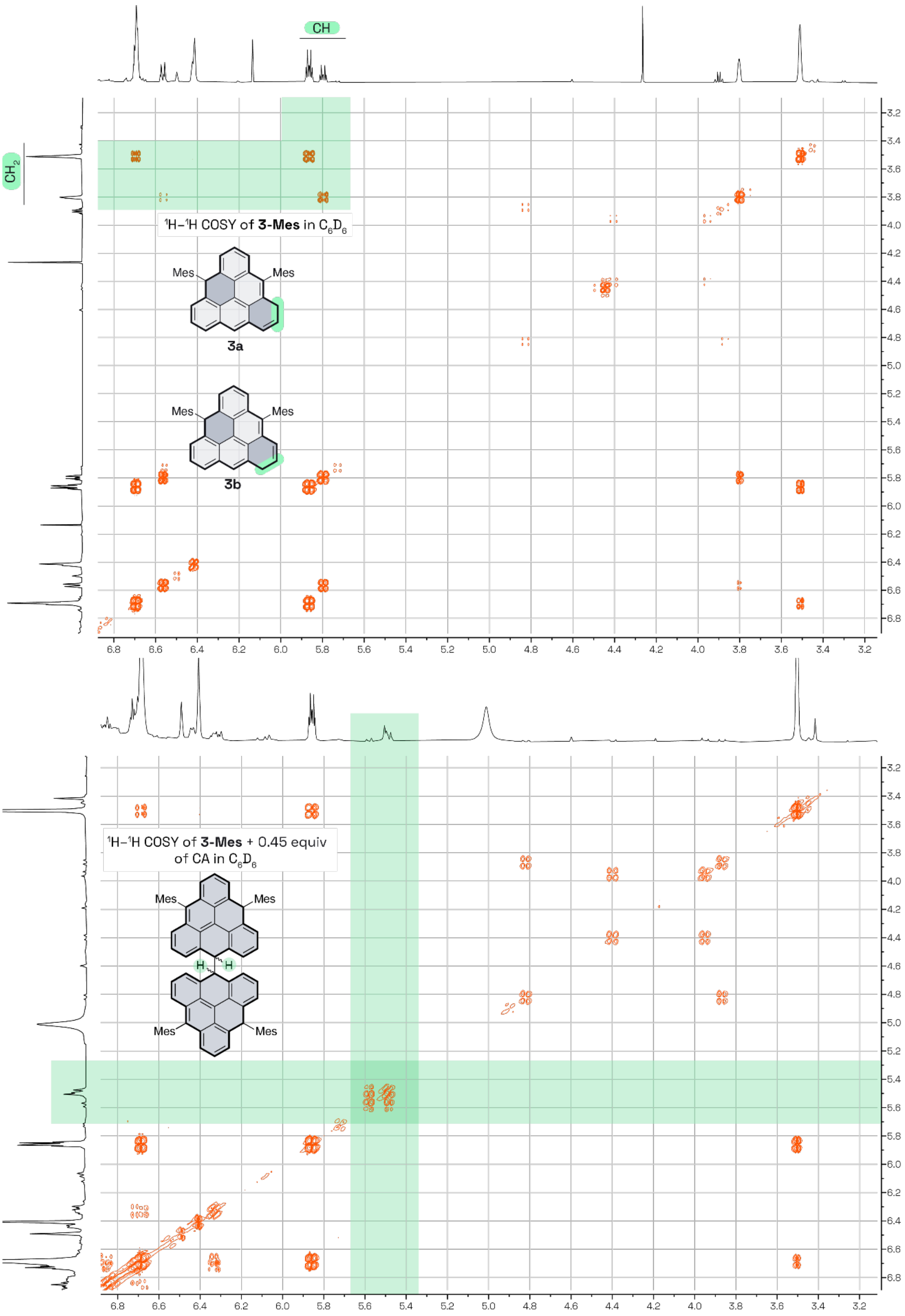

Figure S9. ^{1}H–^{1}H COSY NMR spectra of **3-Mes** and **3-Mes** + 0.45 equivalents of CA at room temperature in C_6D_6. CA = *p*-chloranil.

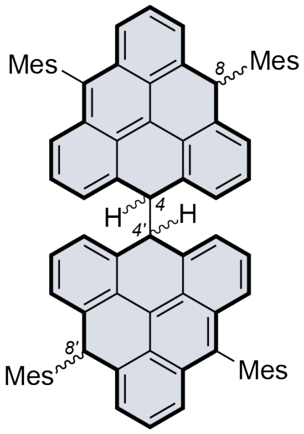

stereodescriptors shown
in the order (4,4′,8,8′)

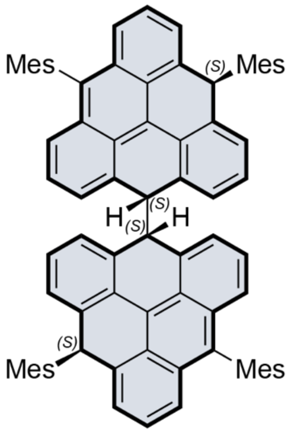

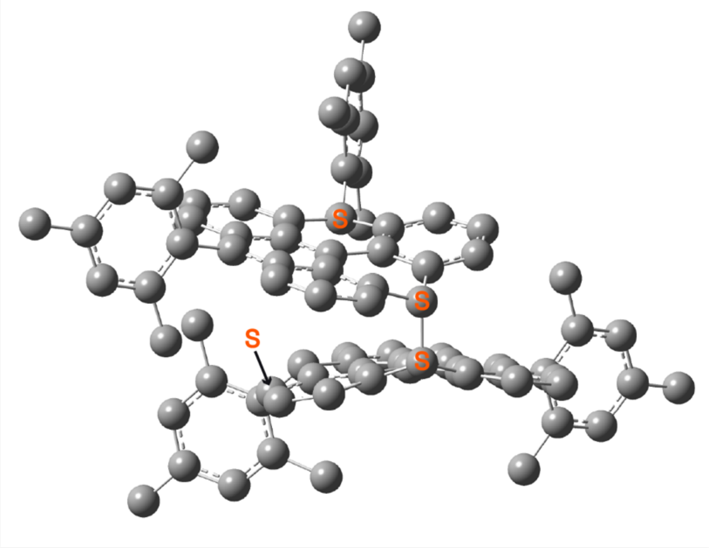

e-(S,S,S,S)-$\alpha_2\alpha_2$-(**9a**)$_2$
eclipsed

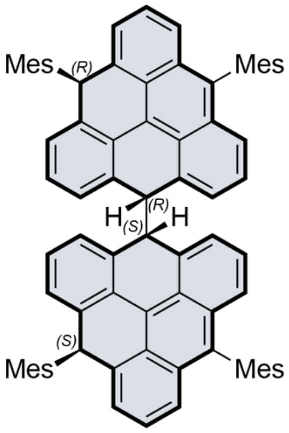

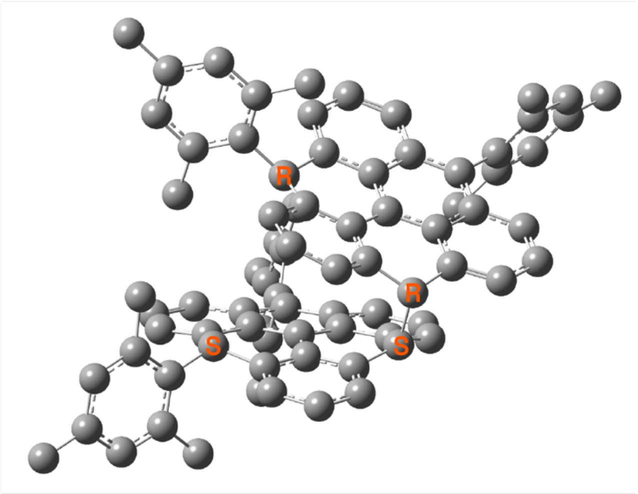

e-(R,S,R,S)-$\alpha_2\alpha_2$-(**9a**)$_2$
meso, eclipsed

Figure S10. The (*S*,*S*,*S*,*S*) and (*R*,*S*,*R*,*S*) diastereomers of $\alpha'_2\alpha'_2$-(**9a**)$_2$, and their corresponding optimized geometries. See Table S3 for relative energies calculated with different density functionals.

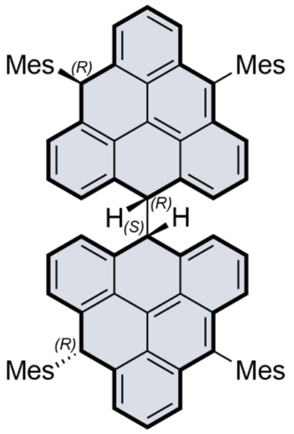

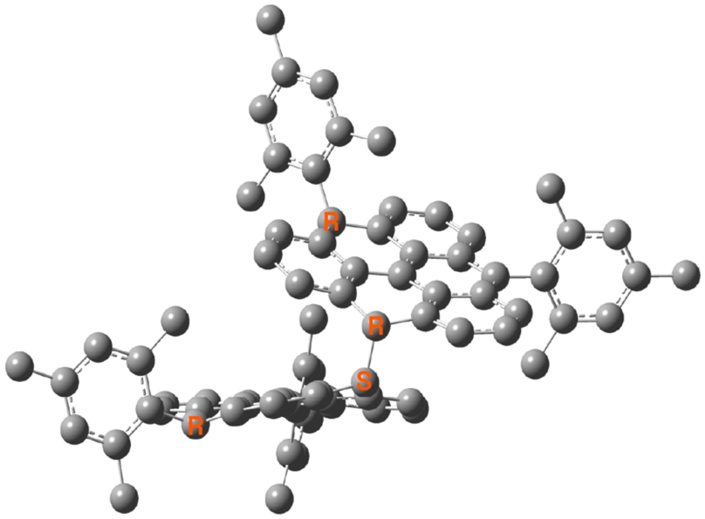

e-(R,S,R,R)-$\alpha_2\alpha_2$-(**9a**)$_2$
eclipsed

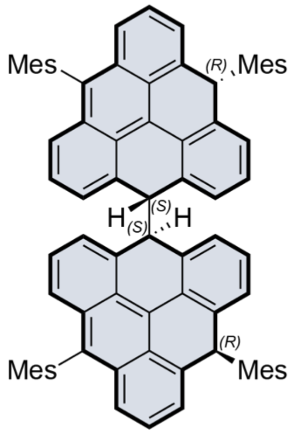

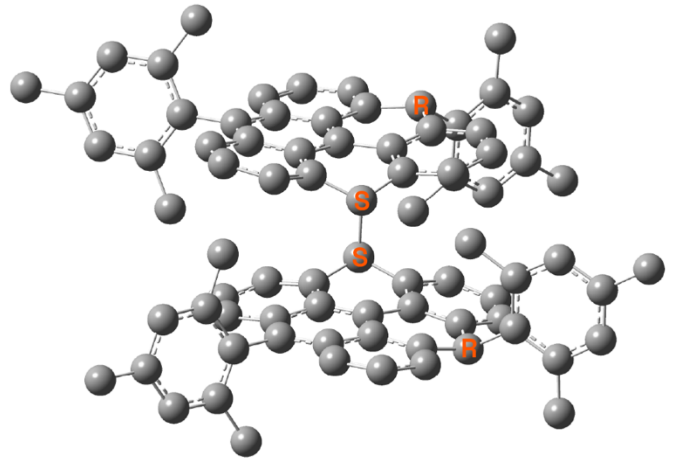

s-(S,S,R,R)-$\alpha_2\alpha_2$-(**9a**)$_2$
staggered

Figure S11. The (*R*,*S*,*R*,*R*) and (*S*,*S*,*R*,*R*) diastereomers of $\alpha'_2\alpha'_2$-(**9a**)$_2$, and their corresponding optimized geometries. See Table S3 for relative energies calculated with different density functionals.

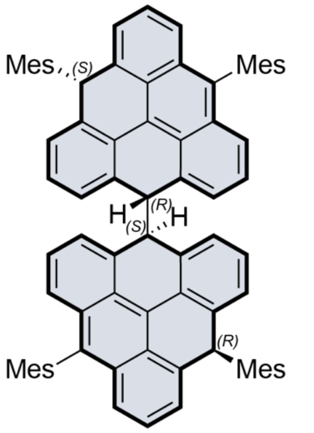

s-(R,S,S,R)-$\alpha_2\alpha_2$-(**9a**)$_2$
meso, staggered

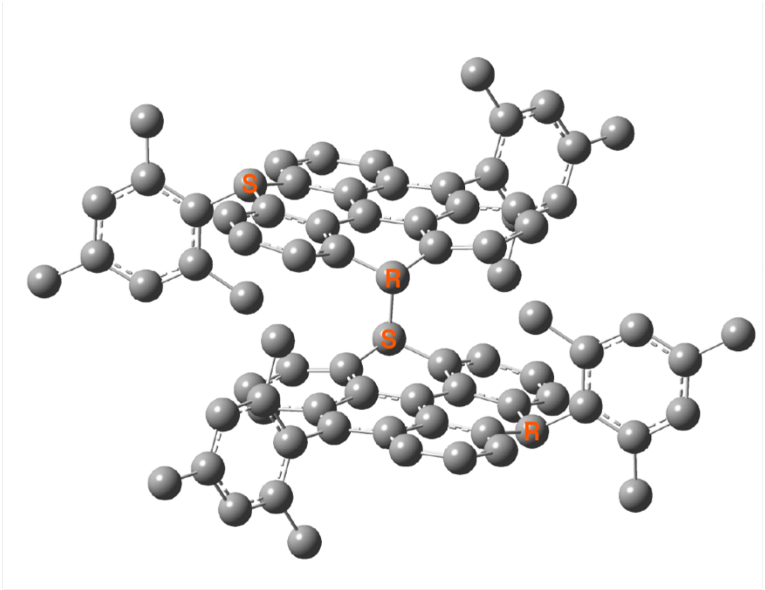

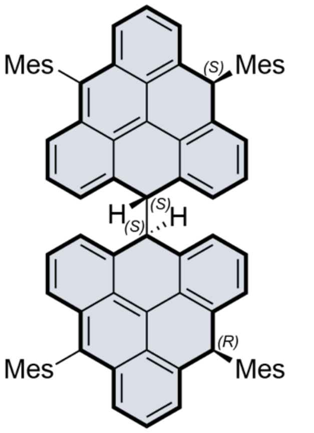

s-(S,S,S,R)-$\alpha_2\alpha_2$-(**9a**)$_2$
staggered

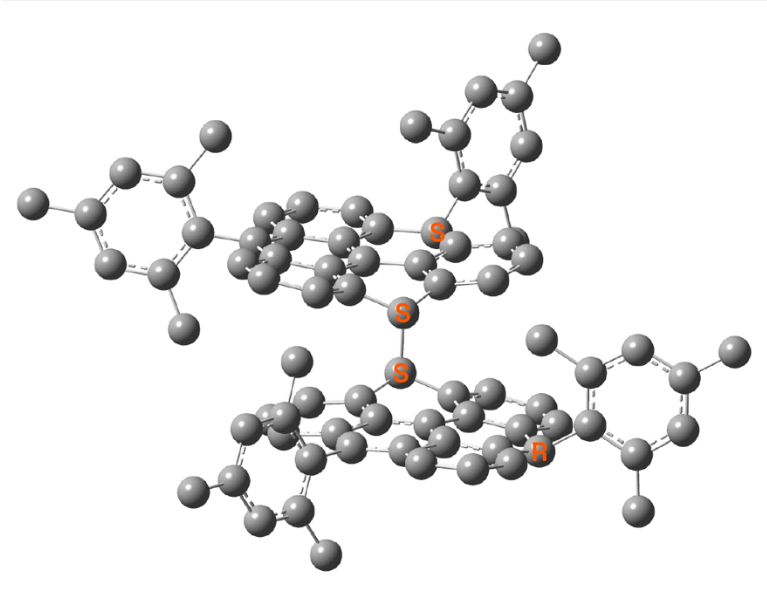

Figure S12. The (*R*,*S*,*S*,*R*) and (*S*,*S*,*S*,*R*) diastereomers of $\alpha'_2\alpha'_2$-(**9a**)$_2$, and their corresponding optimized geometries. See Table S3 for relative energies calculated with different density functionals.

Table S5. Relative energies[a] (in kcal/mol) of the six $\alpha_2\alpha_2$-(**9a**)$_2$ diastereomers (see Figures S10–S13 for structures) calculated with different density functionals.

Diastereomer of $\alpha_2\alpha_2$-(**9a**)$_2$	D3-B3LYP[b]	D3-BMK[b]	M06-2X	D3-M06-2X[b]	MN15	wB97XD
e-(*S*,*S*,*S*,*S*)	0.0	0.0	0.0	0.0	0.0	0.0
e-(*R*,*S*,*R*,*S*)	0.5	0.7	0.5	0.4	0.4	0.8
e-(*R*,*S*,*R*,*R*)	4.5	8.7	4.3	5.4	5.5	5.7
s-(*S*,*S*,*R*,*R*)	9.9	15.4	7.2	9.9	8.4	11.8
s-(*R*,*S*,*S*,*R*)	10.0	15.3	7.2	9.9	8.7	11.8
s-(*S*,*S*,*S*,*R*)	10.2	15.7	7.1	10.0	8.9	12.0

[a]The energies were obtained using cc-pVTZ basis set on the M06-2X/6-31G(d) gas-phase geometries. The zero-point vibrational energy correction is included providing energies at 0 K. Superfine integral grid in Gaussian 16 was used in both the geometry optimizations and single-point-energy calculations. [b]Includes the empirical dispersion-energy correction at the GD3 level.[1]

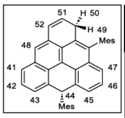

**3a** # Proton	Calculated Chemical shift / ppm	Experimental Chemical shift / ppm	Δδ (δcalc.-δexp.) Chemical shift / ppm
41	7.88	7.54	0.34
42	7.55	7.2	0.35
43	7.23	7.06	0.17
44	6.83	6.41	0.42
45	7.32	7.65	-0.33
46	7.46	7.07	0.39
47	7.38	7.33	0.05
48	7.68	7.38	0.3
49	3.64	3.51	0.13
50	3.64	3.51	0.13
51	6.34	5.86	0.48
52	7.03	6.69	0.34

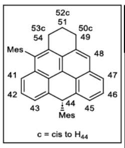

**2H-3** # Proton	Calculated Chemical shift / ppm	Experimental Chemical shift / ppm	Δδ (δcalc.-δexp.) Chemical shift / ppm
41	7.37	7.39	-0.02
42	7.43	7.1	0.33
43	7.28	7.08	0.2
44	6.86	6.5	0.36
45	7.31	7.12	0.19
46	7.62	7.28	0.34
47	7.93	7.64	0.29
48	7.96	7.56	0.4
49	3.28	2.98	0.3
50	3.37	2.98	0.39
51	2.18	1.83	0.35
52	1.96	1.83	0.13
53	2.91	2.74	0.17
54	2.72	2.74	-0.02

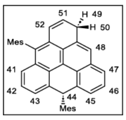

**3b** # Proton	Calculated Chemical shift / ppm	Experimental Chemical shift / ppm	Δδ (δcalc.-δexp.) Chemical shift / ppm
41	7.28	7.35	-0.07
42	7.37	7.02	0.35
43	7.25	7.02	0.23
44	6.81	6.42	0.39
45	7.25	7.09	0.16
46	7.59	7.25	0.34
47	7.88	7.56	0.32
48	8	7.44	0.56
49	4.42	3.8	0.62
50	4.43	3.8	0.63
51	6.26	5.8	0.46
52	6.63	6.56	0.07

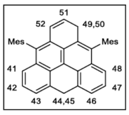

**3c** # Proton	Calculated Chemical shift / ppm	Experimental Chemical shift / ppm	Estimated δExp. Chemical shift / ppm
41	7.28	n/d	7.36
42	5.71	n/d	6.05
43	7.64	n/d	7.84
44	5.35 / 5.37	n/d	5.74 / 5.76
45	5.38 / 5.37	n/d	5.39 / 5.38
46	7.71	n/d	8.07
47	7.58	n/d	7.8
48	7.34	n/d	7.76
49	3.55	n/d	3.9
50	3.55	n/d	3.93
51	6.18	n/d	6.61
52	6.59	n/d	6.77

Figure S13. Recorded NMR signals of a mixture of **3-Mes** versus predicted chemical shifts computed by DFT of the characteristic C(sp^3)–*H* signals. Chemical shielding tensors are referenced to the isotropic chemical shielding value computed for TMS. The chemical shielding tensors were calculated at GIAO/B3LYP/cc-pVTZ (PCM, solvent: benzene) level of theory on the gas-phase M06-2X/6-31G(d) geometries. Superfine integral grid was used in both geometry optimizations and shielding-tensor calculations. To estimate the expected chemical shifts of **3c**, the average Δ*δ* derived from all signals of **3a**, 2*H*-**3**, and **3b** signals was added to (or subtracted from) the calculated value for **3c**.

Table S6. Relative energies[a] (in kcal/mol) of monoradicals **9a**–**9e** (see Figure S3 for structures) calculated with different density functionals.

Monoradical	B3LYP	BMK	M06-2X
**9a**	5.7	4.5	4.1
**9b**	1.7	1.3	1.2
**9c**	1.9	2.0	2.1
**9d**	0.0	0.0	0.0
**9e**	2.2	1.9	1.8

[a]The energies were obtained using cc-pVTZ basis set on the M06-2X/6-31G(d) gas-phase geometries. The zero-point vibrational energy correction is included providing energies at 0 K. Superfine integral grid in Gaussian 16 was used in both the geometry optimizations and single-point-energy calculations.

2. General Information

Chemicals

Anhydrous solvents and chemical reagents were purchased from commercial sources and used without further purification, unless stated otherwise. THF was distilled prior to use.

NMR spectroscopy

The NMR experiments were performed on NMR spectrometers operating at 400, 500, or 600 MHz proton frequencies. Standard pulse sequences were used. Chemical shifts (δ) are reported in parts per million (ppm) relative to the solvent residual peak (^{1}H and ^{13}C NMR, respectively): $CDCl_3$ (δ = 7.26 and 77.16 ppm), CD_2Cl_2 (δ = 5.32 and 53.84 ppm), C_6D_6 (δ = 7.16 ppm and 128.06), toluene-d_8 (δ = 7.09 and 128.87 ppm), THF-d_8 (δ = 3.58 and 67.21 ppm).[3]

IR spectroscopy

IR spectra were recorded on a Perkin Elmer Spectrum Two ATR-FTIR and the compounds were measured as neat samples, unless stated otherwise.

EPR spectroscopy

The X-band continuous wave EPR spectra were recorded on a Bruker EMXnano benchtop EPR spectrometer. The modulation frequency was set to 100 kHz and the modulation amplitude to 0.01 mT unless stated otherwise. The microwave power was adjusted for every sample to avoid saturation effects.

Simulation of continuous wave (cw) EPR spectra

The different continuous wave (cw) EPR spectra were simulated with Matlab using the EasySpin[4] package. Specifically, the EasySpin 'garlic' routine was used to simulate the spin-1/2 systems. The isotropic g value was set to 2.0027 to match the experimental cw EPR spectrum.

For the hyperfine structure, isotropic hyperfine coupling constants obtained from DFT calculations were used directly. To account for unresolved hyperfine interactions, line widths in the range of 0.15 to 0.3 mT were applied.

Preparative gel-permeation chromatography (GPC)

GPC was carried out on a Shimadzu recycling GPC system equipped with an LC-20AR prominence liquid chromatograph pump, an SPDM40 photodiode array detector, a DGU-403 degassing, and a CBM-40 system controller using two ReproGel 500 GPC columns (5 µm, 20 × 600 mm) with chloroform as the eluent passing through at a rate of 3.5 mL per minute.

Mass spectrometric analysis

High-resolution mass spectra (HRMS) were measured as HR-EI-MS, HR-ESI-MS, or HR-APCI-MS. For the APPI-HRMS analysis, methanol and formic acid were purchased from Biosolve (ULC/MS grade, Dieuze, France), dichloromethane from VWR Chemicals (HiPerSolv Chromanorm, Dietikon, Switzerland), and chloroform from Honeywell (Chromasolv Plus, Seelze, Germany). *Agilent* ESI-L low-concentration tuning mix solution (*Agilent*, USA) was used for instrument calibration. Samples were analyzed with a *Vanquish™ Horizon UHPLC* System (*Thermo Fisher Scientific*, Waltham, USA) connected to a *Vanquish* eλ detector and an atmospheric-pressure photoionization (LC-APPI II source from Bruker Daltonics Inc, Billerica, USA) source coupled to a *timsTOF Pro* high-resolution mass spectrometer (*Bruker Daltonics*, Bremen, Germany). The samples were dissolved in MeOH/$CHCl_3$ (3:2, *v:v*) at a concentration of ca. 100 µg L^{-1} and analyzed via continuous flow injection at 303 µL min^{-1} using a tee (3 µL min^{-1} sample solution, 300 µL min^{-1} mobile phase consisting of MeOH + 0.1% HCOOH). The mass spectrometer was operated in the positive ionization mode at 1'000 V capillary voltage and 500 V endplate offset with a APPI heater temperature of 470 °C, N_2 nebulizer pressure of 2.5 bar, and a dry gas flow of 1.5 L min^{-1} at 220 °C. Mass spectra were acquired in a mass range from *m*/*z* 100 and 1'500 at circa 20'000 resolution (*m*/*z* 622) and 1.0 Hz scan rate. The in-source collision-induced dissociation (isCID) energy was kept at 0 eV, the radio frequency (RF) of funnel 2 at 300 V peak-to-peak (Vpp), quadrupole ion energy of 15 eV. The collision cell energy was set to 10 or 70 eV. The mass analyzer was calibrated between *m*/*z* 118 and 2'721 using an *Agilent* ESI-L low concentration tuning mix (*Agilent*, USA) at a resolution of 20'000 giving a mass accuracy below 2 ppm. DataAnalysis 5.3 was used for the data processing. A mass tolerance of ±0.01 was selected for calculating extracted ion chromatograms.

Quantum chemical calculations

Calculations were performed with Gaussian 16 (revision C.02) suite of electronic structure programs.[5] Geometries of molecules in their potential-energy-surface minima were optimized at M06-2X/6-31G(d) level of theory. Subsequent frequency calculation confirmed that the character of the found stationary point was a minimum and provided zero-point vibrational energy corrections (ZPVEs). In two cases of molecules with the large mesityl groups, one of the twelve methyl groups in the calculated $\alpha_1\alpha_2$-(**9a**)$_2$ could not be fully relaxed and an imaginary frequency that corresponded to unhindered Me rotation was found. The errors in the final energies for these cases are <0.3 kcal/mol. Superfine integration grid was used in all calculations, geometry optimizations, single-point-energy calculations, or NMR chemical shielding tensors calculations. Various density functionals were used in single-point-energy calculations to assess the effect of the functional on the relative energies. The cc-pVTZ basis set was employed in all single-point-energy calculations. The relative energies include unscaled ZPVEs, i.e., the final energies are energies at 0 K. The NMR chemical shielding tensors were calculated with respect to the tetramethylsilane reference using GIAO method at the B3LYP/cc-pVTZ level of theory including implicit solvation modelled as PCM with benzene as solvent. The EPR hyperfine couplings to simulate the experimental EPR spectra were calculated at the UB3LYP/cc-pVTZ level of theory.

3. Experimental Procedures

9-(2,6-Bis(methoxymethyl)phenyl)anthracene (12)

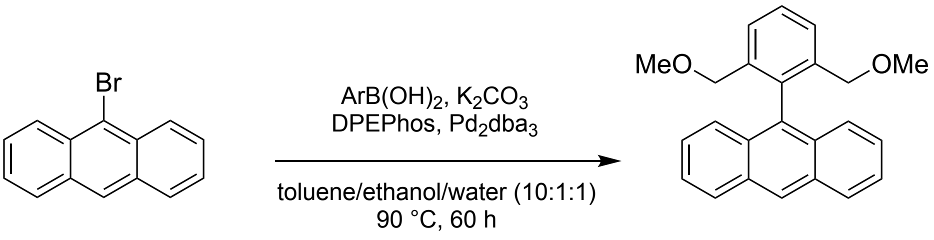

12, 63%

A mixture of 9-bromoanthracene (5.00 g, 19.4 mmol), (2,6-bis(methoxymethyl)phenyl)-boronic acid ($ArB(OH)_2$; 6.13 g, 29.2 mmol), DPEPhos (1.05 g, 1.95 mmol), Pd_2dba_3 (0.89 g, 0.97 mmol), and K_2CO_3 (16.1 g, 117 mmol) in toluene (40 mL), ethanol (4 mL), and water (4 mL) was deoxygenated in a Schlenk flask by freeze-pump-thaw technique in three cycles and then stirred at 90 °C for 60 h. Upon cooling to room temperature, water (60 mL) was added and the mixture was extracted with CH_2Cl_2 (3 × 50 mL). The combined organic phases were dried over $MgSO_4$ and the solvent was evaporated. The residue was purified by column chromatography (SiO_2, cyclohexane/ethyl acetate, 15:1) to afford the product (4.22 g, 63%) as a pale yellow solid (mp 109.3–110.1 °C).

^{1}H NMR (400 MHz, $CDCl_3$, ppm): δ 8.54 (s, 1H), 8.07 (d, J = 8.5 Hz, 2H), 7.70 (d, J = 8.4 Hz, 1H), 7.70 (d, J = 6.5 Hz, 1H), 7.64 (dd, J = 8.8, 6.4 Hz, 1H), 7.50–7.44 (m, 4H), 7.35 (ddd, J = 8.8, 6.4, 1.3 Hz, 2H), 3.83 (s, 4H), 2.99 (s, 6H).

^{13}C NMR (101 MHz, $CDCl_3$, ppm): δ 138.3, 134.7, 132.3, 131.5, 130.1, 128.7, 128.5, 127.1, 126.3, 126.02, 125.96, 125.5, 71.9, 58.3.

IR: 2819, 1650, 1590, 1447, 1438, 1342, 1193, 1100, 980, 969, 891, 760, 739, 693, 558, 486, 477 cm^{-1}.

HRMS (ESI) m/z: $[M + Na]^+$ Calcd for $C_{24}H_{22}O_2$ 365.15120; Found 365.15081.

9-(2,6-Bis(bromomethyl)phenyl)anthracene (13)

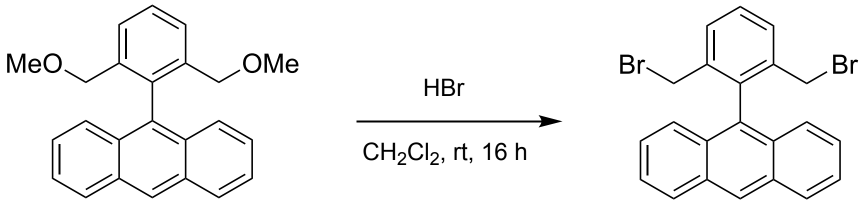

12 **13**, 79%

Compound **12** (8.12 g, 23.7 mmol) was placed into a flame-dried Schlenk flask and the atmosphere was exchanged three times with nitrogen before dry CH_2Cl_2 (40 mL) was added. To this solution, HBr (64.6 mL, 356 mmol, 33% in acetic acid) was added and the reaction mixture was stirred at room temperature for 16 h. The mixture was poured onto water and extracted with CH_2Cl_2 (3 × 80 mL). The combined organic phases were dried over $MgSO_4$ and the solvent was evaporated. The residue was purified by column chromatography (SiO_2, cyclohexane/ethyl acetate, 40:1) to afford the product (8.26 g, 79%) as a white solid (mp 131.9–132.7 °C).

^{1}H NMR (400 MHz, $CDCl_3$, ppm): δ 8.59 (s, 1H), 8.09 (d, J = 8.5, 2H), 7.72 (d, J = 8.0 Hz, 1H), 7.61 (d, J = 7.4, 1H), 7.60 (dd, J = 8.4, 7.0 Hz, 1H), 7.52–7.48 (m, 2H), 7.42–7.36 (m, 4H), 3.95 (s, 4H).

^{13}C NMR (101 MHz, $CDCl_3$, ppm): δ 138.2, 137.8, 131.5, 131.2, 130.52, 130.45, 129.5, 128.7, 128.1, 126.4, 126.2, 125.7, 31.4.

IR: 3045, 1209, 890, 791, 734, 637, 612, 590, 555 cm^{-1}.

HRMS (EI) m/z: $[M]^+$ Calcd for $C_{22}H_{16}Br_2$ 437.96133; Found 437.96198.

(2-(Anthracen-9-yl)-1,3-phenylene)dimethanol (14)

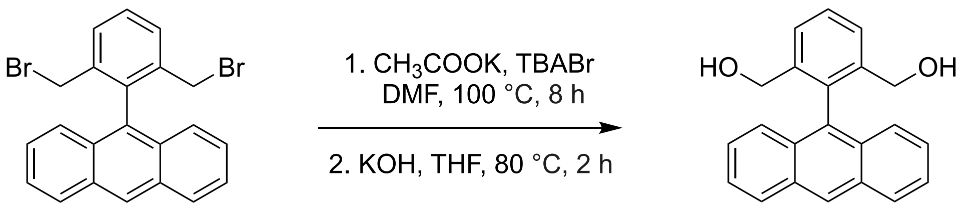

13 **14**, 99%

A solution of compound **13** (8.37 g, 19.0 mmol), potassium acetate (18.7 g, 190 mmol), and tetrabutylammonium bromide (TBABr; 6.13 g, 19.0 mmol) in dimethylformamide (60 mL) was stirred at 100 °C for 8 h under a nitrogen atmosphere. Upon cooling, the reaction mixture was poured over ice and extracted with CH_2Cl_2 (3 × 60 mL). The combined organic phases were extracted twice with an excess of water and aq. LiCl (5%) and then dried over $MgSO_4$. The crude pale yellow oil was dissolved in THF (50 mL), added to a solution of KOH (10.7 g, 4.19 mol) in ethanol (40 mL) and water (20 mL), and the mixture was stirred at 80 °C for 2 h. Then, sat. aq. NH_4Cl (100 mL) was added and the mixture was extracted with CH_2Cl_2 (3 × 60 mL). The combined organic phases were dried over $MgSO_4$ and the solvent was evaporated. The residue was purified by column chromatography (SiO_2, cyclohexane/ethyl acetate, 3:1) to afford the product (5.99 g, 99% over two steps) as a pale yellow solid (mp 200.1–201.1 °C).

^{1}H NMR (400 MHz, $CDCl_3$, ppm): δ 8.55 (s, 1H), 8.08 (d, J = 8.5 Hz, 2H), 7.71 (d, J = 8.5 Hz, 1H), 7.71 (d, J = 6.5 Hz, 1H), 7.65 (dd, J = 8.8, 6.4 Hz, 1H), 7.50–7.46 (m, 2H), 7.41–7.34 (m, 4H), 4.06 (s, 4 H).

^{13}C NMR (101 MHz, $CDCl_3$, ppm): δ 162.7, 140.5, 134.5, 132.0, 131.5, 130.1, 128.9, 127.4, 126.9, 126.5, 125.6, 125.5, 63.0.

IR: 3276, 2920, 1441, 1060, 1042, 885, 737, 613, 555 cm^{-1}.

HRMS (ESI) *m/z*: [*M* + Na]$^+$ Calcd for $C_{22}H_{18}O_2$ 337.11990; Found 337.11983.

2-(Anthracen-9-yl)isophthalaldehyde (15)

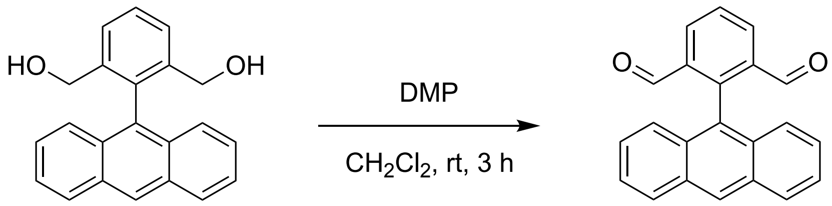

14 **15**, 76%

Compound **14** (5.70 g, 18.1 mmol) was placed in a dry flask and the atmosphere was exchanged three times with nitrogen before dry CH_2Cl_2 (200 mL) was added. To this solution, Dess–Martin periodinane (DMP; 16.9 g, 39.9 mmol) was added in one portion as a solid and the reaction mixture was stirred at room temperature for 3 h. The reaction mixture was quenched by slow addition of sat. aq. $NaHCO_3$ (10 mL) and the mixture was extracted with CH_2Cl_2 (3 × 100 mL) and aq. NaOH (1 M). The combined organic phases were dried over $MgSO_4$ and the solvent was evaporated. The residue was purified by column chromatography (SiO_2, CH_2Cl_2/cyclohexane, 3:1) to afford the product (4.28 g, 76%) as a yellow solid (mp 180.1 –181.3 °C).

^{1}H NMR (400 MHz, $CDCl_3$, ppm): δ 9.22 (s, 2H), 8.67 (s, 1H), 8.46 (d, J = 7.8 Hz, 2H), 8.13 (d, J = 8.5 Hz, 2H), 7.87 (t, J = 7.8 Hz, 1H), 7.52 (ddd, J = 8.3, 6.5, 1.2 Hz, 2H), 7.42 (ddd, J = 8.9, 6.5, 1.3 Hz, 2H), 7.32 (d, J = 8.8 Hz, 2H).

^{13}C NMR (126 MHz, $CDCl_3$, ppm): δ 190.8, 145.7, 136.4, 133.0, 132.0, 131.0, 129.4, 129.1, 127.5, 125.9, 125.7. Two signals were not resolved due to signal overlap.

IR: 3359, 2922, 2855, 1679, 1237, 741, 624, 609, 554 cm^{-1}.

HRMS (ESI) m/z: $[M + Na]^+$ Calcd for $C_{22}H_{14}O_2$ 333.08860; Found 333.08860.

(2-(Anthracen-9-yl)-1,3-phenylene)bis(mesitylmethanol) (16)

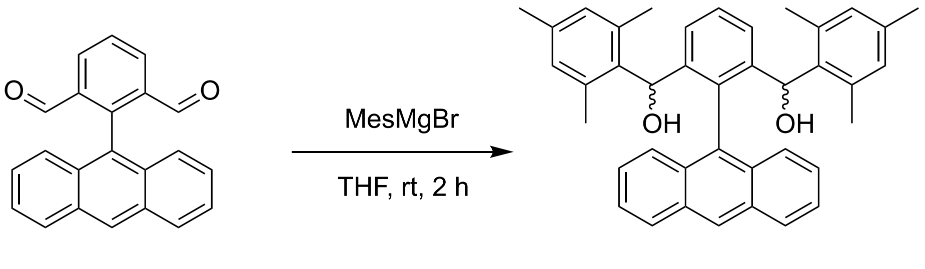

15 **16**, 99%

To a solution of **15** (3.50 g, 11.3 mmol) in dry THF (150 mL), 2-mesitylmagnesium bromide (MesMgBr; 45 mL, 45 mmol, 1.0 M in THF) was added dropwise and the reaction mixture was stirred for additional 2 h. The mixture was quenched by the addition of water (25 mL) and then extracted with CH_2Cl_2 (3 × 100 mL). The combined organic phases were dried over $MgSO_4$ and the solvent was evaporated. The oily residue was heated at 35 °C and kept under high vacuum for 24 h to remove all residual mesitylene. The light-yellow crude mixture was washed with cold hexane to afford the product (6.17 g, 99%) as a white solid (mp 274.9–275.8 °C).

^{1}H NMR (400 MHz, THF-d_8, ppm): δ 8.19 (d, J = 7.9 Hz, 2H), 8.12 (s, 1H), 7.69 (d, J = 8.5 Hz, 2H), 7.59 (t, J = 7.9 Hz, 1H), 7.18–7.12 (m, 4H), 6.91 (dd, J = 8.7, 6.4 Hz, 2H), 5.82 (s, 2H), 5.49 (d, J = 3.4 Hz, 2H), 1.75 (s, 6H), 1.22 (s, 12H).

^{13}C NMR (126 MHz, THF-d_8, ppm): δ 145.7, 137.6, 136.8, 135.3, 135.1, 134.3, 132.8, 130.6, 130.0, 128.9, 127.3, 127.3, 127.1, 126.9, 125.5, 125.0, 70.0, 20.7, 20.4.

IR: 3251, 1195, 1040, 1013, 845, 732, 638 cm^{-1}.

HRMS (ESI) m/z: $[M + Na]^+$ Calcd for $C_{40}H_{38}O_2$ 573.27640; Found 573.27637.

4,8-Dimesityl-4,8-dihydrodibenzo[*cd*,*mn*]pyrene (3-Mes)

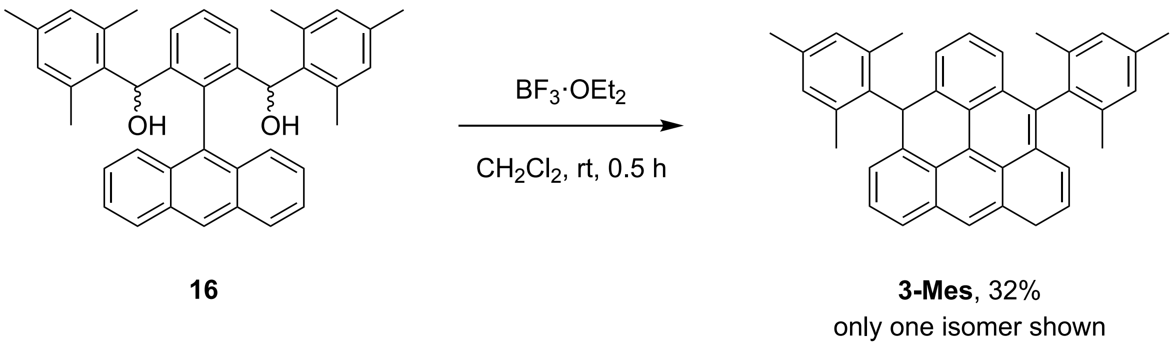

Compound **16** (0.40 g, 0.73 mmol) was suspended in dry CH_2Cl_2 (50 mL) and deoxygenated by freeze-pump-thaw technique in three cycles before $BF_3{\cdot}Et_2O$ (0.39 mL, 1.5 mmol) was added slowly. The reaction mixture was stirred at room temperature for 0.5 h, then quenched by the addition of dry and deoxygenated methanol (2 mL). The solvents were evaporated using a Schlenk line and the residue was purified by column chromatography (SiO_2, pentane/CH_2Cl_2, 5:1) under inert conditions using deoxygenated silica gel and solvent. A yellow compound was collected into a Schlenk flask, and the solvent was evaporated using a Schlenk line. The product (121 mg, 32%) was obtained as an orange solid (mp > 171 °C, decomposition) and as a mixture of structural dihydro-isomers **3a**–**3c** and compound 2H-**3**.

3a:

^{1}H NMR (600 MHz, C_6D_6, ppm): δ 7.54 (dt, J = 7.9, 1.4 Hz, 1H), 7.38 (s, 1H), 7.33 (dt, J = 7.8, 1.6 Hz, 1H), 7.20 (t, J = 7.6 Hz, 1H), 6.96 (d, J = 2.0 Hz, 1H), 6.69 (two overlaid signals, ddt, J = 6.2, 4.4, 2.2 Hz, 2H), 6.41 (s, 1H), 5.86 (dt, J = 9.7, 4.0 Hz, 1H), 3.51 (t, J = 3.2 Hz, 2H), 2.34 (s, 3H), 2.30 (s, 3H), 2.20 (s, 3H), 1.90 (s, 3H), 1.82 (s, 3H), 1.67 (s, 3H).

^{13}C NMR (151 MHz, C_6D_6, ppm): δ 141.8, 138.8, 138.6, 138.4, 137.1, 137.0, 136.3, 136.22, 136.19, 135.9, 135.5, 135.4, 133.6, 132.3, 131.3, 131.2, 129.54, 129.49, 129.46, 128.6, 128.4, 128.2, 128.0, 127.6, 127.4, 127.3, 125.8, 125.5, 125.2, 124.6, 124.5, 124.3, 122.8, 44.3, 31.9, 21.3, 21.01, 20.95, 19.72, 19.68.

3b:

^{1}H NMR (600 MHz, C_6D_6, ppm): δ 7.56 (d, J = 1.3 Hz, 1H), 7.44 (s, 1H), 7.35 (ddd, J = 7.1, 2.7, 1.3 Hz, 1H), 7.25 (t, J = 7.6 Hz, 1H), 6.56 (dq, J = 10.1, 2.1 Hz, 1H), 6.44–6.42 (m, 1H), 5.80 (dt, J = 10.1, 4.0 Hz, 1H), 3.80 (dq, J = 4.0, 1.9 Hz, 2H), 2.34 (s, 3H), 2.31 (s, 3H), 2.20 (s, 3H), 2.08 (s, 3H), 2.01 (s, 3H), 1.67 (s, 3H).

^{13}C NMR (151 MHz, C_6D_6, ppm): δ 141.9, 138.8, 138.5, 138.4, 137.6, 137.44, 137.14, 137.07, 136.3, 135.4, 133.3, 132.6, 132.3, 132.1, 131.9, 128.92, 128.90, 128.63, 128.57, 127.7, 127.2, 127.1, 126.4, 125.8, 125.7, 125.3, 125.01, 124.99, 124.1, 123.8, 44.3, 32.2, 21.3, 21.04, 21.01, 20.9, 20.3.

2*H*-**3**:

^{1}H NMR (600 MHz, C_6D_6, ppm): δ 7.64 (dt, J = 8.0, 1.3 Hz, 1H), 7.56 (s, 1H), 7.39 (t, J = 1.5 Hz, 1H), 7.28 (t, J = 7.6 Hz, 1H), 7.12 (d, J = 2.2 Hz, 1H), 7.10 (d, J = 2.2 Hz, 1H), 7.08 (d, J = 2.5 Hz, 2H), 7.03 (s, 1H), 7.02 (s, 1H), 6.69 (s, 1H), 3.03–2.93 (m, 2H), 2.74 (dd, J = 7.2, 5.2 Hz, 2H), 2.34 (s, 3H), 2.33 (s, 3H), 2.20 (s, 3H), 2.00 (s, 3H), 1.94 (s, 3H), 1.83 (s, 2H).

^{13}C NMR (151 MHz, C_6D_6, ppm): δ 141.9, 138.8, 138.6, 138.4, 137.2, 136.9, 136.8, 136.7, 136.6, 136.3, 135.4, 134.9, 132.2, 132.07, 132.05, 131.1, 129.1, 129.0, 128.6, 127.6, 127.4, 127.2, 126.5, 125.9, 125.6, 125.1, 124.4, 124.0, 123.2, 44.5, 32.4, 29.8, 23.5, 21.3, 21.01, 20.97, 20.2, 20.1.

3c:

Due to major overlap with other **3-Mes** isomers in the aromatic region, only the visible, clearly identifiable signals are reported below.

^{1}H NMR (600 MHz, C_6D_6, ppm): δ 5.73 (dt, J = 10.1, 4.0 Hz, 1H), 4.60 (s, 1H), 3.45 (dd, J = 4.1, 2.4 Hz, 2H); not complete.

The 3-Mes mixture:

IR: 2914, 2857, 1611, 1441, 850, 762 cm^{-1}.

HRMS (APCI) *m*/*z*: $[M + H]^+$ Calcd for $C_{40}H_{34}$ 515.27306; Found 515.27306.

4,9,13,18-Tetramesitylanthra[2,1,9,8-*wxyza*]benzo[*def*]naphtho[3,2,1,8,7-*qrstu*]pyran-threne (1-Mes)

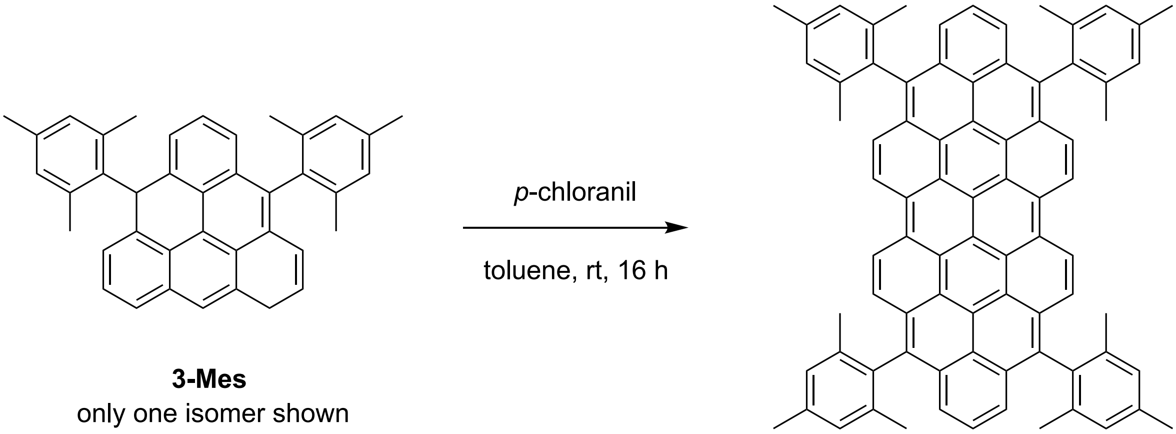

3-Mes
only one isomer shown

1-Mes, 33%

A mixture of dihydro-precursors of dimesityltriangulene (**3-Mes**; 40 mg, 0.081 mmol) was placed in a flame-dried Schlenk tube and the atmosphere was exchanged three times with nitrogen. To another flame-dried Schlenk tube, *p*-chloranil (96 mg, 0.39 mmol) was dissolved in dry toluene (4 mL), and the solution was deoxygenated by freeze-pump-thaw technique in three cycles. This solution was transferred under a nitrogen atmosphere into the Schlenk tube with **3-Mes**. The reaction mixture was stirred for 16 h at room temperature in the dark. Toluene was evaporated using a Schlenk line and the crude product was redissolved in chloroform and injected into recycling GPC. Number of cycles can vary, but typically three cycles were sufficient to obtain the pure product (13 mg, 33%) as a green solid (mp > 389.9 °C decomposition).

^{1}H NMR (400 MHz, C_6D_6, ppm): δ 9.17 (d, J = 9.9 Hz, 4H), 8.40 (d, J = 9.3 Hz, 8H, two overlapped signals), 7.96 (t, J = 7.7 Hz, 2H), 7.24 (s, 8H), 2.49 (s, 12H), 2.07 (s, 24H).

^{13}C NMR (126 MHz, C_6D_6, ppm): δ 138.5, 137.8, 136.0, 135.8, 131.6, 130.1, 129.3, 125.5, 127.7, 127.3, 125.01, 124.2, 123.1, 123.0, 122.7, 121.8, 119.8, 21.5, 20.6.

IR: 2916, 1438, 1378, 1206, 1012, 883, 844, 802, 751, 728, 687, 626, 559 cm^{-1}.

HRMS (APCI) *m*/*z*: [*M* + H]$^{+}$ Calcd for $C_{80}H_{58}$ 1019.46113; Found 1019.46144.

3,12-Bis(3,5-di-*tert*-butylphenyl)-9-(*p*-tolyl)dibenzo[*cd,mn*]pyrene-4,8-dione (18-Tol)

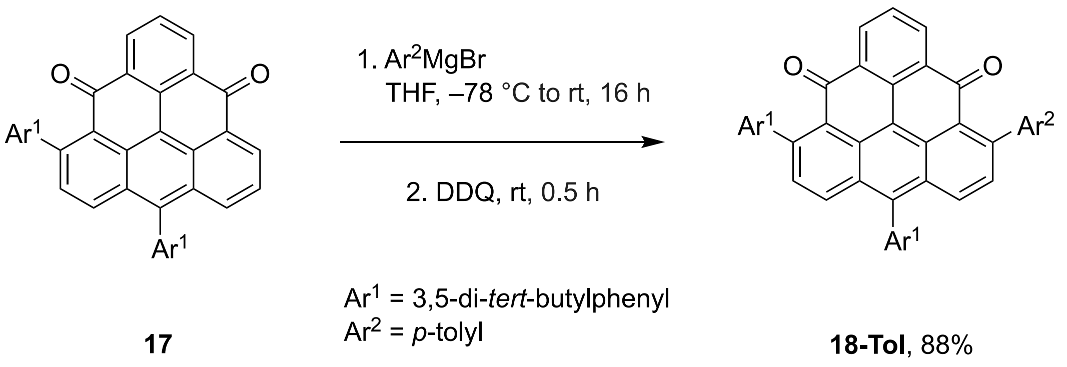

A solution of *p*-tolylmagnesium bromide (Ar^2MgBr; 0.37 mL, 0.11 mmol, 0.30 M in THF) was added dropwise to a cooled (−78 °C) solution of **17** (70 mg, 0.10 mmol) and LiCl (185 mg, 4.32 mmol) in dry THF (20 mL) under inert atmosphere and the reaction mixture was stirred at −78 °C for 2 h before it was allowed to warm to room temperature over 14 h. Then, 2,3-dichloro-5,6-dicyano-1,4-benzoquinone (DDQ; 26 mg, 0.11 mmol) was added, and the reaction mixture was stirred under air for 30 min before it was extracted with CH_2Cl_2 (3 × 10 mL). The combined organic layers were washed with water and brine, dried over anhydrous $MgSO_4$ and filtered. After evaporation of the solvents, the residue was purified by column chromatography (SiO_2, CH_2Cl_2, then CH_2Cl_2/MeOH, 50:1) to afford the product (70 mg, 88%) as a deep red solid (mp > 299.8 °C, decomposition).

^{1}H NMR (400 MHz, CD_2Cl_2, ppm): δ 8.58 (d, J = 6.5 Hz, 2H), 8.18 (dd, J = 8.9, 3.0 Hz, 2H), 7.72 (t, J = 1.8 Hz, 1H), 7.69 (t, J = 7.7 Hz, 1H), 7.61 (d, J = 8.8 Hz, 1H), 7.56 (d, J = 9.0 Hz, 1H), 7.54 (t, J = 2.4 Hz, 2H), 7.45–7.33 (m, 6H), 2.50 (s, 3H), 1.44 (s, 18H), 1.41 (s, 18H).

^{13}C NMR (101 MHz, CD_2Cl_2, ppm): δ 183.7, 183.6, 151.4, 151.3, 151.1, 150.4, 146.1, 143.2, 141.3, 137.5, 136.6, 134.59, 134.56, 134.3, 132.8, 132.7, 132.0, 131.7, 129.72, 129.69, 129.5, 128.7, 128.5, 128.2, 128.1, 126.2, 125.6, 125.5, 123.0, 122.7, 121.6, 117.2, 35.4, 31.7 (2 ×), 21.5. Three signals were not resolved due to signal overlap.

IR: 2956, 2866, 1645, 1595, 1541, 1393, 1362, 1247, 1230, 953, 876, 802 cm^{-1}.

HRMS (ESI) m/z: $[M + H]^+$ Calcd for $C_{57}H_{56}O_2$ 773.43531; Found 773.43426.

3,12-Bis(3,5-di-*tert*-butylphenyl)-9-(*p*-tolyl)-4,8-dihydrodibenzo[*cd*,*mn*]pyrene (4-Tol)

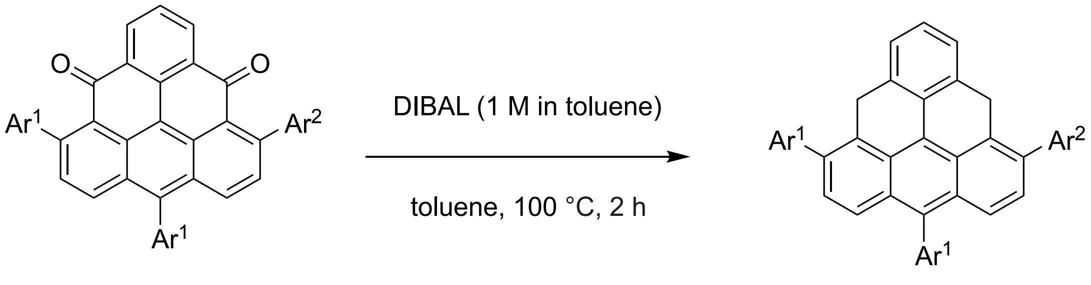

18-Tol Ar^1 = 3,5-di-*tert*-butylphenyl, Ar^2 = *p*-tolyl **4-Tol**, 68%

Compound **18-Tol** (50 mg, 0.072 mmol) was placed into a dried flask, dissolved in 10 mL of dry toluene and degassed by freeze-pump-thaw technique in three cycles. Then, diisobutylaluminiumhydrid (DIBAL; 0.37 mL, 0.37 mmol) was dropwise added at room temperature and the reaction mixture was heated to 100 °C and stirred for 2 hours. The excessive DIBAL was quenched by the addition of deoxygenated water (0.1 mL) and the solvent was evaporated using a Schlenk line. The residue was purified by column chromatography (SiO_2, pentane/CH_2Cl_2, 4:1) under inert conditions using deoxygenated silica gel and solvent. An orange, fluorescent compound was collected into a Schlenk flask and the solvent was evaporated using a Schlenk line to afford the product (37 mg, 68%) as a deep orange solid (mp > 107.9 °C decomposition).

Because of significant overlap in the aromatic region, the ^1H NMR and ^{13}C NMR (CD_2Cl_2) spectra are not fully described; please refer to the copies of the NMR spectra below.

IR: 2954, 2923, 2861, 1592, 1461, 1393, 1362, 1247, 899, 879, 815, 798, 718, 541 cm^{-1}.

HRMS (APCI) *m*/*z*: $[M + H]^+$ Calcd for $C_{57}H_{60}$ 745.47678; Found 745.47645.

1,4,9,12-Tetrakis(3,5-di-*tert*-butylphenyl)-6a,6b-di-*p*-tolyl-6a,6a^1,6b,6b^1-tetrahydroanthra[2,1,9,8-*wxyza*]benzo[*def*]naphtho[3,2,1,8,7-*qrstu*]pyranthrene (5-Tol) and **1,4,11,14-tetrakis(3,5-di-*tert*-butylphenyl)-7,17-di-*p*-tolyldianthra[2,1,9,8-*hijkl*:2',1',9',8'-*stuva*]dibenzo[*de,op*]pentacene (6-Tol)**

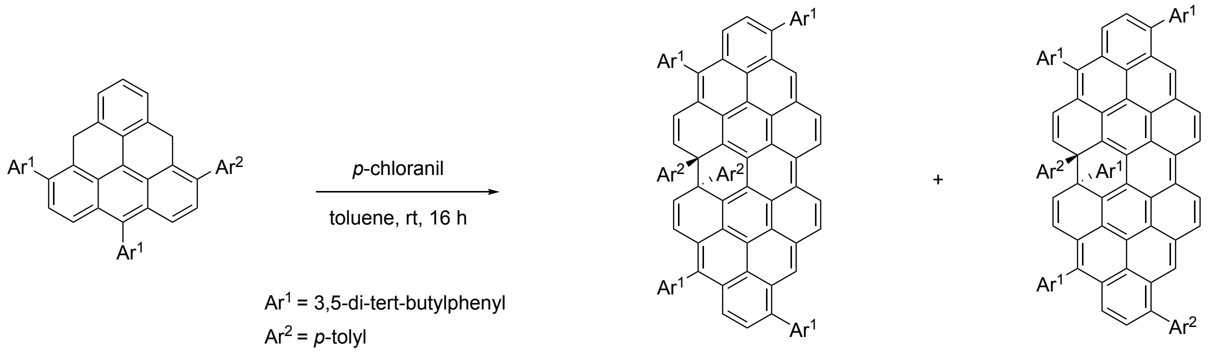

4-Tol **5-Tol**, 29% **6-Tol**, 17%

A mixture of dihydro-precursors of triaryltriangulene (**4-Tol**; 7 mg, 0.01 mol) was placed in a flame dried Schlenk tube and the atmosphere was exchanged three times with nitrogen. To another flame dried Schlenk tube, *p*-chloranil (11.5 mg, 47.0 µmol) was placed, dissolved in dry toluene (4 mL), and the solution was deoxygenated by freeze-pump-thaw technique in three cycles. This solution was transferred under a nitrogen atmosphere into the Schlenk tube with the dihydro-precursors. The reaction mixture was stirred for 16 h at room temperature in the dark. Toluene was evaporated using a Schlenk line and the crude product was redissolved in chloroform and injected into recycling GPC to obtain pure **5-Tol** and **6-Tol** as blue solids (2.3 mg, 29% and 1.2 mg, 17% respectively).

5-Tol:

^{1}H NMR (400 MHz, C_6D_6, ppm): δ 9.19 (s, 2H), 8.64 (d, *J* = 9.6 Hz, 2H), 8.07 (d, *J* = 7.9 Hz, 2H), 7.91 (d, *J* = 7.9 Hz, 2H), 7.87 (d, *J* = 9.5 Hz, 2H), 7.85 (d, *J* = 1.8 Hz, 4H), 7.81 (t, *J* = 1.9 Hz, 2H), 7.79 (t, *J* = 1.8 Hz, 2H), 7.72 (t, *J* = 1.7 Hz, 2H), 7.59 (t, *J* = 1.7 Hz, 2H), 7.36 (s, 2H), 7.33 (d, *J* = 7.6 Hz, 4H), 7.25 (d, *J* = 10.2 Hz, 2H), 6.35 (d, *J* = 8.5 Hz, 4H), 1.50 (s, 18H), 1.47 (s, 42H), 1.36 (s, 18H).

^{13}C NMR (126 MHz, C_6D_6, ppm): δ 151.6, 151.33, 151.32, 143.7, 141.7, 139.6, 138.8, 135.44, 134.8, 134.0, 132.9, 131.2, 129.8, 129.6, 129.1, 128.5, 126.7, 126.6, 126.38, 126.32, 126.1, 125.9, 125.5, 125.1, 124.8, 124.6, 124.14, 124.11, 123.5, 122.5, 121.51, 121.47, 121.45, 121.38, 55.3, 35.26, 35.25, 35.19, 31.85, 31.81, 31.75, 20.4.

IR: 2957, 1592, 1461, 1362, 1260, 1022, 879, 796 cm^{-1}.

HRMS (APCI) *m*/*z*: $[M + H]^+$ Calcd for $C_{114}H_{112}$ 1480.87585; Found 1480.87533.

6-Tol:

^1H NMR (500 MHz, C_6D_6, ppm): δ 9.25 (s, 1H), 9.10 (s, 1H), 8.59 (d, *J* = 4.2 Hz, 1H), 8.56 (d, *J* = 4.2 Hz, 1H), 8.14 (d, *J* = 7.9 Hz, 1H), 8.07 (d, *J* = 7.9 Hz, 1H), 7.98 (d, *J* = 1.8 Hz, 1H), 7.96 (s, 2H), 7.91 (*t*, J = 1.7 Hz, 1H), 7.86 (d, *J* = 8.4 Hz, 2H), 7.83 (2 × t (overlaid), *J* = 1.9 Hz, 1H + 1H), 7.82–7.80 (m, 2H), 7.79 (s, 2H), 7.78 (t, *J* = 1.8 Hz, 1H), 7.76 (t, *J* = 1.7 Hz, 1H), 7.64 (t, *J* = 1.7 Hz, 1H), 7.61 (d, *J* = 8.0 Hz, 2H), 7.55 (t, *J* = 1.7 Hz, 1H), 7.47 (d, *J* = 10.1 Hz, 2H), 7.37 (d, *J* = 2.0 Hz, 1H), 7.36–7.34 (m, 2H), 7.24 (d, *J* = 8.1 Hz, 2H), 6.80 (t, *J* = 1.7 Hz, 1H), 6.29 (d, *J* = 8.5 Hz, 2H), 2.31 (s, 3H), 1.57 (s, 9H), 1.49 (s, 9H), 1.45 (s, 9H), 1.44 (2 × s (overlaid), 3H + 18H), 1.30 (s, 9H), 0.75 (s, 18H).

^{13}C NMR (126 MHz, C_6D_6, ppm): δ 173.3, 151.8, 151.6, 151.4, 151.3, 149.3, 145.0, 141.6, 139.7, 139.4, 139.1, 138.6, 137.7, 137.5, 137.1, 135.4, 135.3, 134.6, 133.2, 132.9, 131.0, 130.2, 130.2, 129.9, 129.8, 129.6, 129.38, 129.35, 128.3, 128.2, 128.1, 127.9, 126.57, 126.55, 126.34, 126.31, 126.28, 126.0, 125.9, 125.8, 125.7, 125.5, 125.3, 124.9, 124.73, 124.70, 124.68, 124.5, 124.4, 124.2, 123.7, 123.4, 122.5, 122.2, 121.64, 121.62, 121.50, 121.48, 121.4, 119.7, 56.2, 54.4, 35.4, 35.3, 35.23, 35.16, 32.0, 31.84, 31.79, 31.7, 21.3. Please note that some signals lie beneath the residual solvent peak; however, all are reported, as their shifts were unambiguously determined from 2D spectra.

IR: 2921, 2852, 1460, 1377, 1261, 1026, 802, 727 cm^{-1}.

HRMS (APCI) *m*/*z*: $[M + H]^+$ Calcd for $C_{114}H_{112}$ 1480.87585; Found 1480.87533.

3,12-Bis(3,5-di-*tert*-butylphenyl)-9-(3,5-dimethylphenyl)dibenzo[*cd*,*mn*]pyrene-4,8-dione (18-Xyl)

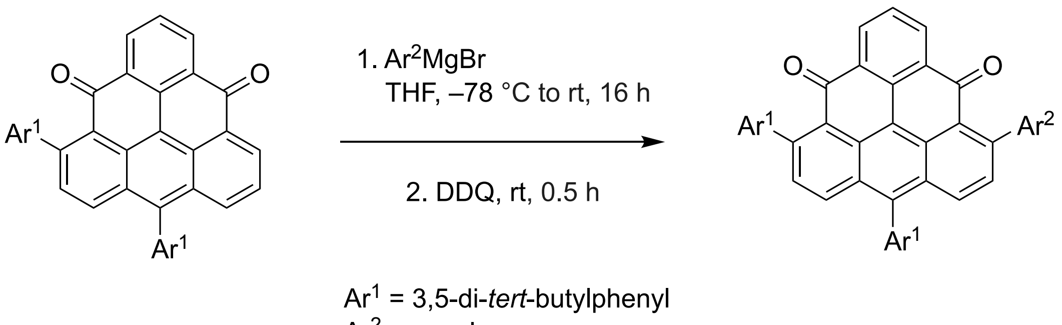

17 **18-Xyl**, 55%

A solution of (3,5-dimethylphenyl)magnesium bromide (Ar^2MgBr; 0.14 mL, 0.056 mmol, 0.40 M in THF) was added dropwise to a cooled (−78 °C) solution of **17** (32 mg, 0.047 mmol) in dry THF (10 mL) under inert atmosphere. The reaction mixture was stirred at −78 °C for 2 h before it was allowed to warm to room temperature over 14 h. Then, DDQ (11.7 mg, 51.6 μmol) was added, and the reaction mixture was stirred under air for 30 min. The reaction mixture was extracted with CH_2Cl_2 (3 × 10 mL). The combined organic layers were washed with water, brine, dried over anhydrous $MgSO_4$, and filtered. After evaporation of the solvents, the residue was purified by column chromatography (SiO_2, CH_2Cl_2, then CH_2Cl_2/MeOH (50:1)) to afford the product (20 mg, 55%) as a deep red solid (mp 227.6–228.5 °C).

^{1}H NMR (400 MHz, $CDCl_3$, ppm): δ 8.68 (dd, J = 7.7, 1.5 Hz, 1H), 8.64 (dd, J = 7.6, 1.5 Hz, 1H), 8.15 (dd, J = 8.9, 1.2 Hz, 2H), 7.69 (t, J = 7.7 Hz, 1H), 7.68 (t, J = 1.8 Hz, 1H), 7.64 (d, J = 8.9 Hz, 1H), 7.55 (d, J = 8.8 Hz, 1H), 7.52 (t, J = 1.8 Hz, 1H), 7.39 (d, J = 1.8 Hz, 2H), 7.36 (d, J = 1.8 Hz, 2H), 7.09 (s, 1H), 7.08 (s, 2H), 2.43 (s, 3H), 1.43 (s, 9H), 1.41 (s, 9H).

^{13}C NMR (126 MHz, $CDCl_3$, ppm): δ 183.7, 183.6, 151.4, 151.0, 150.68, 150.66, 145.6, 144.0, 142.6, 138.1, 136.4, 134.4, 134.1, 134.0, 133.0, 132.9, 131.8, 131.7, 131.4, 131.3, 129.4, 129.3, 128.4, 128.1, 128.0, 126.0, 125.4, 125.2, 122.6, 122.3, 121.4, 117.1, 35.2, 31.7, 21.7. Two signals were not resolved due to signal overlap.

IR: 2956, 2865, 1646, 1597, 1541, 1467, 1394, 1362, 1320, 1278, 1247, 1230, 1205, 963, 851, 837, 806, 757, 712, 695, 684, 657 cm^{-1}.

HRMS (ESI) *m*/*z*: [*M* + H]$^+$ Calcd for $C_{58}H_{59}O_2$ 787.45096; Found 787.45032.

3,12-Bis(3,5-di-*tert*-butylphenyl)-9-(3,5-dimethylphenyl)-4,8-dihydrodibenzo-[*cd,mn*]pyrene (4-Xyl)

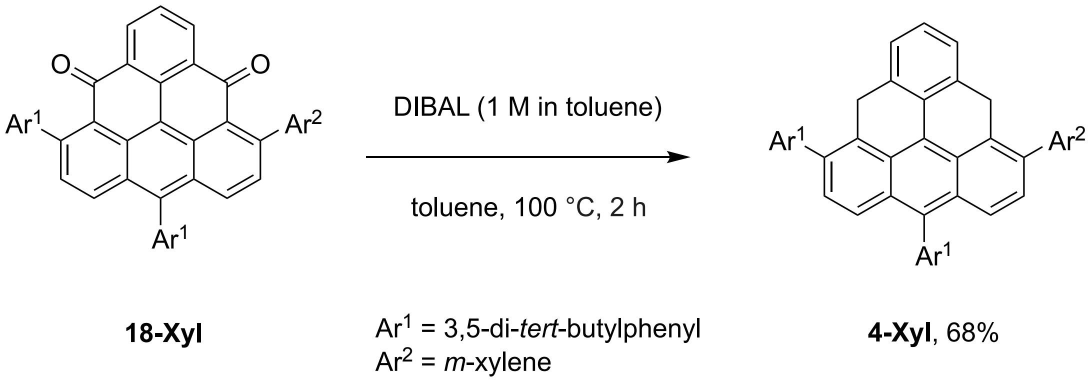

Compound **18-Xyl** (25 mg, 32 μmol) was placed into a dried flask, dissolved in 10 mL of dry toluene and degassed by freeze-pump-thaw technique in three cycles. Then, DIBAL (0.16 mL, 0.16 mmol) was dropwise added at room temperature and the reaction mixture was heated to 100 °C and stirred for 2 hours. The excessive DIBAL was quenched by the addition of deoxygenated water (0.1 mL) and the solvent was evaporated using a Schlenk line. The residue was purified by column chromatography (SiO_2, pentane/CH_2Cl_2, 5:1) under inert conditions using deoxygenated silica gel and solvent. A yellow, fluorescent compound was collected into a Schlenk flask and the solvent was evaporated using a Schlenk line to afford the product (15 mg, 62%) as a pale yellow solid (mp > 113.2 °C decomposition).

Because of significant overlap in the aromatic region, the ^1H NMR and ^{13}C NMR (CD_2Cl_2) spectra are not fully described; please refer to the copies of the NMR spectra below.

IR: 2956, 2923, 2864, 1593, 1462, 1408, 1393, 1362, 1311, 1283, 1246, 1207, 1189, 1156, 1038, 1007, 899, 878, 852, 822, 798, 718 cm^{-1}.

HRMS (APCI) *m*/*z*: $[M + H]^+$ Calcd for $C_{58}H_{62}$ 759.49243; Found 759.49357.

1,4,9,12-Tetrakis(3,5-di-*tert*-butylphenyl)-6a,6b-bis(3,5-dimethylphenyl)-6a,6a^1,6b,6b^1-tetrahydroanthra[2,1,9,8-*wxyza*]benzo[*def*]naphtho[3,2,1,8,7-*qrstu*]pyranthrene (5-Xyl)

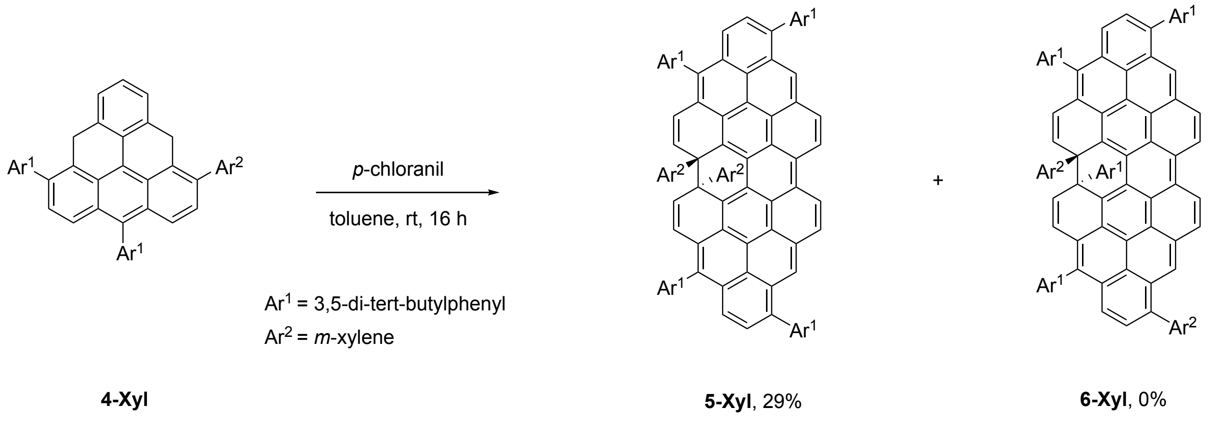

A mixture of dihydro-precursors of triaryltriangulene (**4-Xyl**; 5.0 mg, 6.6 µmol) was placed in a flame-dried Schlenk tube and the atmosphere was exchanged three times with nitrogen. To another flame-dried Schlenk tube, *p*-chloranil (8.1 mg, 33 µmol) was placed, dissolved in dry toluene (4 mL), and the solution was deoxygenated by freeze-pump-thaw technique in three cycles. This solution was transferred under a nitrogen atmosphere into the Schlenk tube with the dihydro-precursors. The reaction mixture was stirred for 16 h at room temperature in the dark. Toluene was evaporated using a Schlenk line and the crude product was redissolved in chloroform and injected into recycling GPC to obtain pure **5-Xyl** (1.3 mg, 26%) as a blue solid.

^{1}H NMR (600 MHz, C_6D_6, ppm): δ 9.11 (s, 2H), 8.60 (d, J = 9.6 Hz, 2H), 8.07 (d, J = 7.8 Hz, 2H), 7.91 (d, J = 7.8 Hz, 2H), 7.83 (d, J = 1.7 Hz, 4H), 7.81 (d, J = 2.0 Hz, 4H),7.78 (t, J = 1.8 Hz, 2H), 7.58 (t, J = 1.7 Hz, 2H), 7.35 (d, J = 10.0 Hz, 2H), 7.22 (d, J = 10.4 Hz, 2H), 7.21 (s, 2H), 7.06 (d, J = 7.3 Hz, 2H), 6.06 (s, 2H), 1.57 (s, 12H), 1.55 (s, 18H), 1.47 (s, 36H), 1.35 (s, 18H). Two signals are not described, due to overlap with the signals at 7.83–7.81 ppm. Please refer to the copy of the spectrum, and the assignment of resonances.

^{13}C NMR (151 MHz, C_6D_6, ppm): δ 151.6, 151.4, 151.3, 147.3, 141.7, 139.5, 139.0, 137.9, 137.5, 136.8, 135.3, 133.9, 133.1, 131.2, 129.8, 129.5, 129.3, 127.1, 126.4, 126.16, 126.13, 126.11, 125.9, 124.9, 124.8, 124.6, 124.08, 124.05, 123.4, 122.5, 121.44, 121.42, 121.3, 56.1, 34.29, 35.23, 35.19, 32.4, 31.9, 31.81, 31.76, 21.43, 21.35. Please note that some signals lie beneath the residual solvent peak; however, all are reported, as their shifts were unambiguously determined from 2D spectra.

IR: 2953, 2922, 2854, 1592, 1460, 1362, 1247, 1022, 796, 717 cm^{-1}.

HRMS (APCI) *m*/*z*: [*M* + H]$^+$ Calcd for $C_{116}H_{116}$ 1509.91498; Found 1509.91553.

3,9,12-Tris(3,5-di-*tert*-butylphenyl)-4,8-dihydrodibenzo[*cd*,*mn*]pyrene (4-tBP)

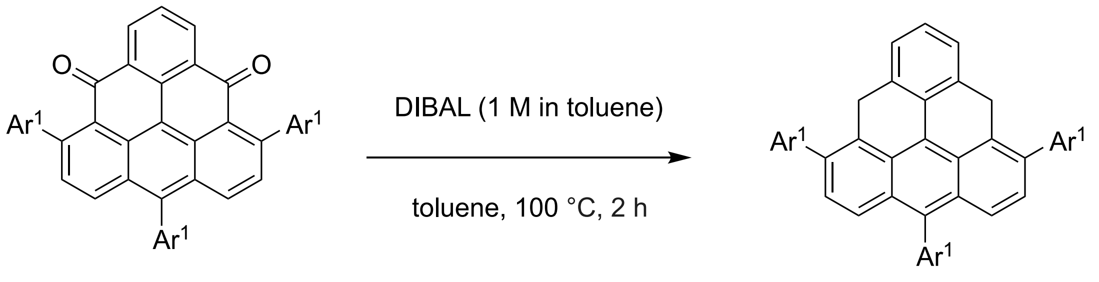

18-tBP Ar[1] = 3,5-di-*tert*-butylphenyl **4-tBP**, 52%

Compound **18-tBP** (20 mg, 23 µmol) was placed into a dried flask, dissolved in 10 mL of dry toluene and degassed by freeze-pump-thaw technique in three cycles. Then, DIBAL (0.12 mL, 0.12 mmol) was dropwise added at room temperature and the reaction mixture was heated to 100 °C and stirred for 2 hours. The excessive DIBAL was quenched by the addition of deoxygenated water (0.1 mL) and the solvent was evaporated using a Schlenk line. The residue was purified by column chromatography (SiO_2, pentane/CH_2Cl_2, 5:1) under inert conditions using deoxygenated silica gel and solvent. A yellow, fluorescent compound was collected into a Schlenk flask and the solvent was evaporated using a Schlenk line to afford the title compound (10 mg, 52%) as a pale yellow solid (mp 113.2–115.9 °C).

Because of significant overlap in the aromatic region, the ^{1}H NMR and ^{13}C NMR (C_6D_6) spectra are not fully described; please refer to the copies of the NMR spectra below.

IR: 2955, 2923, 2864, 1592, 1462, 1393, 1362, 1246, 1202, 899, 718 cm^{-1}.

HRMS (APCI) *m*/*z*: [*M* + H]$^+$ Calcd for $C_{64}H_{75}$ 843.58605; Found 843.58561.

4. Copies of the NMR and HRMS Spectra

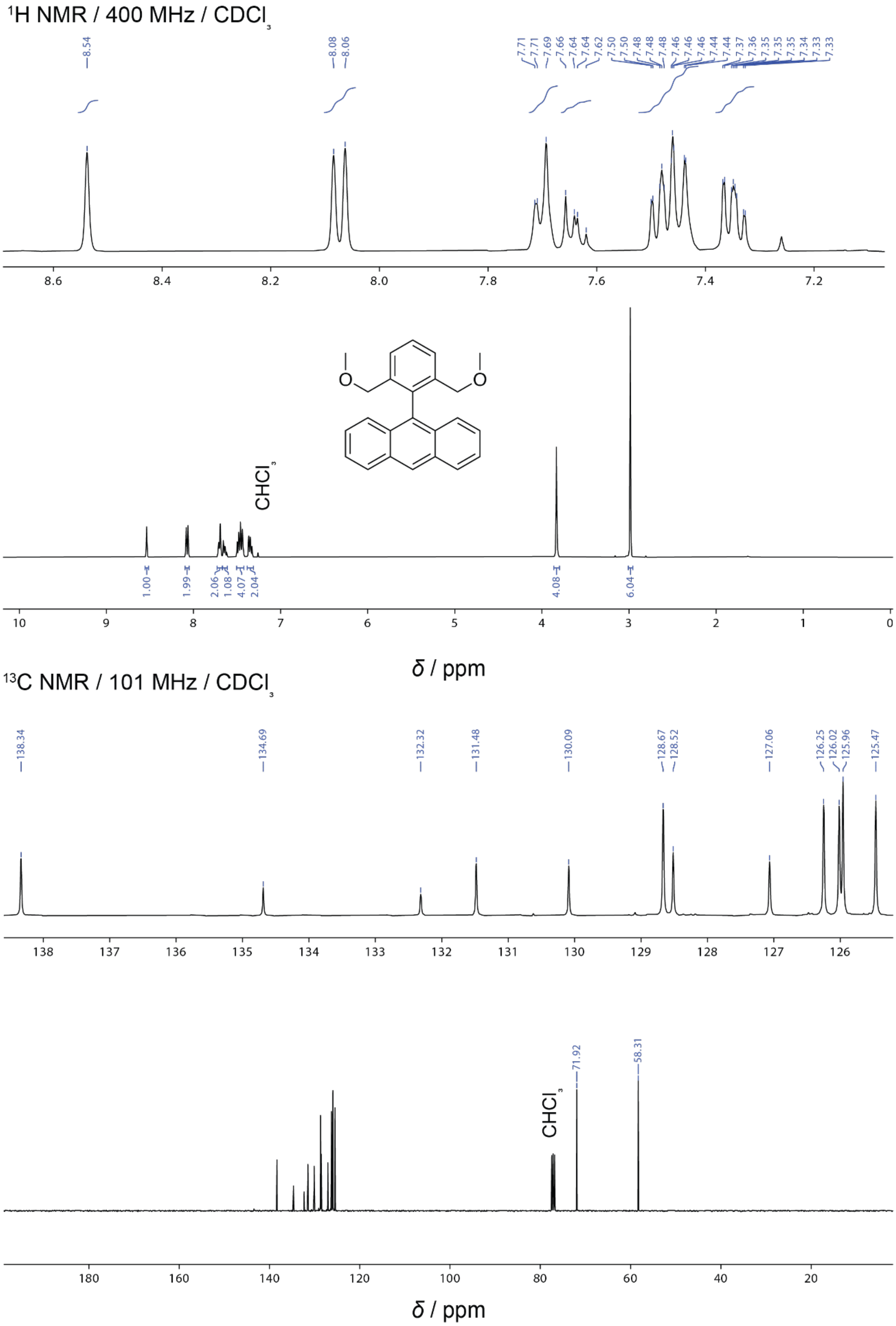

Figure S14. ^{1}H NMR (top) and ^{13}C NMR (bottom) of **12** in $CDCl_3$.

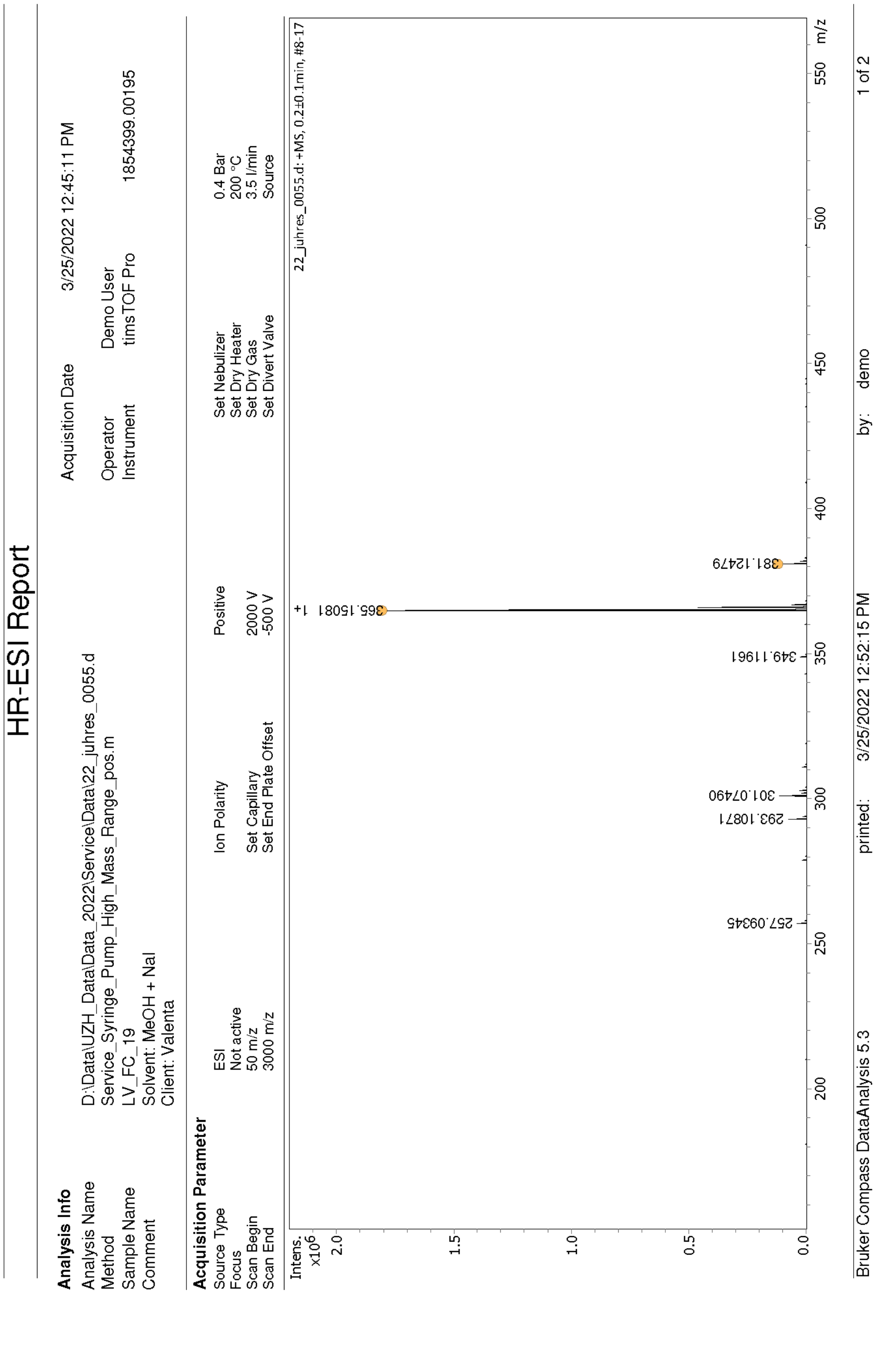

Figure S15. HRMS (ESI) of **12**.

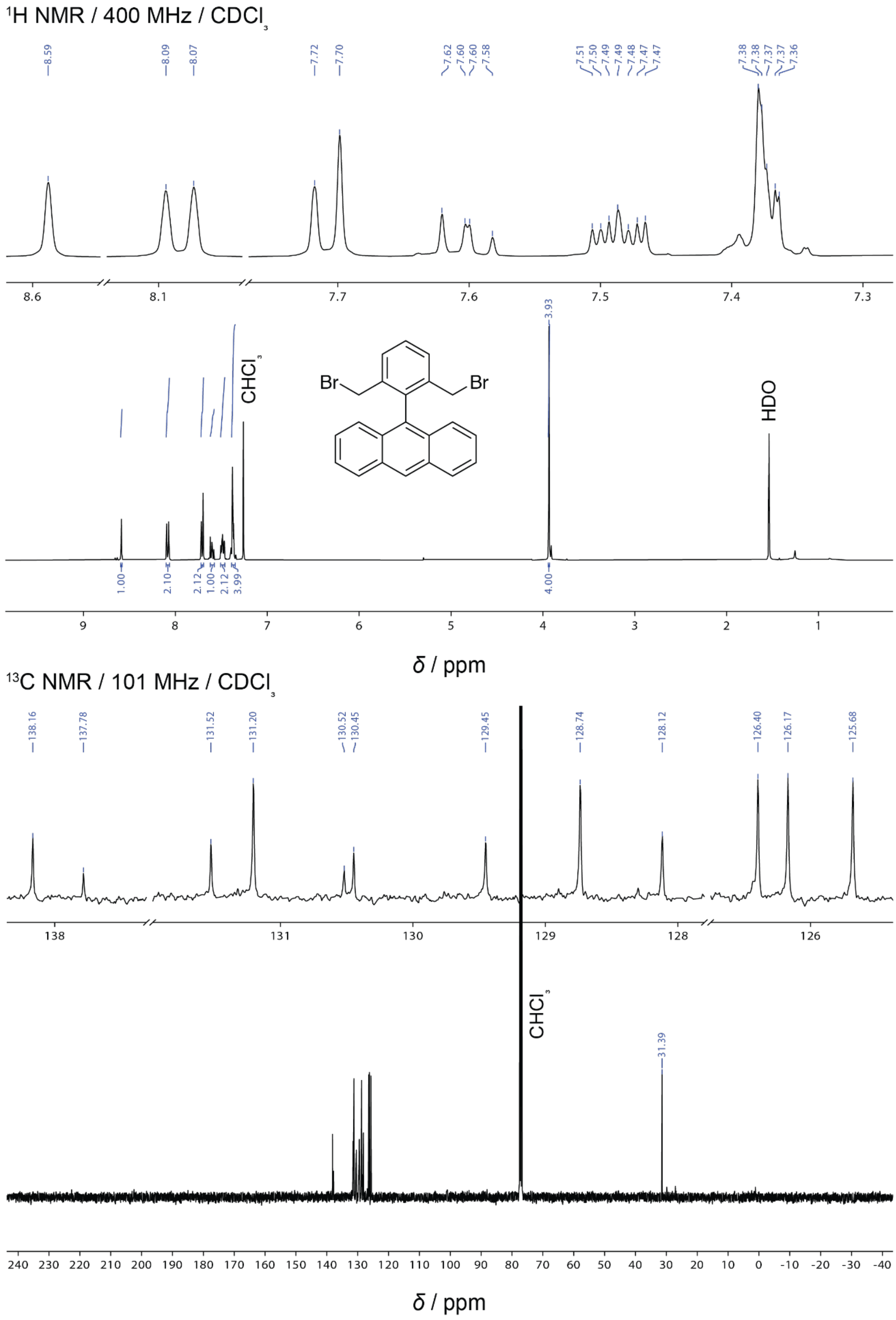

Figure S16. ^{1}H NMR (top) and ^{13}C NMR (bottom) of **13** in $CDCl_3$.

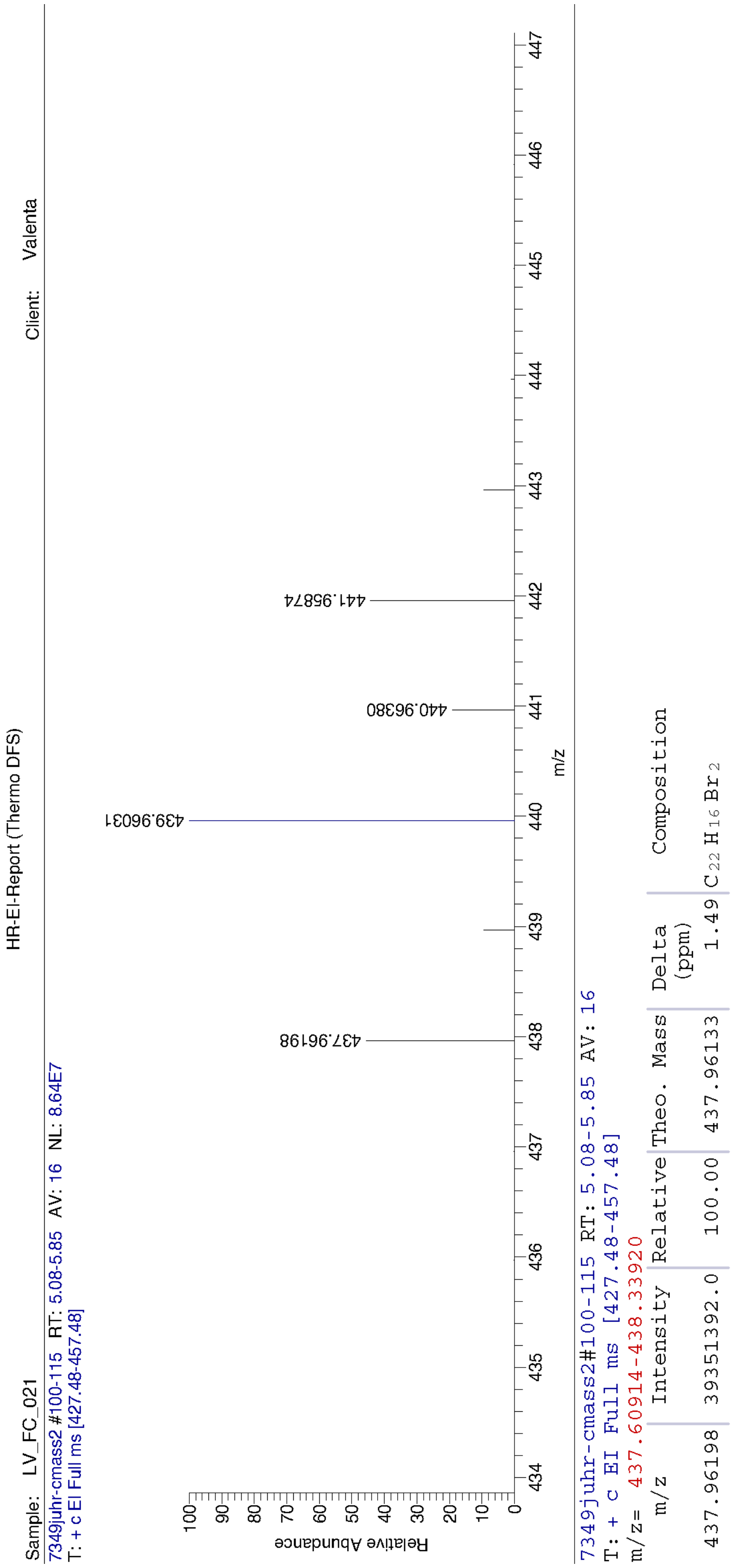

Figure S17. HRMS (EI) of **13**.

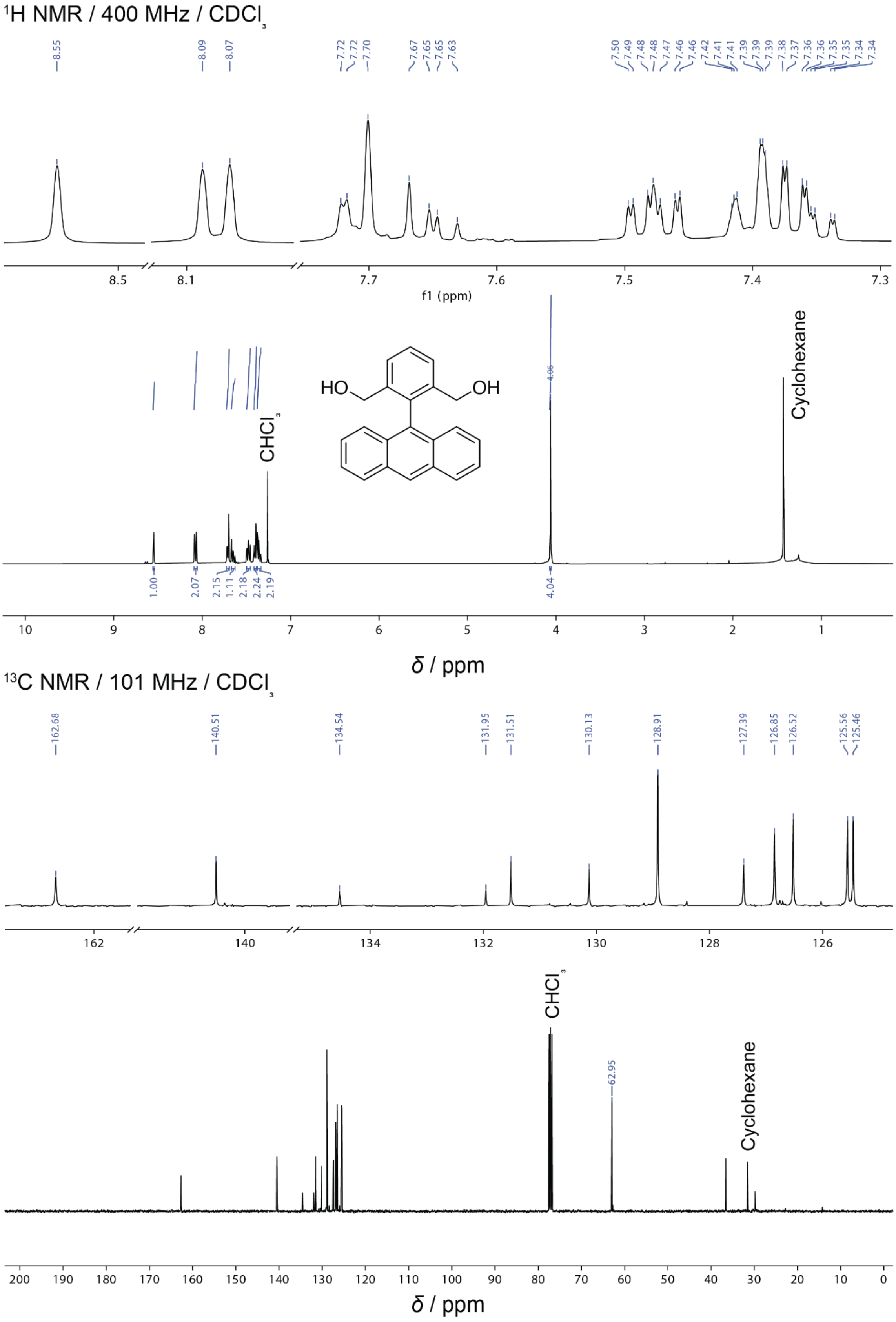

Figure S18. ^{1}H NMR (top) and ^{13}C NMR (bottom) of **14** in $CDCl_3$.

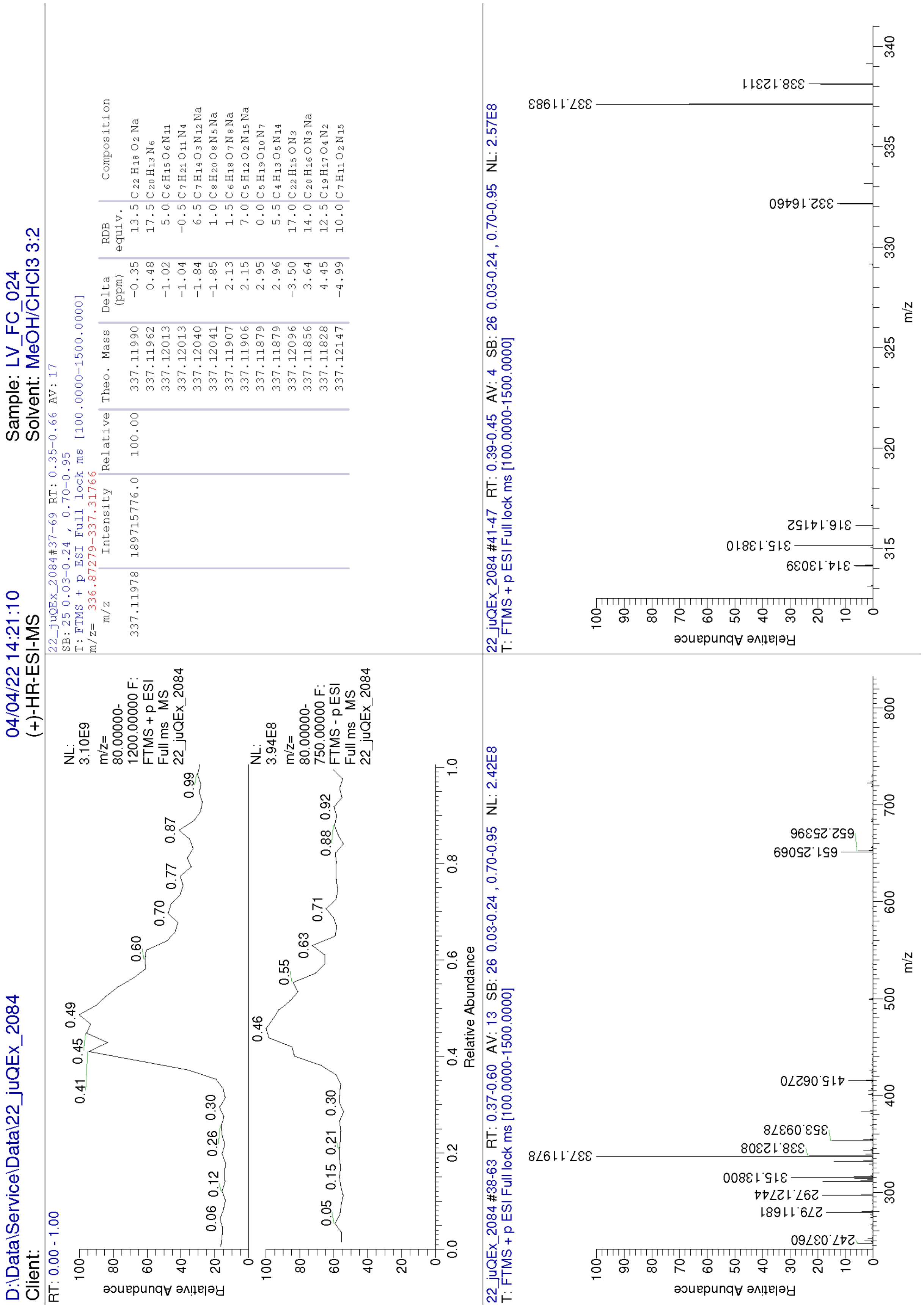

Figure S19. HRMS (ESI) of **14**.

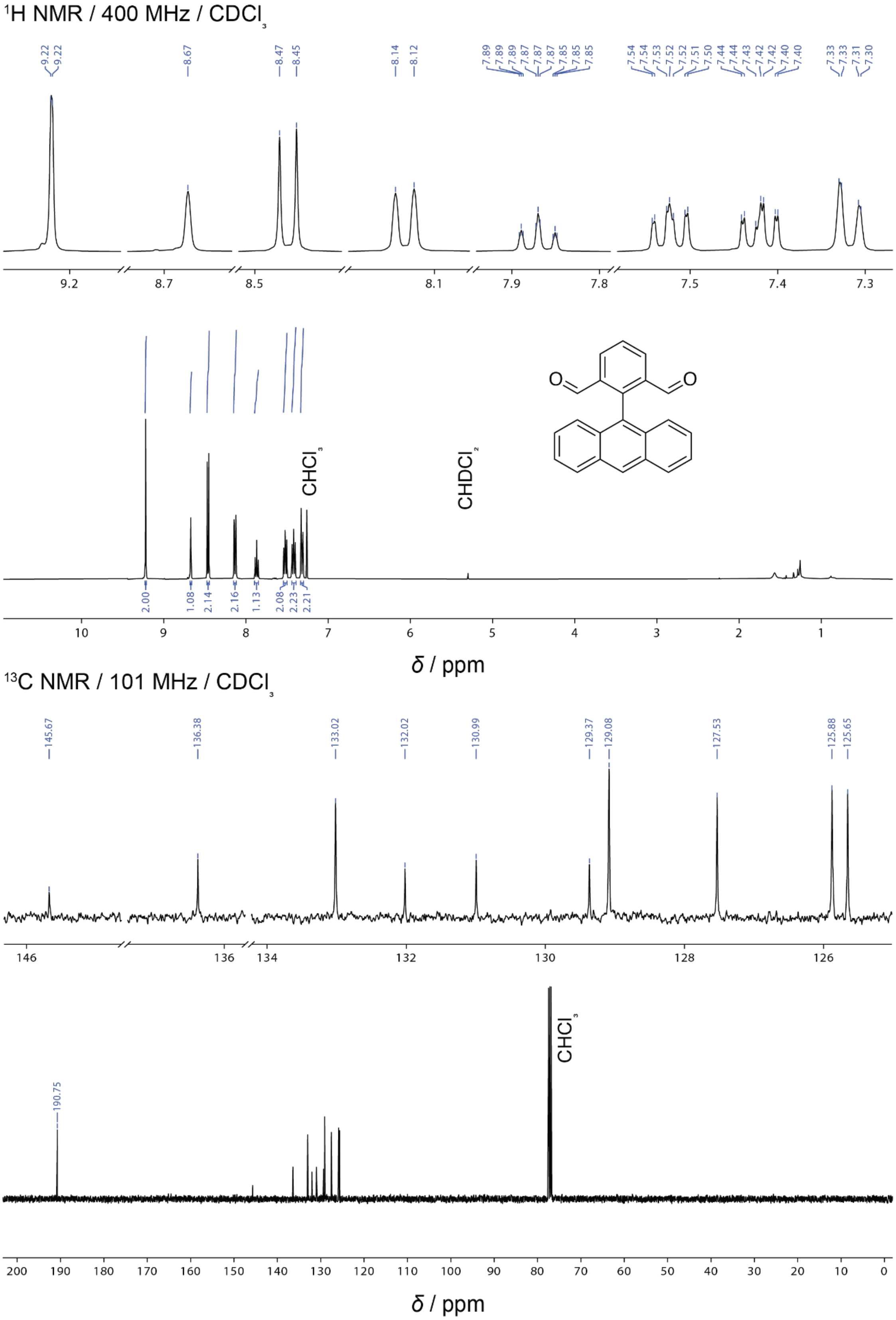

Figure S20. ^{1}H NMR (top) and ^{13}C NMR (bottom) of **15** in $CDCl_3$.

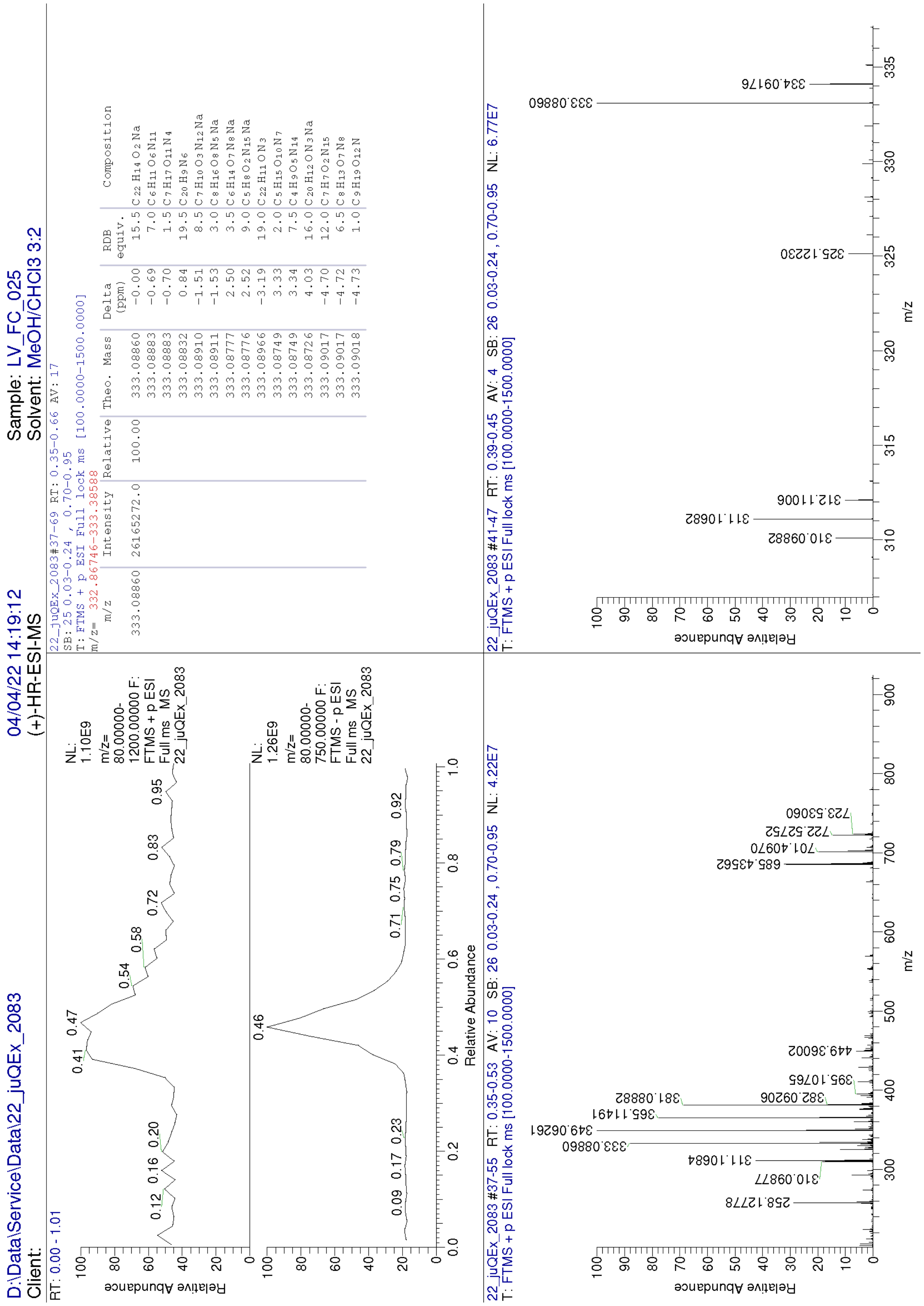

Figure S21. HRMS (ESI) of **15.**

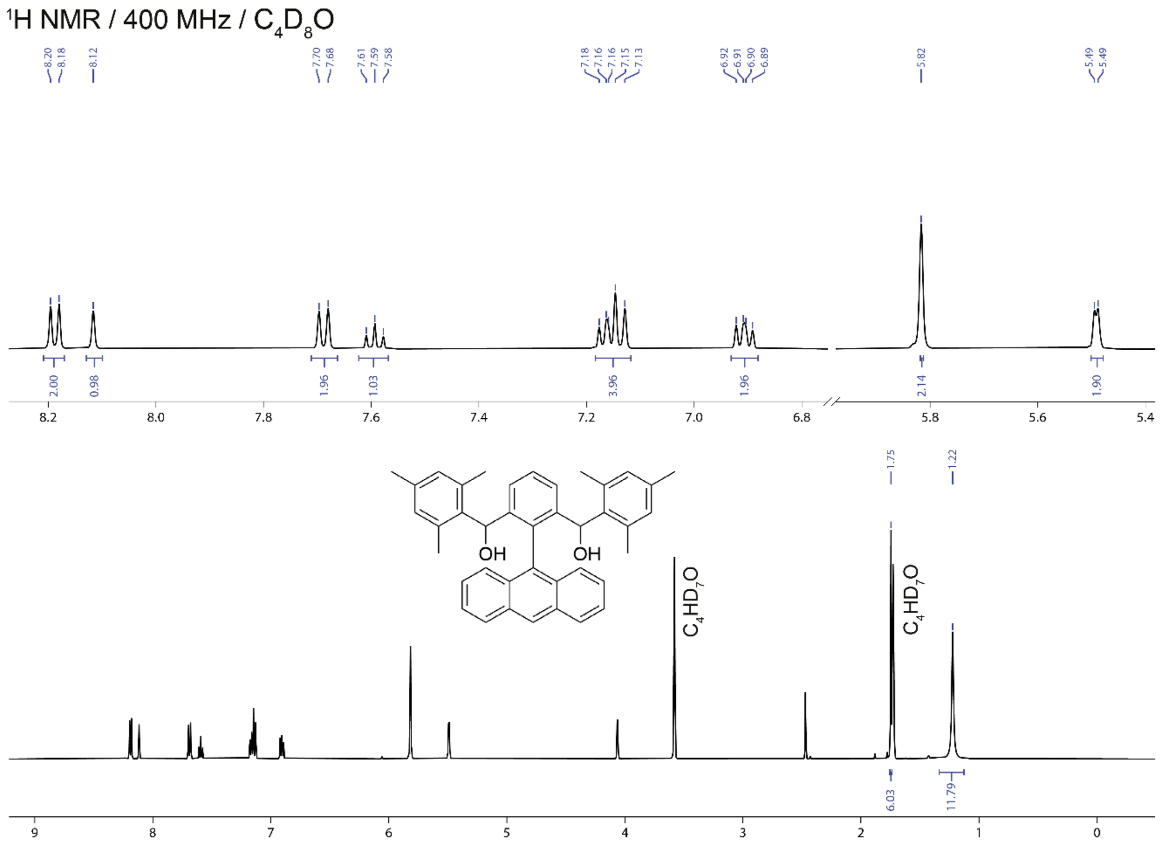

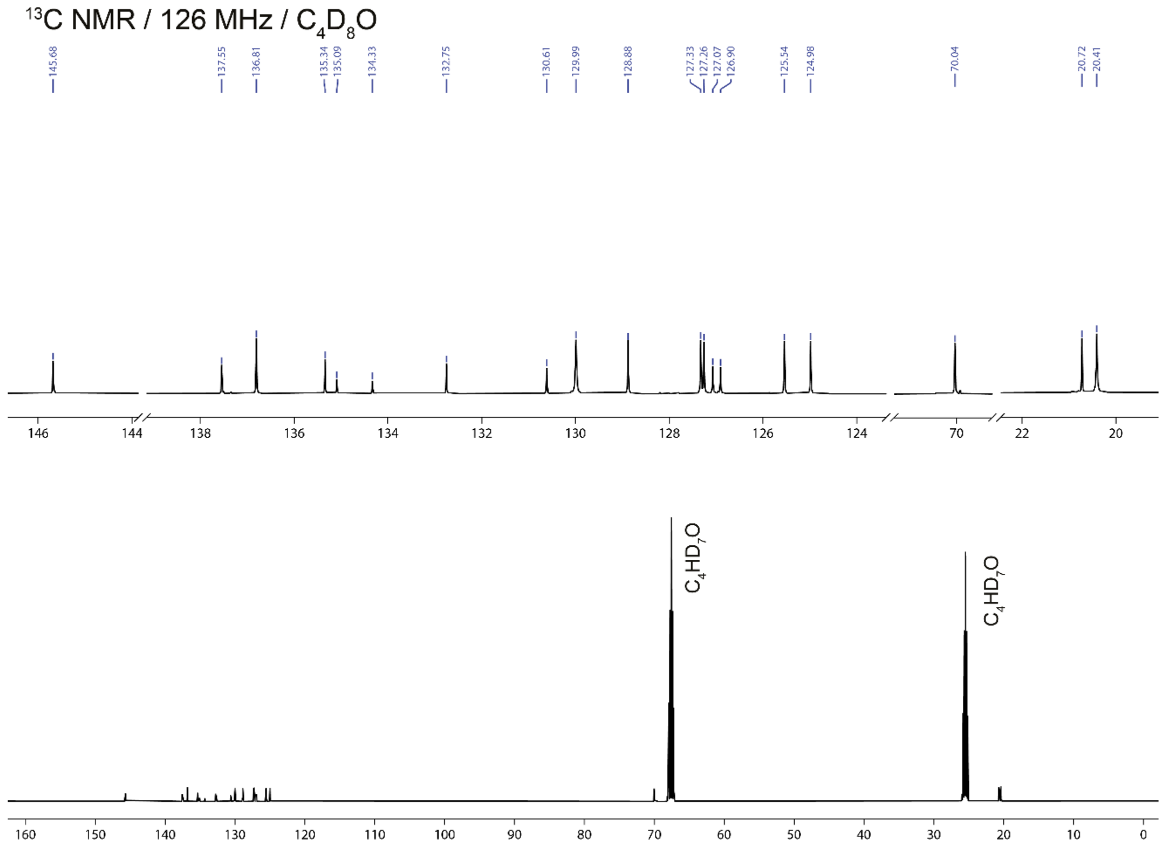

Figure S22. ^{1}H NMR (top) and ^{13}C NMR (bottom) of **16** in THF-d_8.

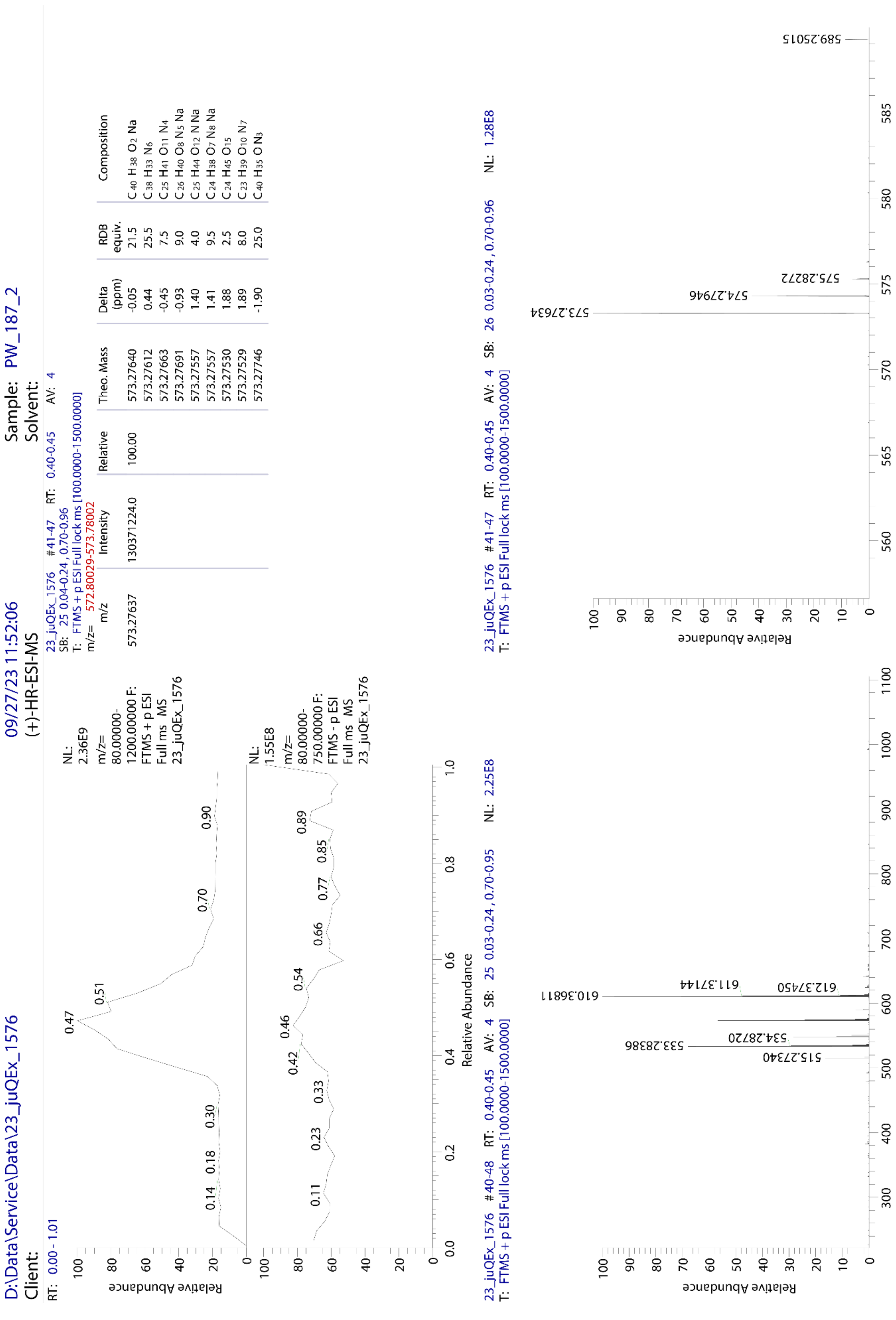

Figure S23. HRMS (ESI) of **16**.

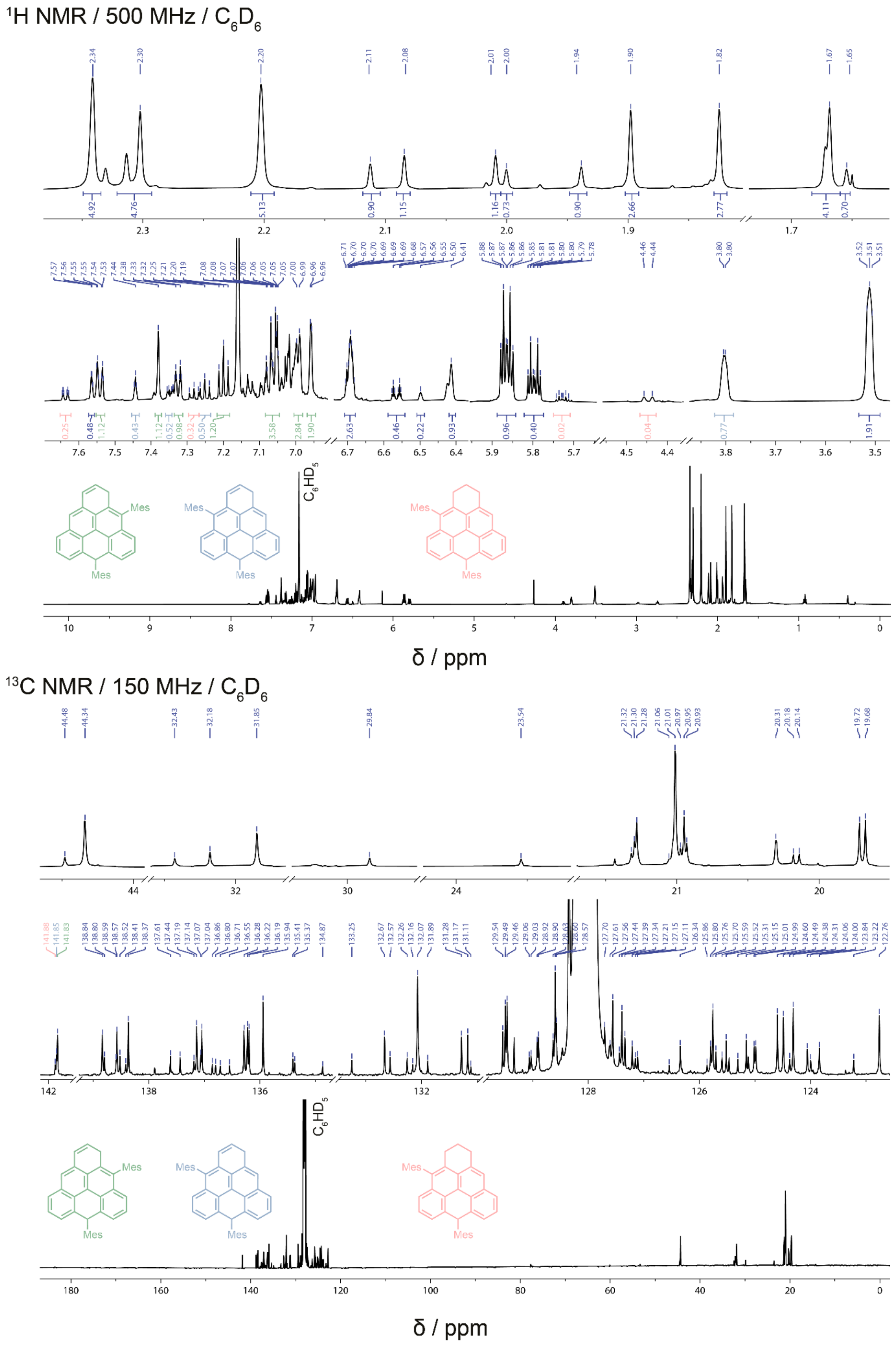

Figure S24. ^{1}H NMR (top) and ^{13}C NMR (bottom) of **3-Mes** in C_6D_6.

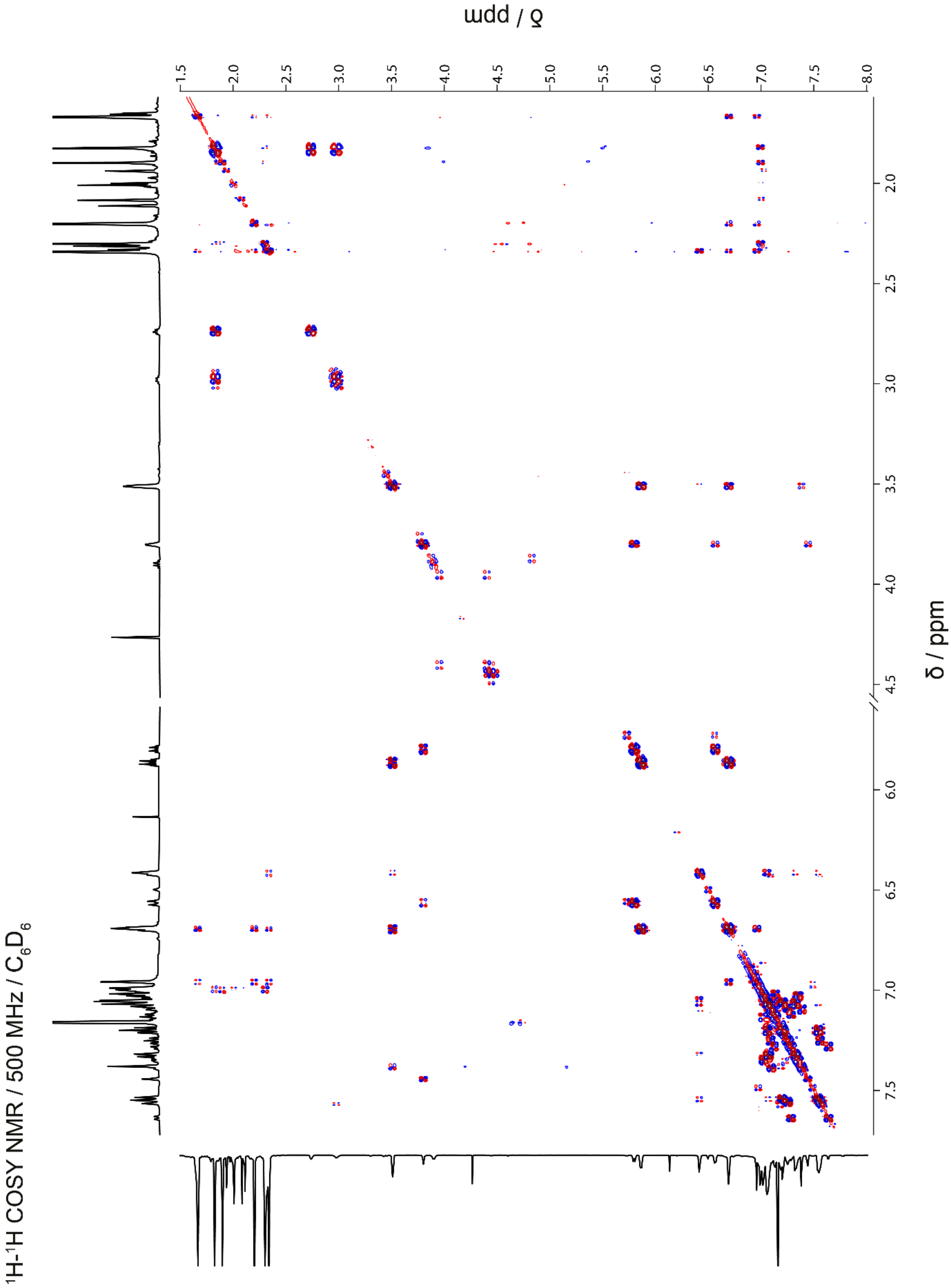

Figure S25. ^{1}H–^{1}H COSY NMR of **3-Mes** in C_6D_6.

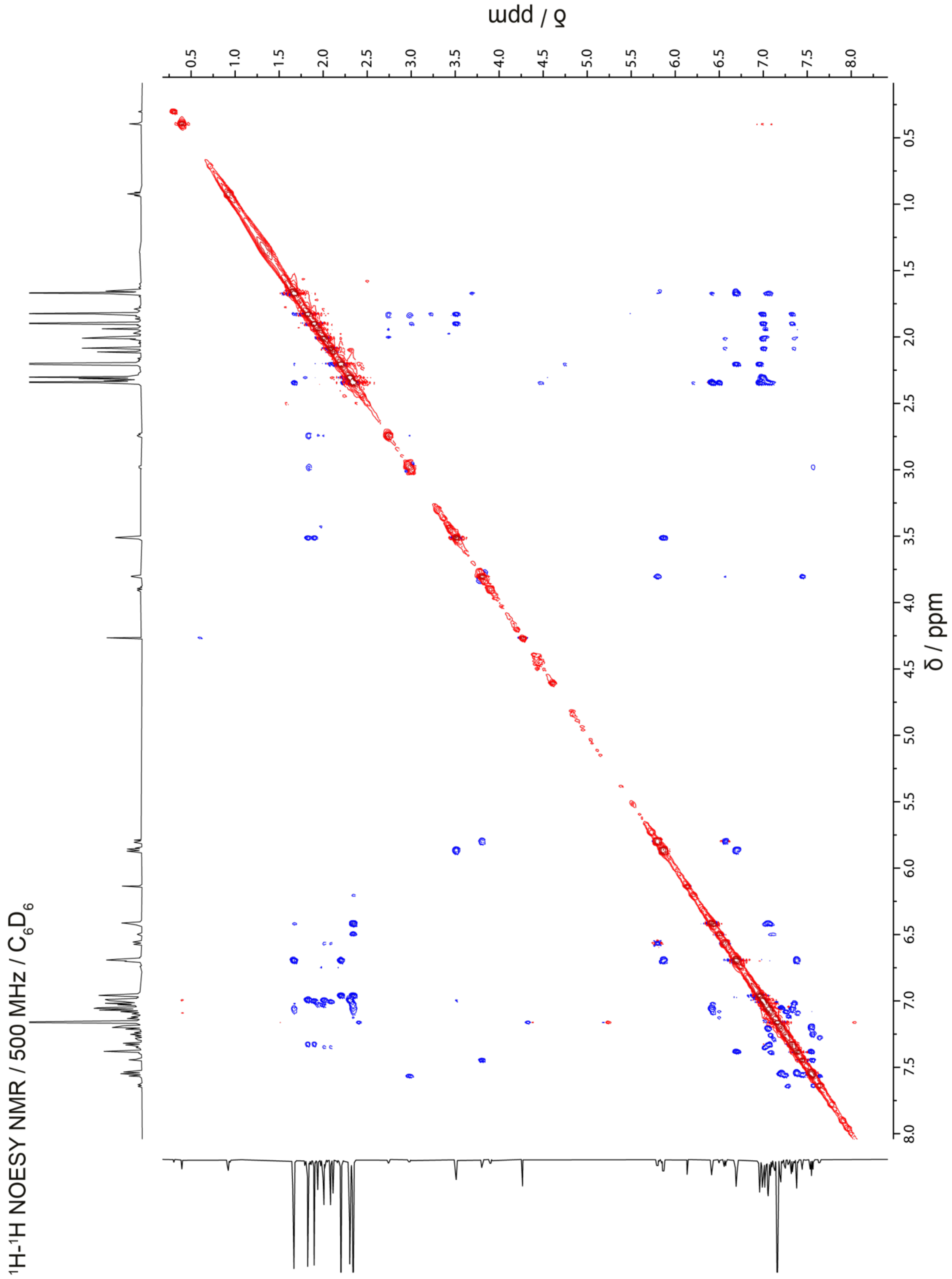

Figure S26. ^1H–^1H NOESY NMR of **3-Mes** in C_6D_6.

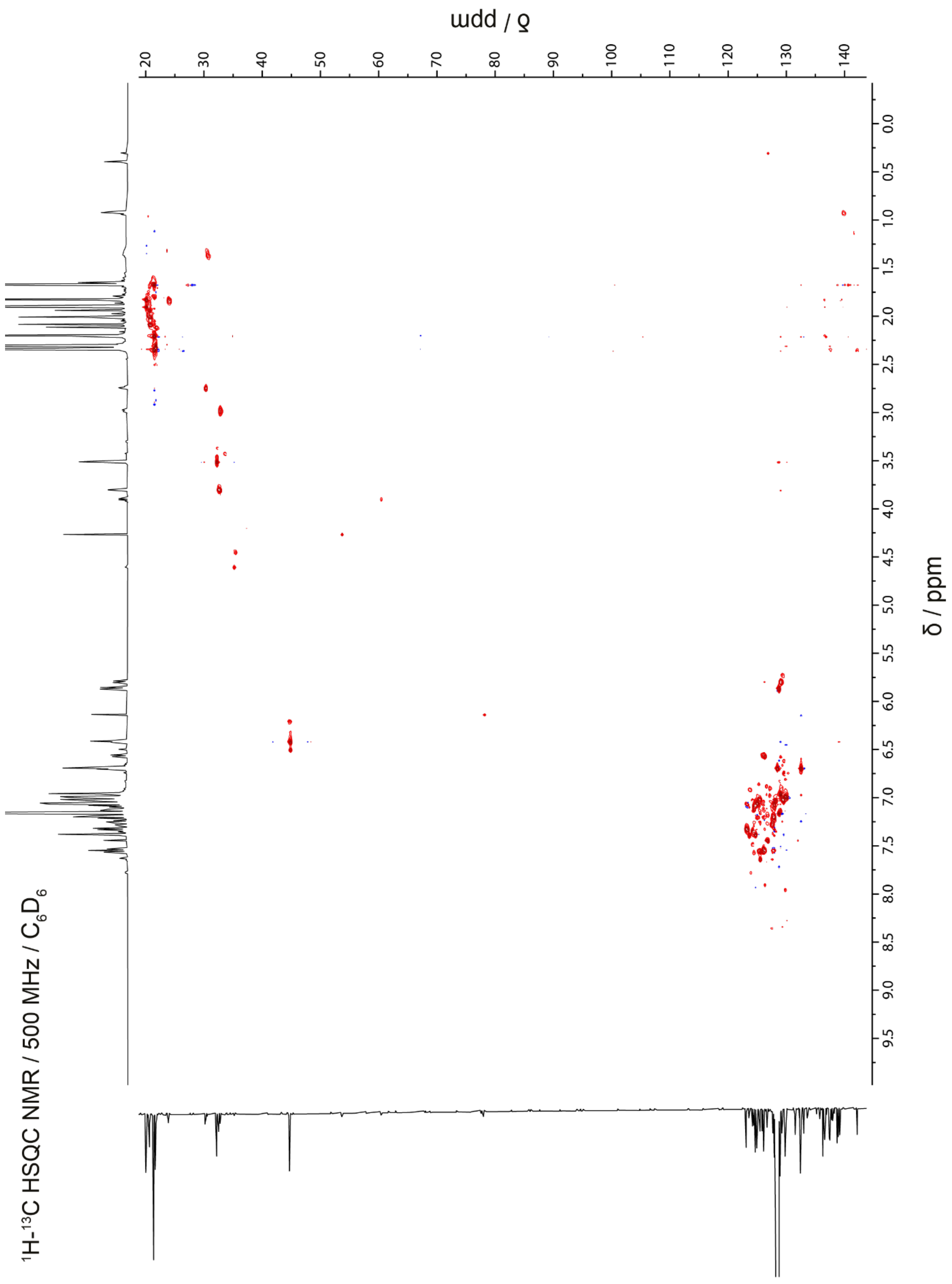

Figure S27. ^{1}H–^{13}C HSQC NMR of **3-Mes** in C_6D_6.

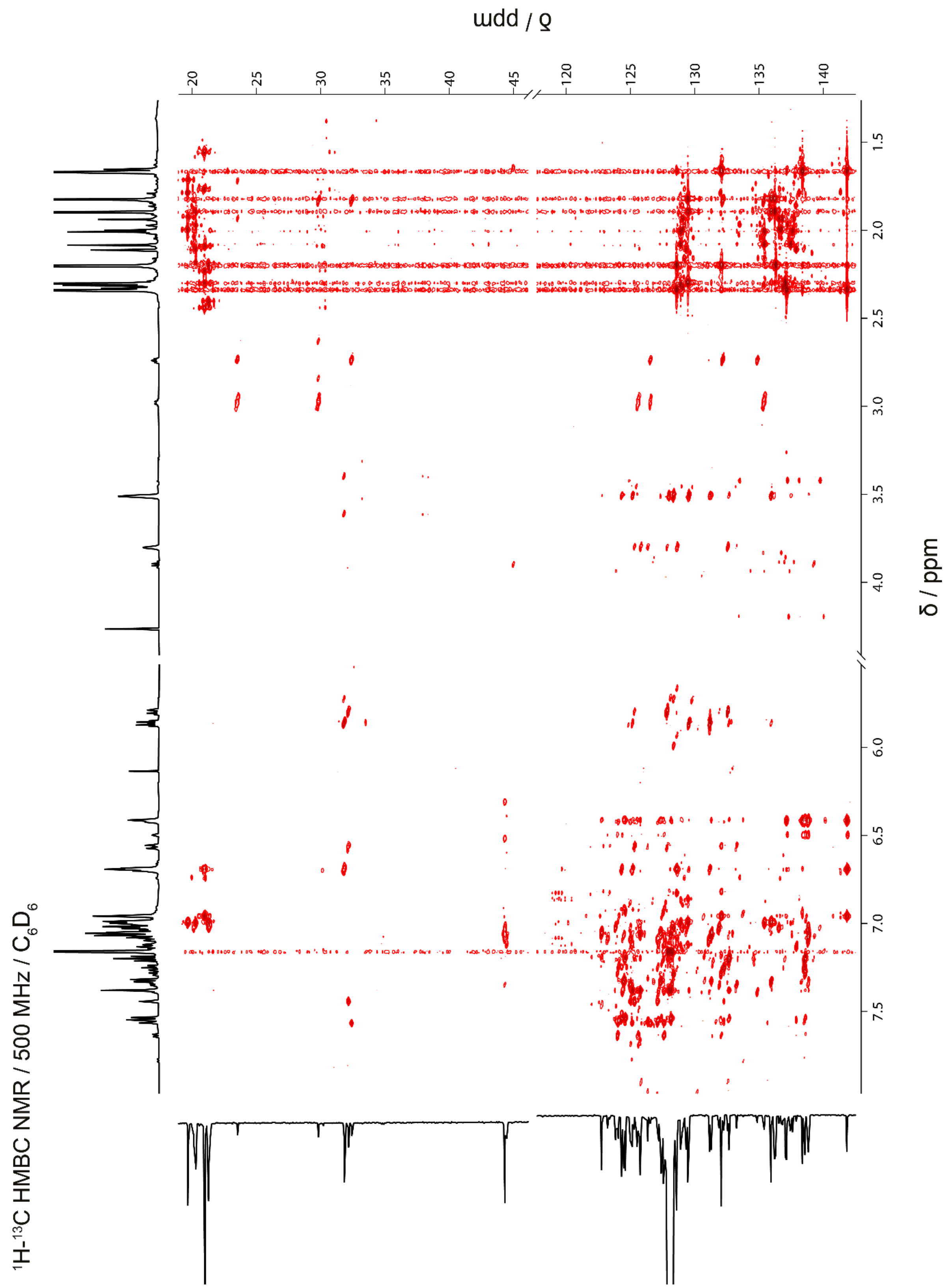

Figure S28. $^{1}H–^{13}C$ HMBC NMR of **3-Mes** in C_6D_6.

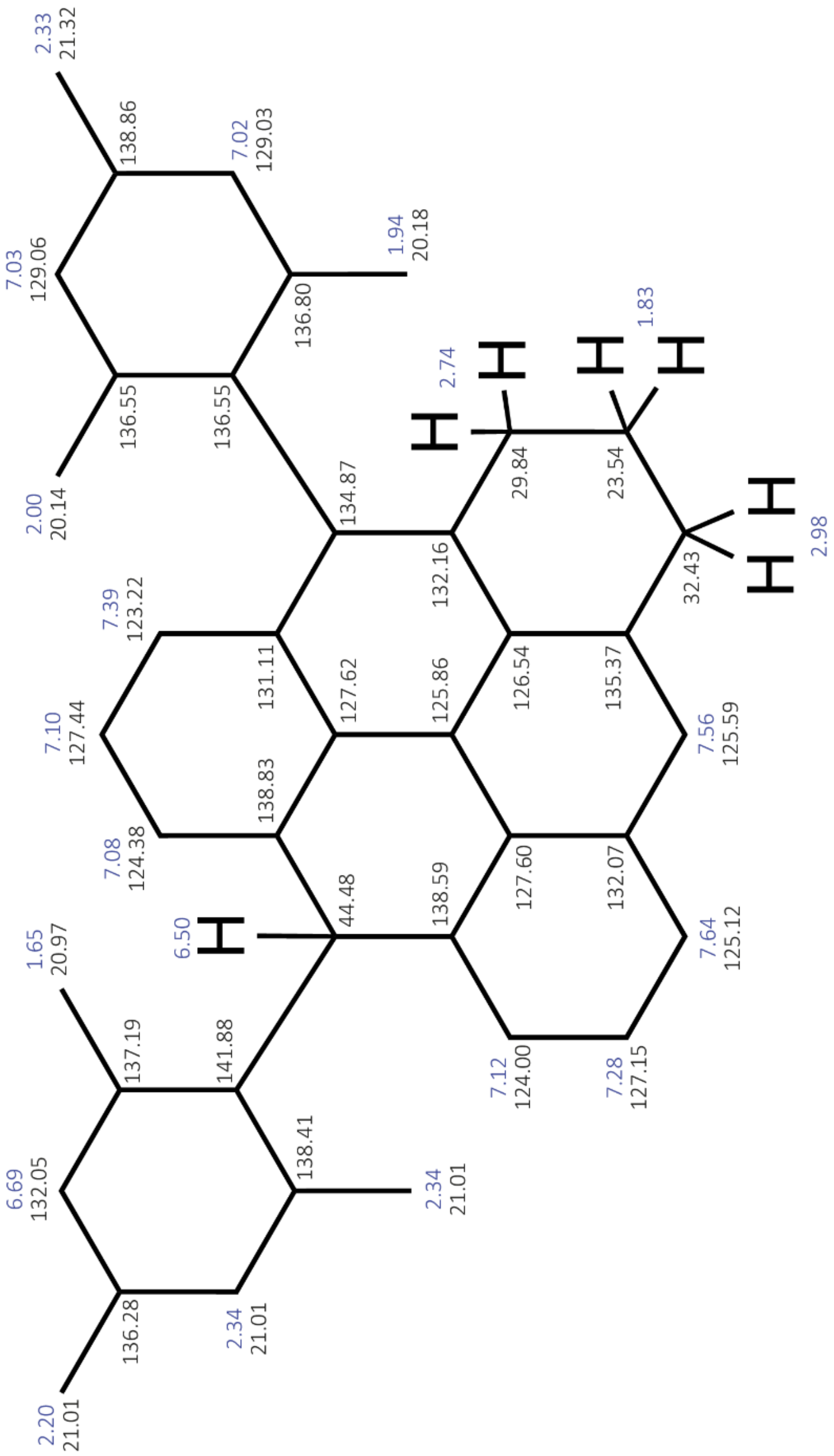

Figure S29. Assignment of ^{1}H (blue) and ^{13}C (black) NMR resonances (in ppm) of 2H-**3**. π-Bonds are omitted for clarity.

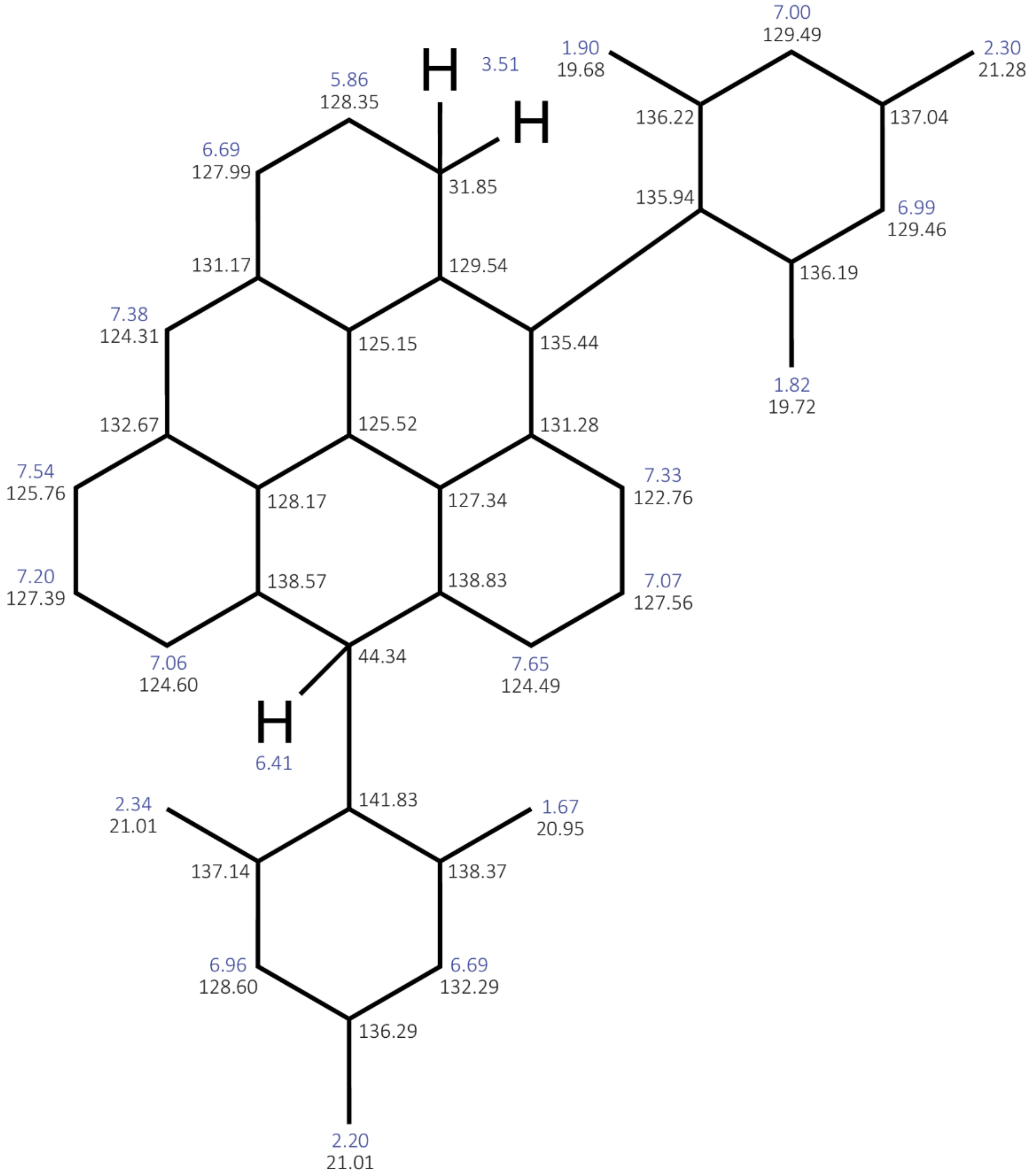

Figure S30. Assignment of ^{1}H (blue) and ^{13}C (black) NMR resonances (in ppm) of **3a**. π-Bonds are omitted for clarity.

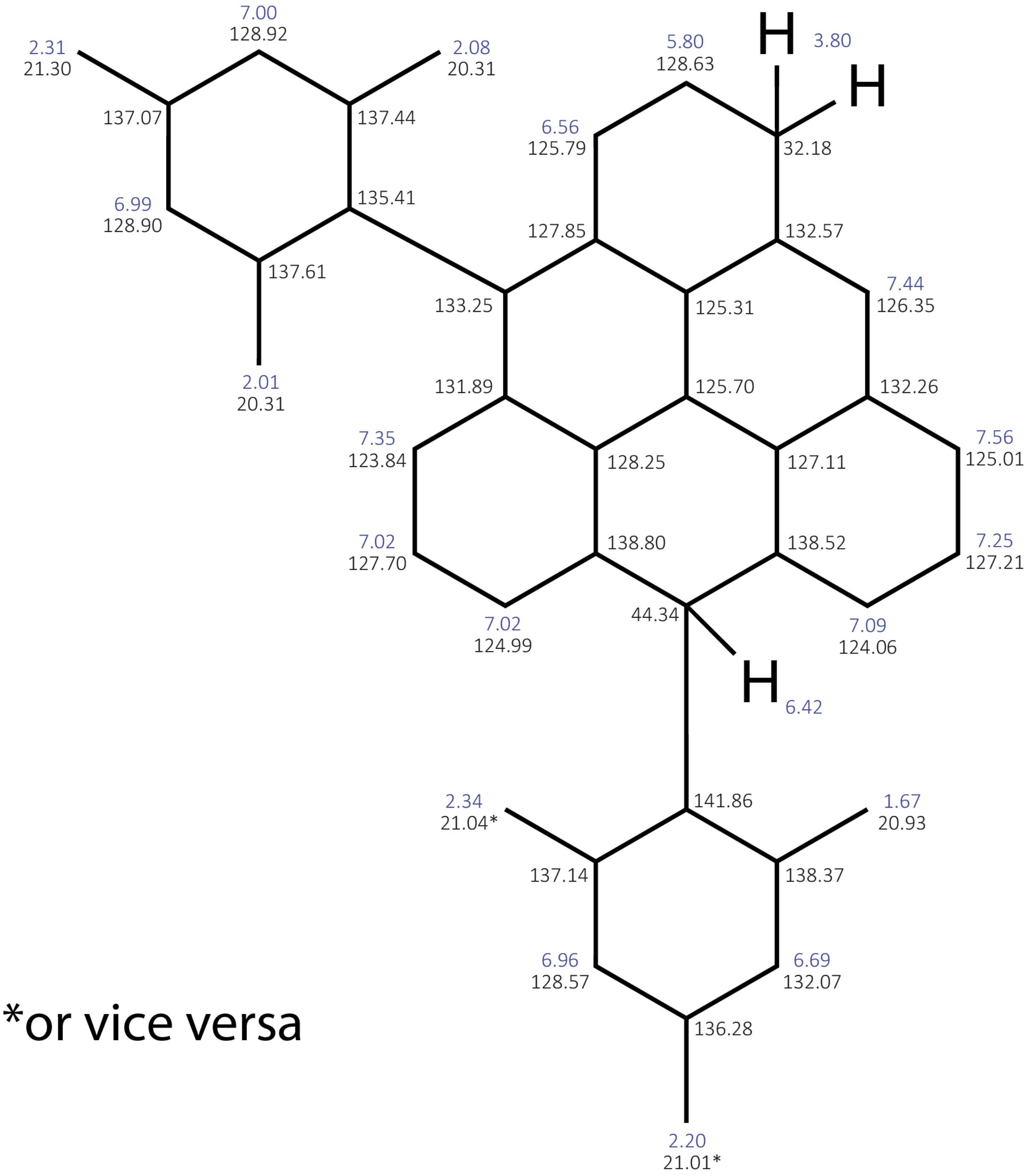

Figure S31. Assignment of ^{1}H (blue) and ^{13}C (black) NMR resonances (in ppm) of **3b**. π-Bonds are omitted for clarity.

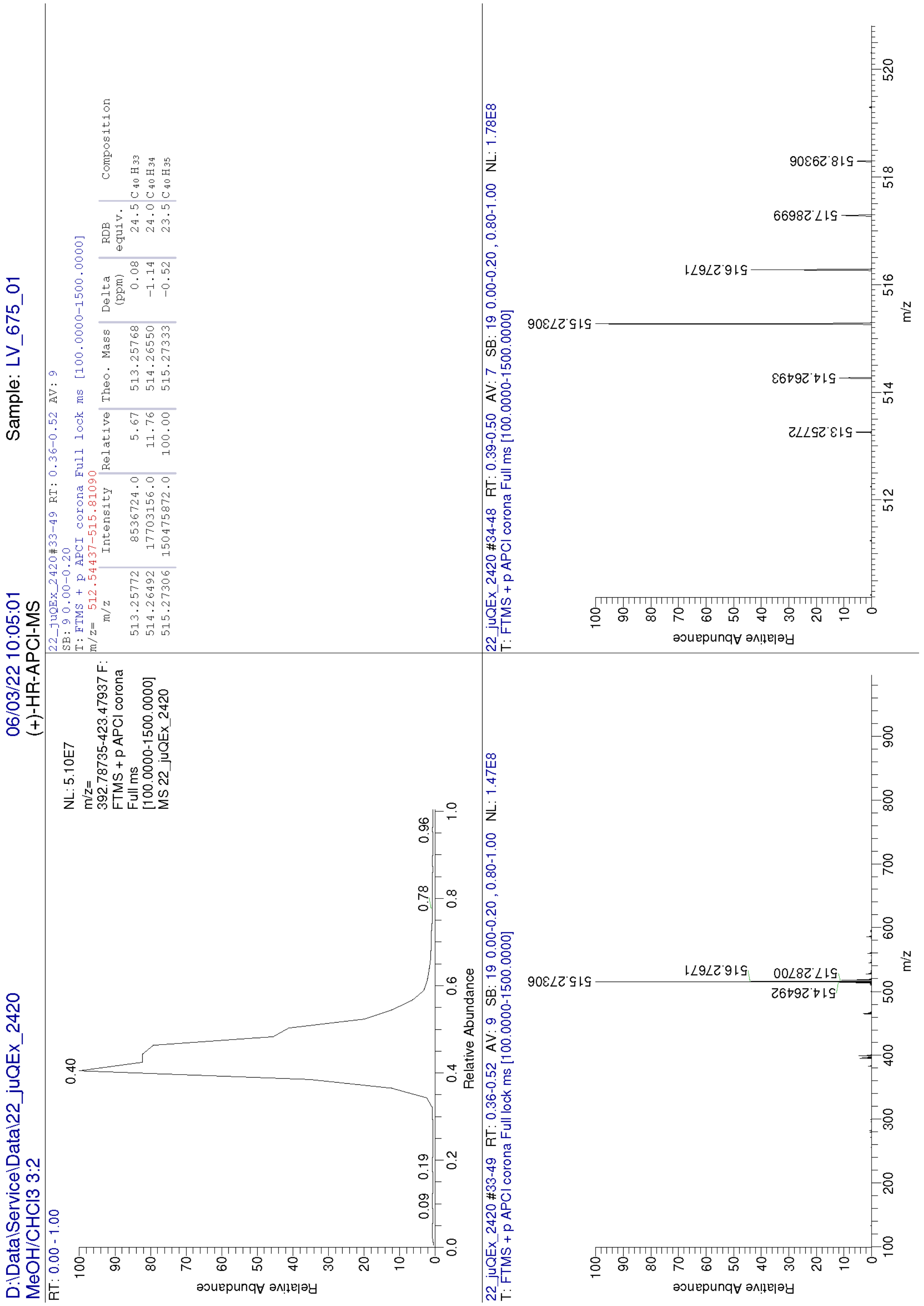

Figure S32. HRMS (APCI) of compound **3-Mes**.

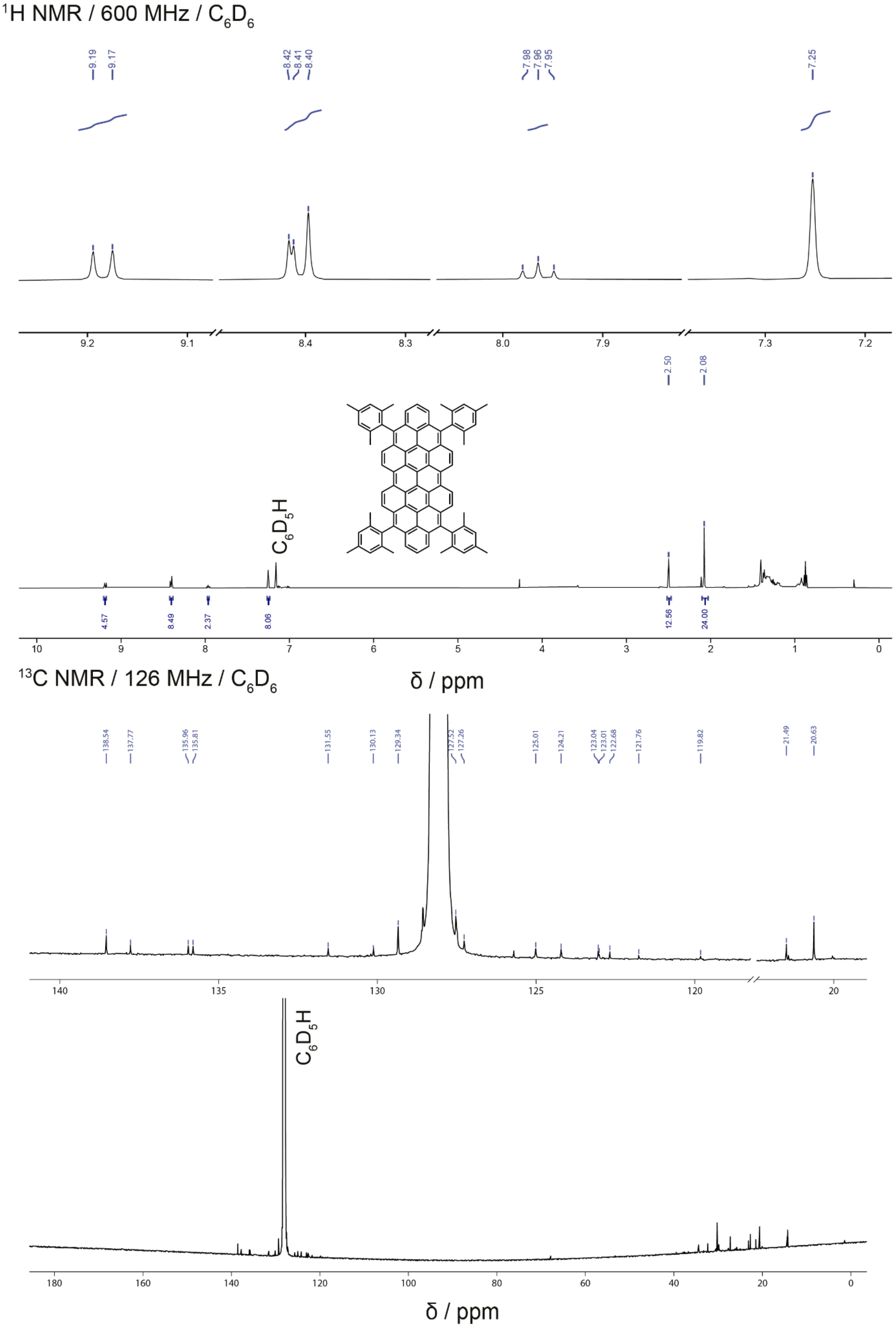

Figure S33. ^{1}H NMR (top) and ^{13}C NMR (bottom) of **1-Mes** in C_6D_6.

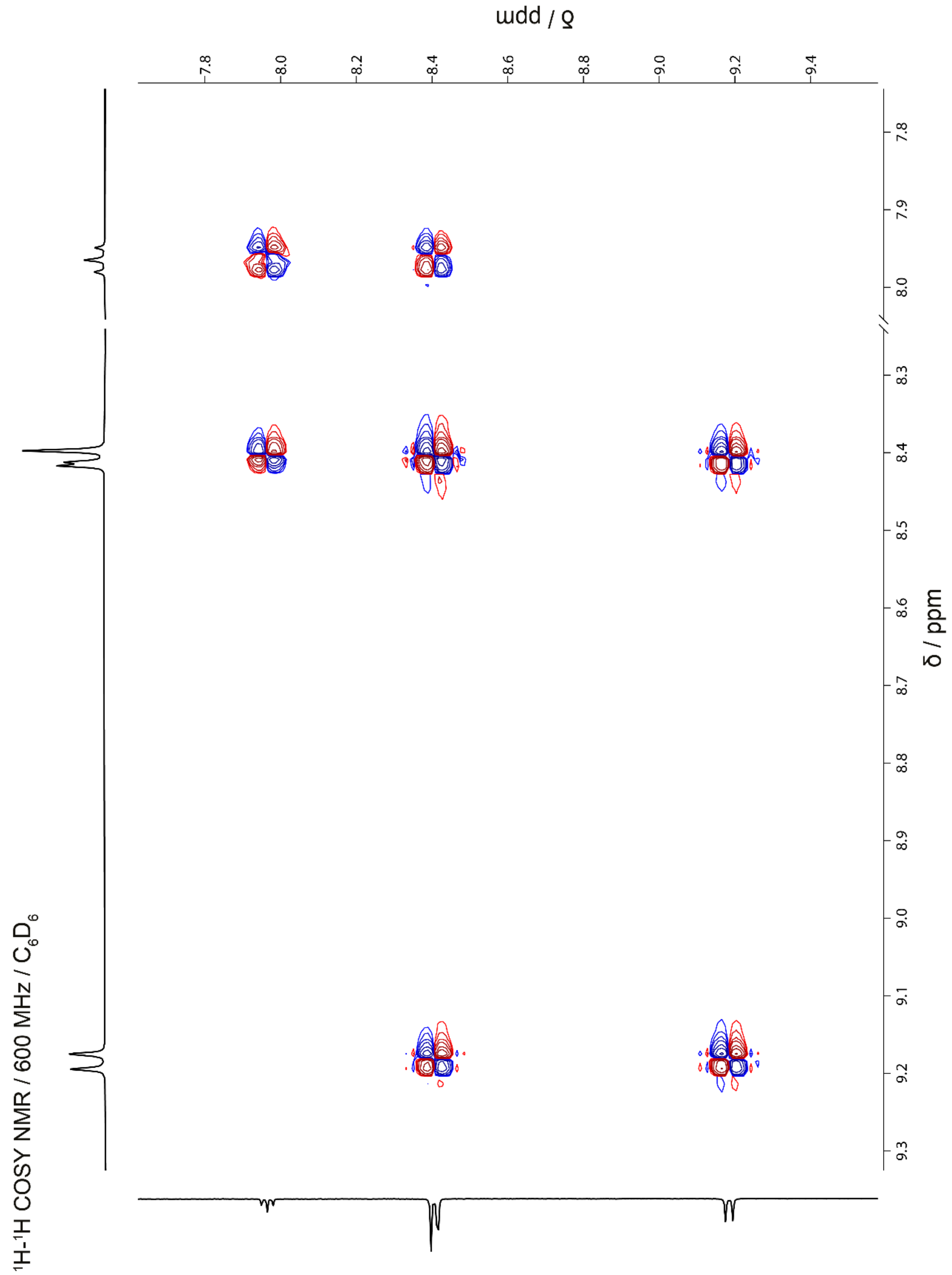

Figure S34. ^{1}H–^{1}H COSY NMR of **1-Mes** in C_6D_6.

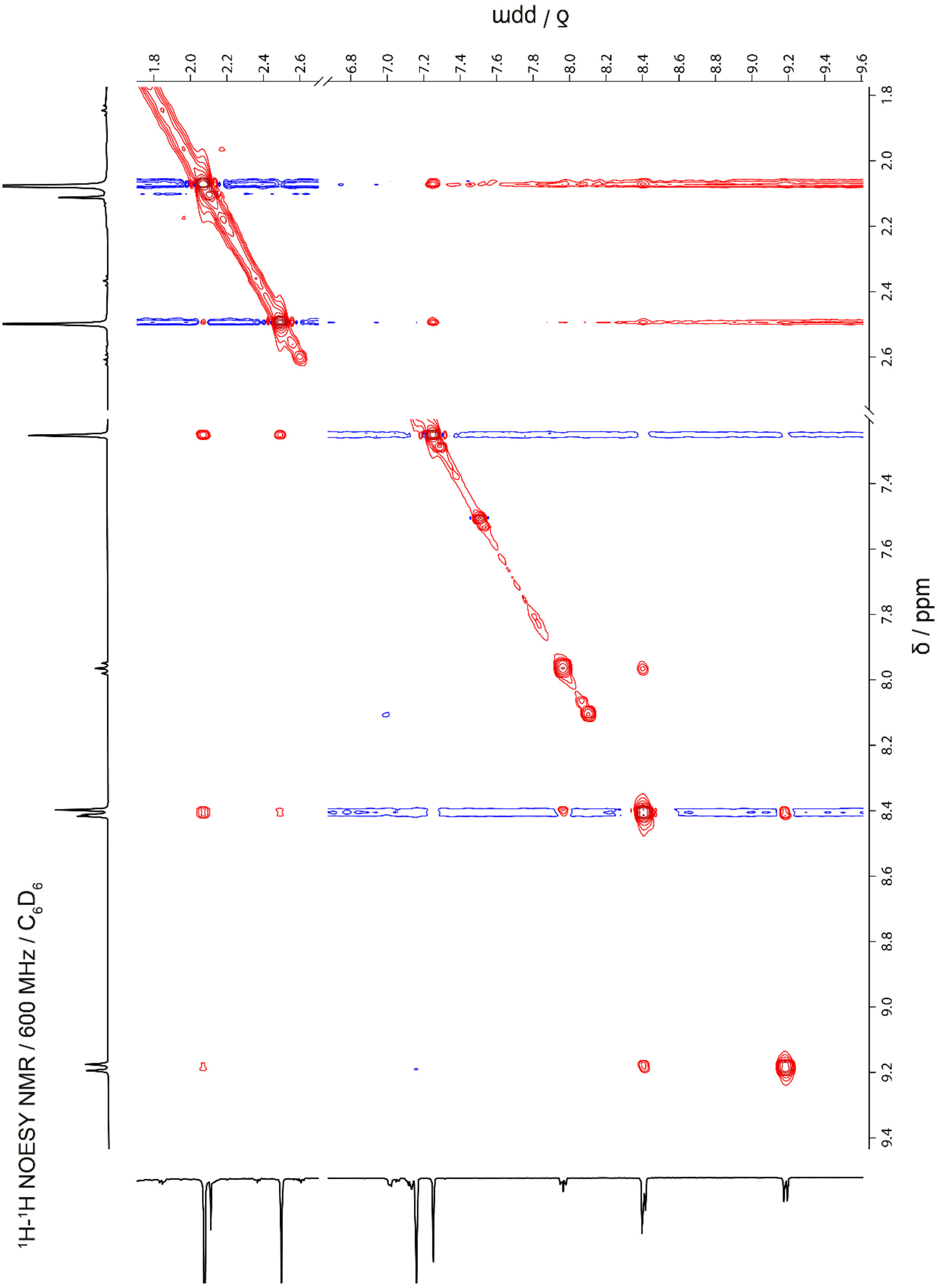

Figure S35. ^1H–^1H NOESY NMR of **1-Mes** in C_6D_6.

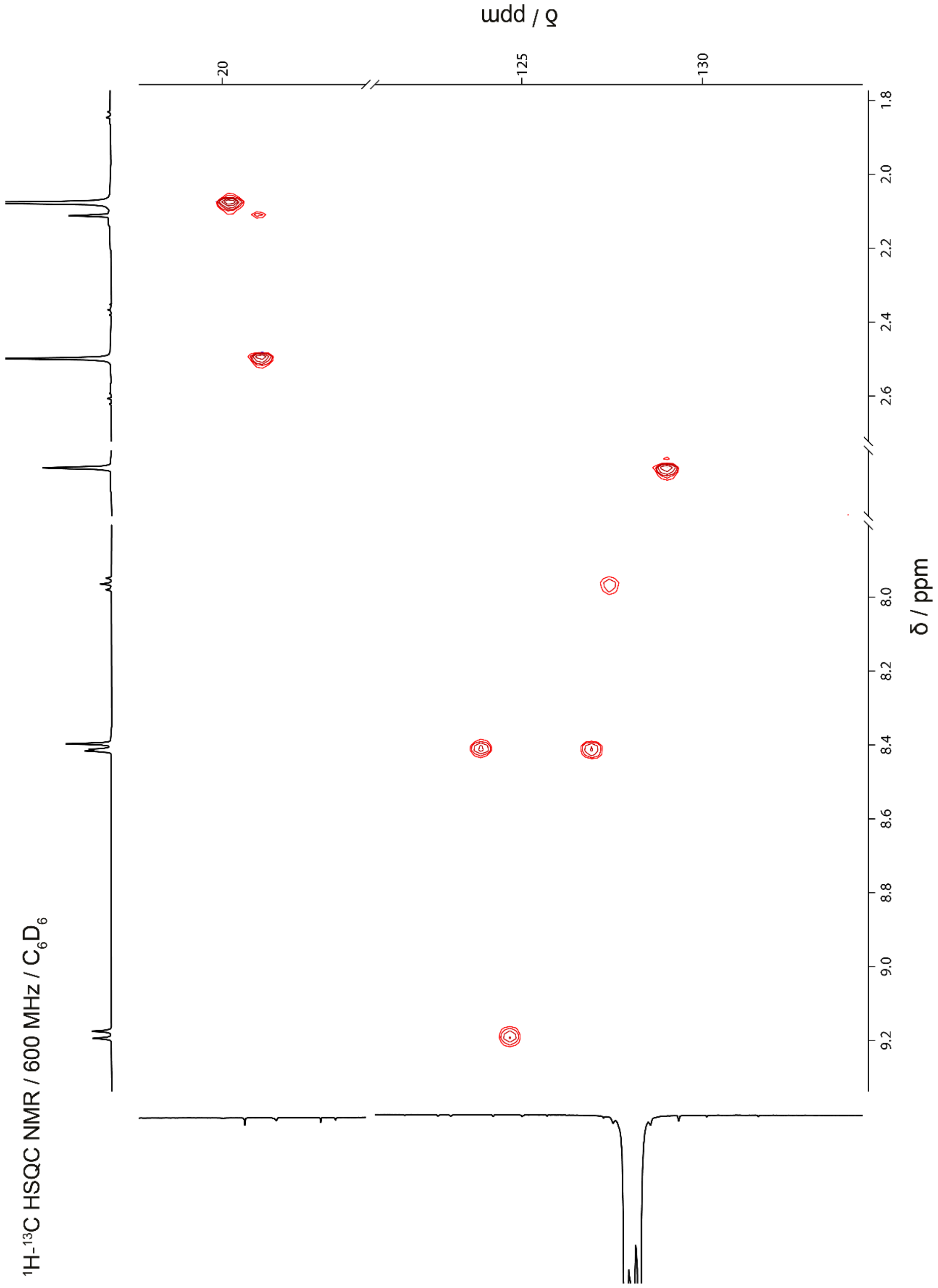

Figure S36. $^{1}H–^{13}C$ HSQC NMR of **1-Mes** in C_6D_6.

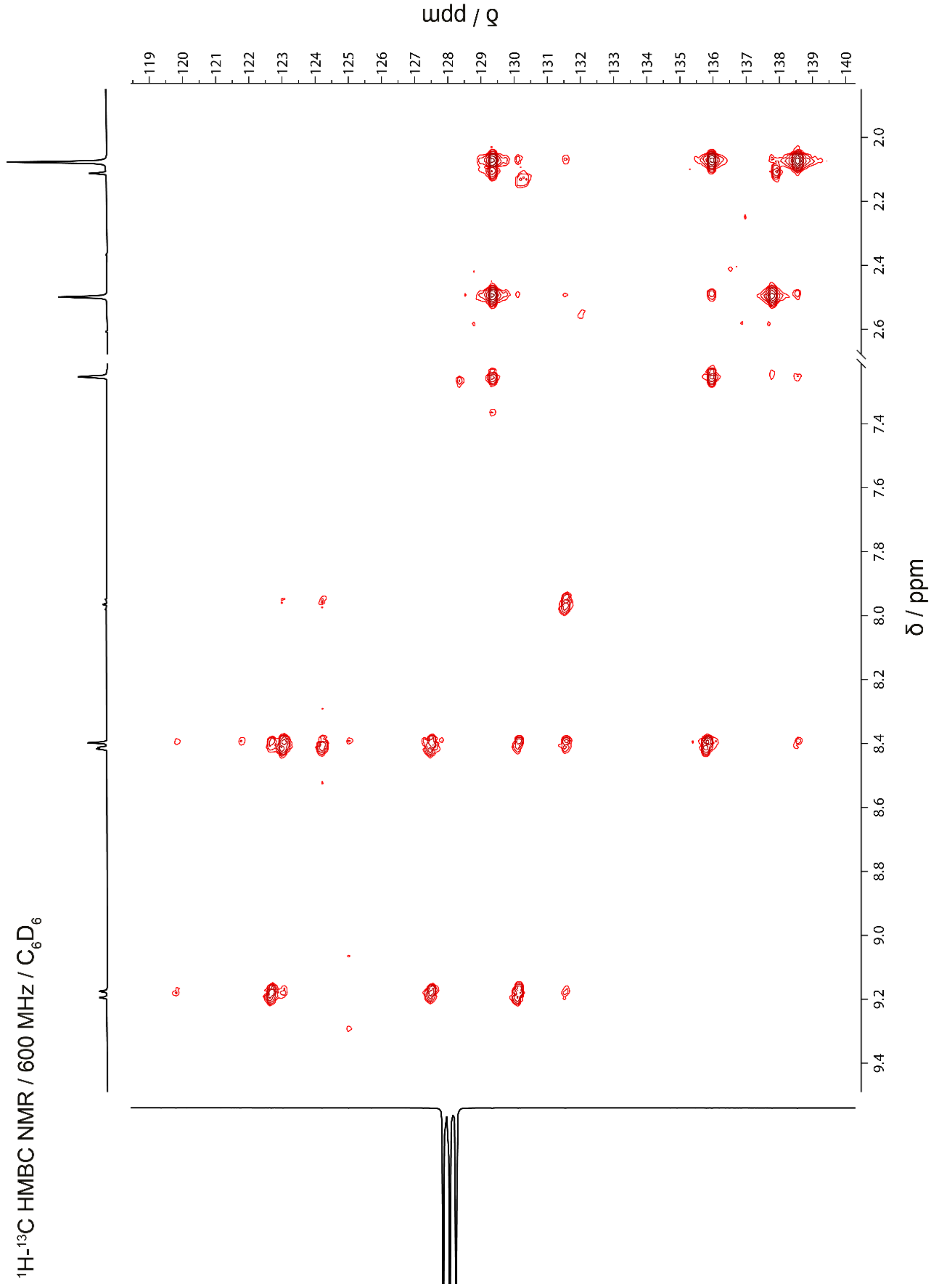

Figure S37. ^{1}H–^{13}C HMBC NMR of **1-Mes** in C_6D_6.

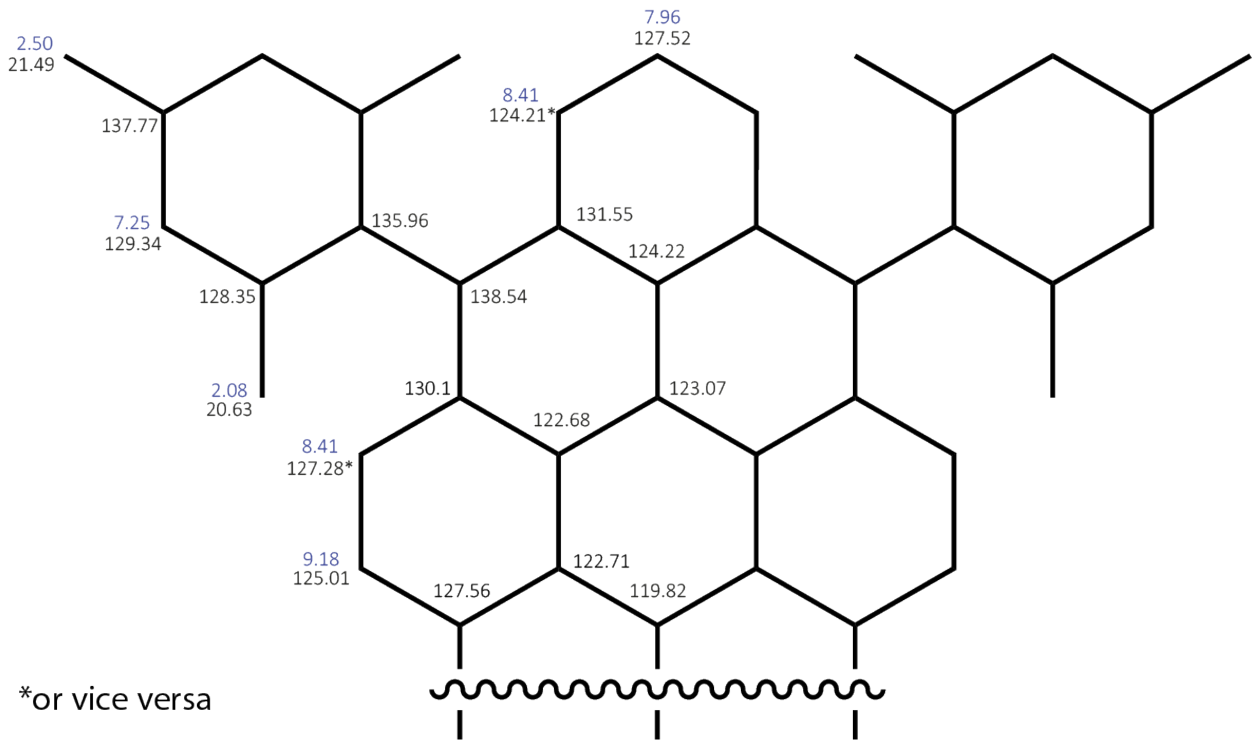

Figure S38. Assignment of ^{1}H (blue) and ^{13}C (black) NMR resonances (in ppm) of **1-Mes**. π-Bonds are omitted for clarity.

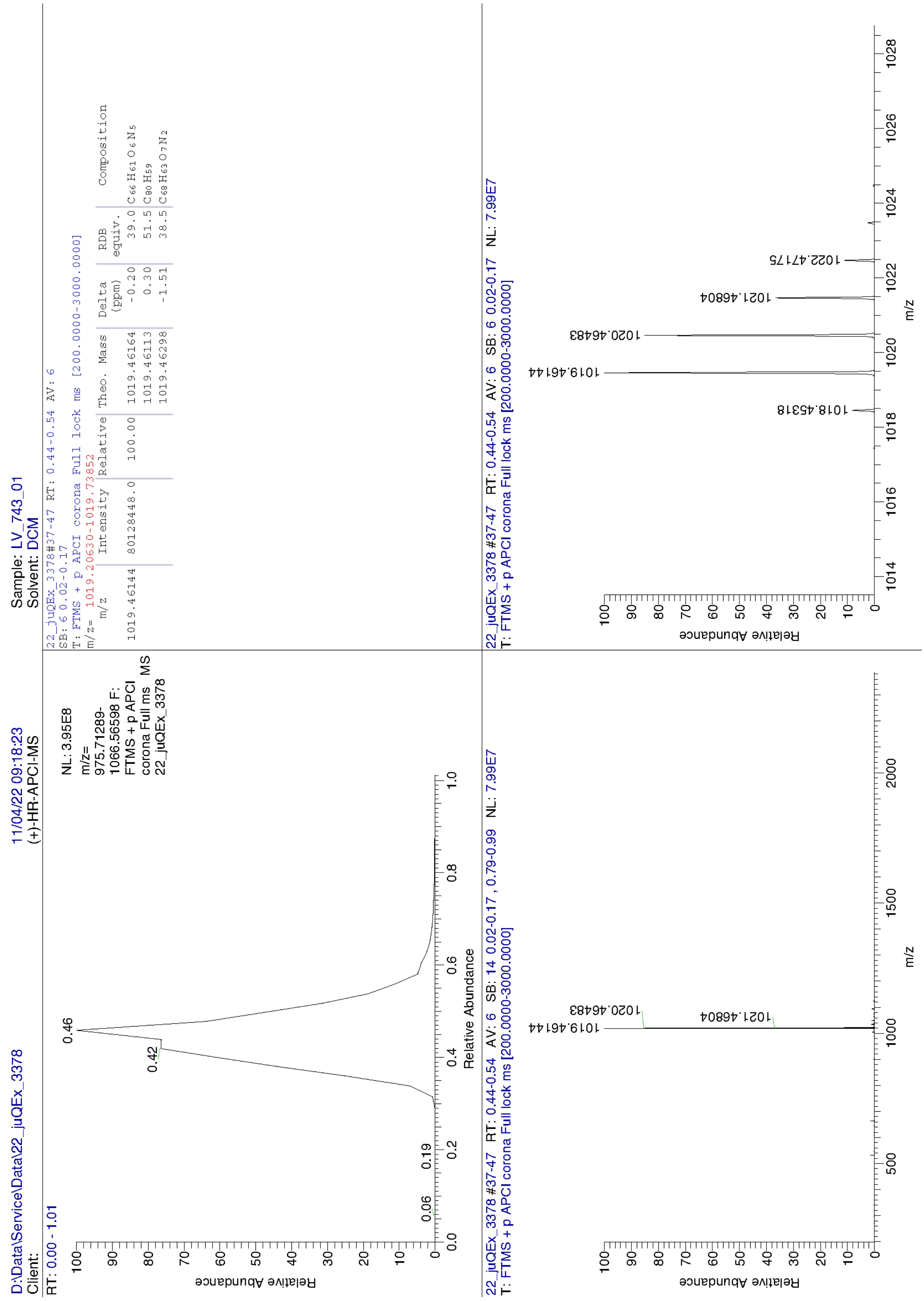

Figure S39. HRMS (APCI) of **1-Mes**.

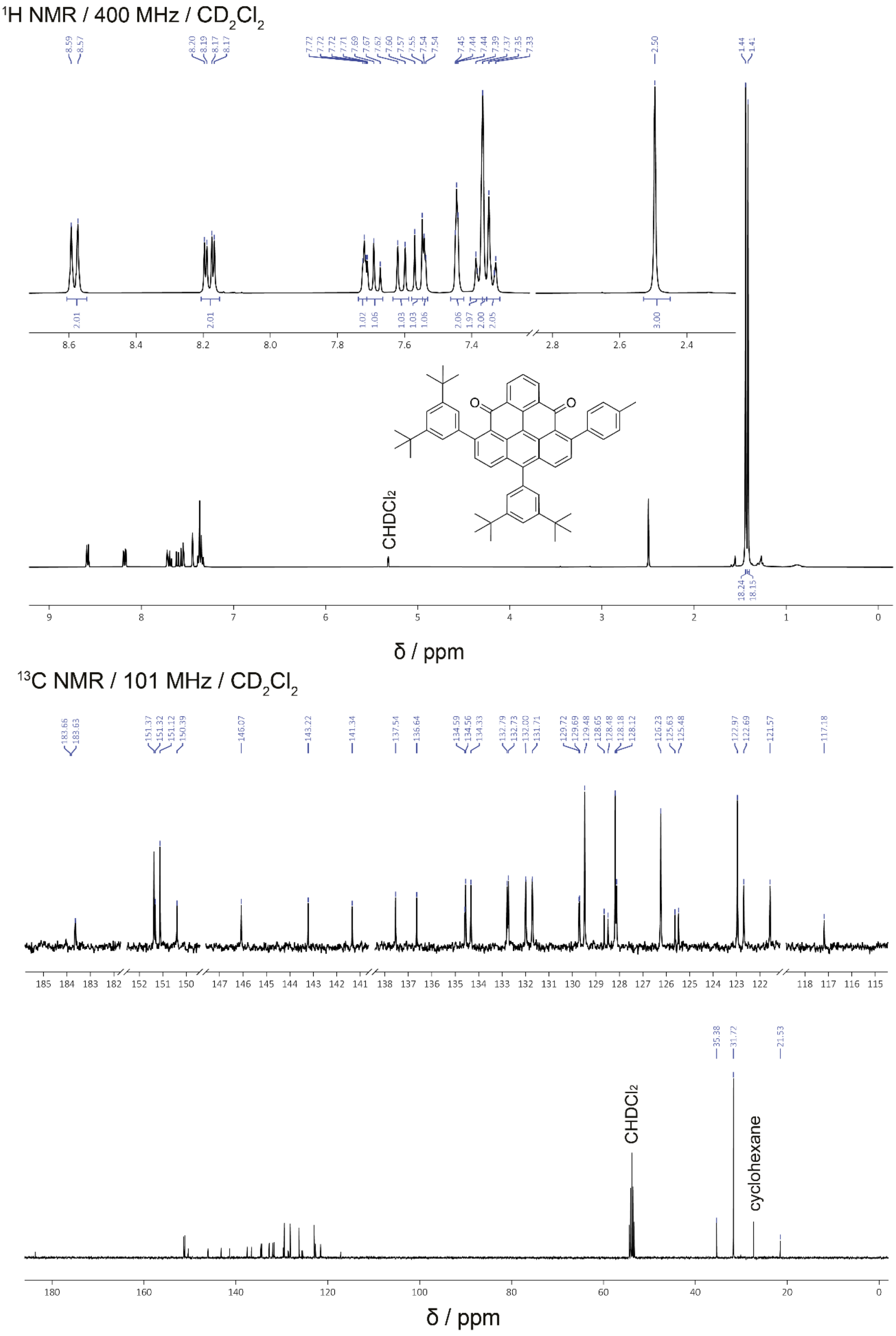

Figure S40. ^{1}H NMR (top) and ^{13}C NMR (bottom) of **18-Tol** in CD_2Cl_2.

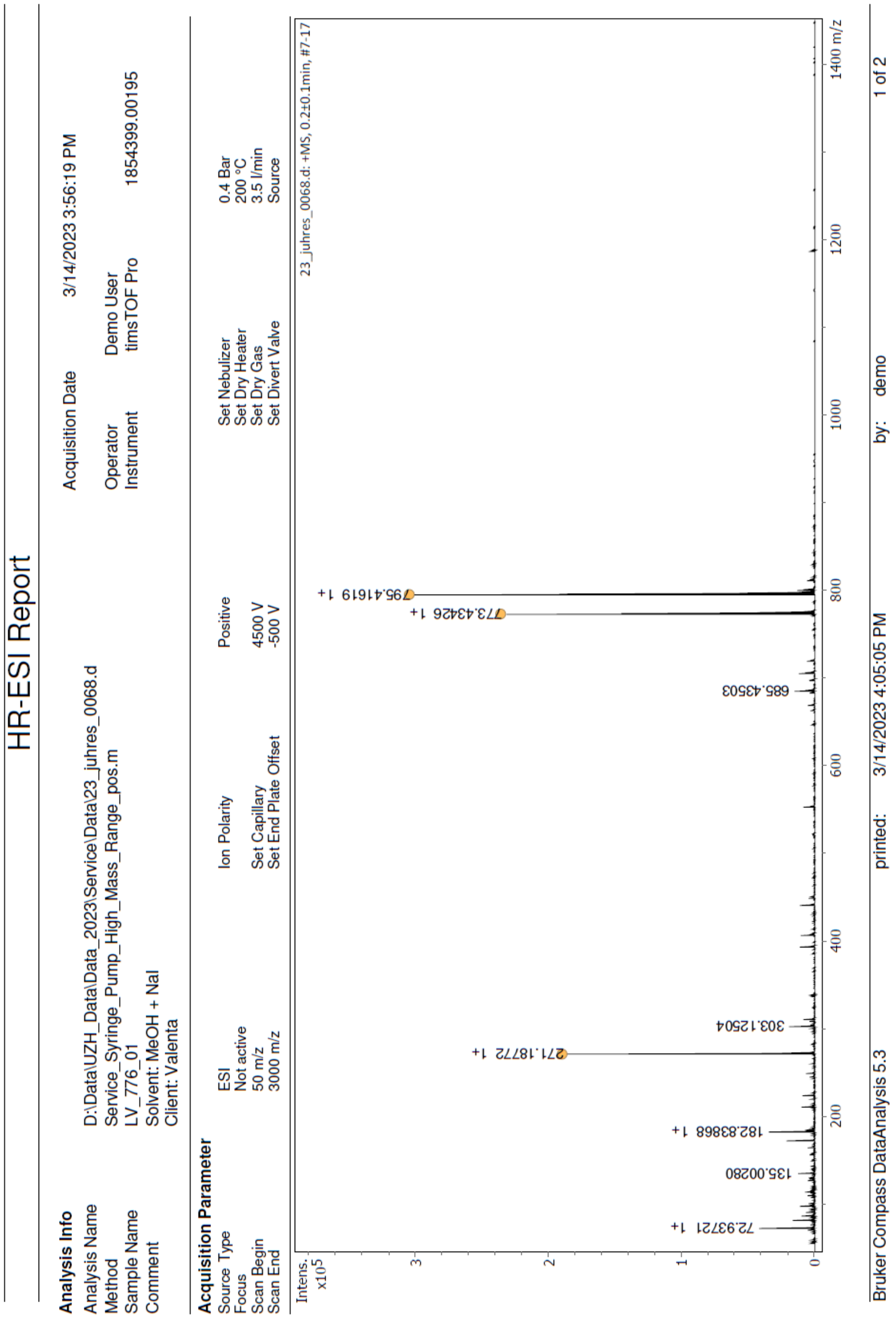

Figure S41. HRMS (ESI) of **18-Tol**.

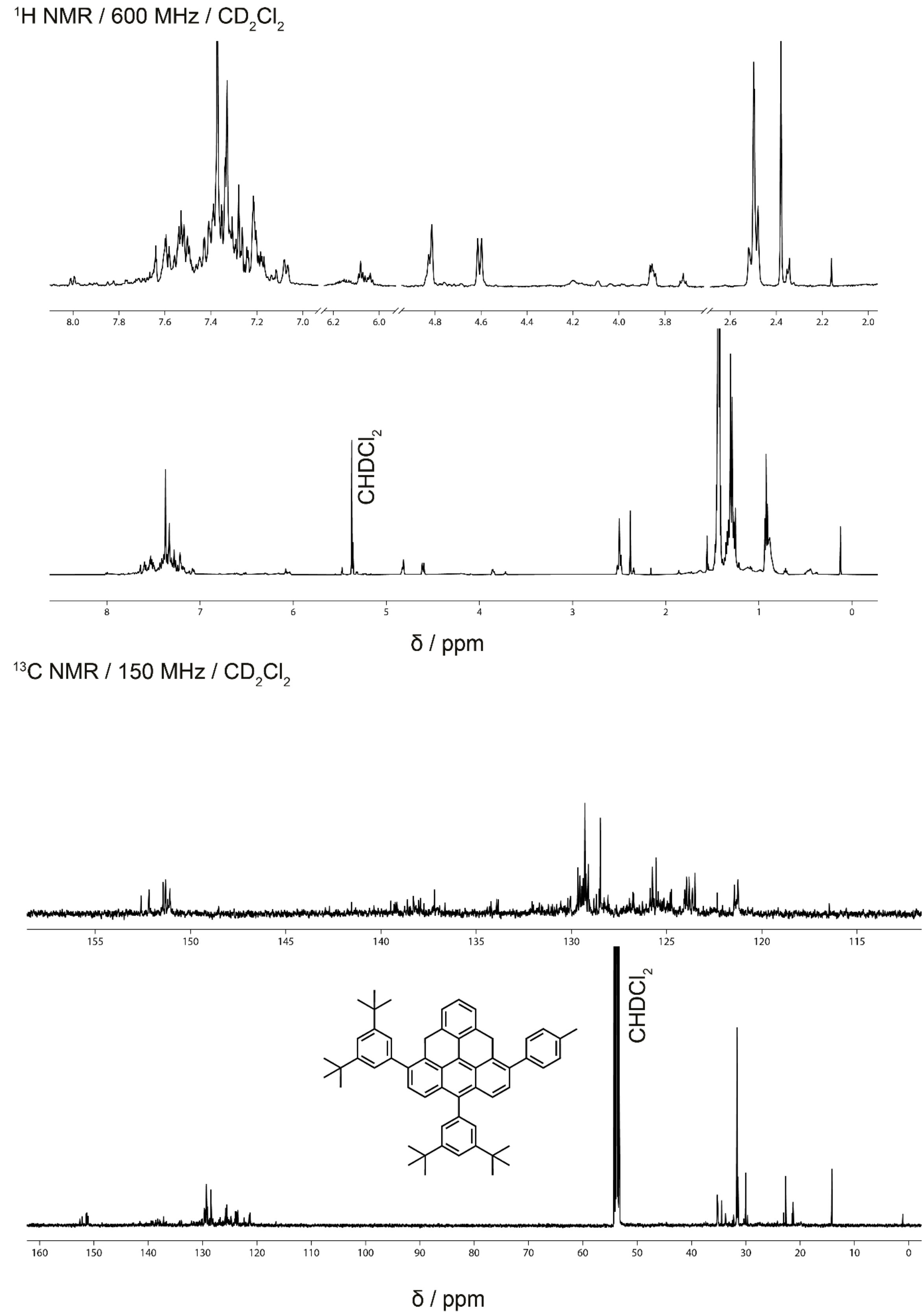

Figure S42. ^{1}H NMR (top) and ^{13}C NMR (bottom) of **4-Tol** CD_2Cl_2.

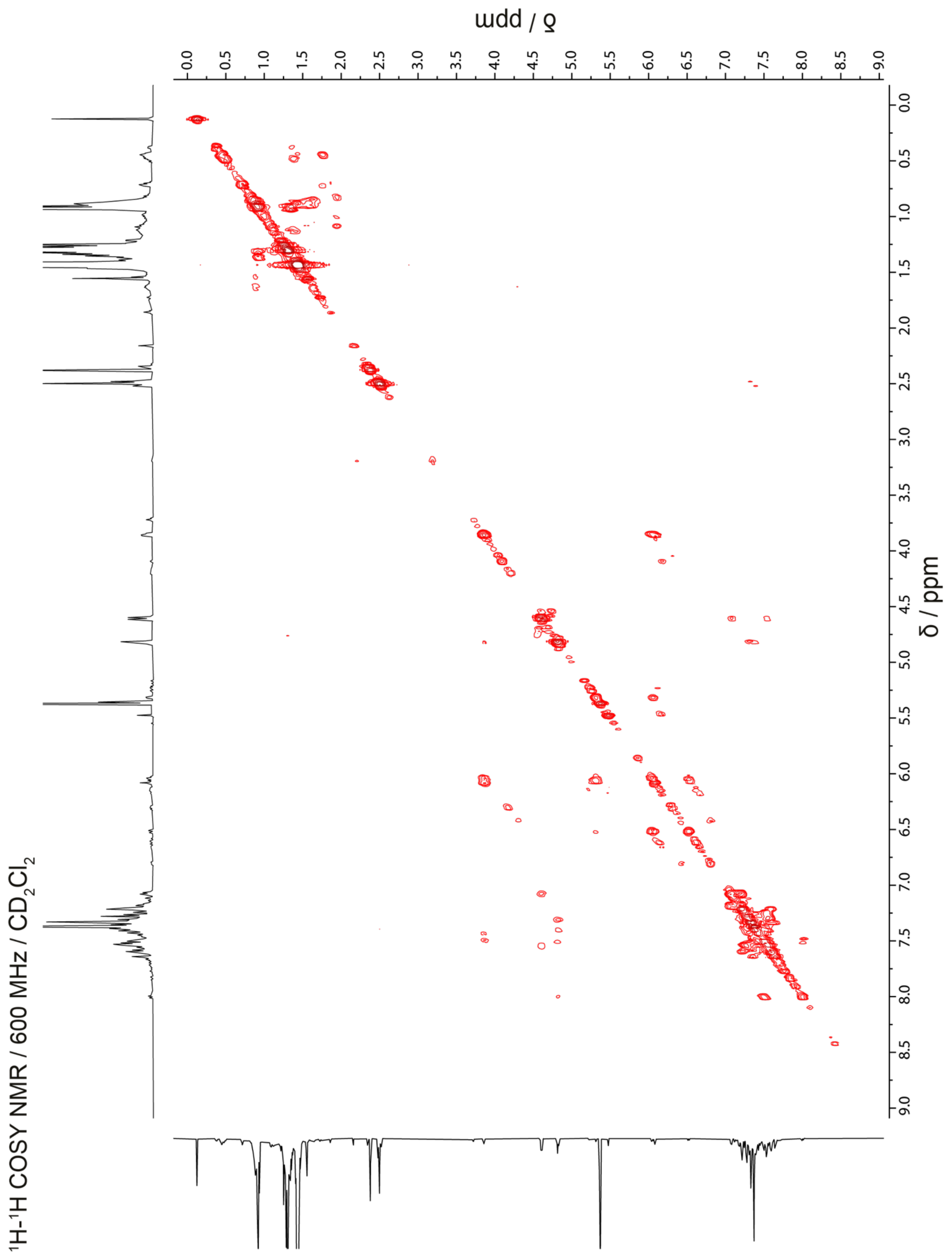

Figure S43. ^{1}H–^{1}H COSY NMR of **4-Tol** in CD_2Cl_2.

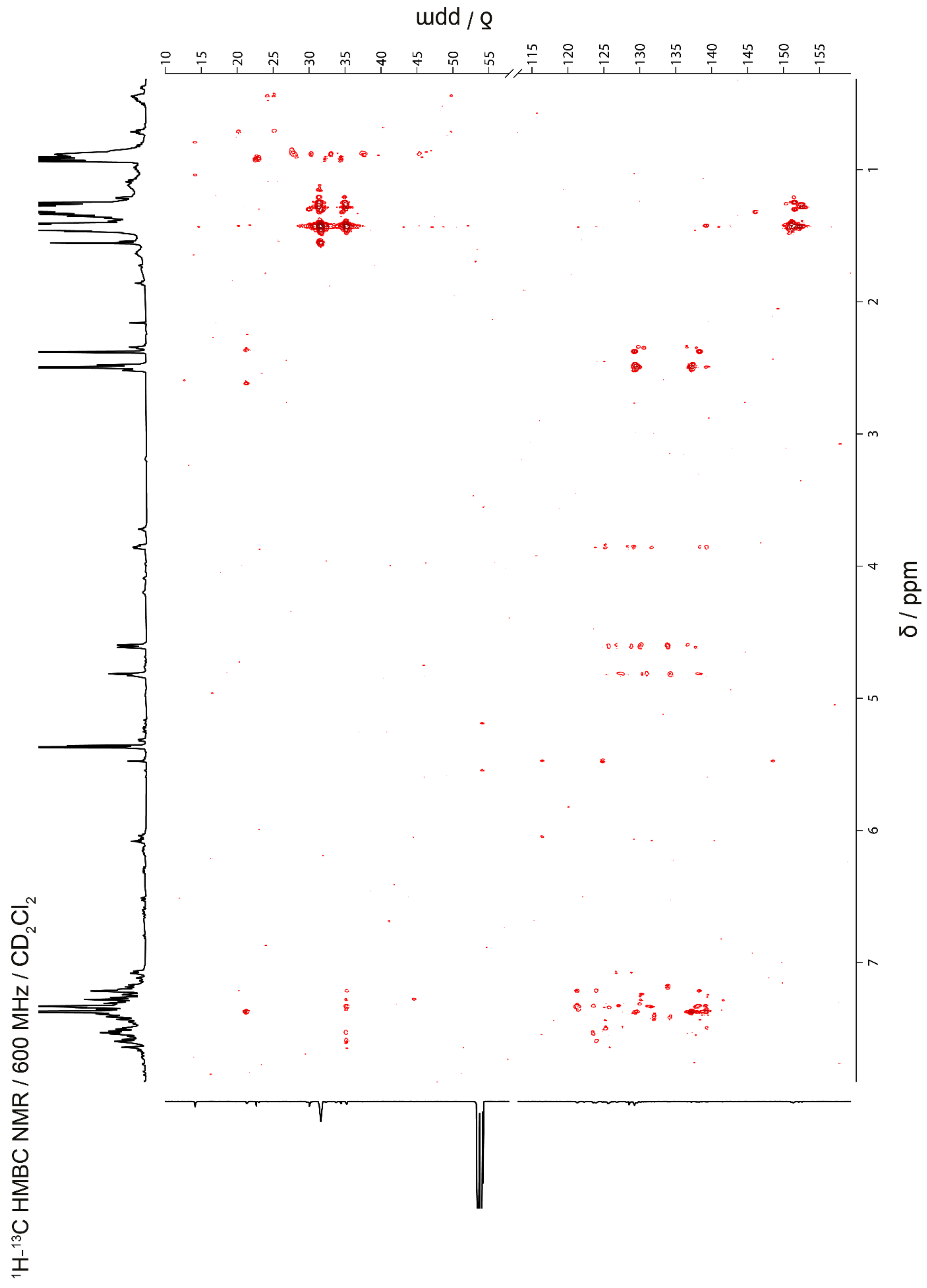

Figure S44. ^{1}H–^{13}C HMBC NMR of **4-Tol** in CD_2Cl_2.

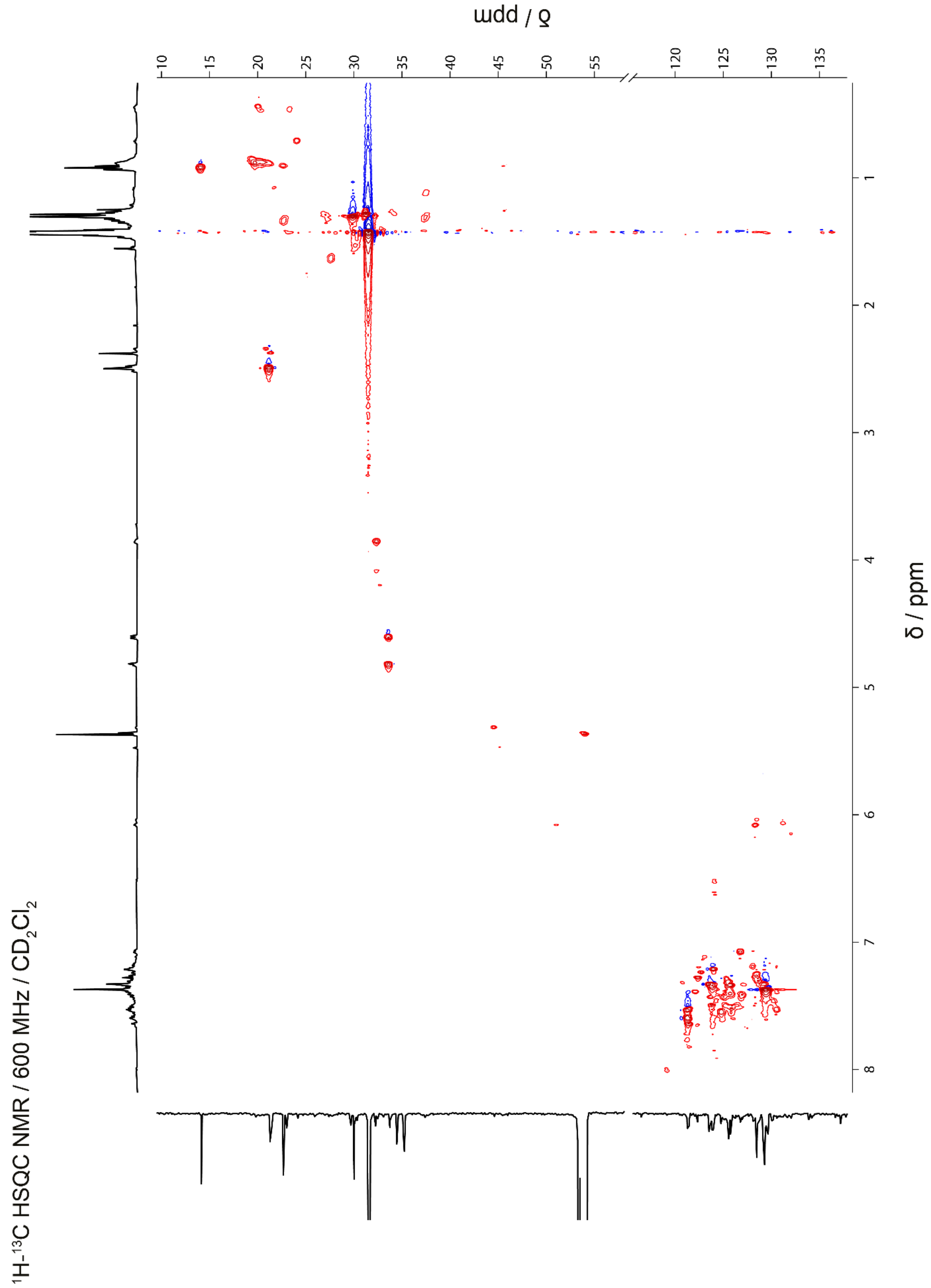

Figure S45. ^{1}H–^{13}C HSQC NMR of **4-Tol** in CD_2Cl_2.

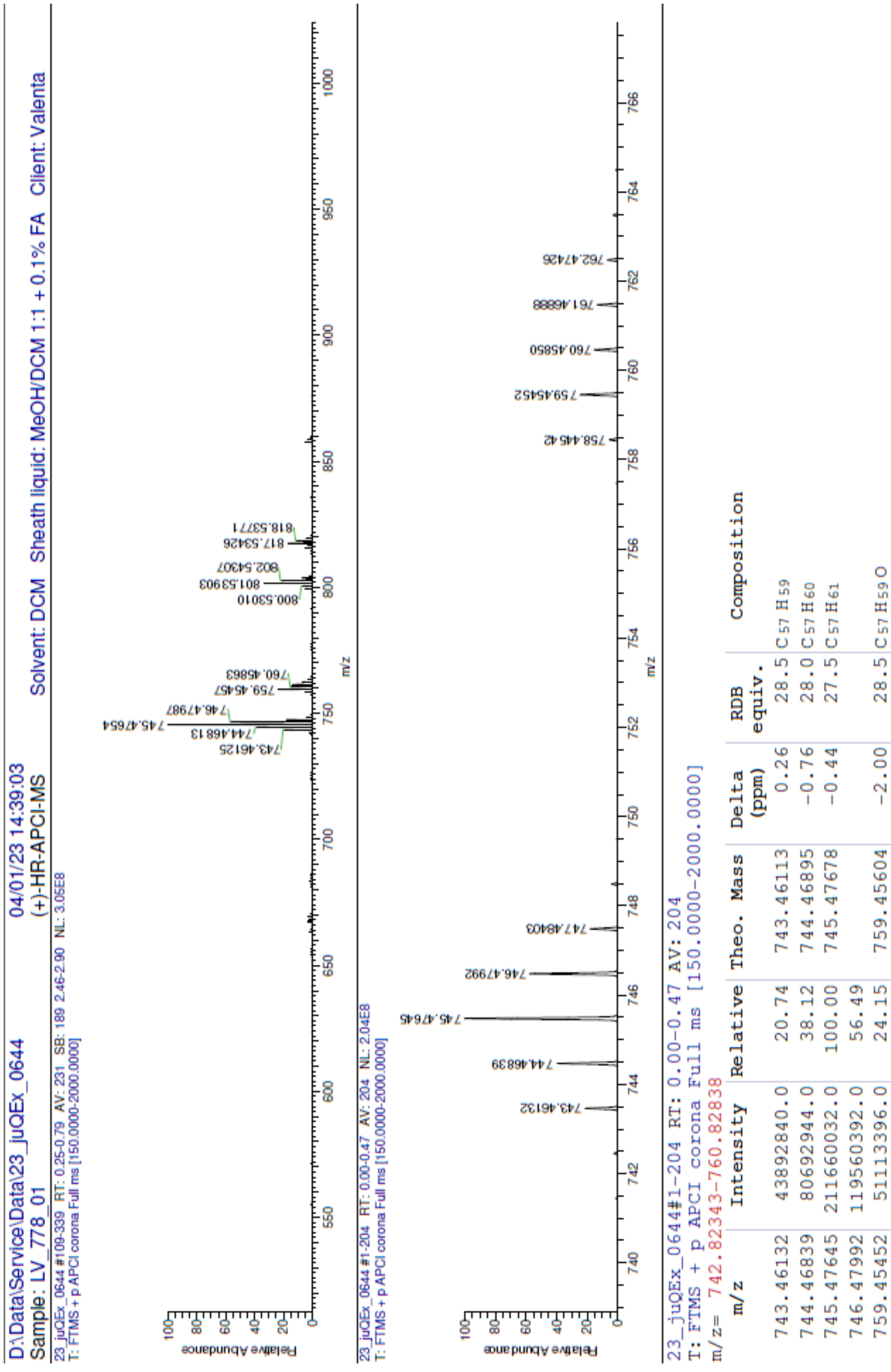

23_juQEx_0644#1-204 RT: 0.00-0.47 AV: 204
T: FTMS + p APCI corona Full ms [150.0000-2000.0000]
m/z= 742.82343-760.82838

m/z	Intensity	Relative	Theo. Mass	Delta (ppm)	RDB equiv.	Composition
743.46132	43892840.0	20.74	743.46113	0.26	28.5	$C_{57}H_{59}$
744.46839	80692944.0	38.12	744.46895	-0.76	28.0	$C_{57}H_{60}$
745.47645	211660032.0	100.00	745.47678	-0.44	27.5	$C_{57}H_{61}$
746.47992	119560392.0	56.49				
759.45452	51113396.0	24.15	759.45604	-2.00	28.5	$C_{57}H_{59}O$

Figure S46. HRMS (APCI) of **4-Tol**.

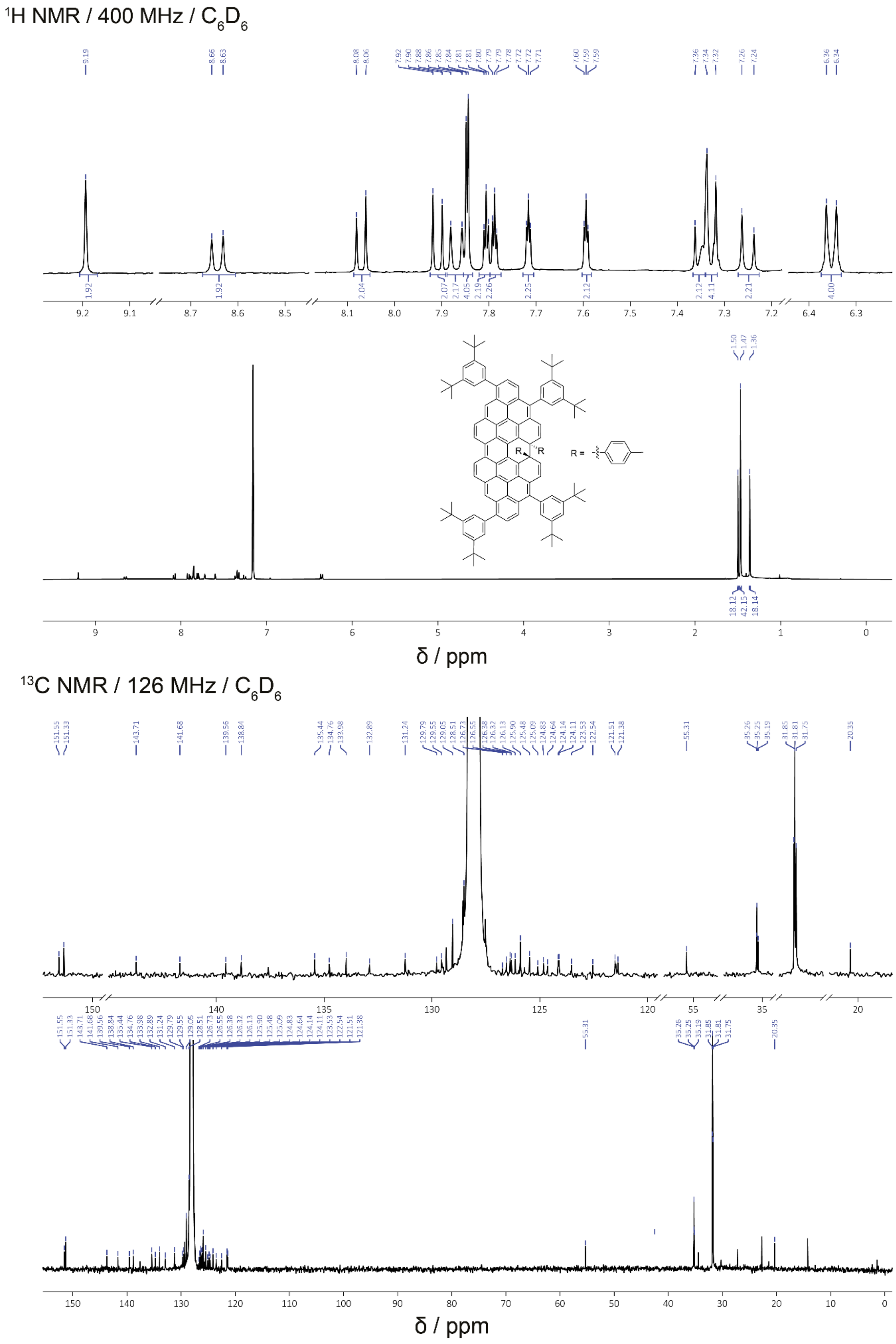

Figure S47. ^{1}H NMR (top) and ^{13}C NMR (bottom) of **5-Tol** in C_6D_6.

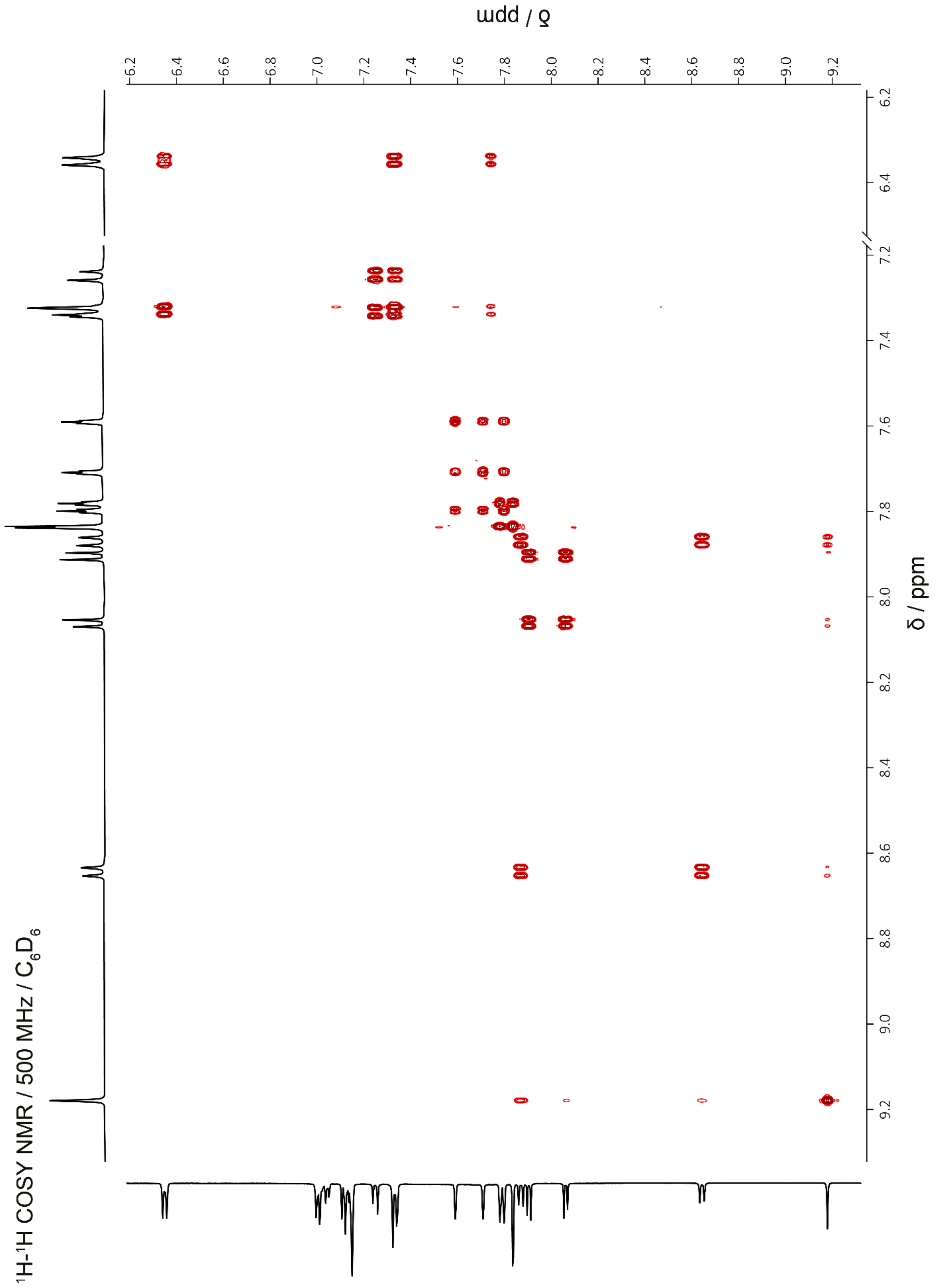

Figure S48. ^{1}H–^{1}H COSY NMR of **5-Tol** in C_6D_6.

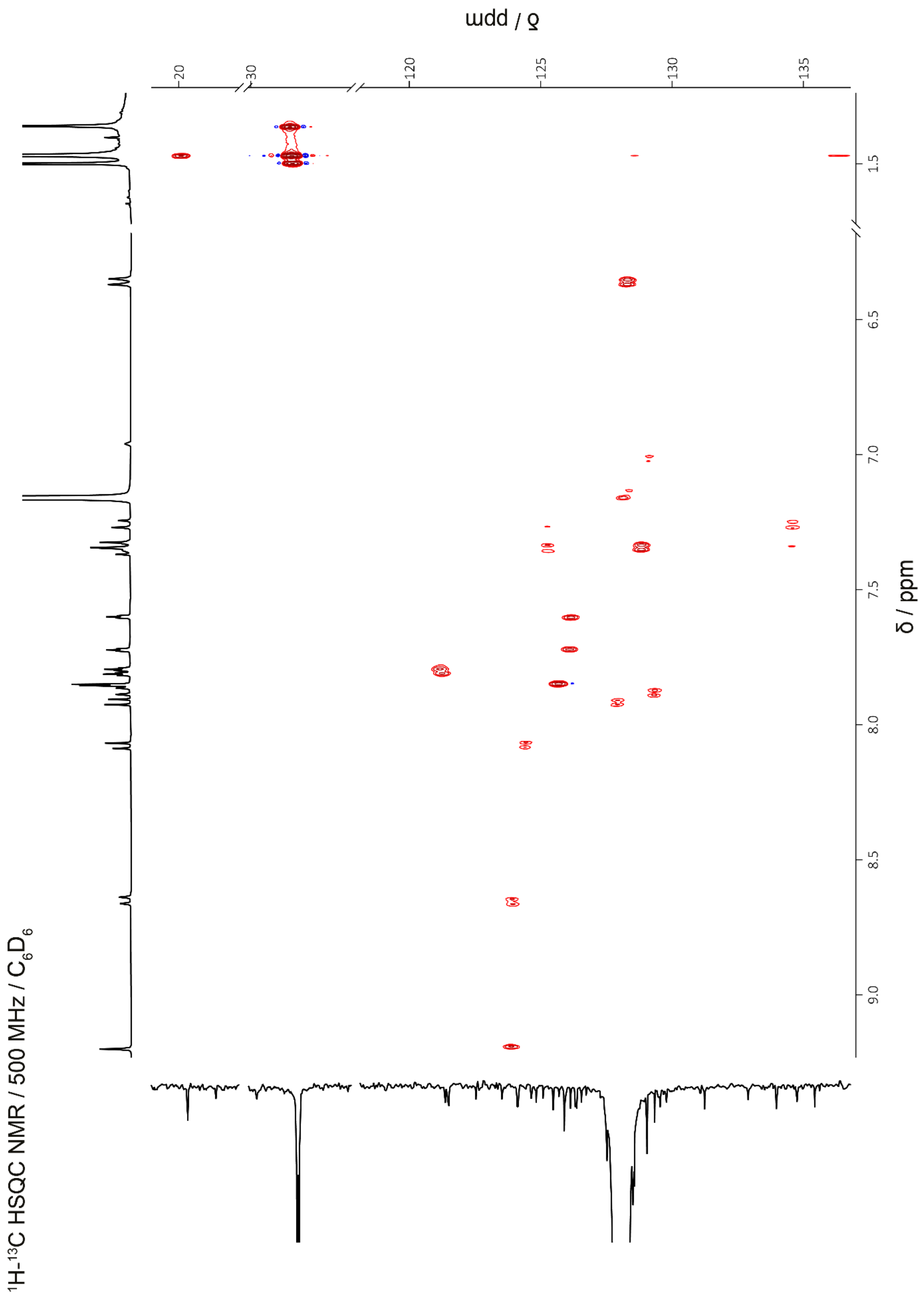

Figure S49. ^{1}H–^{13}C HSQC NMR of **5-Tol** in C_6D_6.

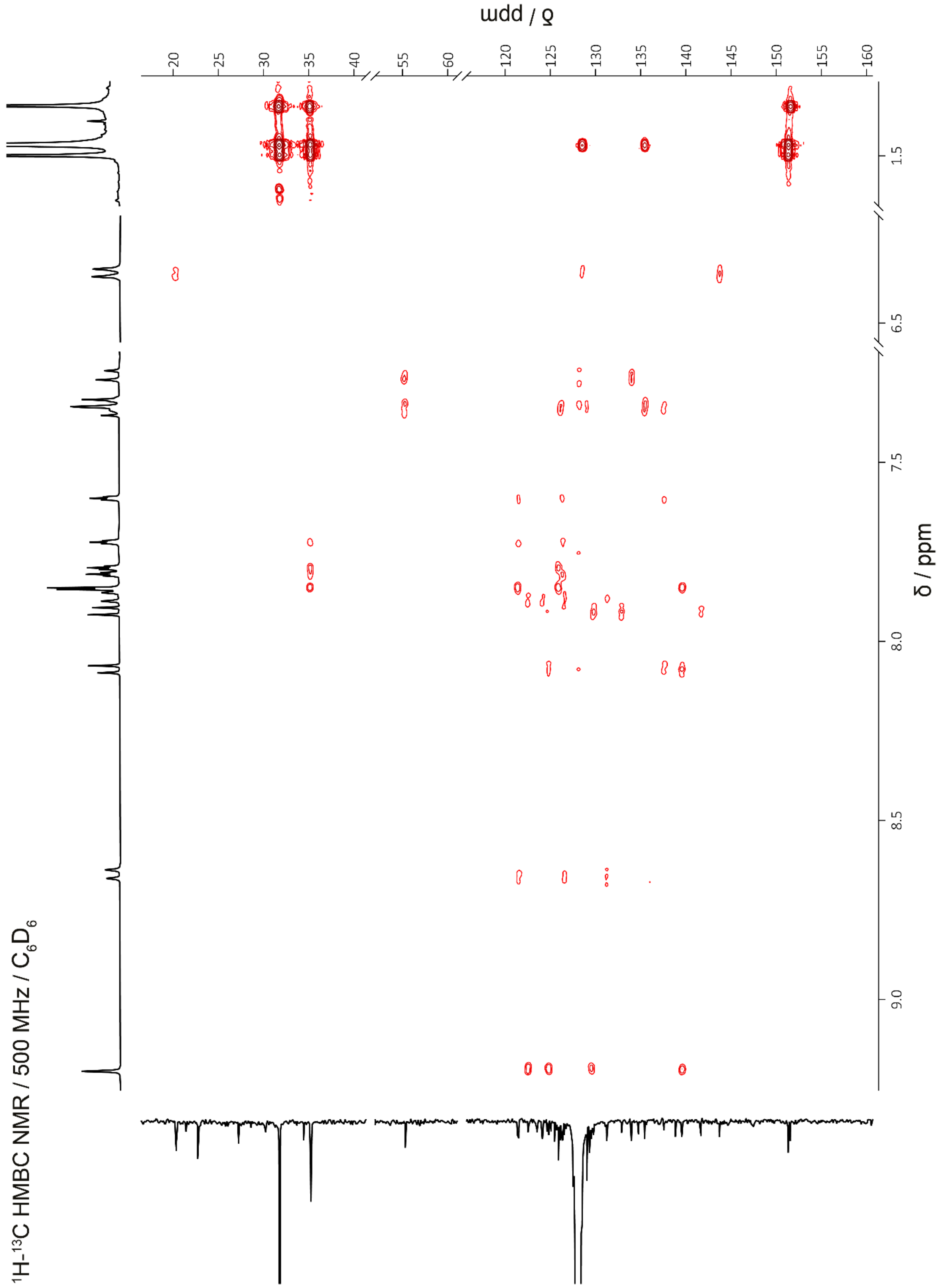

Figure S50. ^{1}H–^{13}C HMBC NMR of **5-Tol** in C_6D_6.

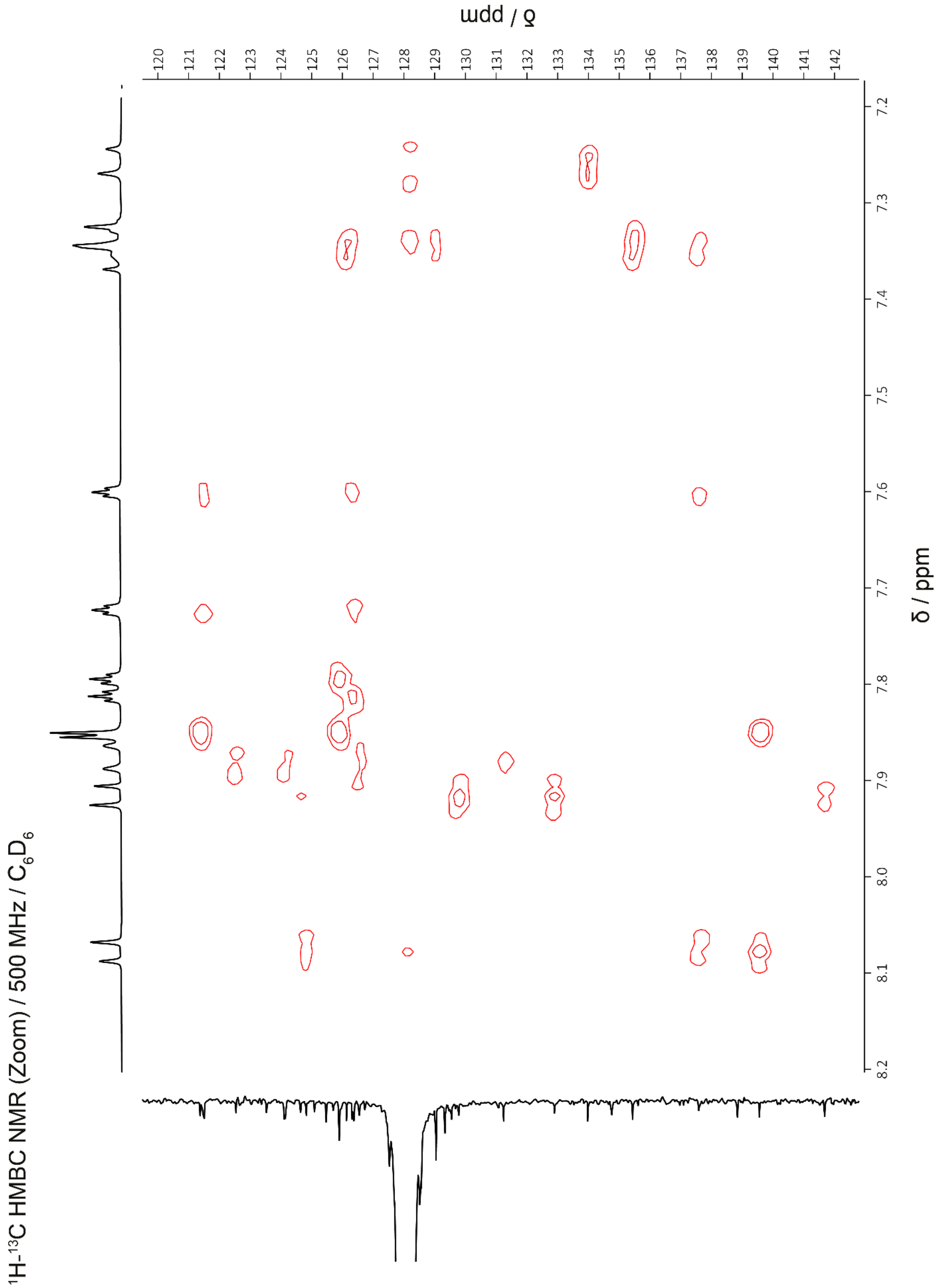

Figure S51. ^{1}H–^{13}C HSQC NMR (Zoom aromatic region) of **5-Tol** in C_6D_6.

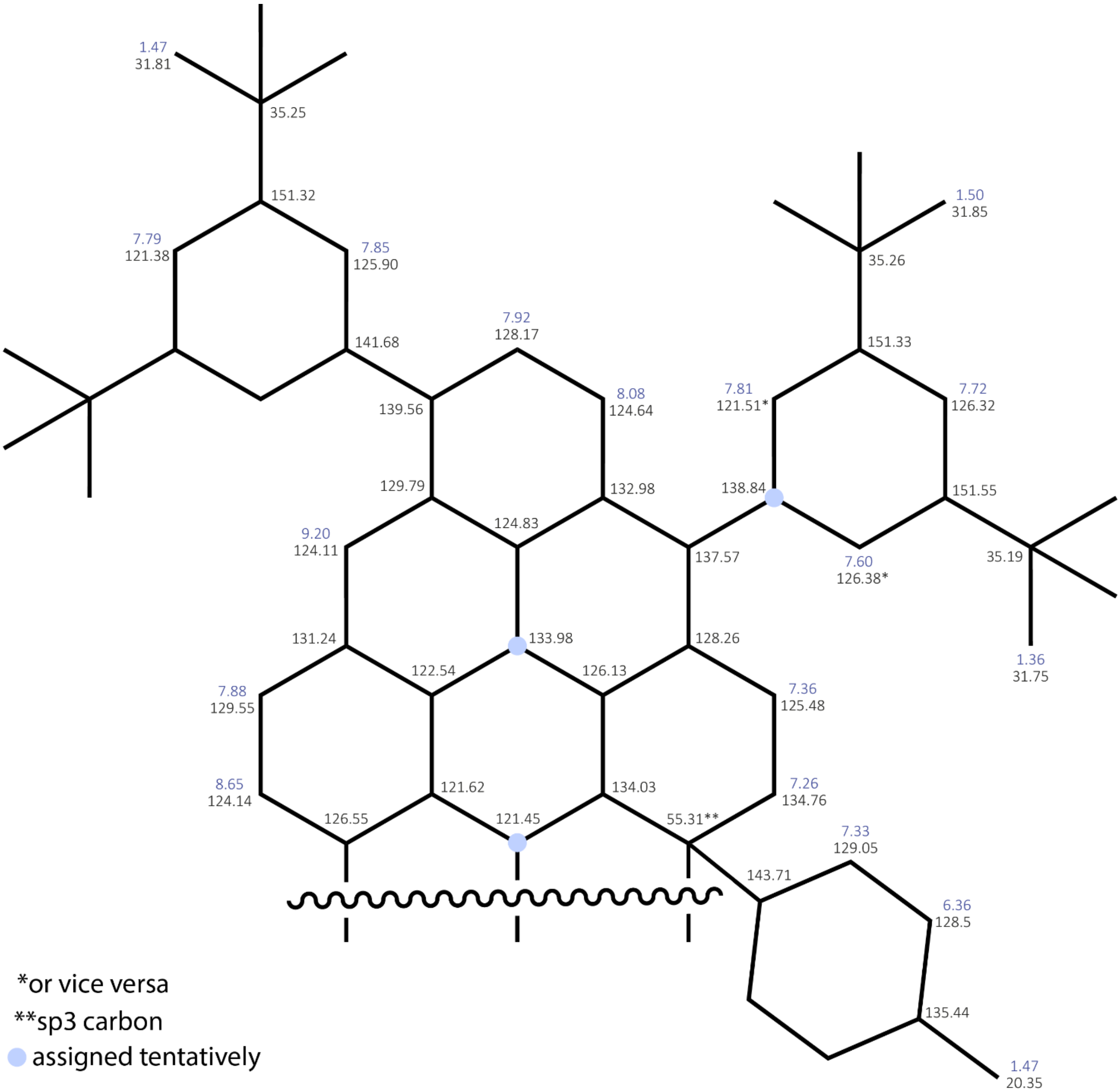

Figure S52. Assignment of ^{1}H (blue) and ^{13}C (black) NMR resonances (in ppm) of **5-Tol**. π-Bonds are omitted for clarity.

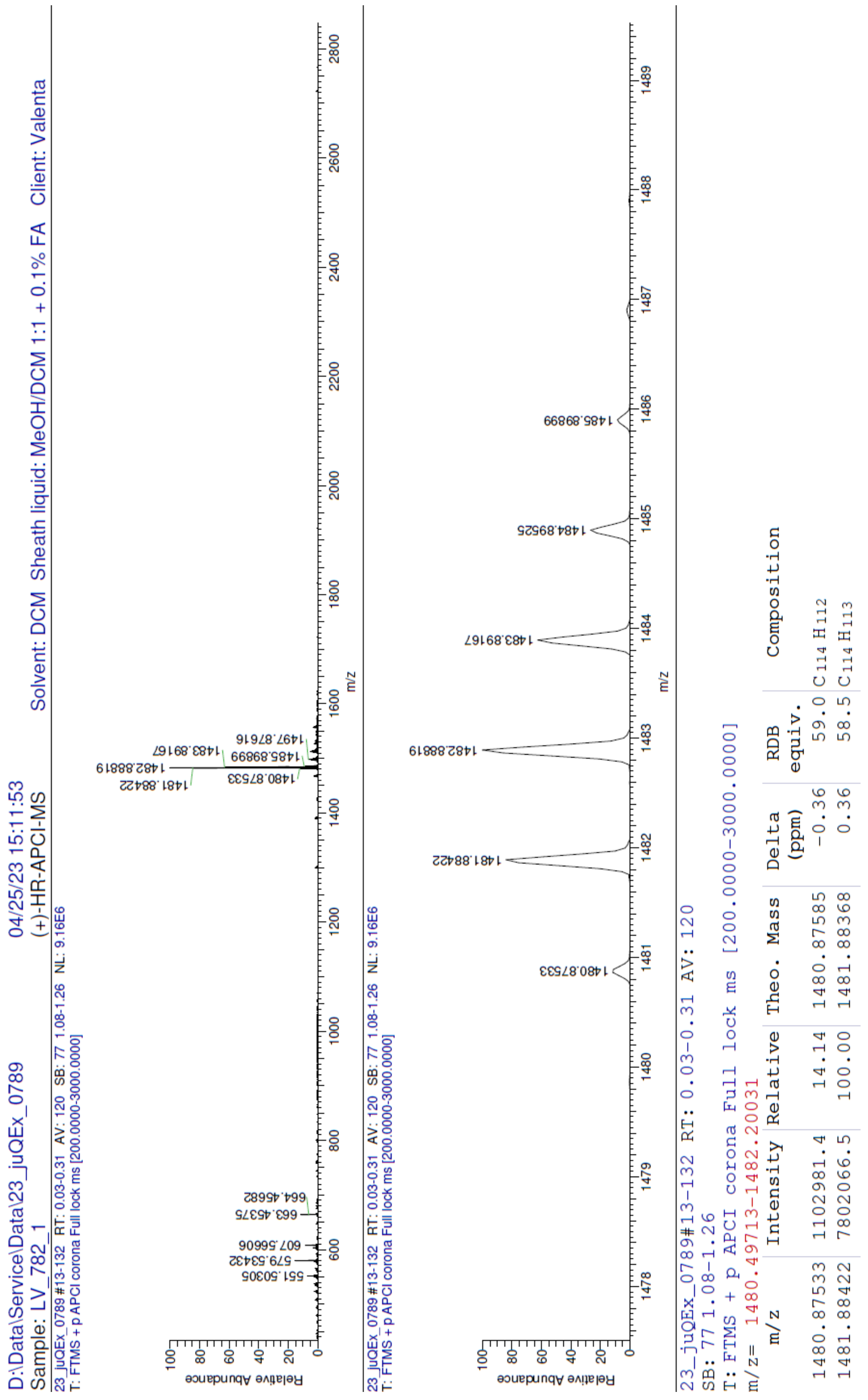

Figure S53. HRMS (APCI) of **5-Tol**.

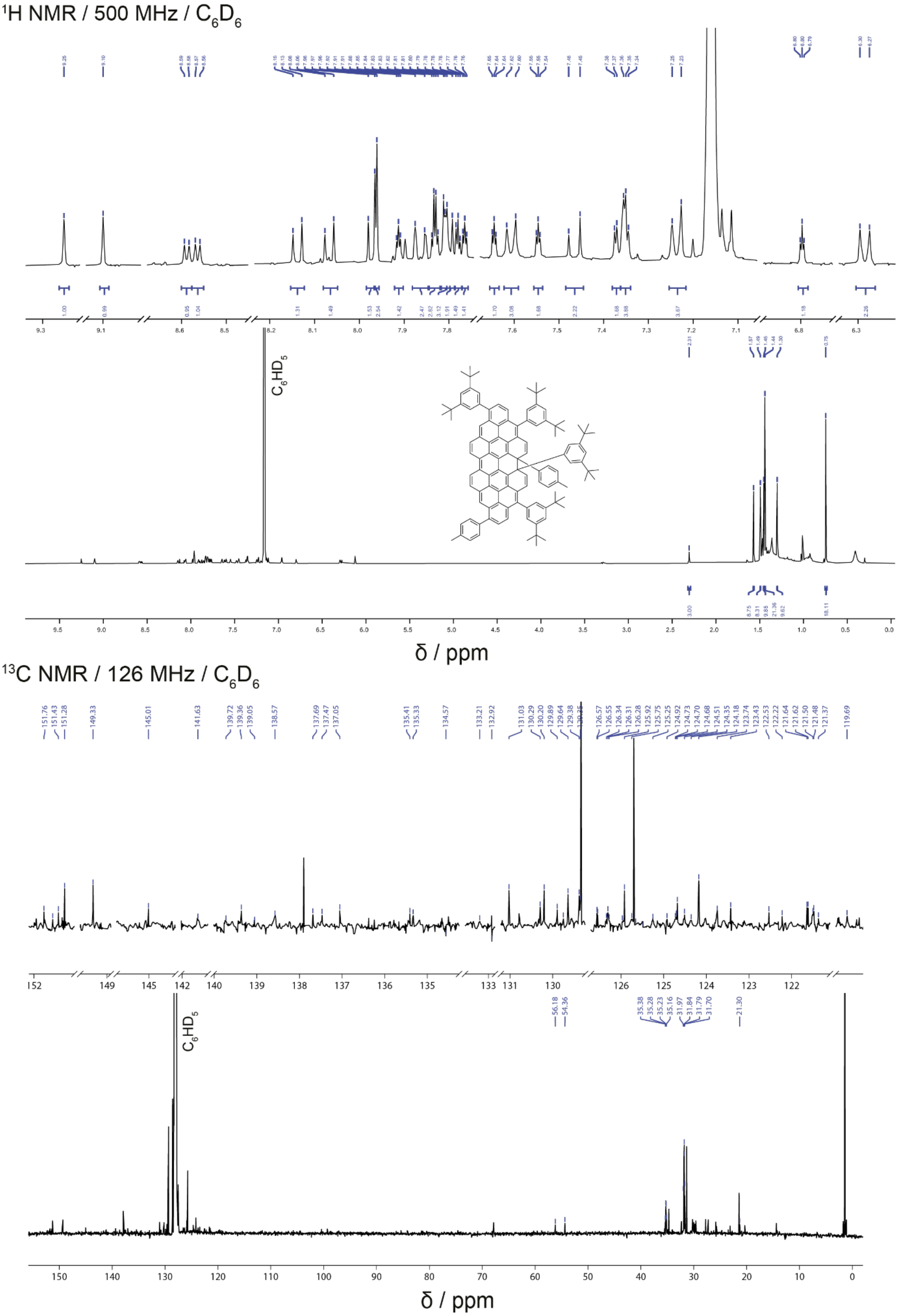

Figure S54. ¹H NMR (top) and ¹³C NMR (bottom) of **6-Tol** in C_6D_6.

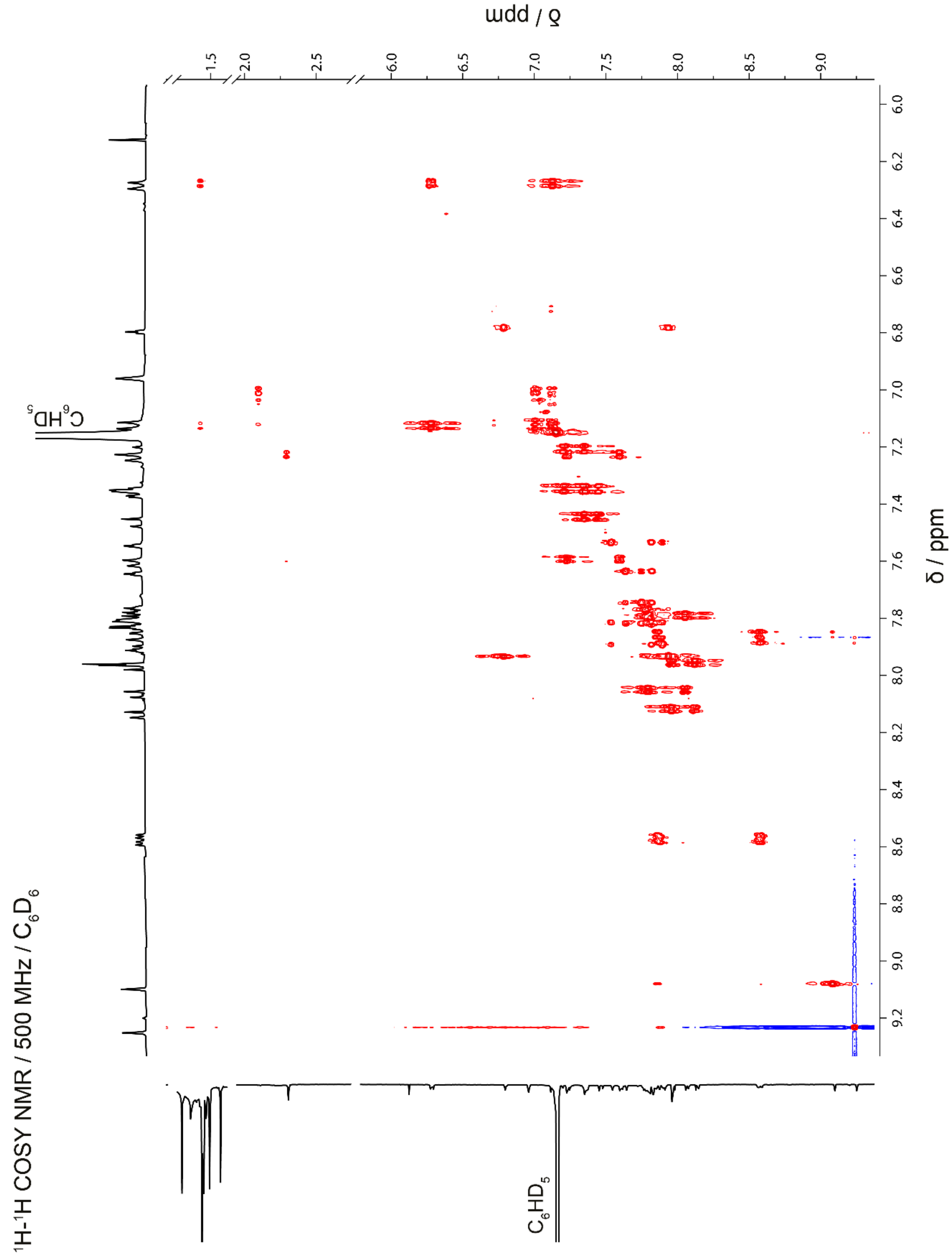

Figure S55. ^1H–^1H COSY NMR of **6-Tol** in C_6D_6.

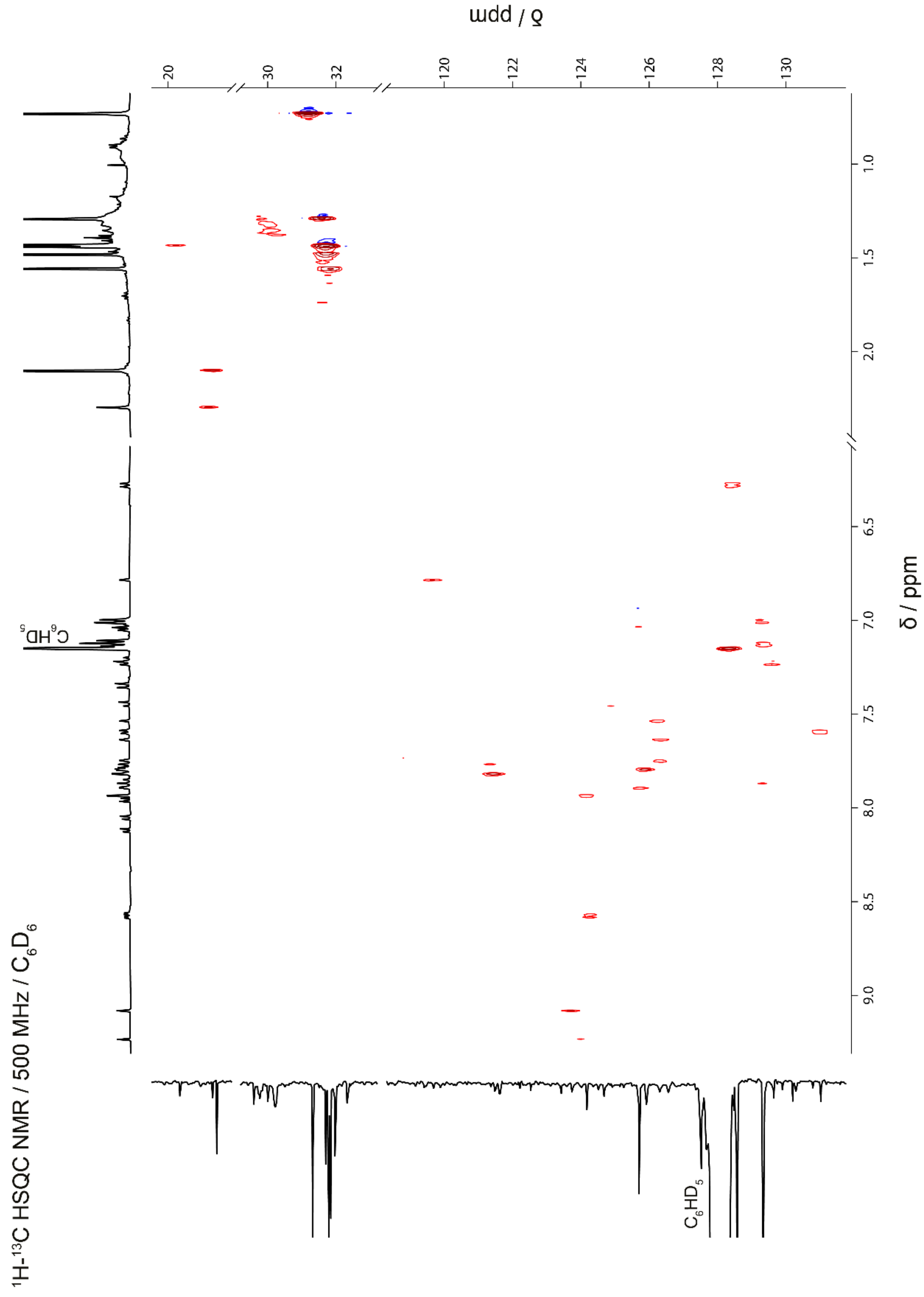

Figure S56. ^{1}H–^{13}C HSQC NMR of **6-Tol** in C_6D_6.

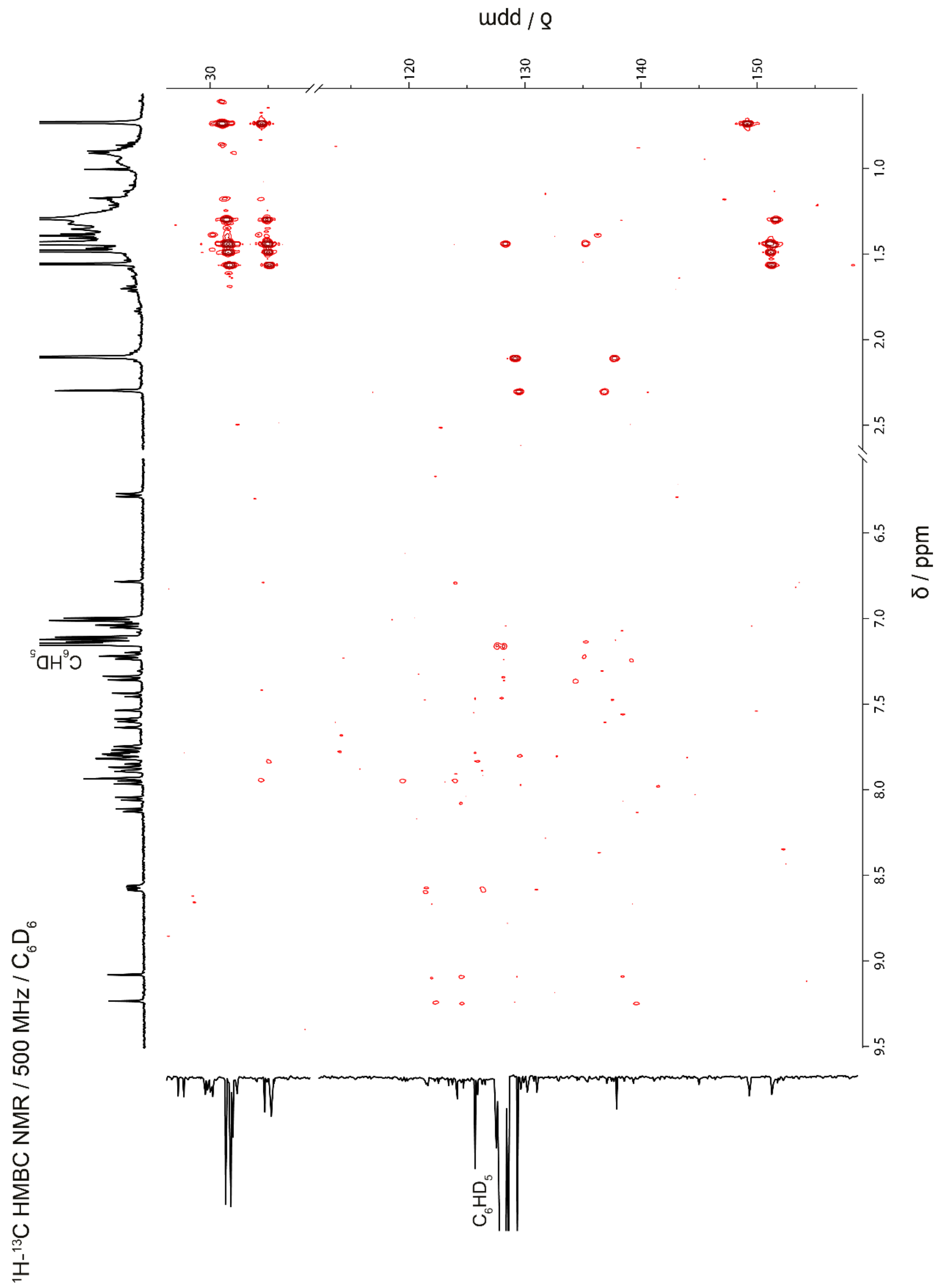

Figure S57. ^{1}H–^{13}C HMBC NMR of **6-Tol** in C_6D_6.

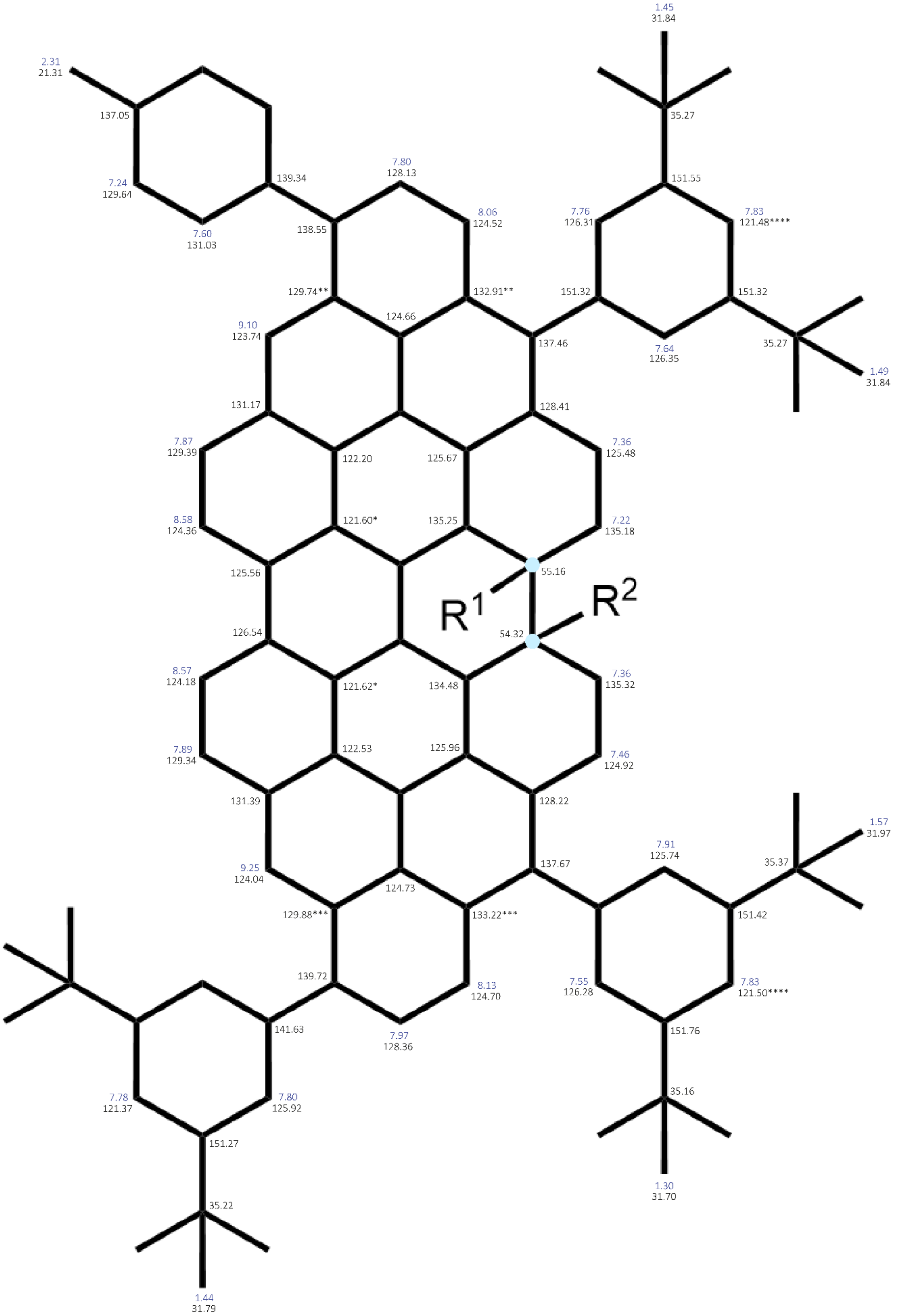

*/**/***/**** or vice versa

sp^3 carbon

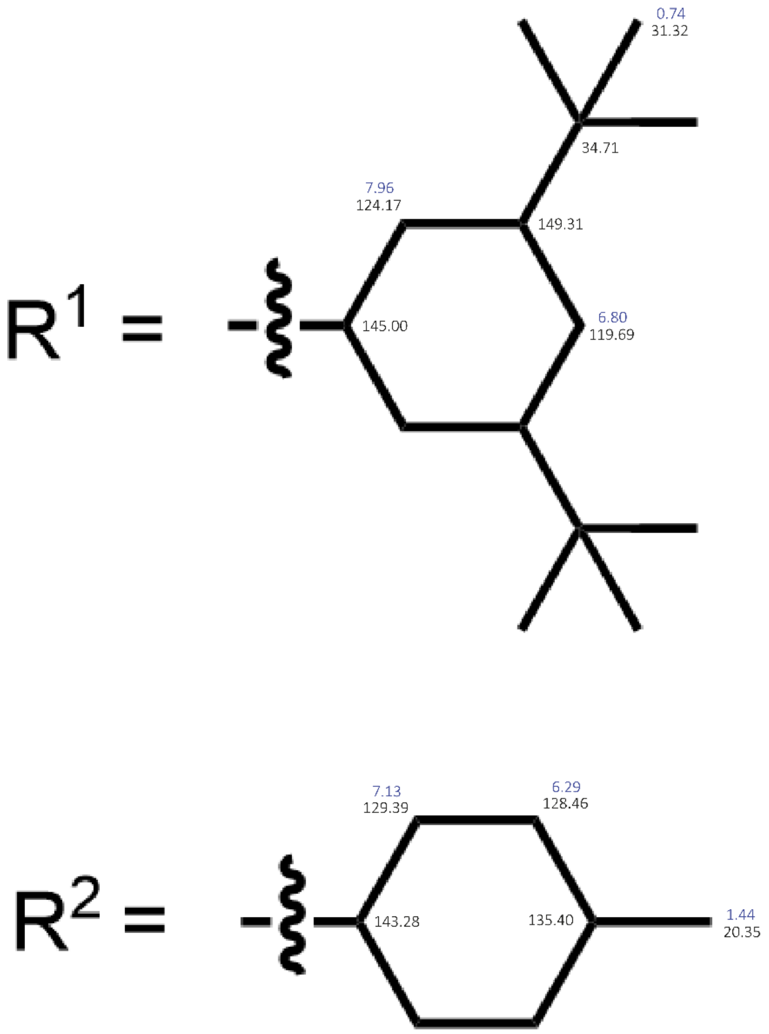

Figure S58. Assignment of ^{1}H (blue) and ^{13}C (black) NMR resonances (in ppm) of **6-Tol**. π-Bonds are omitted for clarity.

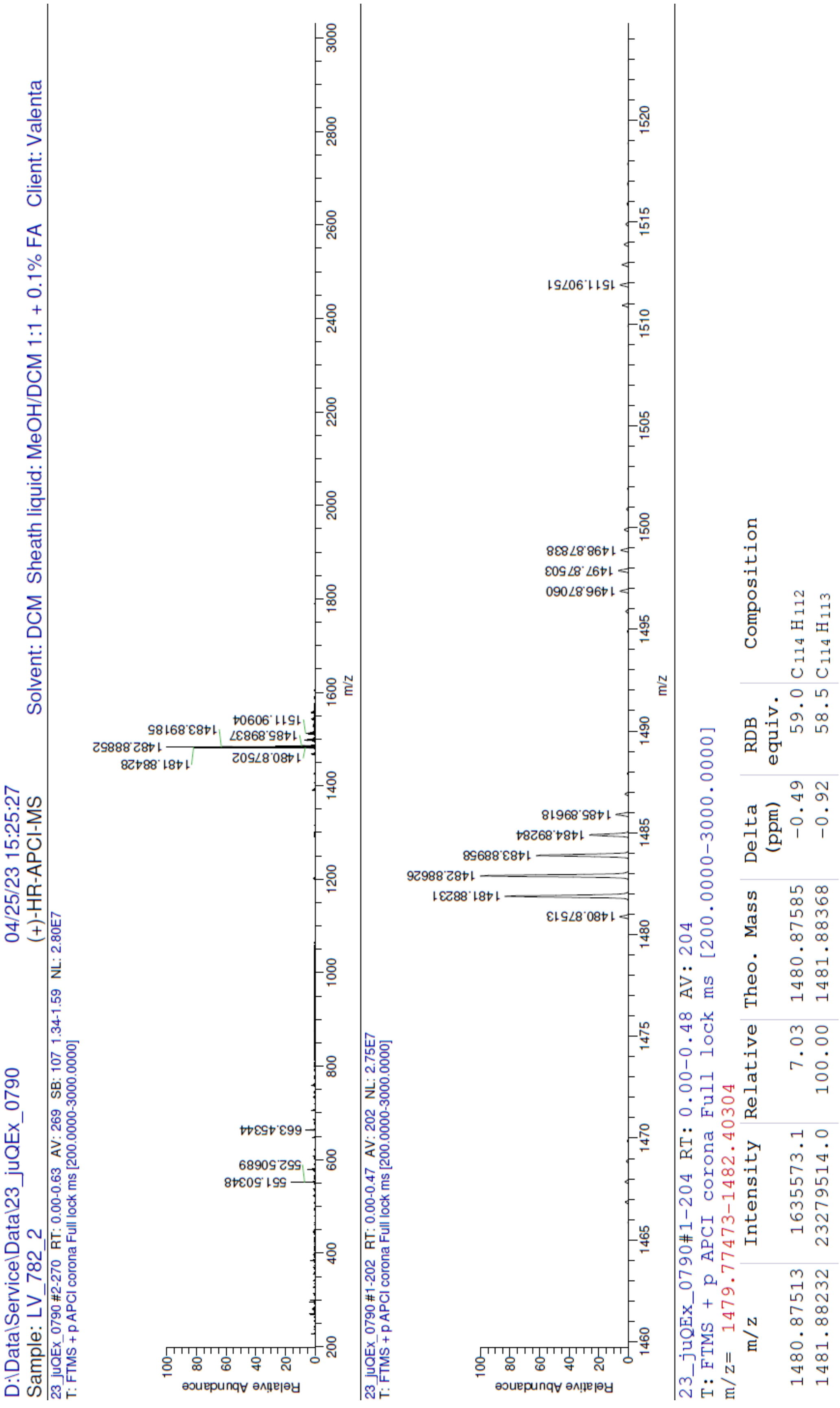

Figure S59. HRMS (APCI) of **6-Tol**.

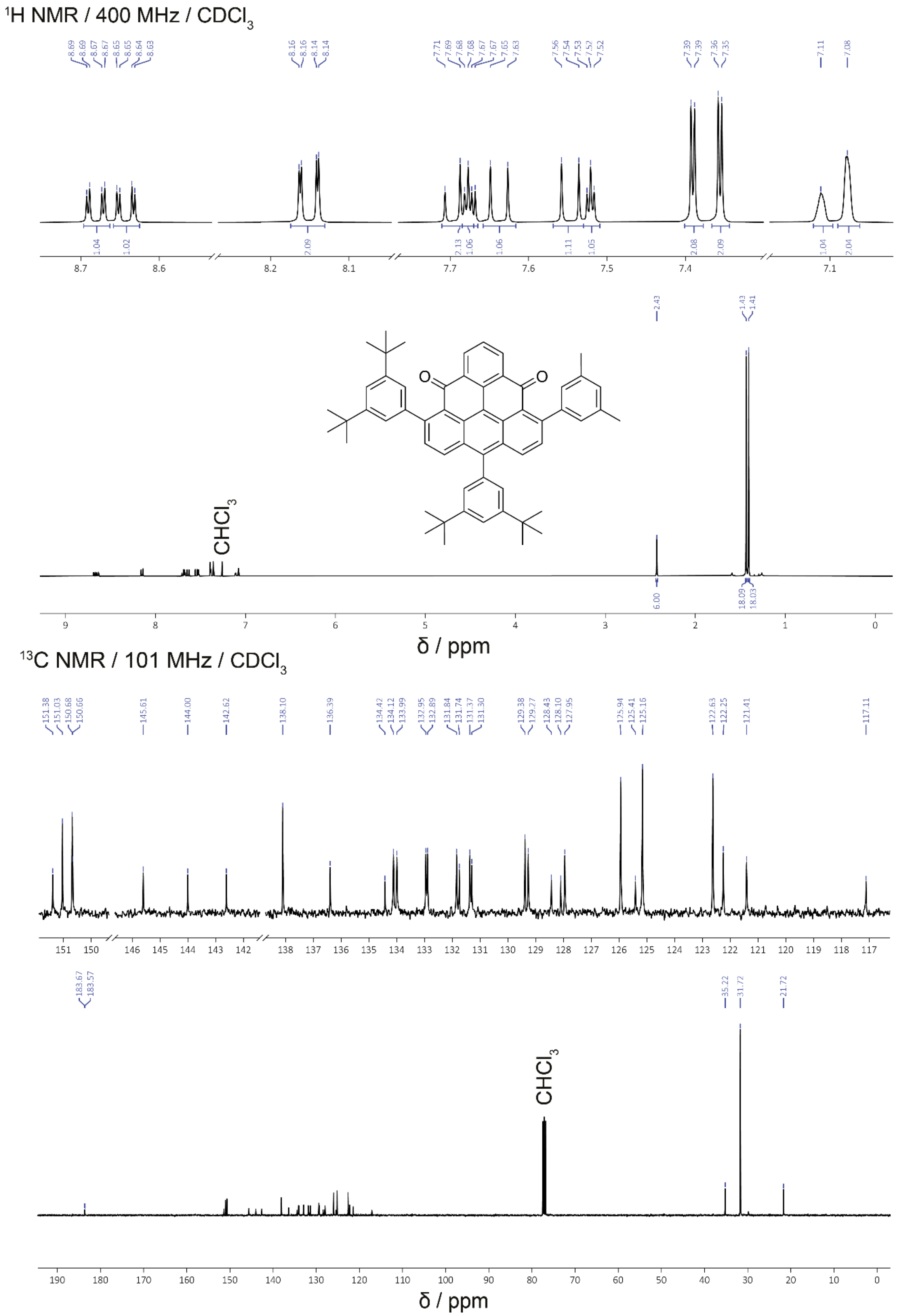

Figure S60. ^{1}H NMR (top) and ^{13}C NMR (bottom) of **18-Xyl** in $CDCl_3$.

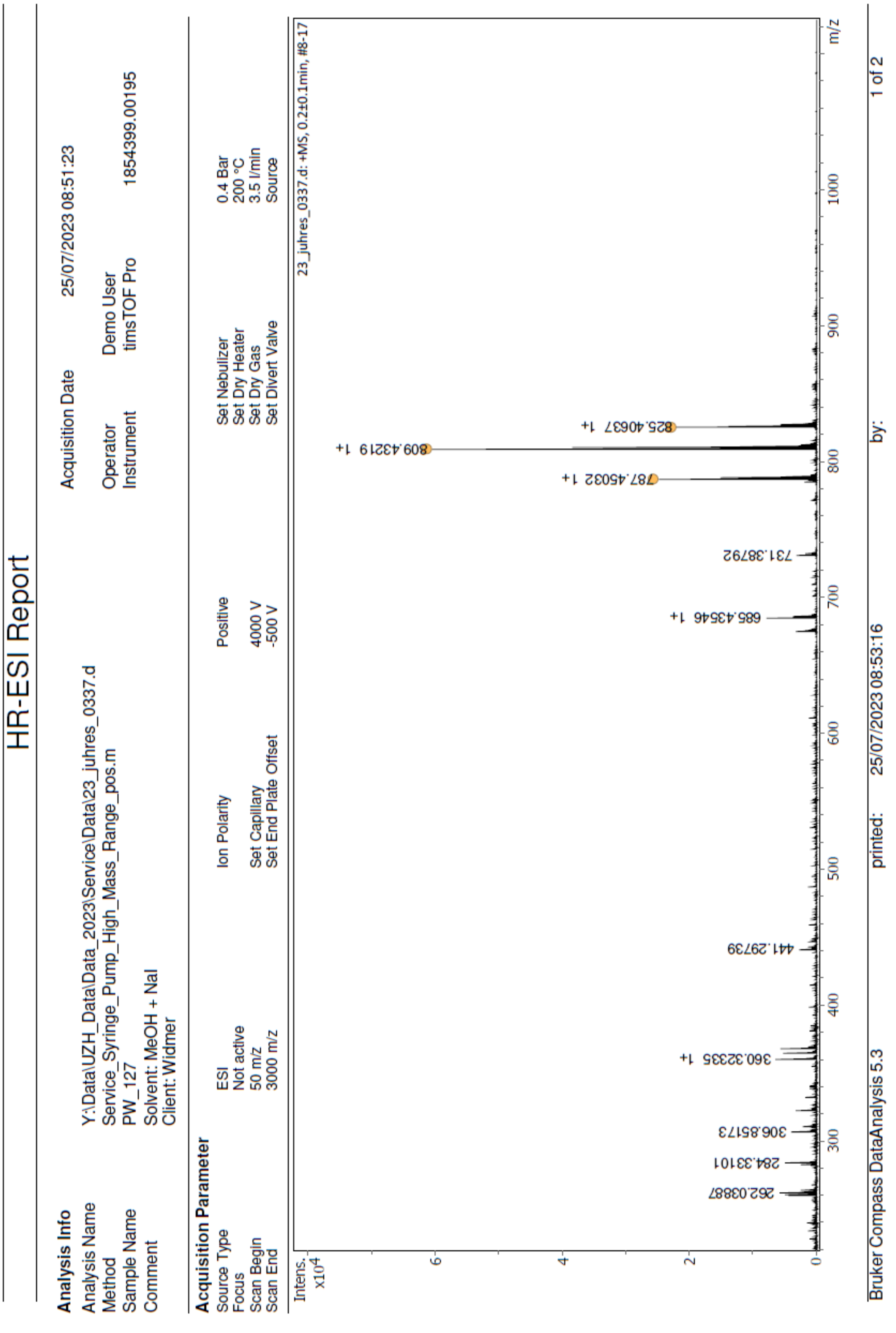

Figure S61. HRMS (ESI) of **18-Xyl**.

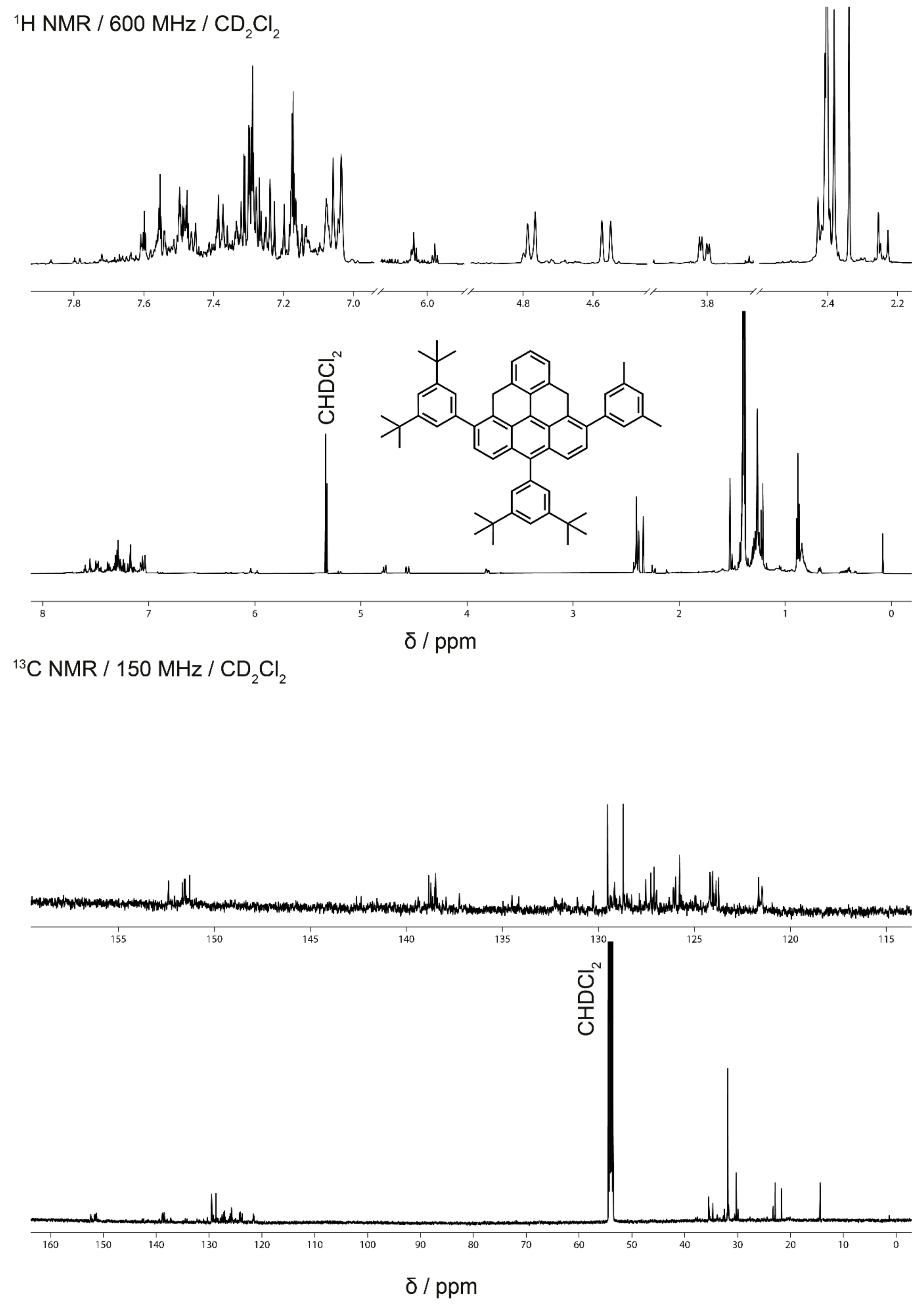

Figure S62. ^{1}H NMR (top) and ^{13}C NMR (bottom) of **4-Xyl** in CD_2Cl_2.

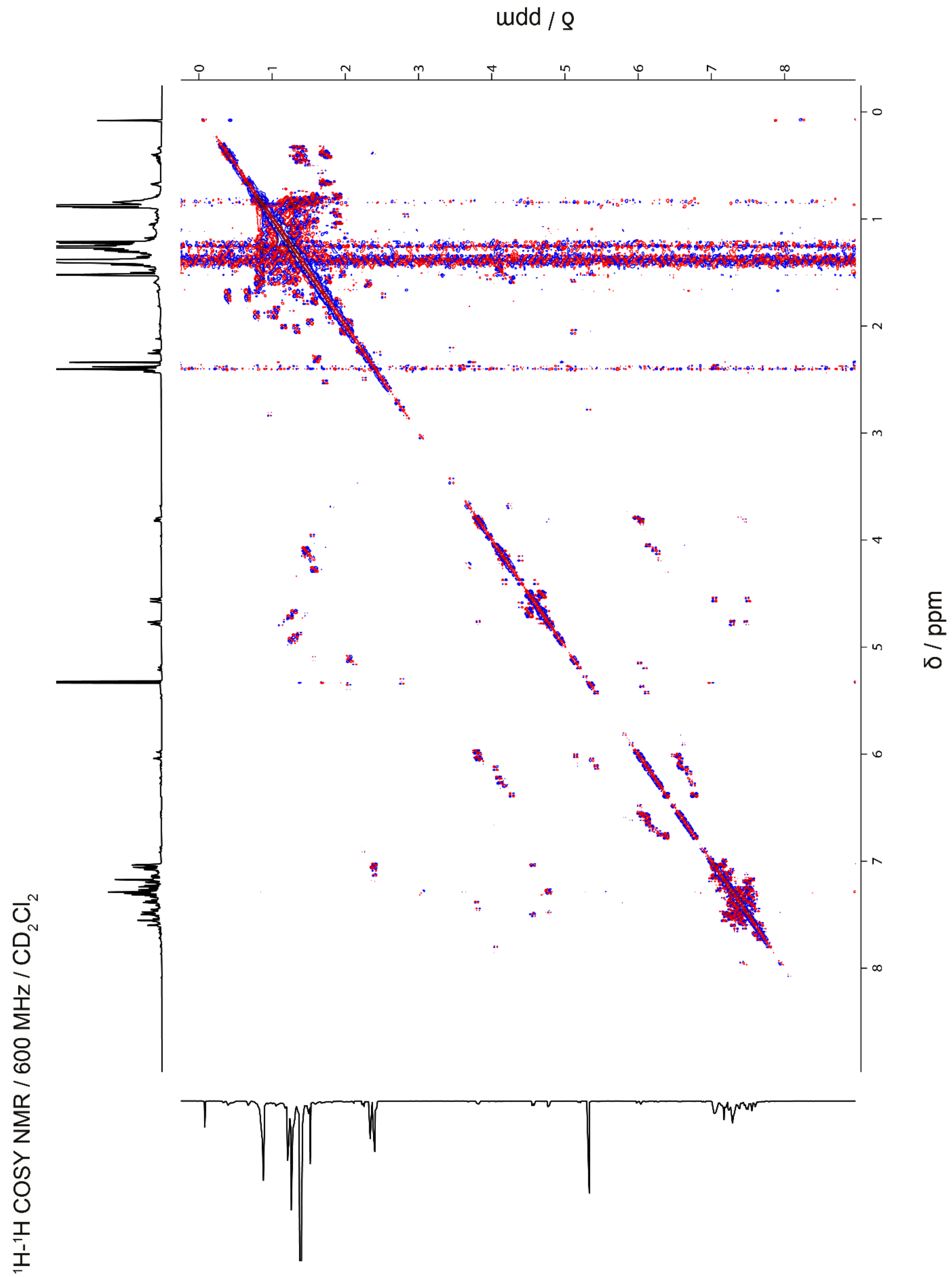

Figure S63. ^{1}H–^{1}H COSY NMR of **4-Xyl** in CD_2Cl_2.

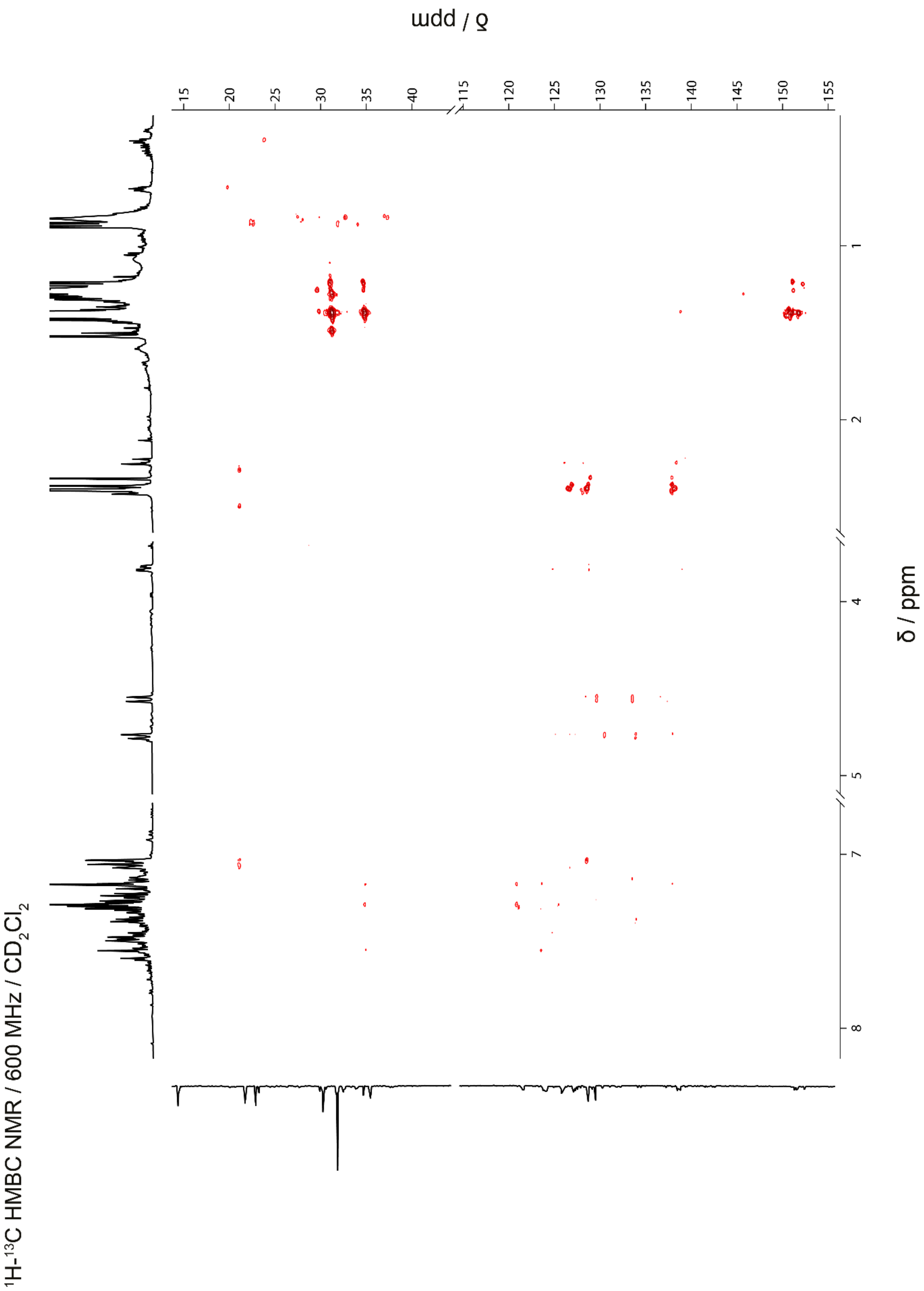

Figure S64. ^{1}H–^{13}C HMBC NMR of **4-Xyl** in CD_2Cl_2.

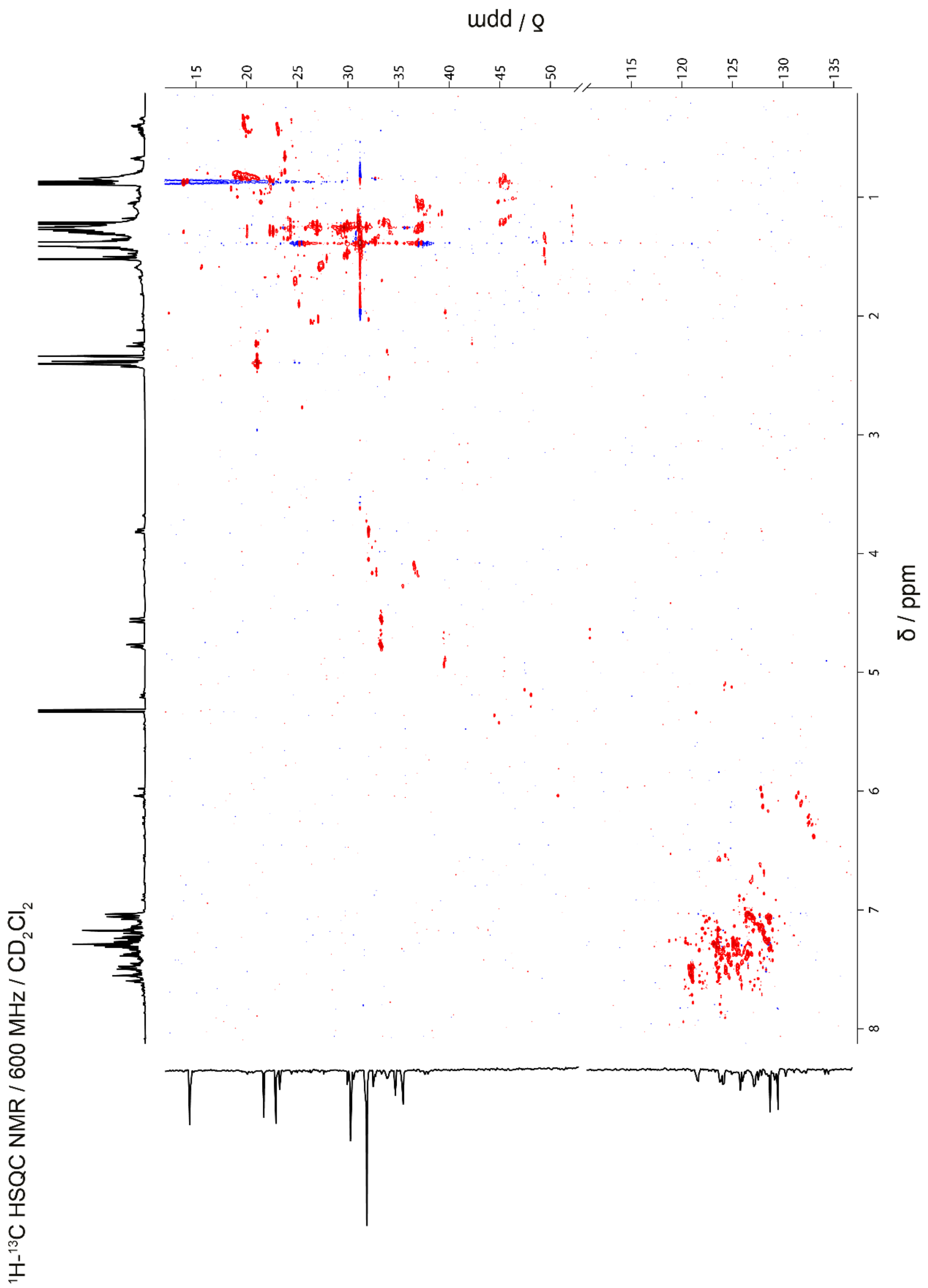

Figure S65. ^{1}H–^{13}C HSQC NMR of **4-Xyl** in CD_2Cl_2.

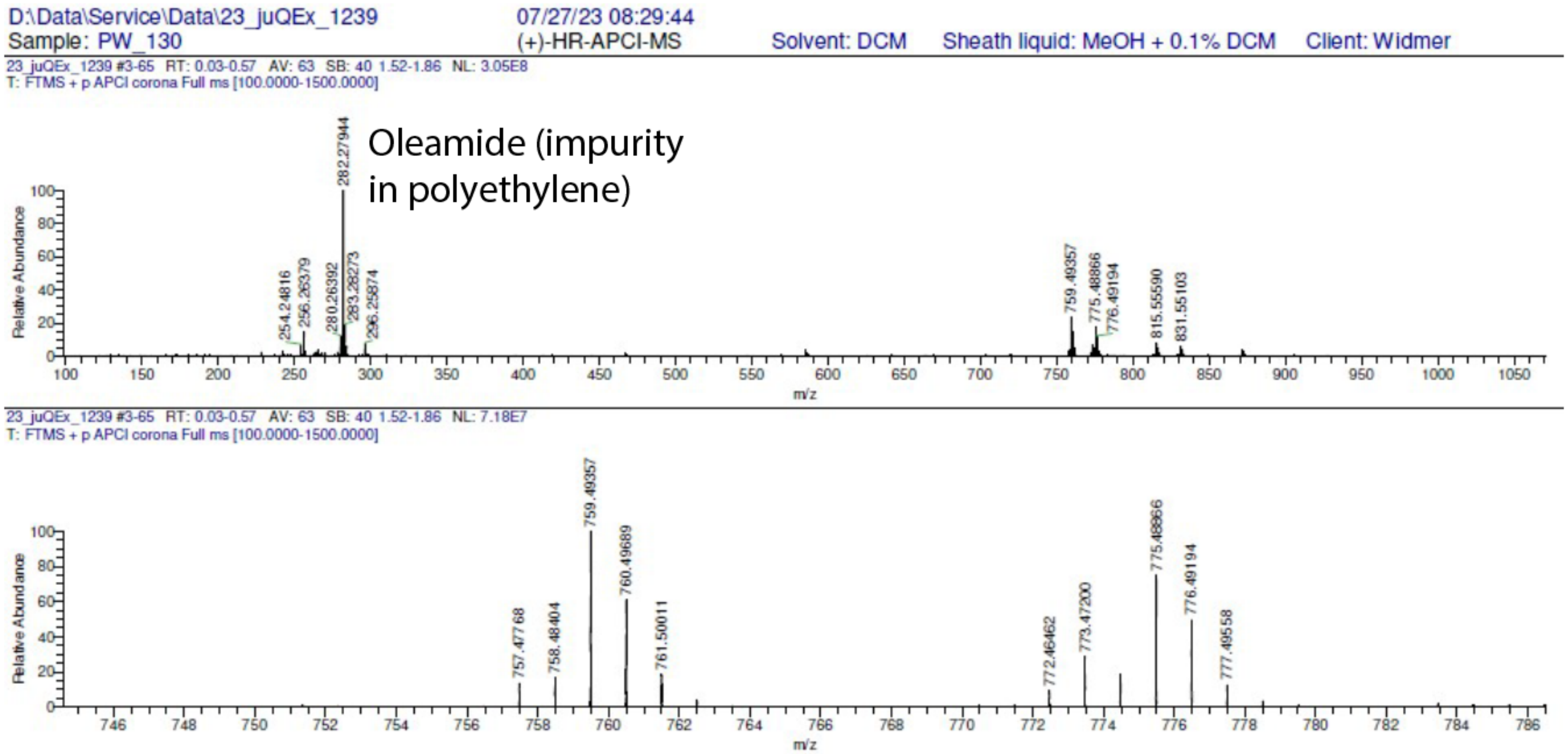

23_juQEx_1239#3-65 RT: 0.03-0.57 AV: 63
SB: 40 1.52-1.86
T: FTMS + p APCI corona Full ms [100.0000-1500.0000]
m/z= 759.02316-775.99110

m/z	Intensity	Relative	Theo. Mass	Delta (ppm)	RDB equiv.	Composition
759.49357	72745104.0	100.00	759.49243	1.51	27.5	$C_{58}H_{63}$
760.49689	44869260.0	61.68				
773.47200	21089516.0	28.99	773.47169	0.40	28.5	$C_{58}H_{61}O$
775.48866	55353560.0	76.09	775.48734	1.70	27.5	$C_{58}H_{63}O$

Figure S66. HRMS (APCI) of **4-Xyl**.

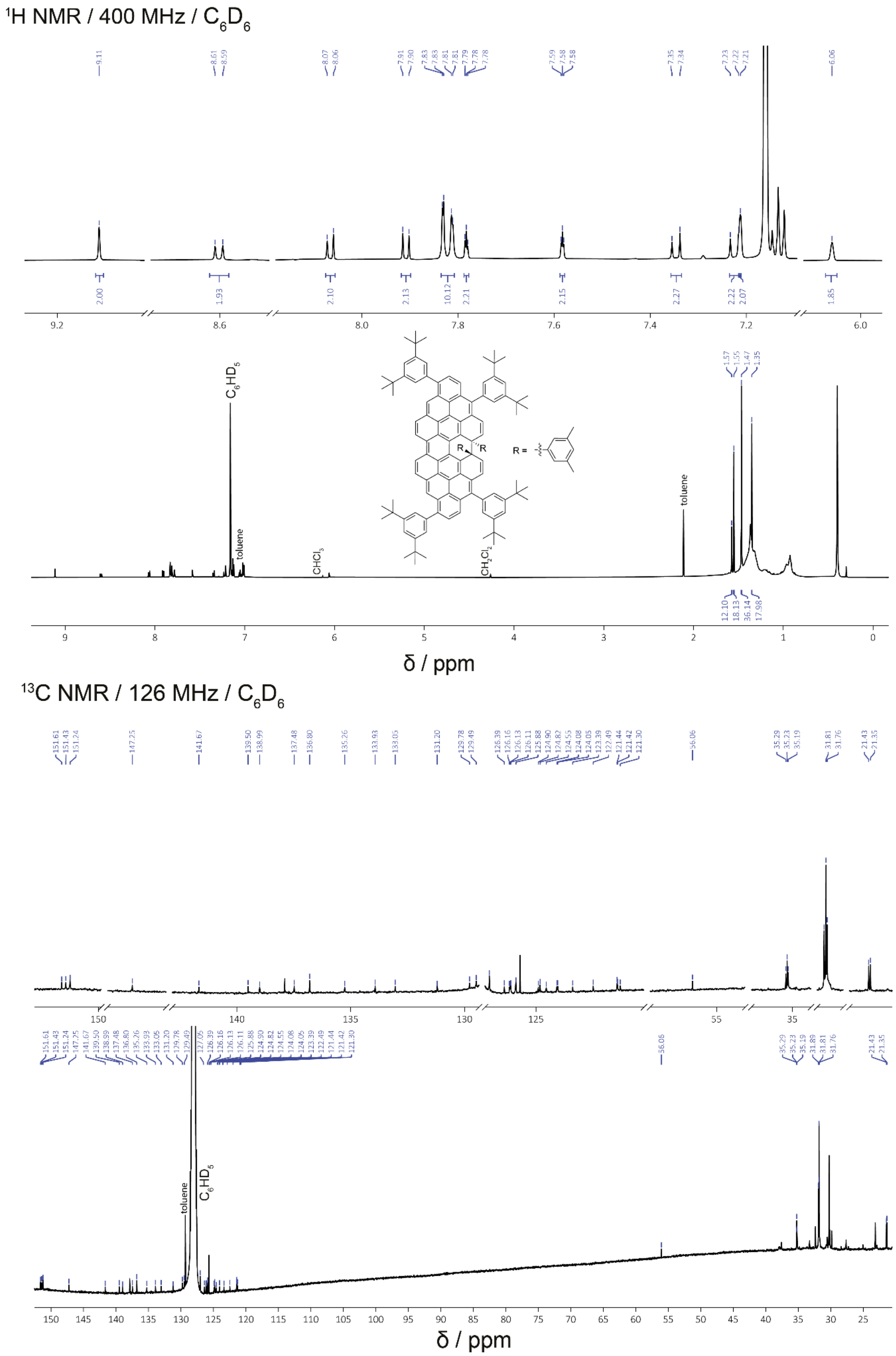

Figure S67. ^{1}H NMR (top) and ^{13}C NMR (bottom) of **5-Xyl** in C_6D_6.

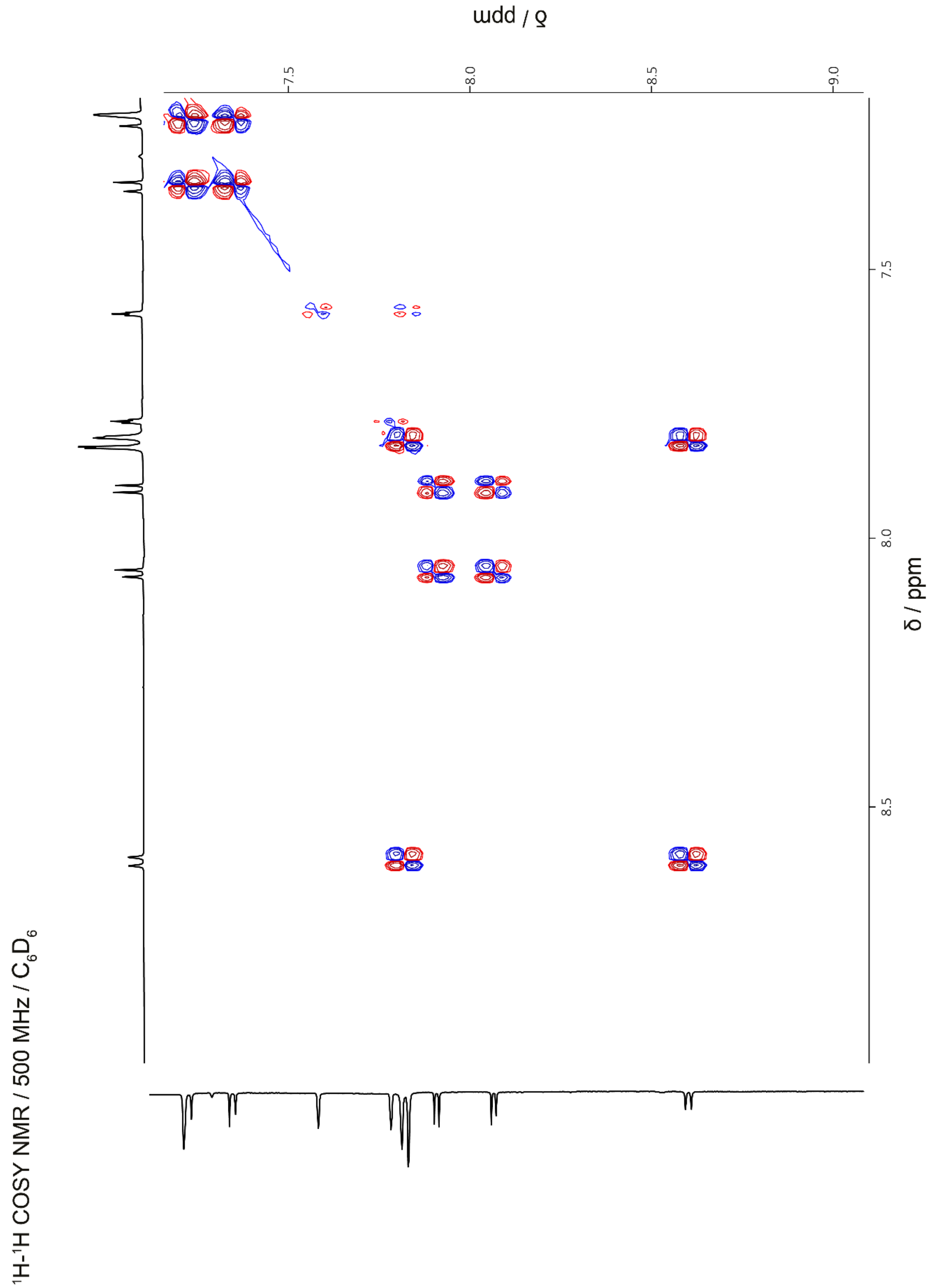

Figure S68. ^1H–^1H COSY NMR of **5-Xyl** in C_6D_6.

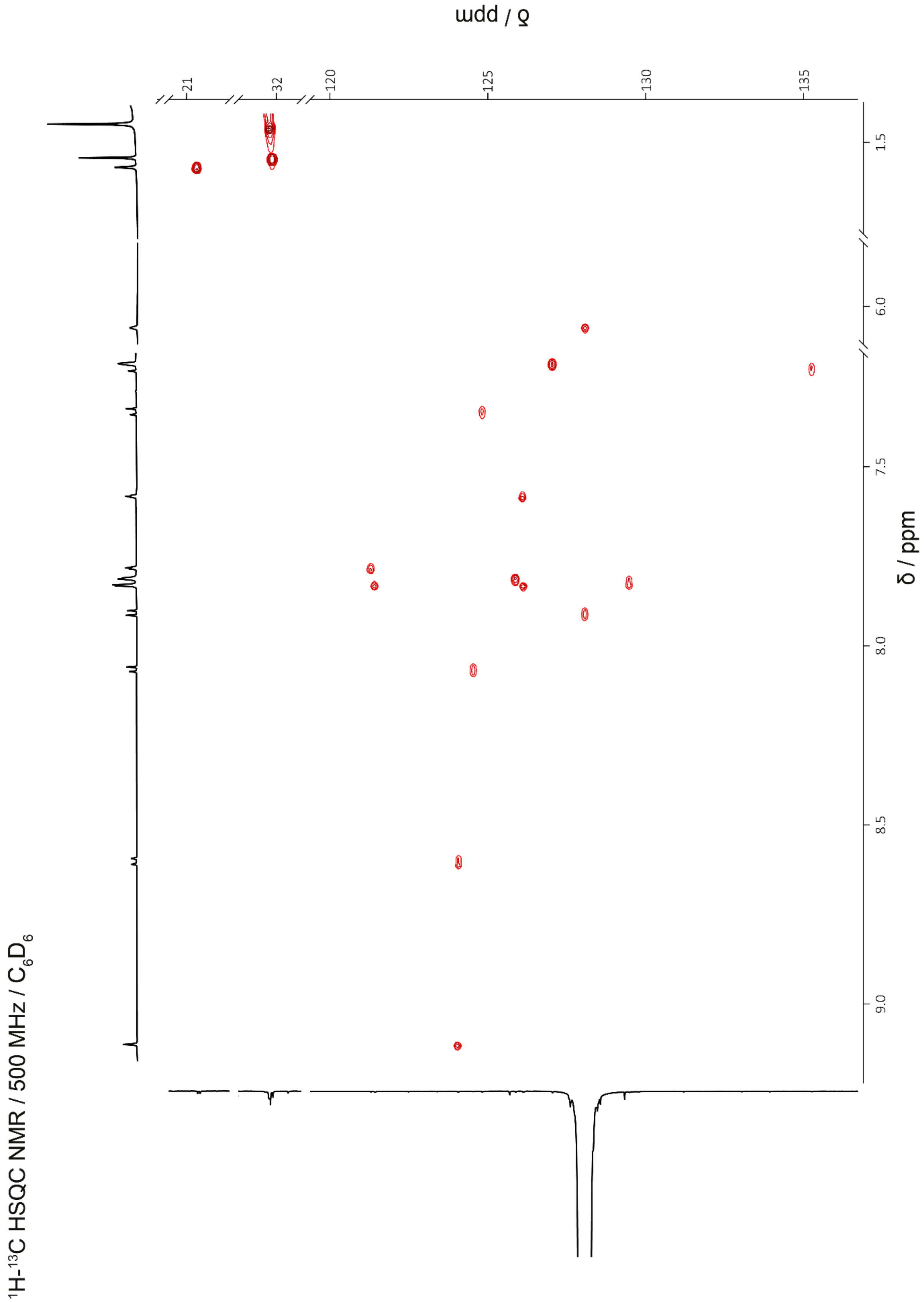

Figure S69. ^{1}H–^{13}C HSQC NMR of **5-Xyl** in C_6D_6.

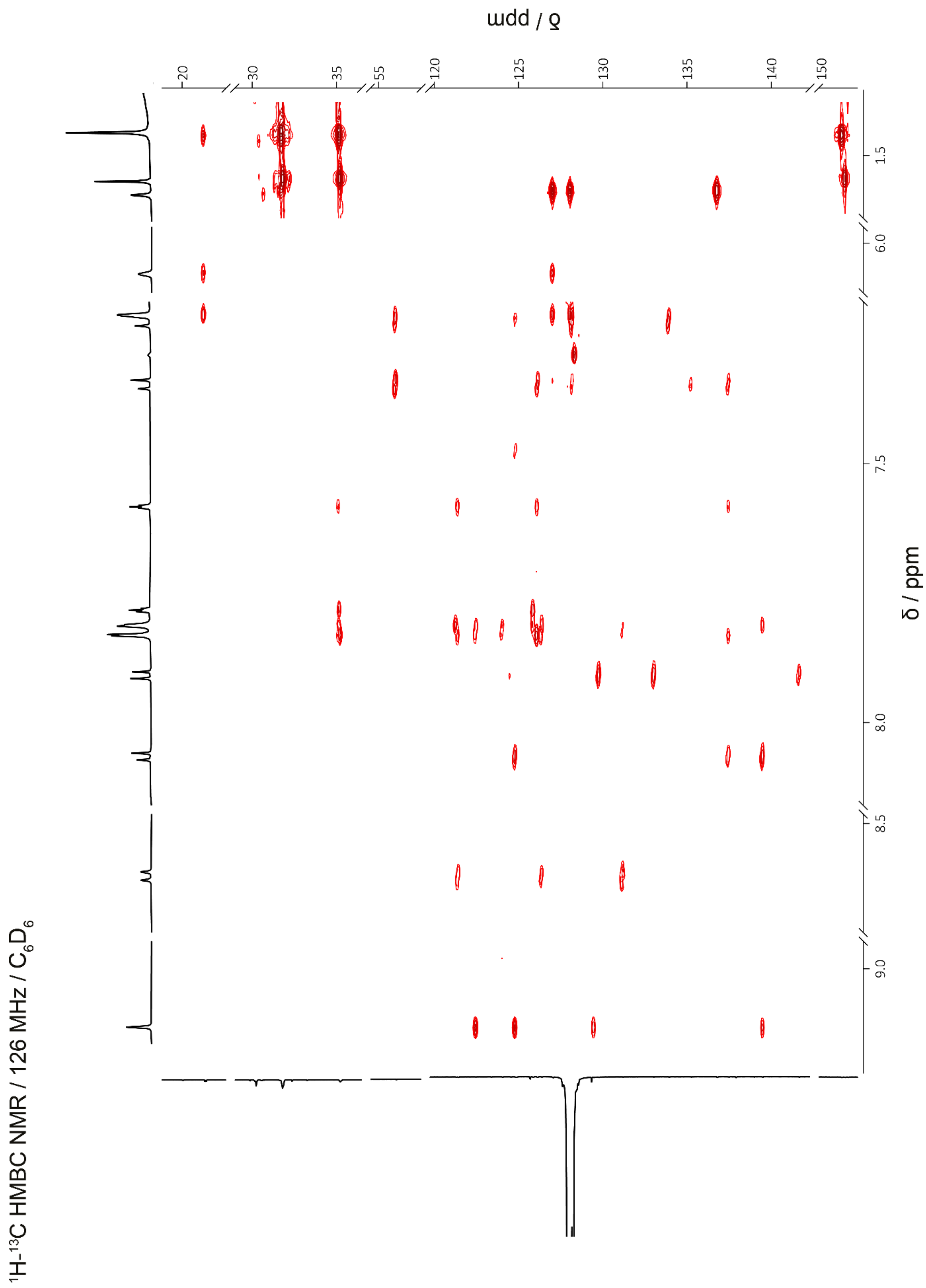

Figure S70. ^1H–^{13}C HMBC NMR of **5-Xyl** in C_6D_6.

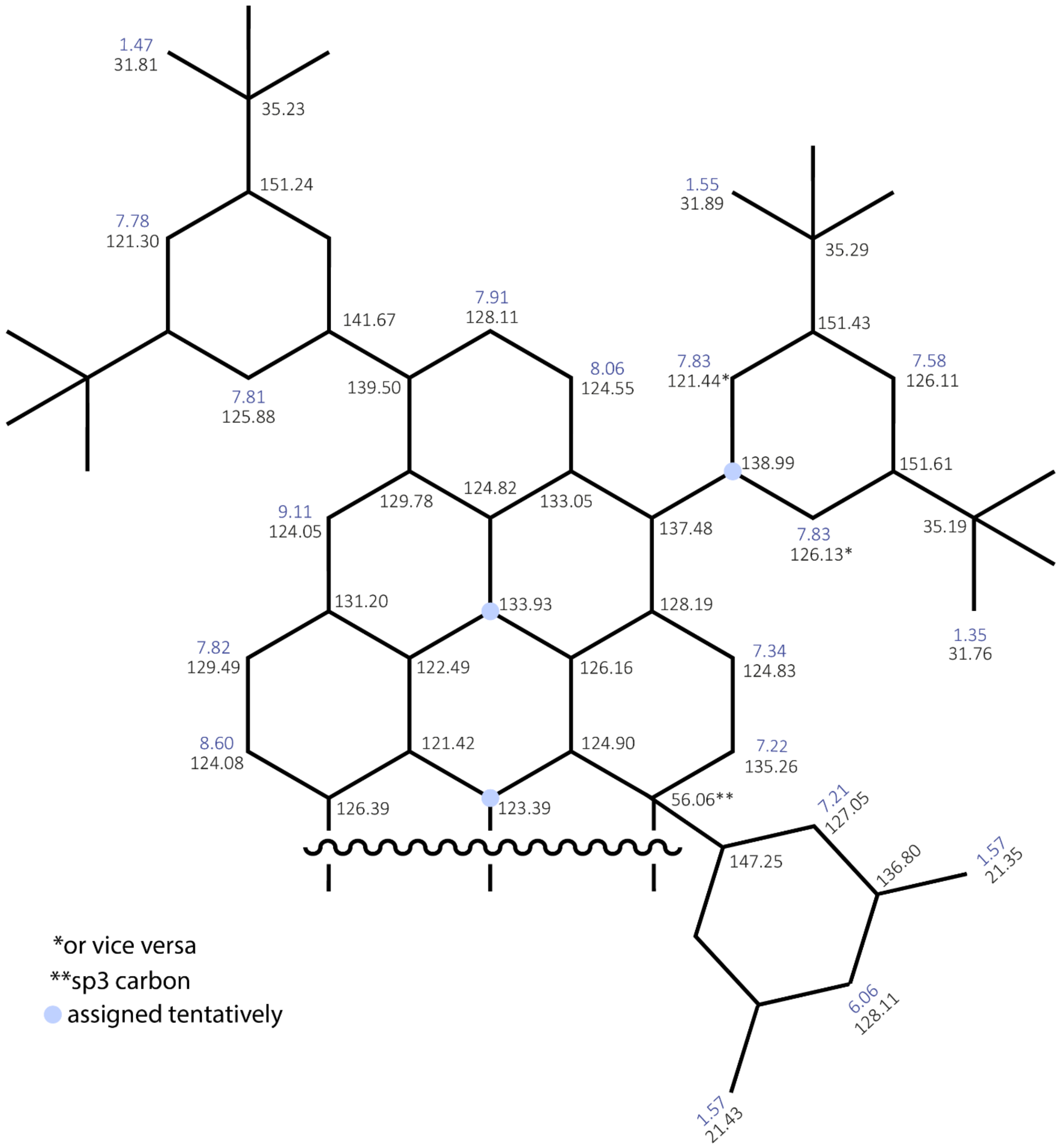

Figure S71. Assignment of ^{1}H (blue) and ^{13}C (black) NMR resonances (in ppm) of **5-Xyl**. π-Bonds are omitted for clarity.

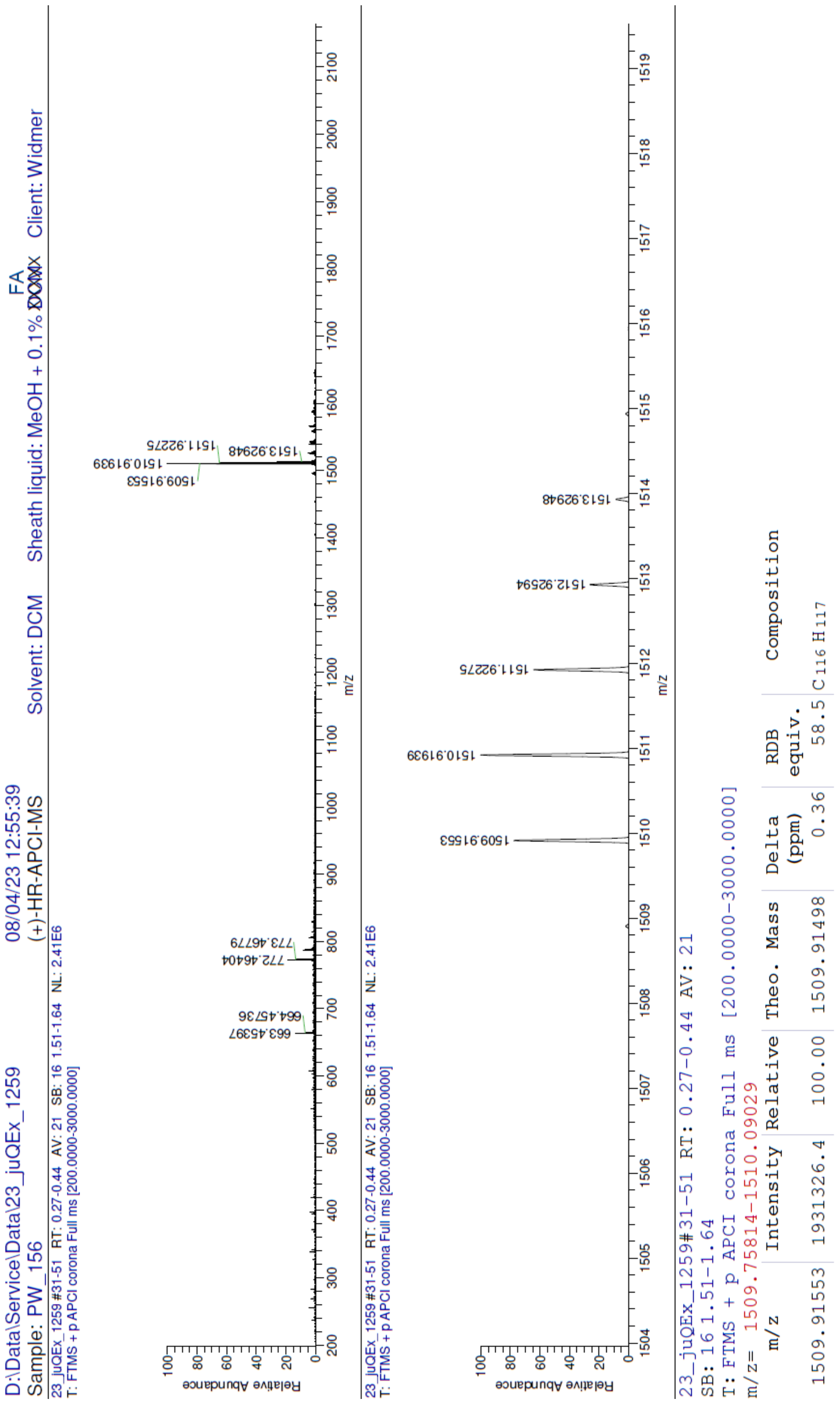

Figure S72. HRMS (APCI) of **5-Xyl**.

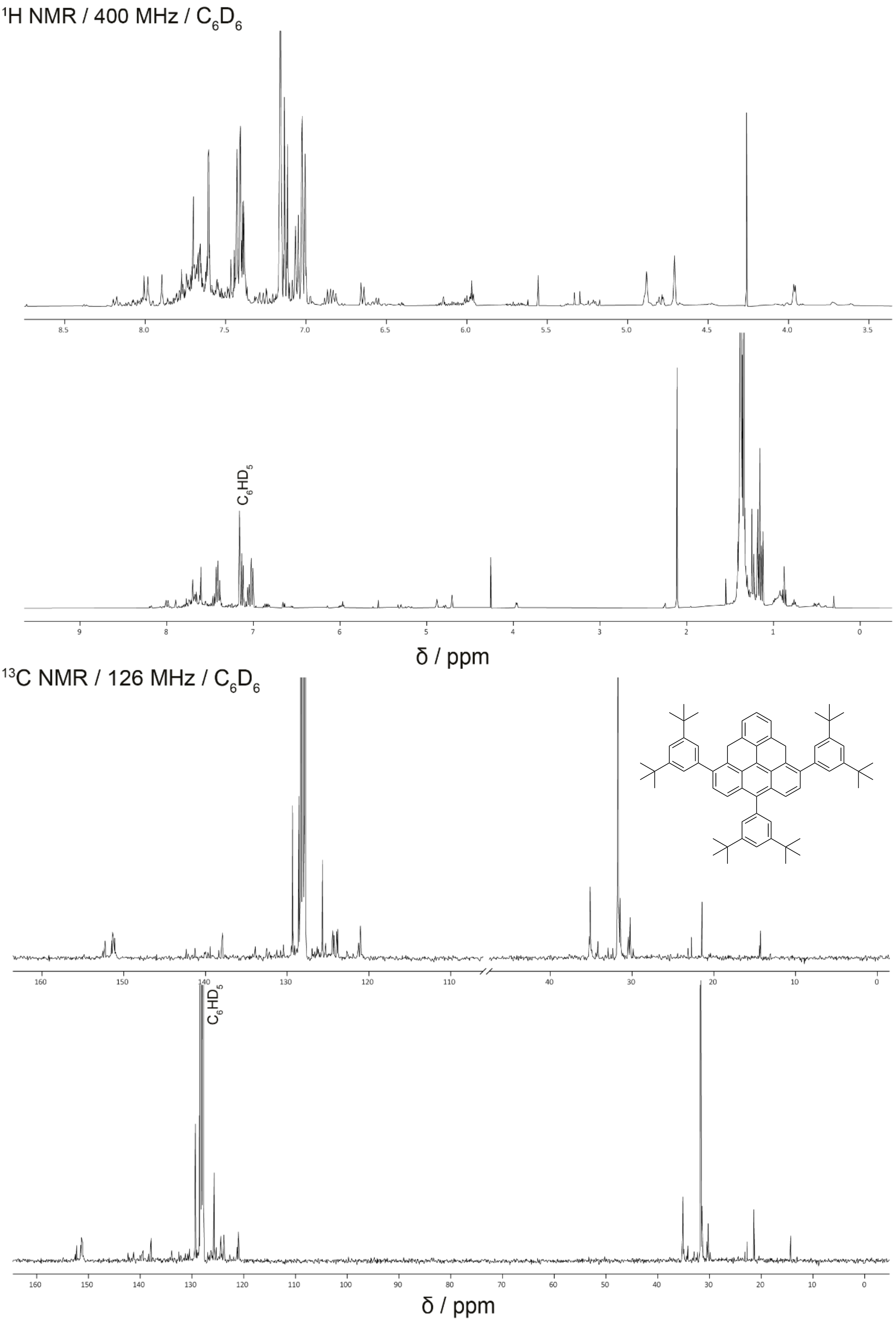

Figure S73. ^{1}H NMR (top) and ^{13}C NMR (bottom) of **4-tBP** in C_6D_6.

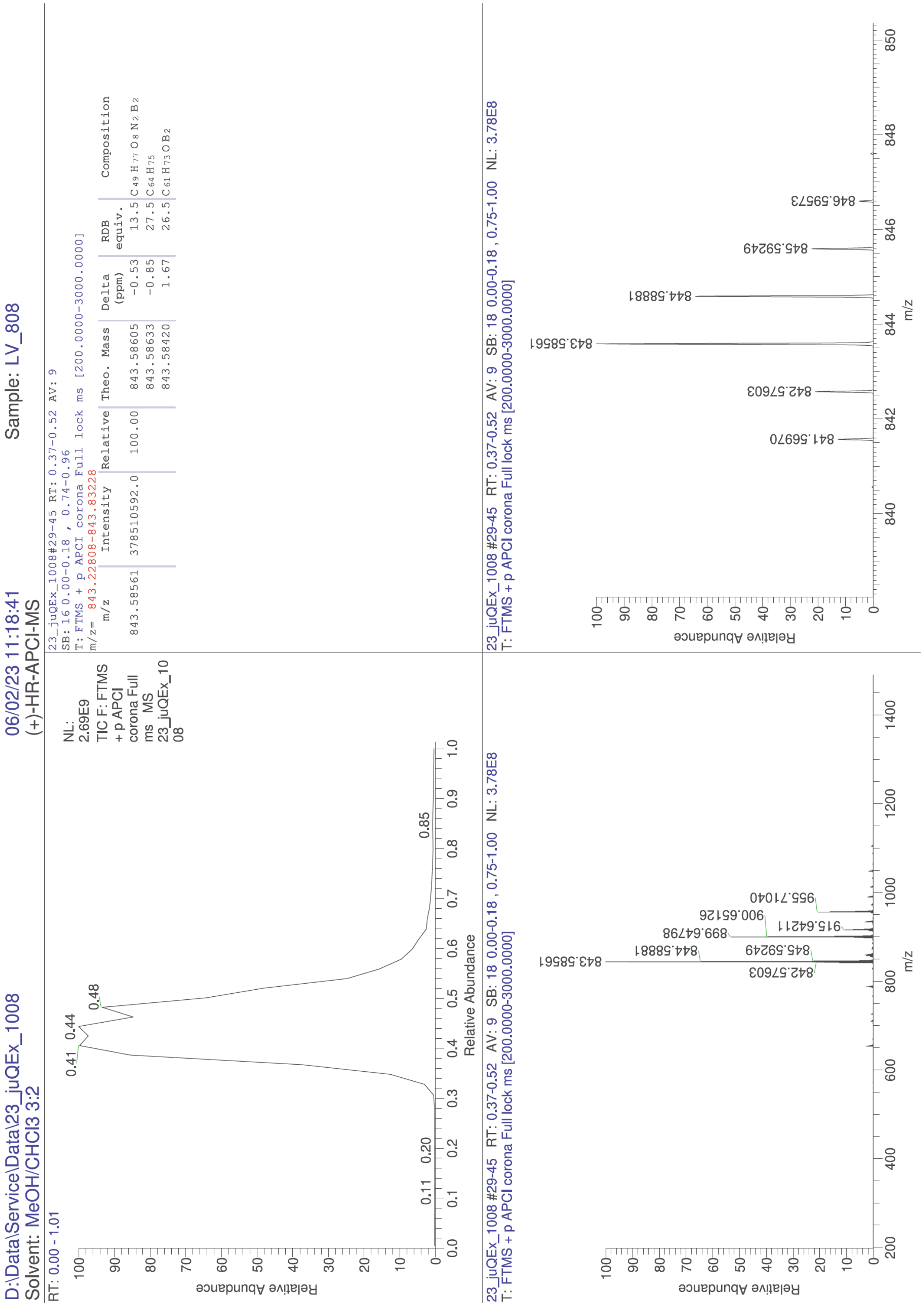

Figure S74. HRMS (APCI) of **4-tBP**.

5. Additional Spectra

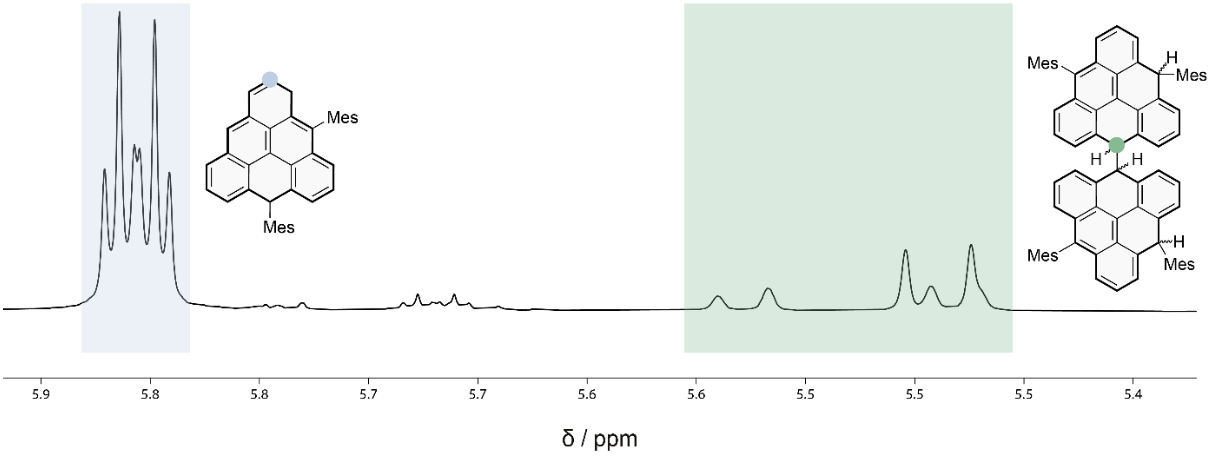

Figure S75. Cutout of the ^{1}H NMR of a reaction mixture of compound **3-Mes** and **3-Mes** + 0.45 equivalents of CA in C_6D_6. CA = *p*-chloranil.

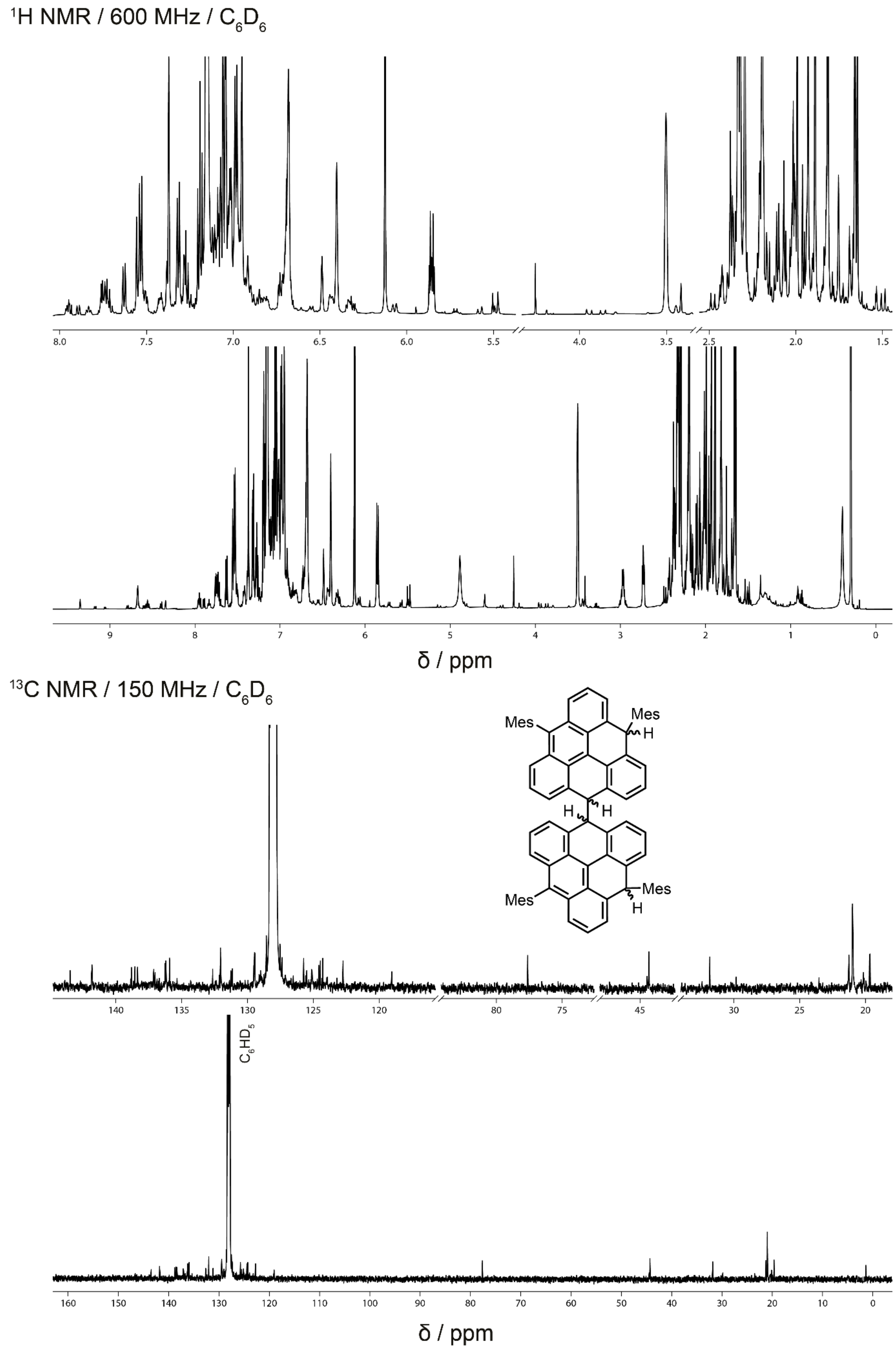

Figure S76. ^{1}H NMR (top) and ^{13}C NMR (bottom) of the reaction mixture of compound **3-Mes** + 0.45 equivalents of CA in C_6D_6. CA = *p*-chloranil.

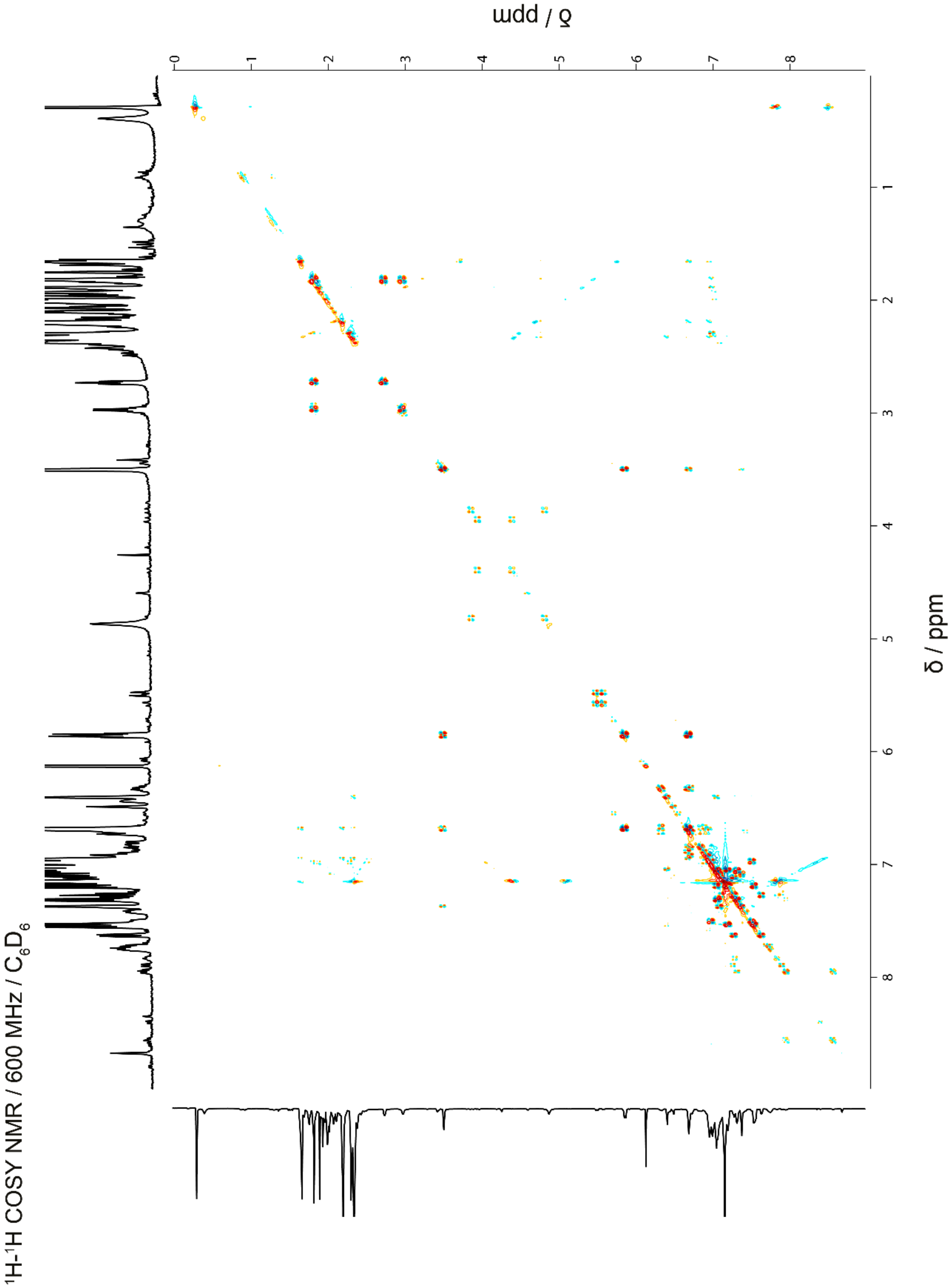

Figure S77. ^{1}H–^{1}H COSY NMR of **3-Mes** + 0.45 equivalents of CA in C_6D_6. CA = *p*-chloranil.

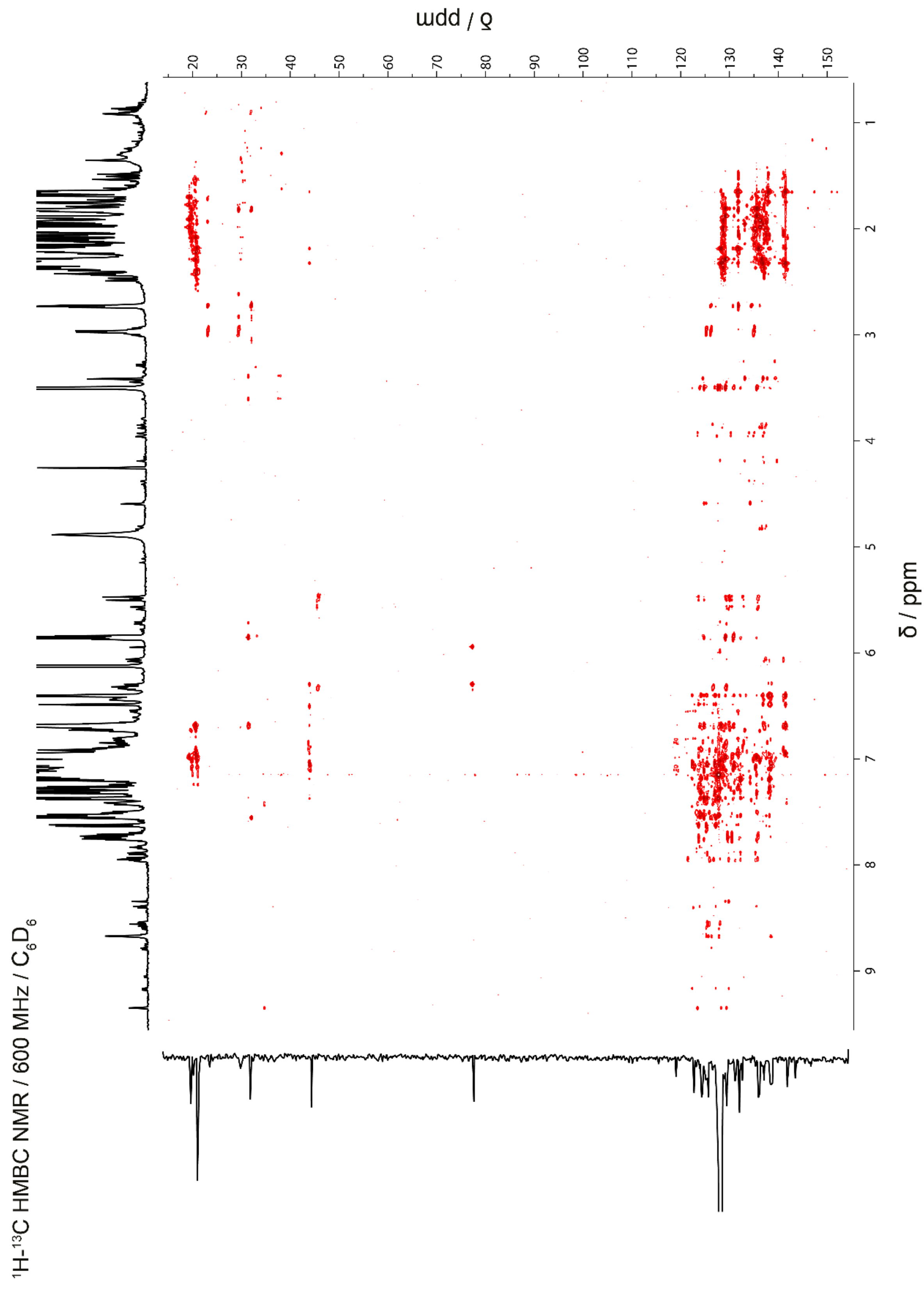

Figure S78. ^{1}H–^{13}C HMBC NMR of **3-Mes** + 0.45 equivalents of CA in C_6D_6. CA = *p*-chloranil.

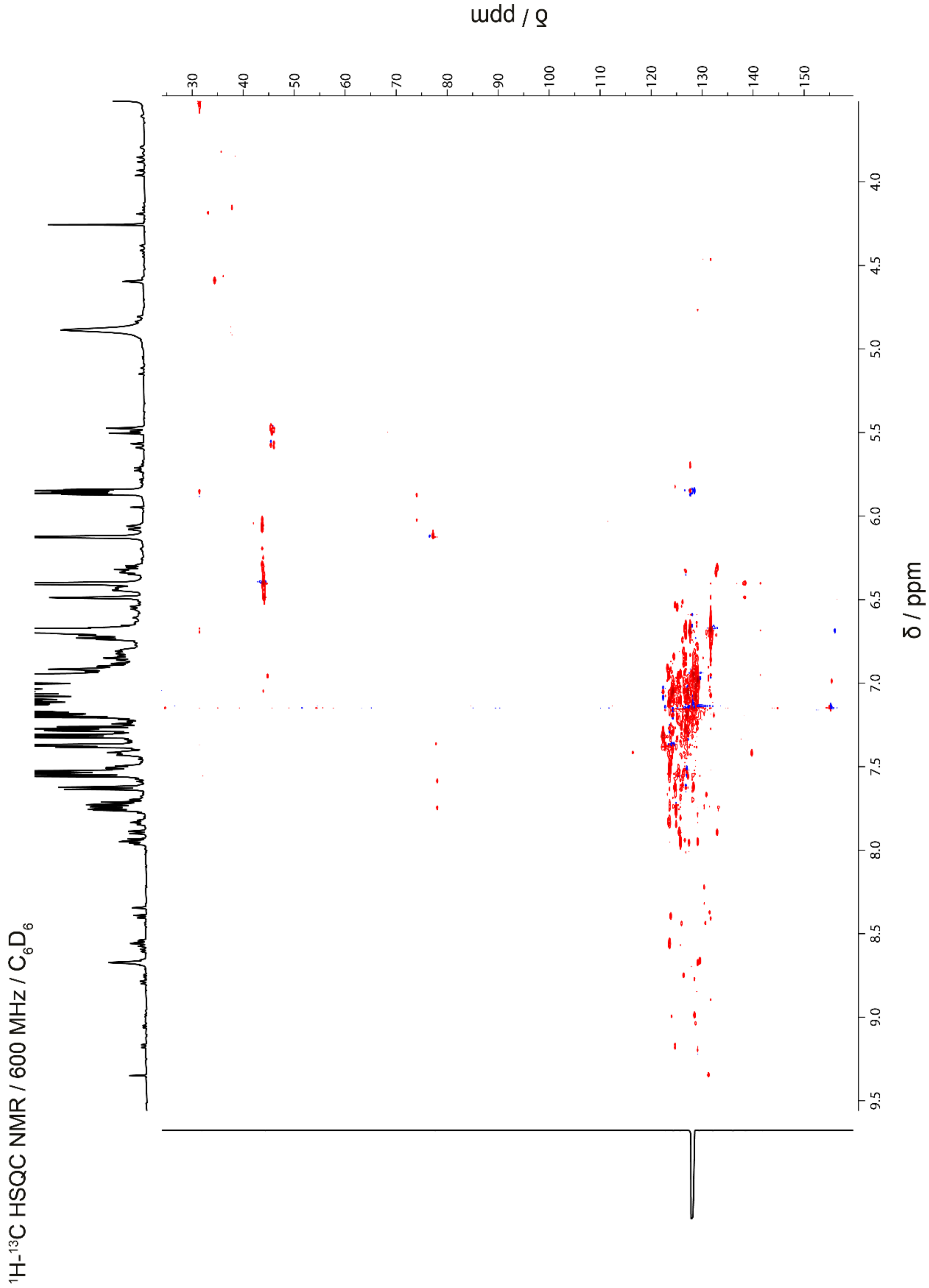

Figure S79. ^{1}H–^{13}C HSQC NMR of **3-Mes** + 0.45 equivalents of CA in C_6D_6. CA = *p*-chloranil.

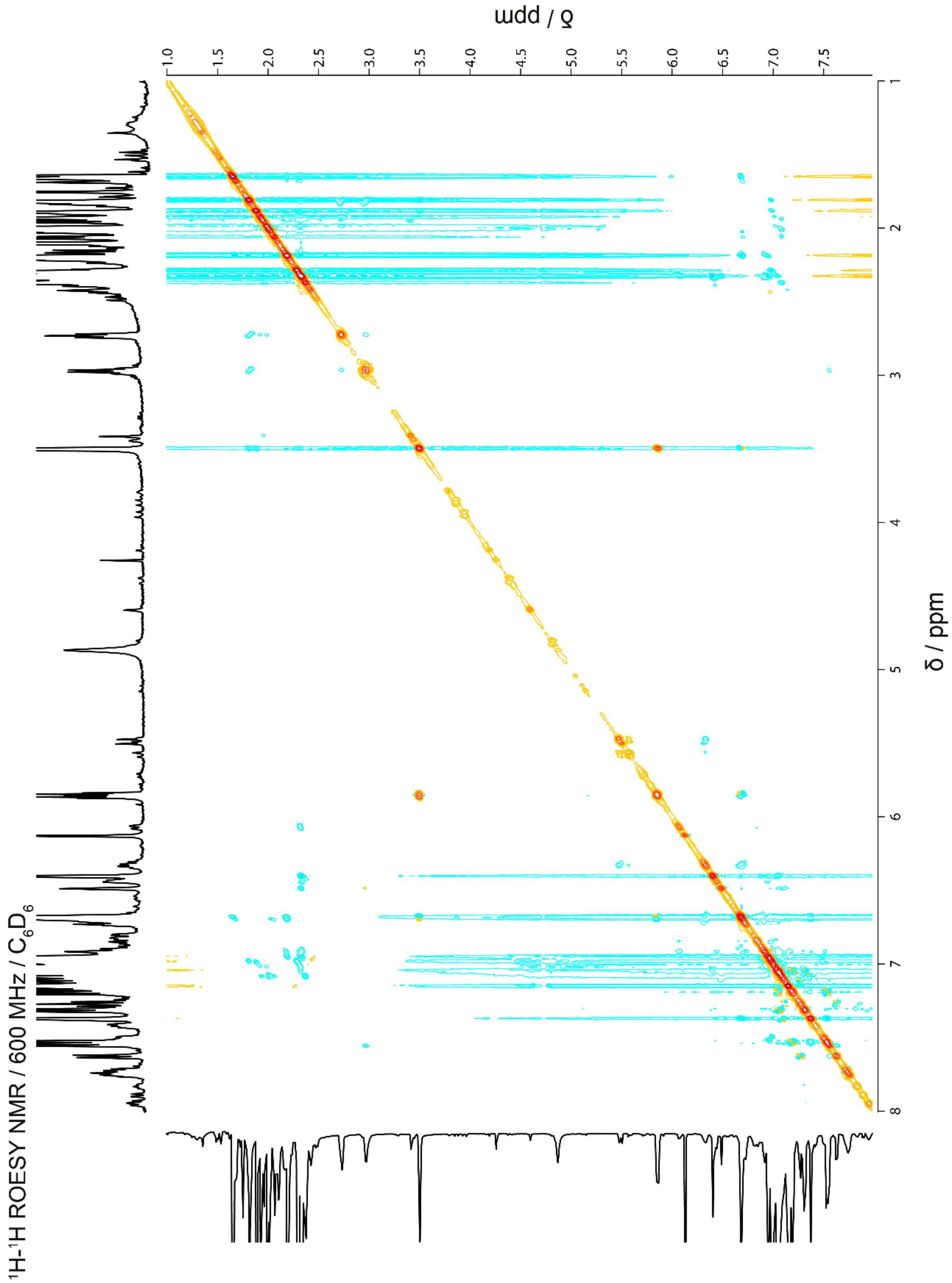

Figure S80. ^1H–^1H ROESY NMR of **3-Mes** + 0.45 equivalents of CA in C_6D_6. CA = *p*-chloranil.

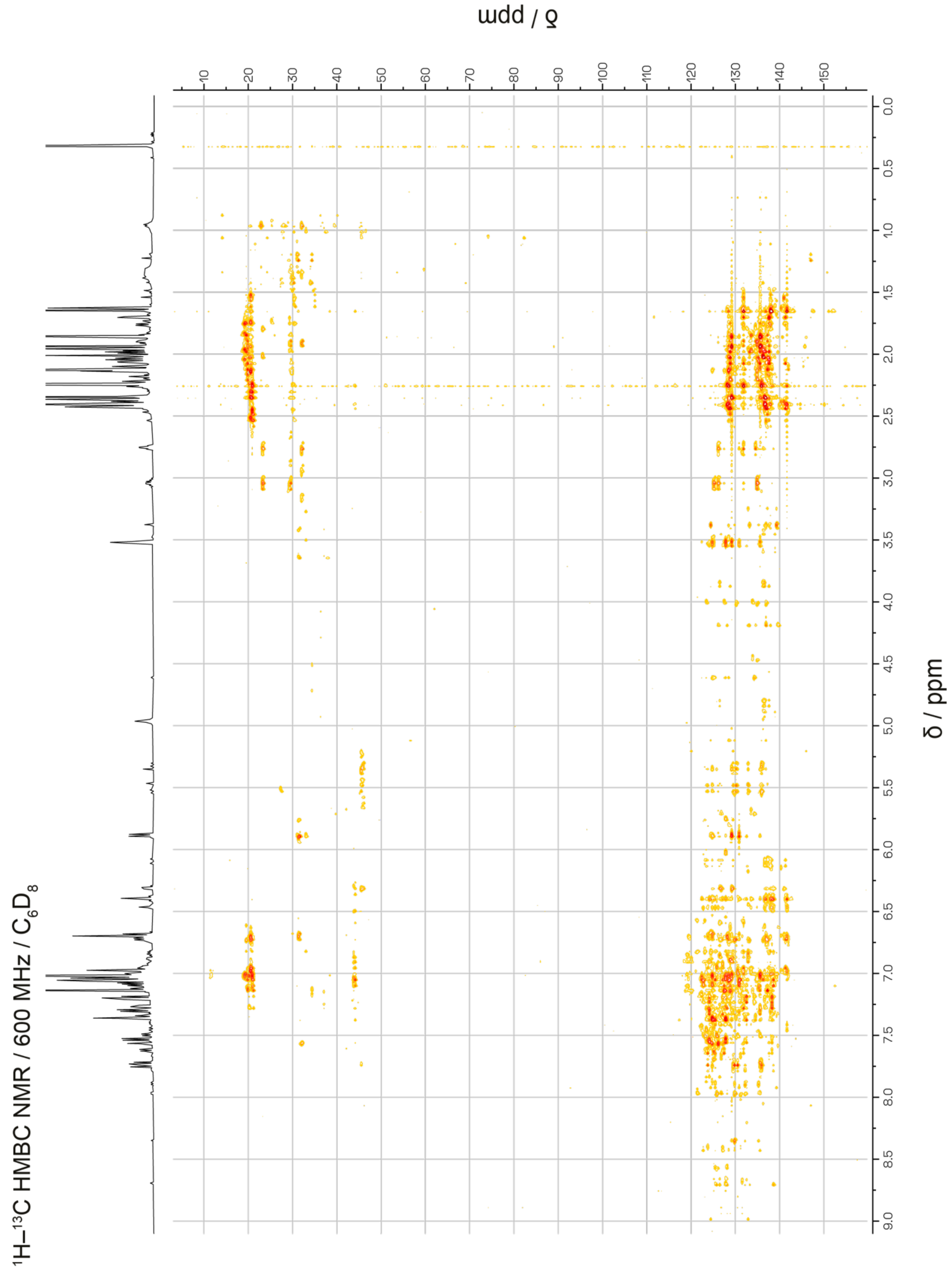

Figure S81. ^{1}H–^{1}H HMBC NMR of **3-Mes** + 0.45 equivalents of CA in C_6D_8. CA = *p*-chloranil.

6. X-Ray Crystallography

1-Mes, CCDC 2503689

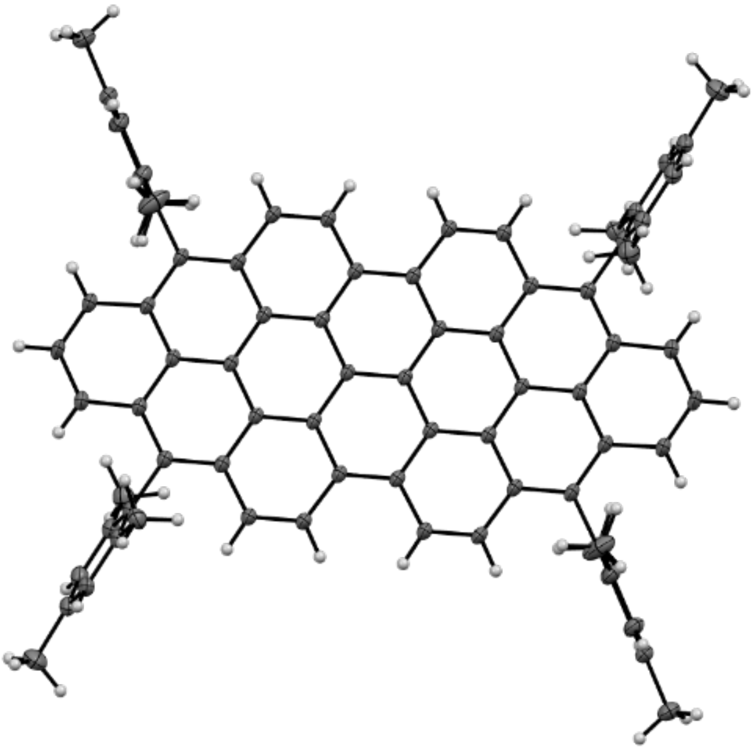

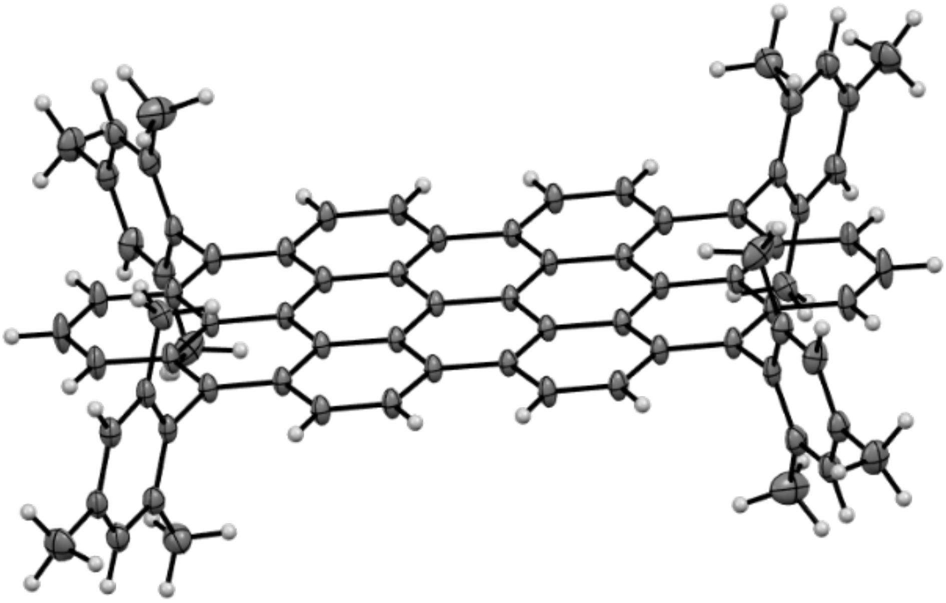

Table S7. Crystal data and structure refinement for compound **1-Mes**.

Empirical formula	$C_{88}H_{74}O_2$
Formula weight	1163.47
Temperature/K	160.0(1)
Crystal system	monoclinic
Space group	$P2_1/n$
a/Å	15.1736(6)
b/Å	11.9967(10)
c/Å	17.8079(10)
α/°	90
β/°	103.798(5)
γ/°	90
Volume/Å^3	3148.1(3)
Z	2
ρ_{calc}g/cm^3	1.227
μ/mm^{-1}	0.544
F(000)	1236.0
Crystal size/mm^3	0.07 × 0.03 × 0.01
Radiation	Cu Kα (λ = 1.54184)
2Θ range for data collection/°	6.89 to 148.998
Index ranges	-18 ≤ h ≤ 13, -14 ≤ k ≤ 14, -22 ≤ l ≤ 22
Reflections collected	31461
Independent reflections	6221 [R_{int} = 0.0947, R_{sigma} = 0.0635]
Data/restraints/parameters	6221/336/458
Goodness-of-fit on F^2	1.014
Final R indexes [I>=2σ (I)]	R_1 = 0.0779, wR_2 = 0.2041
Final R indexes [all data]	R_1 = 0.1769, wR_2 = 0.2645
Largest diff. peak/hole / e Å^{-3}	0.36/-0.22

9a, CCDC 2503690

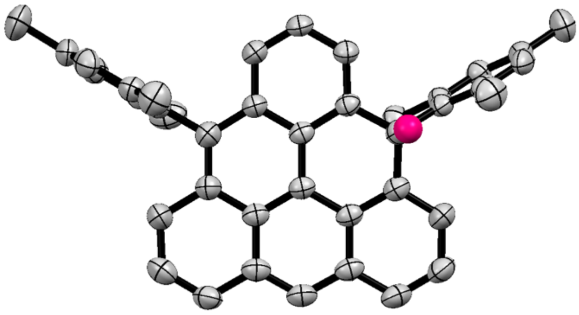

Table S8. Crystal data and structure refinement for **9a**.

Empirical formula	$C_{40}H_{33}$
Formula weight	513.66
Temperature/K	160.0(1)
Crystal system	monoclinic
Space group	P21/c
a/Å	15.0982(6)
b/Å	21.2080(8)
c/Å	8.9631(3)
α/°	90
β/°	104.048(4)
γ/°	90
Volume/Å3	2784.17(18)
Z	4
ρcalcg/cm3	1.225
μ/mm-1	0.520
F(000)	1092.0
Crystal size/mm3	0.11 × 0.05 × 0.02
Radiation	Cu Kα (λ = 1.54184)
2Θ range for data collection/°	7.336 to 136.794
Index ranges	-18 ≤ h ≤ 17, -25 ≤ k ≤ 25, -10 ≤ l ≤ 10
Reflections collected	9285
Independent reflections	9285 [Rint = ?, Rsigma = 0.0226]
Data/restraints/parameters	9285/0/368
Goodness-of-fit on F2	1.061
Final R indexes [I>=2σ (I)]	R1 = 0.0617, wR2 = 0.1677
Final R indexes [all data]	R1 = 0.0851, wR2 = 0.1826
Largest diff. peak/hole / e Å-3	0.42/-0.19

7. Cartesian Coordinates

3a, M06-2X/6-31G(d), Superfine integration grid, E_{el} = –1544.18019411 Hartree

6	2.571885	4.134235	0.054792
6	3.749229	3.463446	-0.180555
6	3.732106	2.084902	-0.453901
6	2.543867	1.379524	-0.497187
6	1.324737	2.051182	-0.239167
6	1.342070	3.441945	0.035988
6	2.555320	-0.088560	-0.895405
6	1.224459	-0.801086	-0.716769
6	0.037362	-0.076986	-0.439803
6	0.067897	1.342940	-0.217931
6	1.163472	-2.169187	-0.891355
6	-0.059803	-2.855562	-0.805129
6	-1.223108	-2.169504	-0.546097
6	-1.199596	-0.767219	-0.357508
6	-1.120846	2.029566	0.054636
6	-1.083131	3.451946	0.300194
6	0.110036	4.113586	0.290938
6	-2.406014	-0.033373	-0.087476
6	-2.371785	1.324034	0.106265
6	-3.663869	2.065744	0.384073
6	-3.511911	3.538561	0.606077
6	-2.332890	4.161128	0.568302
6	3.717484	-0.848771	-0.265114
6	-3.707563	-0.764921	-0.017451
6	4.786894	-1.291171	-1.058786
6	5.850044	-1.973848	-0.459897
6	5.881454	-2.227556	0.906332
6	4.810386	-1.777555	1.678954
6	3.730722	-1.096663	1.122196
6	-4.153394	-1.281177	1.209289
6	-5.374842	-1.952504	1.258247
6	-6.162125	-2.123379	0.119957
6	-5.693724	-1.613802	-1.090486
6	-4.477270	-0.937026	-1.178211
6	4.829269	-1.048618	-2.551177
6	7.033256	-2.961536	1.544436
6	2.599111	-0.661185	2.021318

6	-3.980950	-0.414026	-2.503046
6	-3.309742	-1.125737	2.449959
6	-7.495316	-2.823076	0.200352
1	2.570339	5.200483	0.264374
1	4.696261	3.993628	-0.156445
1	4.667408	1.559070	-0.627342
1	2.722337	-0.082524	-1.979748
1	2.080087	-2.719787	-1.087680
1	-0.082226	-3.931974	-0.945202
1	-2.171939	-2.693402	-0.481778
1	0.134273	5.183619	0.486377
1	-4.163028	1.615905	1.255393
1	-4.369008	1.892178	-0.442893
1	-4.419904	4.100874	0.807819
1	-2.266009	5.232790	0.739106
1	6.675325	-2.312284	-1.083030
1	4.815756	-1.962099	2.751923
1	-5.717910	-2.356469	2.208916
1	-6.288209	-1.750700	-1.991861
1	4.786496	0.019407	-2.793252
1	3.992948	-1.533997	-3.067064
1	5.754107	-1.446956	-2.975114
1	7.784107	-3.243290	0.801808
1	6.691404	-3.874875	2.042308
1	7.523793	-2.342129	2.302505
1	2.900598	-0.728769	3.069781
1	1.717004	-1.295423	1.880357
1	2.291236	0.370063	1.823380
1	-4.699871	-0.620599	-3.299627
1	-3.025231	-0.877354	-2.772206
1	-3.807958	0.667337	-2.467731
1	-3.798958	-1.579414	3.315348
1	-3.120492	-0.070389	2.675982
1	-2.330804	-1.600220	2.318776
1	-7.499093	-3.580036	0.989518
1	-7.743583	-3.313450	-0.744943
1	-8.298351	-2.111468	0.423096

3b, M06-2X/6-31G(d), Superfine integration grid, E_{el} = –1544.17866572 Hartree

6	-1.242465	-2.153829	0.553735
6	-0.081879	-2.846196	0.816596
6	1.142278	-2.166215	0.901054
6	1.208688	-0.796991	0.720880
6	0.027116	-0.069231	0.442800
6	-1.212999	-0.753818	0.361314
6	2.545048	-0.093958	0.896894
6	2.544094	1.372060	0.493173
6	1.325519	2.051017	0.236979
6	0.067415	1.355581	0.219179
6	3.734180	2.069452	0.441384
6	3.758298	3.449028	0.162617
6	2.585887	4.126111	-0.069148
6	1.350361	3.440467	-0.042434
6	-1.116807	2.051203	-0.051011
6	-2.374235	1.342079	-0.106914
6	-2.420436	-0.016266	0.085969
6	0.122365	4.116605	-0.291563
6	-1.077059	3.468180	-0.292746
6	-2.344644	4.253416	-0.553083
6	-3.589633	3.423551	-0.594650
6	-3.594231	2.104475	-0.390598
6	-3.715703	-0.760382	0.012726
6	3.701568	-0.864468	0.268743
6	4.770697	-1.307156	1.062723
6	5.829443	-1.998019	0.465586
6	5.856468	-2.260314	-0.899190
6	4.786549	-1.808653	-1.672318
6	3.711155	-1.119564	-1.117210
6	-4.488580	-0.936388	1.170988
6	-5.683995	-1.649895	1.085945
6	-6.129471	-2.190959	-0.119444
6	-5.343532	-2.007991	-1.256639
6	-4.142709	-1.300170	-1.210638
6	-7.440017	-2.932605	-0.196598
6	-4.028740	-0.358811	2.485799
6	-3.310905	-1.113767	-2.454526
6	4.817869	-1.054856	2.553342
6	2.580866	-0.682017	-2.017051
6	6.999296	-3.011286	-1.533634
1	-2.192818	-2.674429	0.490235
1	-0.110750	-3.921948	0.960261
1	2.056981	-2.719084	1.099760
1	2.712048	-0.086044	1.981256
1	4.667639	1.539492	0.612221
1	4.708886	3.972545	0.132418
1	2.589822	5.191743	-0.281962
1	0.156283	5.187181	-0.487677
1	-2.243791	4.805196	-1.498145
1	-2.455457	5.029007	0.218546
1	-4.522347	3.942706	-0.799694
1	-4.526705	1.549302	-0.431074
1	6.654973	-2.335785	1.088767
1	4.789574	-1.997928	-2.744462
1	-6.281038	-1.788713	1.985336
1	-5.672139	-2.429107	-2.204963
1	-7.673940	-3.424288	0.751705
1	-7.419135	-3.694450	-0.980777
1	-8.264730	-2.247986	-0.424721
1	-3.964392	0.733662	2.434033
1	-3.030412	-0.725062	2.748785
1	-4.717258	-0.624434	3.291756
1	-3.762141	-1.627037	-3.307234
1	-2.297309	-1.504617	-2.312535
1	-3.211130	-0.051894	-2.704842
1	4.778393	0.014869	2.788381
1	5.742870	-1.452738	2.977353
1	3.981775	-1.534727	3.074724
1	2.882202	-0.752343	-3.065387
1	2.276129	0.350499	-1.820875
1	1.696745	-1.313266	-1.875140
1	7.792851	-3.211727	-0.809208
1	7.432811	-2.443271	-2.362958
1	6.662794	-3.971859	-1.938064

3c, M06-2X/6-31G(d), Superfine integration grid, E_{el} = –1544.18529678 Hartree

6	3.701202	2.053588	-0.040944
6	3.717055	3.430313	-0.058530
6	2.511808	4.147358	-0.041639
6	1.294964	3.493689	-0.008866
6	1.258672	2.079641	0.001439
6	2.475284	1.350685	-0.010762
6	0.023744	4.305386	0.035596
6	-1.258880	3.512317	-0.006918
6	-1.239224	2.095487	0.002778
6	0.002448	1.369067	0.015591
6	-2.466844	4.178434	-0.037243
6	-3.682562	3.474838	-0.051579
6	-3.684080	2.099533	-0.034548
6	-2.464803	1.382251	-0.008071
6	-0.007156	-0.028614	0.025364
6	1.242866	-0.756467	0.027684
6	2.442898	-0.089585	0.008056
6	-2.445967	-0.054585	0.008479
6	-1.258435	-0.740405	0.027125
6	-1.288124	-2.254323	0.045216
6	0.056754	-2.906317	0.055745
6	1.201659	-2.221003	0.047223
6	3.738939	-0.836145	0.003086
6	-3.740571	-0.801944	0.004999
6	4.379631	-1.133675	1.216099
6	5.597541	-1.812699	1.190531
6	6.192861	-2.203545	-0.008329
6	5.541213	-1.893988	-1.201449
6	4.322154	-1.216382	-1.215600
6	-4.359530	-1.134199	1.220143
6	-5.562270	-1.839815	1.197455
6	-6.164246	-2.221844	-0.001039
6	-5.538990	-1.869606	-1.196496
6	-4.335673	-1.164393	-1.213208
6	7.494618	-2.964427	-0.012751
6	3.754750	-0.723147	2.525569
6	3.634734	-0.896396	-2.518997
6	-3.688549	-0.776952	-2.519412
6	-3.738138	-0.713871	2.528672
6	-7.447325	-3.013764	-0.002988
1	4.629920	1.491805	-0.051453
1	4.662364	3.963564	-0.084338
1	2.529396	5.234849	-0.051893
1	0.028624	5.025197	-0.792918
1	0.029208	4.913587	0.950430
1	-2.472852	5.266055	-0.047161
1	-4.620837	4.020546	-0.074682
1	-4.619044	1.547596	-0.042516
1	-1.865131	-2.619460	-0.817792
1	-1.868568	-2.598393	0.914655
1	0.076049	-3.993078	0.070812
1	2.153171	-2.744173	0.055132
1	6.094858	-2.040364	2.131552
1	5.994255	-2.185303	-2.147236
1	-6.043724	-2.093564	2.140060
1	-6.001922	-2.146833	-2.141719
1	8.116623	-2.691363	0.844214
1	8.064927	-2.769582	-0.925171
1	7.317197	-4.044518	0.040375
1	2.776364	-1.197363	2.659420
1	3.592098	0.359639	2.561695
1	4.391501	-1.004415	3.367960
1	4.244236	-1.210571	-3.369889
1	3.441964	0.178124	-2.609097
1	2.664674	-1.401424	-2.585433
1	-4.272993	-1.139569	-3.368531
1	-2.674702	-1.184559	-2.600533
1	-3.597917	0.311798	-2.602620
1	-2.725474	-1.117446	2.639167
1	-4.338300	-1.056769	3.375016
1	-3.650738	0.376756	2.586966
1	-8.067661	-2.766168	0.862882
1	-7.243424	-4.089782	0.035425
1	-8.030711	-2.821797	-0.907672

syn-**3d**, M06-2X/6-31G(d), Superfine integration grid, E_{el} = –1544.17127835 Hartree

6	3.646965	-2.152304	-0.141052	6	-2.396561	0.487369	1.972826
6	3.637772	-3.497921	0.315426	6	-4.854401	1.054428	-2.462305
6	2.455480	-4.144128	0.514882	6	-6.611741	3.199399	1.732922
6	1.215674	-3.478776	0.270365	1	4.597711	-1.643099	-0.277642
6	1.221296	-2.121490	-0.198835	1	4.580403	-4.001352	0.506572
6	2.484968	-1.474546	-0.398913	1	2.428997	-5.171918	0.866341
6	0.000021	-4.123949	0.489574	1	0.000029	-5.152175	0.844531
6	-1.215640	-3.478784	0.270392	1	-2.428939	-5.171931	0.866402
6	-1.221282	-2.121499	-0.198811	1	-4.580361	-4.001373	0.506702
6	0.000002	-1.452045	-0.428704	1	-4.597703	-1.643120	-0.277504
6	-2.455437	-4.144143	0.514943	1	2.862803	-0.174137	-1.991049
6	-3.637737	-3.497939	0.315523	1	-2.862834	-0.174157	-1.990995
6	-3.646950	-2.152323	-0.140955	1	-2.145503	2.532863	-1.454911
6	-2.484963	-1.474563	-0.398860	1	-0.000032	3.746125	-1.747098
6	-0.000007	-0.038345	-0.846650	1	2.145451	2.532898	-1.454904
6	1.212422	0.652844	-1.020202	1	6.489145	2.482909	-0.898664
6	2.553076	-0.060359	-0.944663	1	4.411325	2.029328	2.811236
6	-2.553091	-0.060378	-0.944614	1	-6.489699	2.482060	-0.898362
6	-1.212444	0.652832	-1.020183	1	-4.411479	2.029016	2.811381
6	-1.200503	2.008216	-1.342262	1	7.394133	3.518994	1.039741
6	-0.000026	2.688841	-1.500948	1	7.089887	2.643287	2.547336
6	1.200461	2.008231	-1.342265	1	6.161476	4.094693	2.171394
6	3.619063	0.780202	-0.248307	1	2.514447	-0.591055	2.124643
6	-3.619067	0.780182	-0.248238	1	2.382221	0.968098	2.954248
6	4.696771	1.303618	-0.978768	1	1.419555	0.643652	1.504683
6	5.657202	2.080954	-0.324011	1	3.997498	1.431170	-3.033596
6	5.575497	2.354878	1.036099	1	4.953483	-0.012673	-2.688842
6	4.496227	1.826191	1.745017	1	5.748326	1.556844	-2.839415
6	3.520258	1.044154	1.132753	1	-2.514356	-0.591020	2.124678
6	-4.696973	1.303326	-0.978621	1	-2.382150	0.968140	2.954279
6	-5.657501	2.080480	-0.323812	1	-1.419507	0.643716	1.504697
6	-5.575633	2.354626	1.036254	1	-4.953692	-0.013359	-2.688403
6	-4.496300	1.826035	1.745127	1	-3.998123	1.430656	-3.033594
6	-3.520217	1.044173	1.132801	1	-5.748942	1.555943	-2.839116
6	6.612481	3.198264	1.733143	1	-7.403676	3.503898	1.043881
6	2.396623	0.487331	1.972793	1	-6.163237	4.105823	2.152938
6	4.853972	1.055048	-2.462532	1	-7.075208	2.652496	2.560446

anti-**3d**, M06-2X/6-31G(d), Superfine integration grid, E_{el} = –1544.17110190 Hartree

6	-0.371068	3.627672	2.044251
6	-0.430426	3.612201	3.464510
6	-0.302931	2.438352	4.142949
6	-0.136210	1.208130	3.436741
6	-0.100332	1.217058	2.001459
6	-0.191471	2.477704	1.322265
6	-0.000000	0.000000	4.118262
6	0.136210	-1.208130	3.436741
6	0.100332	-1.217058	2.001459
6	-0.000000	0.000000	1.293938
6	0.302931	-2.438352	4.142949
6	0.430426	-3.612201	3.464510
6	0.371068	-3.627672	2.044251
6	0.191471	-2.477704	1.322265
6	0.000000	-0.000000	-0.179647
6	-0.031727	1.211287	-0.891639
6	-0.000000	2.554073	-0.182896
6	-0.000000	-2.554073	-0.182896
6	0.031727	-1.211287	-0.891639
6	0.028021	-1.200163	-2.284727
6	0.000000	-0.000000	-2.984150
6	-0.028021	1.200163	-2.284727
6	-0.920514	3.583153	-0.830684
6	0.920514	-3.583153	-0.830684
6	-0.395729	4.781767	-1.337638
6	-1.258208	5.719792	-1.913164
6	-2.628306	5.501037	-1.997116
6	-3.131560	4.305696	-1.482748
6	-2.307271	3.343980	-0.904068
6	2.307271	-3.343980	-0.904068
6	3.131560	-4.305696	-1.482748
6	2.628306	-5.501037	-1.997116
6	1.258208	-5.719792	-1.913164
6	0.395729	-4.781767	-1.337638
6	-3.550803	6.516947	-2.621190
6	1.082220	5.097076	-1.274826
6	-2.924882	2.066726	-0.388432
6	-1.082220	-5.097076	-1.274826
6	2.924882	-2.066726	-0.388432
6	3.550803	-6.516947	-2.621190
1	-0.468532	4.574242	1.519154
1	-0.569436	4.546052	4.000358
1	-0.331336	2.410572	5.228767
1	-0.000000	0.000000	5.206028
1	0.331336	-2.410572	5.228767
1	0.569436	-4.546052	4.000358
1	0.468532	-4.574242	1.519154
1	1.029215	2.910350	-0.318034
1	-1.029215	-2.910350	-0.318034
1	0.061187	-2.145800	-2.819673
1	0.000000	-0.000000	-4.069752
1	-0.061187	2.145800	-2.819673
1	-0.842597	6.647166	-2.301964
1	-4.202506	4.115972	-1.532235
1	4.202506	-4.115972	-1.532235
1	0.842597	-6.647166	-2.301964
1	-3.005280	7.413992	-2.925126
1	-4.336129	6.819324	-1.920870
1	-4.045924	6.107165	-3.507862
1	1.458942	5.082642	-0.245732
1	1.276918	6.090992	-1.684628
1	1.677554	4.379452	-1.850829
1	-4.008404	2.179250	-0.298315
1	-2.530559	1.786941	0.593262
1	-2.725939	1.230602	-1.067892
1	-1.458942	-5.082642	-0.245732
1	-1.276918	-6.090992	-1.684628
1	-1.677554	-4.379452	-1.850829
1	2.530559	-1.786941	0.593262
1	2.725939	-1.230602	-1.067892
1	4.008404	-2.179250	-0.298315
1	4.045924	-6.107165	-3.507862
1	3.005280	-7.413992	-2.925126
1	4.336129	-6.819324	-1.920870

3e, M06-2X/6-31G(d), Superfine integration grid, E_{el} = –1544.17656964 Hartree

6 -3.544850 2.078679 0.535418
6 -3.488333 3.411090 0.817784
6 -2.256730 4.109544 0.724413
6 -1.104653 3.473518 0.350135
6 -1.118680 2.066652 0.069914
6 -2.366037 1.360662 0.157809
6 0.163313 4.268520 0.180436
6 1.421249 3.453492 0.034897
6 1.362761 2.079891 -0.253159
6 0.066730 1.374810 -0.259864
6 2.652162 4.096018 0.128750
6 3.834701 3.394352 -0.068405
6 3.782089 2.040866 -0.379222
6 2.559759 1.379590 -0.480905
6 0.015068 -0.010640 -0.516639
6 -1.240131 -0.708576 -0.450816
6 -2.415604 -0.017719 -0.112168
6 2.552066 -0.072874 -0.926168
6 1.201744 -0.758305 -0.820621
6 1.120784 -2.104223 -1.057940
6 -0.121747 -2.788319 -1.014734
6 -1.268411 -2.114092 -0.718856
6 -3.715558 -0.750502 -0.024714
6 3.668244 -0.884766 -0.275589
6 -4.083105 -1.379525 1.173420
6 -5.301282 -2.059093 1.236683
6 -6.158281 -2.127773 0.141119
6 -5.772557 -1.494118 -1.041478
6 -4.565270 -0.806483 -1.142806
6 3.619352 -1.176089 1.102409
6 4.658058 -1.902623 1.679041
6 5.746547 -2.358612 0.934827
6 5.776090 -2.062472 -0.422876
6 4.756081 -1.332507 -1.040778
6 -4.170476 -0.128468 -2.430536
6 -3.172681 -1.321560 2.374194
6 -7.470031 -2.867438 0.218578
6 4.864513 -1.046189 -2.522125
6 2.463521 -0.740075 1.969849
6 6.848818 -3.149550 1.592046
1 -4.483018 1.536722 0.599880
1 -4.385465 3.946332 1.113348
1 -2.228503 5.174423 0.943496
1 0.061460 4.892752 -0.719655
1 0.272912 4.972043 1.014204
1 2.677201 5.158729 0.357995
1 4.792330 3.898861 0.014169
1 4.700107 1.480617 -0.535211
1 2.767029 -0.031190 -2.001252
1 2.030689 -2.660802 -1.268300
1 -0.149749 -3.855478 -1.212552
1 -2.222057 -2.630543 -0.676870
1 -5.585699 -2.546038 2.167333
1 -6.430172 -1.537569 -1.908025
1 4.616168 -2.119406 2.745154
1 6.616250 -2.404376 -1.023856
1 -4.923979 -0.291357 -3.205037
1 -3.210552 -0.508426 -2.796383
1 -4.052509 0.951141 -2.286089
1 -2.206212 -1.790429 2.157815
1 -3.619855 -1.835173 3.228828 1
-2.967548 -0.284914 2.662307
1 -7.636438 -3.275934 1.218557
1 -7.496247 -3.698702 -0.493805
1 -8.309963 -2.207302 -0.021143
1 5.790132 -1.465437 -2.923599
1 4.869268 0.029449 -2.732141
1 4.031885 -1.484552 -3.084056
1 2.185376 0.302851 1.790069
1 2.722395 -0.843930 3.026705
1 1.574296 -1.350475 1.776855
1 7.274915 -2.603407 2.439774
1 7.656573 -3.364808 0.887856
1 6.473570 -4.104223 1.975684

9a, M06-2X/6-31G(d), Superfine integration grid, E_{el} = –1543.56648873 Hartree

6	3.566552	2.121870	-0.348014
6	3.514390	3.498003	-0.538697
6	2.307787	4.176736	-0.494155
6	1.091046	3.483872	-0.244328
6	1.124456	2.060107	-0.028813
6	2.385929	1.368009	-0.095847
6	-0.131104	4.148458	-0.213826
6	-1.339845	3.466620	0.033644
6	-1.314501	2.062253	0.277498
6	-0.060957	1.360252	0.233427
6	-2.581873	4.152641	0.047517
6	-3.747100	3.472445	0.291650
6	-3.718399	2.084697	0.538754
6	-2.529442	1.383047	0.538985
6	-0.018181	-0.065409	0.421073
6	1.228086	-0.751739	0.320435
6	2.427144	-0.024468	0.074401
6	-2.533265	-0.094599	0.892104
6	-1.202777	-0.801729	0.673529
6	-1.141297	-2.176106	0.794232
6	0.085666	-2.858126	0.693901
6	1.248216	-2.164611	0.468645
6	-3.698731	-0.845610	0.257335
6	3.729118	-0.750611	-0.009112
6	4.488787	-0.960149	1.152515
6	5.698889	-1.647027	1.055579
6	6.172163	-2.127708	-0.164571
6	5.399592	-1.911159	-1.305227
6	4.185009	-1.228453	-1.248209
6	-4.649588	-1.488981	1.064380
6	-5.703456	-2.183469	0.462811
6	-5.838163	-2.258052	-0.918654
6	-4.881036	-1.614772	-1.703941
6	-3.817904	-0.909645	-1.145440
6	-2.836344	-0.220370	-2.061886
6	-4.568162	-1.459445	2.574437
6	-6.979723	-3.006057	-1.558829
6	3.996493	-0.450599	2.483621
6	3.366207	-1.006669	-2.494875
6	7.497834	-2.840496	-0.253836
1	4.516809	1.600027	-0.392661
1	4.432746	4.044781	-0.729592
1	2.270439	5.250729	-0.651404
1	-0.157879	5.221926	-0.386424
1	-2.588496	5.222789	-0.140473
1	-4.697374	3.997317	0.297598
1	-4.647509	1.551631	0.723725
1	-2.685162	-0.118548	1.978409
1	-2.057385	-2.736204	0.962146
1	0.107599	-3.938724	0.796342
1	2.197852	-2.685076	0.393987
1	6.285614	-1.812122	1.957268
1	5.750233	-2.285099	-2.265363
1	-6.435074	-2.680729	1.096350
1	-4.964238	-1.664100	-2.788353
1	-2.956126	-0.581770	-3.086454
1	-2.993934	0.863684	-2.064372
1	-1.799264	-0.395916	-1.760050
1	-3.626853	-1.884200	2.941432
1	-4.640718	-0.438688	2.966794
1	-5.384866	-2.039313	3.010817
1	-7.596688	-3.506309	-0.807997
1	-7.625019	-2.328084	-2.127421
1	-6.611892	-3.765784	-2.255962
1	3.866279	0.636908	2.463748
1	3.022574	-0.884711	2.735038
1	4.700214	-0.698485	3.282004
1	3.851053	-1.453968	-3.366040
1	2.367399	-1.444995	-2.392588
1	3.227970	0.062755	-2.688312
1	7.741557	-3.343944	0.685692
1	7.492979	-3.588558	-1.051494
1	8.308063	-2.134946	-0.469667

9b, M06-2X/6-31G(d), Superfine integration grid, E_{el} = –1543.57005822 Hartree

6	3.674728	2.120818	-0.000001
6	3.642053	3.510390	-0.000002
6	2.432419	4.191721	-0.000002
6	1.213570	3.484529	-0.000001
6	1.231652	2.066874	-0.000001
6	2.478984	1.366905	-0.000001
6	-0.044491	4.155692	-0.000001
6	-1.231107	3.473267	-0.000001
6	-1.232578	2.036335	-0.000001
6	0.000917	1.345255	-0.000000
6	-2.511663	4.179619	-0.000001
6	-3.678597	3.532823	-0.000001
6	-3.785651	2.038299	-0.000000
6	-2.459036	1.306309	-0.000000
6	0.019299	-0.076674	-0.000000
6	1.268475	-0.780206	0.000000
6	2.489437	-0.054692	-0.000000
6	-2.444337	-0.071890	0.000000
6	-1.204160	-0.794547	0.000000
6	-1.170980	-2.208202	0.000001
6	0.040623	-2.884814	0.000001
6	1.246064	-2.192940	0.000001
6	3.788983	-0.786721	-0.000000
6	-3.732977	-0.828475	0.000001
6	4.397166	-1.131973	-1.217444
6	5.610210	-1.819734	-1.197238
6	6.233576	-2.169636	0.000000
6	5.610213	-1.819728	1.197239
6	4.397170	-1.131966	1.217444
6	-4.335549	-1.183182	1.217010
6	-5.544419	-1.878502	1.197199
6	-6.167264	-2.229585	0.000001
6	-5.544419	-1.878503	-1.197197
6	-4.335549	-1.183183	-1.217008
6	-3.670261	-0.832348	-2.524457
6	-3.670261	-0.832347	2.524458
6	-7.490970	-2.951526	0.000001
6	3.739717	-0.765543	-2.524082
6	3.739724	-0.765530	2.524082
6	7.561199	-2.884382	0.000001
1	4.625240	1.596513	-0.000001
1	4.574252	4.066934	-0.000002
1	2.410848	5.277915	-0.000002
1	-0.050956	5.243658	-0.000002
1	-2.479579	5.266348	-0.000002
1	-4.611273	4.090901	-0.000001
1	-4.382039	1.715152	0.866558
1	-4.382039	1.715152	-0.866559
1	-2.107226	-2.757735	0.000001
1	0.047852	-3.970569	0.000001
1	2.187633	-2.733146	0.000001
1	6.079278	-2.090229	-2.141379
1	6.079284	-2.090217	2.141380
1	-6.009169	-2.156874	2.141195
1	-6.009169	-2.156876	-2.141193
1	-4.272668	-1.167122	-3.372427
1	-3.515162	0.248300	-2.618024
1	-2.682023	-1.299888	-2.597481
1	-3.515162	0.248301	2.618025
1	-4.272668	-1.167120	3.372429
1	-2.682023	-1.299886	2.597482
1	-7.598207	-3.583489	0.885911
1	-8.324735	-2.240405	0.000002
1	-7.598208	-3.583488	-0.885908
1	3.605246	0.318632	-2.605323
1	4.340337	-1.102454	-3.372511
1	2.745281	-1.218232	-2.603834
1	4.340345	-1.102438	3.372511
1	3.605256	0.318646	2.605318
1	2.745287	-1.218215	2.603838
1	7.671848	-3.515698	-0.885955
1	8.391045	-2.168687	0.000014
1	7.671837	-3.515717	0.885945

9c, M06-2X/6-31G(d), Superfine integration grid, E_{el} = –1543.56855678 Hartree

6	-3.677726	-2.105610	0.004197
6	-3.651820	-3.497057	0.007060
6	-2.447715	-4.184365	0.008422
6	-1.222452	-3.481667	0.007024
6	-1.233228	-2.064492	0.004227
6	-2.478818	-1.357278	0.002621
6	0.029499	-4.155922	0.008164
6	1.223508	-3.483997	0.006865
6	1.227826	-2.055767	0.004584
6	-0.000396	-1.354897	0.003019
6	2.463624	-1.321524	0.004006
6	2.461206	0.055697	0.001317
6	1.218184	0.783890	-0.001402
6	-0.008702	0.071464	-0.000065
6	1.190951	2.194570	-0.005613
6	-0.018318	2.878761	-0.007609
6	-1.225063	2.193172	-0.005826
6	-1.252695	0.780298	-0.002147
6	-2.480259	0.061869	-0.000616
6	2.520018	-4.264595	0.007394
6	3.752808	-3.413426	0.007968
6	3.720052	-2.078771	0.006625
6	3.743722	0.824461	0.000164
6	-3.774927	0.802502	-0.002592
6	-4.384270	1.142488	-1.221028
6	-5.597411	1.829965	-1.202693
6	-6.217293	2.189353	-0.006362
6	-5.596406	1.838409	1.191764
6	-4.383119	1.150970	1.213832
6	4.350591	1.170299	-1.217506
6	5.542442	1.894212	-1.198904
6	6.142700	2.285666	-0.002644
6	5.523998	1.929279	1.194905
6	4.331690	1.205752	1.216265
6	3.684546	0.827151	2.524593
6	3.723960	0.752644	-2.523947
6	7.413496	3.097187	-0.005477
6	-3.732652	0.762116	-2.526601
6	-3.730419	0.779797	2.521513
6	-7.512438	2.961376	-0.009026
1	-4.625918	-1.577137	0.003119
1	-4.587297	-4.048118	0.008203
1	-2.431319	-5.270632	0.010570
1	0.026972	-5.244779	0.010005
1	2.128927	2.740620	-0.007392
1	-0.019248	3.964502	-0.010761
1	-2.164717	2.736681	-0.007414
1	2.542350	-4.933732	0.879352
1	2.543066	-4.933552	-0.864717
1	4.708631	-3.930997	0.009816
1	4.644524	-1.508813	0.007615
1	-6.070906	2.090122	-2.147541
1	-6.069021	2.105161	2.135188
1	6.014341	2.158528	-2.143413
1	5.981353	2.220964	2.138466
1	4.248455	1.228293	3.370307
1	2.659181	1.208654	2.580896
1	3.626420	-0.261316	2.634334
1	2.694257	1.118311	-2.600763
1	4.292484	1.141643	-3.372248
1	3.682134	-0.338890	-2.609089
1	8.036979	2.854861	-0.870587
1	7.193095	4.169751	-0.049071
1	8.000283	2.918592	0.899740
1	-3.610569	-0.323906	-2.602879
1	-4.330305	1.101876	-3.375987
1	-2.733253	1.203102	-2.609480
1	-4.328069	1.124294	3.368993
1	-3.606966	-0.305556	2.604710
1	-2.731522	1.222539	2.601089
1	-8.118152	2.716887	-0.886031
1	-8.103704	2.747367	0.885542
1	-7.324643	4.040812	-0.029390

9d, M06-2X/6-31G(d), Superfine integration grid, E_{el} = –1543.57250686 Hartree

6	-3.701036	2.069093	-0.046445
6	-3.702913	3.441649	-0.067688
6	-2.487927	4.150570	-0.047953
6	-1.277721	3.488714	-0.008651
6	-1.249260	2.073045	0.002592
6	-2.477077	1.348628	-0.011563
6	0.000000	4.289553	0.048311
6	1.277722	3.488714	-0.008653
6	1.249260	2.073044	0.002590
6	0.000000	1.359512	0.018409
6	2.487927	4.150570	-0.047957
6	3.702913	3.441649	-0.067694
6	3.701036	2.069092	-0.046451
6	2.477077	1.348628	-0.011567
6	0.000000	-0.039996	0.029512
6	-1.244932	-0.768365	0.033098
6	-2.463660	-0.074278	0.009677
6	2.463660	-0.074278	0.009674
6	1.244932	-0.768365	0.033097
6	1.207591	-2.191171	0.054880
6	-0.000000	-2.873705	0.067020
6	-1.207591	-2.191171	0.054880
6	-3.754438	-0.825155	0.004797
6	3.754438	-0.825156	0.004793
6	4.385357	-1.139287	1.219036
6	5.594534	-1.833673	1.194183
6	6.189826	-2.224736	-0.004634
6	5.547604	-1.899304	-1.198698
6	4.337413	-1.206162	-1.214129
6	-4.337427	-1.206141	-1.214126
6	-5.547618	-1.899282	-1.198694
6	-6.189826	-2.224736	-0.004628
6	-5.594522	-1.833694	1.194189
6	-4.385343	-1.139308	1.219041
6	-3.661323	-0.865347	-2.518164
6	-3.760822	-0.726168	2.527887
6	-7.481237	-3.002989	-0.008235
6	3.661295	-0.865393	-2.518166
6	3.760850	-0.726124	2.527881
6	7.481238	-3.002988	-0.008238
1	-4.634087	1.514544	-0.057968
1	-4.642482	3.984713	-0.097928
1	-2.497852	5.238040	-0.059886
1	-0.000000	5.025591	-0.765527
1	0.000001	4.879088	0.975381
1	2.497852	5.238040	-0.059890
1	4.642482	3.984712	-0.097937
1	4.634087	1.514543	-0.057977
1	2.145747	-2.736231	0.060968
1	-0.000000	-3.959360	0.084159
1	-2.145747	-2.736231	0.060967
1	6.084645	-2.073806	2.135862
1	6.000831	-2.190846	-2.144303
1	-6.000855	-2.190807	-2.144298
1	-6.084622	-2.073842	2.135869
1	-3.519378	0.216405	-2.616752
1	-2.668527	-1.324816	-2.576974
1	-4.252678	-1.214207	-3.368277
1	-4.376466	-1.044317	3.372791
1	-2.763792	-1.165223	2.642650
1	-3.639200	0.361372	2.579678
1	-8.057934	-2.810419	-0.917089
1	-7.288894	-4.080841	0.037626
1	-8.103168	-2.743496	0.852960
1	4.252646	-1.214257	-3.368280
1	2.668503	-1.324873	-2.576962
1	3.519338	0.216356	-2.616767
1	3.639239	0.361418	2.579660
1	2.763817	-1.165167	2.642658
1	4.376497	-1.044268	3.372785
1	8.103231	-2.743386	0.852880
1	7.288899	-4.080835	0.037774
1	8.057868	-2.810530	-0.917157

9e, M06-2X/6-31G(d), Superfine integration grid, E_{el} = –1543.56872488 Hartree

6	3.678618	2.083722	0.005772
6	3.678181	3.475487	0.008357
6	2.486612	4.184085	0.008575
6	1.247570	3.506643	0.006391
6	1.241983	2.075761	0.003644
6	2.466370	1.361135	0.002998
6	0.021312	4.199958	0.006951
6	-1.212507	3.524569	0.005169
6	-1.223941	2.091882	0.002726
6	0.002222	1.370832	0.001857
6	-2.444103	4.215377	0.005853
6	-3.645427	3.520003	0.004283
6	-3.663338	2.129966	0.002053
6	-2.456582	1.392615	0.001248
6	-0.007724	-0.042998	-0.000537
6	1.241317	-0.756896	-0.001721
6	2.441221	-0.078327	-0.000194
6	-2.444645	-0.041755	-0.000895
6	-1.255103	-0.739908	-0.001733
6	-1.288999	-2.253508	-0.003684
6	0.057339	-2.906032	-0.005320
6	1.203753	-2.222339	-0.004681
6	3.741256	-0.816957	-0.000822
6	-3.742436	-0.782524	-0.002125
6	4.346886	-1.167291	1.215940
6	5.569414	-1.838472	1.195769
6	6.202754	-2.170157	-0.001254
6	5.585375	-1.808456	-1.198144
6	4.363239	-1.136933	-1.217930
6	-4.350733	-1.125331	-1.219827
6	-5.556828	-1.825261	-1.201215
6	-6.172236	-2.191397	-0.004546
6	-5.557261	-1.828858	1.193165
6	-4.351005	-1.128819	1.214186
6	-3.715192	-0.729409	2.522370
6	-3.714326	-0.722115	-2.526547
6	-7.458792	-2.977622	-0.006649
6	3.679950	-0.821753	2.523480
6	3.714122	-0.757964	-2.525164
6	7.508810	-2.923620	-0.002513
1	4.617266	1.538773	0.005987
1	4.623153	4.010024	0.010360
1	2.489961	5.270501	0.010594
1	0.029345	5.287235	0.008934
1	-2.435643	5.301724	0.007669
1	-4.583402	4.066791	0.004849
1	-4.607766	1.594652	0.000923
1	-1.868330	-2.606572	-0.870229
1	-1.867431	-2.608801	0.862565
1	0.075479	-3.992897	-0.007454
1	2.155280	-2.745659	-0.006467
1	6.039869	-2.106863	2.139816
1	6.068419	-2.053442	-2.142230
1	-6.030070	-2.087370	-2.145669
1	-6.030683	-2.093764	2.136707
1	-4.306138	-1.085598	3.369679
1	-2.701428	-1.134664	2.615535
1	-3.626967	0.360158	2.597129
1	-2.700672	-1.127449	-2.620581
1	-4.305096	-1.075497	-3.375153
1	-3.625676	0.367645	-2.597915
1	-8.069542	-2.736641	-0.881140
1	-7.259222	-4.054838	-0.031573
1	-8.050054	-2.773424	0.890191
1	2.708547	-1.320526	2.612854
1	4.300334	-1.124100	3.370751
1	3.493780	0.255170	2.597916
1	4.339676	-1.051104	-3.371860
1	2.737739	-1.242719	-2.634003
1	3.541873	0.322501	-2.580435
1	8.090958	-2.709912	0.898100
1	7.336812	-4.005407	-0.034614
1	8.116863	-2.661925	-0.87292

8. Supporting References

(1) Grimme, S.; Antony, J.; Ehrlich, S.; Krieg, H. A Consistent and Accurate Ab Initio Parameterization of Density Functional Dispersion Correction (DFT-D) for the 94 Elements H–Pu. *J. Chem. Phys.* **2010**, *132*, 154104, DOI: 10.1063/1.3382344.

(2) Loos, M.; Gerber, C.; Corona, F.; Hollender, J.; Singer, H. Accelerated Isotope Fine Structure Calculation Using Pruned Transition Trees. *Anal. Chem.* **2015**, *87*, 5738–5744, DOI: 10.1021/acs.analchem.5b00941.

(3) Fulmer, G. R.; Miller, A. J. M.; Sherden, N. H.; Gottlieb, H. E.; Nudelman, A.; Stoltz, B. M.; Bercaw, J. E.; Goldberg, K. I. NMR Chemical Shifts of Trace Impurities: Common Laboratory Solvents, Organics, and Gases in Deuterated Solvents Relevant to the Organometallic Chemist. *Organometallics* **2010**, *29*, 2176–2179, DOI: 10.1021/om100106e.

(4) Stoll, S.; Schweiger, A. EasySpin, a Comprehensive Software Package for Spectral Simulation and Analysis in EPR. *J. Magn. Reson.* **2006**, *178*, 42–55, DOI: 10.1016/j.jmr.2005.08.013.

(5) Gaussian 16, Revision C.02. Frisch, M. J.; Trucks, G. W.; Schlegel, H. B.; Scuseria, G. E.; Robb, M. A.; Cheeseman, J. R.; Scalmani, G.; Barone, V.; Petersson, G. A.; Nakatsuji, H.; Li, X.; Caricato, M.; Marenich, A. V.; Bloino, J. ; Janesko, B. G.; Gomperts, R.; Mennucci, B.; Hratchian, H. P.; Ortiz, J. V.; Izmaylov, A. F.; Sonnenberg, J. L.; Williams-Young, D.; Ding, F.; Lipparini, F.; Egidi, F.; Goings, J.; Peng, B.; Petrone, A.; Henderson, T.; Ranasinghe, D.; Zakrzewski, V. G.; Gao, J. ; Rega, N. ; Zheng, G.; Liang, W.; Hada, M.; Ehara, M.; Toyota, K.; Fukuda, R.; Hasegawa, J.; Ishida, M.; Nakajima, T.; Honda, Y.; Kitao, O.; Nakai, H.; Vreven, T.; Throssell, K.; Montgomery Jr., J. A.; Peralta, J. E.; Ogliaro, F.; Bearpark, M. J.; Heyd, J. J.; Brothers, E. N.; Kudin, K. N.; Staroverov, V. N.; Keith, T. A.; Kobayashi, R.; Normand, J.; Raghavachari, K.; Rendell, A. P.; Burant, J. C.; Iyengar, S. S.; Tomasi, J. ; Cossi, M.; Millam, J. M.; Klene, M.; Adamo, ; Cammi, R.; Ochterski, J. W.; Martin, R. L.; Morokuma, K.; Farkas, O.; Foresman J. B.; Fox, D. J. Gaussian, Inc., Wallingford CT, 2016.
